# Machine learning methods for estimating personalized treatment effects—insights on validity from two large trials

**DOI:** 10.1093/aje/kwag065

**Published:** 2026-03-20

**Authors:** Hongruyu Chen, Helena Aebersold, Milo Alan Puhan, Miquel Serra-Burriel

**Affiliations:** Epidemiology, Biostatistics and Prevention Institute, University of Zurich, Zurich, Switzerland; Epidemiology, Biostatistics and Prevention Institute, University of Zurich, Zurich, Switzerland; Epidemiology, Biostatistics and Prevention Institute, University of Zurich, Zurich, Switzerland; Department of Epidemiology, Bloomberg School of Public Health, Johns Hopkins University, Baltimore, Maryland, United States; Epidemiology, Biostatistics and Prevention Institute, University of Zurich, Zurich, Switzerland

**Keywords:** causal machine learning, individualized treatment effects, validation

## Abstract

Machine learning (ML) methods have the potential to improve precision medicine by estimating personalized treatment effects. However, formal validation of these methods remains limited, leaving their reliability in empirical settings largely uncertain. In this study, we evaluated the internal and external validity of 17 causal heterogeneity ML methods—including metalearners, tree-based methods, and deep learning methods—using data from two large randomized controlled trials: the International Stroke Trial (*n* = 19 435) and the Chinese Acute Stroke Trial (*n* = 21 106). We assessed performance using three visual-based metrics and three quantitative metrics. Our analysis found that none of the ML methods consistently demonstrated reliable performance, neither internal nor external. Heterogeneous treatment effects estimated from training data failed to generalize to the test data, even in the absence of distribution shifts. These results raise concerns about the current applicability of ML models in precision medicine and highlight the need for more robust validation techniques to ensure generalizability.

## Introduction

Personalized medicine involves identifying which patients will benefit from a given treatment and those who will not.^1-3^ Traditionally, evidence-based treatment recommendations rely on Randomized Controlled Trials (RCTs), which are considered the gold standard of evidence due to their high internal validity. RCTs investigating therapeutic options primarily estimate average treatment effects (ATEs). While the ATE as an estimand offers a straightforward, easy-to-interpret summary of the overall effect, relying on it for decision-making introduces inherent limitations.^4^ ATEs are not informative about treatment effect variation across patient, temporal, or contextual subgroups.^5^^,^^6^

To address this limitation, numerous causal Machine Learning (ML) methods have emerged in recent years. These methods estimate individualized or subgroup treatment effects by leveraging patient characteristics to provide personalized treatment recommendations.^7-10^ There is a substantial body of literature developing causal ML methods for learning heterogeneous treatment effects (HTE), comparing these methods using synthetic, RCT, or observational data, and applying them in specific contexts.^11-17^

Despite the proliferation of new models, a crucial yet often overlooked question remains: how can these heterogeneous treatment effects be systematically validated? Since individual treatment effects are fundamentally unobservable, direct validation is impossible. However, by grouping patients with similar predicted treatment effects and estimating the subgroup ATE, we can assess the consistency between predicted and observed effects. Several quantitative metrics have been proposed to assess the validity of HTE estimates, including c-for-benefit,^18^ mbcb,^19^ and calibration-based pseudo R-squared.^20^

Despite these possibilities, most studies either stop at reporting the distribution of individualized treatment effect estimates or rely on synthetic data, where the validity of the assumptions is imposed.^11^^,^^13^^,^^21^ Other validation research using observational data suffers from inherent unobservable confounding that is only amplified when creating subgroups.^15^ RCTs, by contrast, offer a unique opportunity for validation because they address confounding by design, providing a cleaner setting to evaluate HTE estimates. Our objective is, therefore, to use large RCTs as a vehicle to examine the broad methodological question of how well causal ML estimates perform under rigorous validation.

This study investigates to what extent estimates of individualized and subgroup treatment effects based on causal ML methods withstand systematic validation. Given that RCTs offer distinct advantages over observational studies,^22^ and that ML methods rely on large datasets, we utilized two large RCTs designed for parallel analysis. First, we estimated individualized treatment effects (ITEs) using a wide range of causal ML models that are used in medical studies. Second, we validated these estimates through internal training-test data splits and external cross-validation, employing established and novel metrics including c-for-benefit,^18^ mbcb,^19^ calibration-based pseudo R-squared,^20^ outcome-ITE plots, ATE-ITE subgroup analysis, and benefit-harm ATE density plots. Furthermore, we conducted simulation studies, with and without unobservables, to explore the fundamental limitations of causal ML methods.

## Methods

### Data

The International Stroke Trial (IST)^23^ and the Chinese Acute Stroke Trial (CAST)^24^ were two large, parallel-group randomized controlled trials that investigated the effects of early aspirin therapy in patients with suspected acute ischemic stroke. Both trials showed that starting aspirin within 48 hours of symptom onset produced a modest but consistent net benefit, with about 10 fewer deaths or non-fatal recurrent strokes per 1000 patients during the first few weeks, and approximately 13 fewer patients dead or dependent per 1000 after several weeks or months of follow-up. These benefits were accompanied by only a small, non-statistically significant excess of hemorrhagic stroke or extracranial bleeding in both IST and CAST. The IST used a factorial design to test both aspirin and subcutaneous heparin, there was no interaction between the two treatments, and heparin showed no net clinical benefit. This evidence supports early initiation of aspirin, if patients have no strong contraindications.^23^^,^^24^ The IST enrolled 19 435 patients from 16 countries across 467 hospitals. Patients were randomly allocated to receive either 300 mg of aspirin daily for 14 days or no aspirin in a factorial design. Similarly, CAST enrolled 21 106 patients from 413 hospitals in China, where participants were randomly assigned to receive either 160 mg of aspirin daily for four weeks or placebo. The primary outcomes measured in IST were death within 14 days and death or dependency at 6 months. In contrast, CAST focused on death within four weeks and death or dependency at discharge. Both trials collected baseline patient characteristics at the time of randomization. This comprehensive data collection was intended to facilitate an integrated analysis of both trials.^25^ The individual patient data from IST has been made publicly available by the International Stroke Trial Collaborative Group.^26^ The patient entry and discharge forms from CAST were provided by the University of Oxford upon request. In this study, we focused on the intention-to-treat contrast, including all patients irrespective of their final diagnosis of ischemic stroke. Considering the relatively small fraction of missing data and both large trial sizes, we only used the complete cases for analysis. All the data cleaning and causal ML analyses in this paper were conducted using R statistical software version 4.3.3^27^ (packages listed in Appendix S1).

### Heterogeneous treatment effect modeling

We made use of Rubin’s potential outcome framework.^28^ Consider a binary treatment assignment $T$ (where $T=0$ denotes control and $T=1$ treatment) and a binary outcome $Y$ (where $Y=0$ indicates the absence of the target event, and $Y=1$ indicates the event occurrence, $Y=1$ is harmful in our RCT settings). In this framework, each patient has two potential outcomes: ${Y}^{(0)}$ represents the outcome that would be observed if the patient did not receive the treatment, and ${Y}^{(1)}$represents the outcome that would be observed if the patient did receive the treatment. For any individual $i$, only one of these potential outcomes is observable, depending on the actual treatment assignment ${T}_i$; while the other potential outcome is its counterfactual. Consequently, the individual treatment effect, defined as the difference between the two potential outcomes of that individual: ${Y}_i^{(1)}-{Y}_i^{(0)}$, is fundamentally non-identifiable. While the true individual treatment effect is unobservable, we can instead estimate the average treatment effect (ATE) in the target population defined as the expectation of individual treatment effects: $\mathrm{ATE}=\mathbb{E}\left[{\mathrm{Y}}^{(1)}-{\mathrm{Y}}^{(0)}\right]$, also called the causal risk difference. ATEs can be estimated empirically in a valid RCT sample $S$ under standard identifiability assumptions (consistency, exchangeability, positivity, and no interference^11^). Specifically, the estimator is defined as ${\overline{ATE}}_S=\frac{1}{n_1}{\sum}_{i\in S,\kern0.5em {T}_i=1}{Y}_i-\frac{1}{n_0}{\sum}_{i\in S,\kern0.5em {T}_i=0}{Y}_i$, where ${n}_1$ is the treatment group size and ${n}_0$ the control group size. There are other widely-used effect measures in the literature^29^ such as the odds ratio: ${ATE}^{OR}=\kern0.5em \frac{P\left({Y}^{(1)}=1\right)}{P\left({Y}^{(1)}=0\right)}/\frac{P\left({Y}^{(0)}=1\right)}{P\left({Y}^{(0)}=0\right)}$, or risk ratio: ${ATE}^{RR}=\frac{P\left({Y}^{(1)}=1\right)}{P\left({Y}^{(0)}=1\right)}$, which quantify the same causal effect as an odds/risk ratio.

 To capture treatment effect heterogeneity across patients, we can define the individualized treatment effect (ITE) for a patient $ i $ with baseline covariates $ X={x}_i $ as:


(1) \begin{equation*} {\mathrm{ITE}}_{\mathrm{i}}\left({\mathrm{x}}_{\mathrm{i}}\right)=\mathrm{P}\left({\mathrm{Y}}_{\mathrm{i}}^{(1)}=1|\mathrm{X}={\mathrm{x}}_{\mathrm{i}}\right)-\mathrm{P}\left({\mathrm{Y}}_{\mathrm{i}}^{(0)}=1|\mathrm{X}={\mathrm{x}}_{\mathrm{i}}\right). \end{equation*}

Under similar identifiability assumptions on consistency, exchangeability, positivity, and no interference,^11^ we can rewrite the definition of individualized treatment effect in terms of observable quantities:


(2) \begin{equation*} {\mathrm{ITE}}_{\mathrm{i}}\left({\mathrm{x}}_{\mathrm{i}}\right)=\mathrm{P}\left({\mathrm{Y}}_{\mathrm{i}}=1|\mathrm{T}=1,\mathrm{X}={\mathrm{x}}_{\mathrm{i}}\right)-\mathrm{P}\left({\mathrm{Y}}_{\mathrm{i}}=1|\mathrm{T}=0,\mathrm{X}={\mathrm{x}}_{\mathrm{i}}\right). \end{equation*}

Here, unlike in the ATE, we explicitly aim to estimate counterfactual potential outcomes for individuals, conditioning on their covariates. Therefore, the ITE above is also called conditional average treatment effect (CATE).

By defining treatment effects in terms of observable quantities as in equation (2), causal ML models can be effectively applied to real data. More specifically, we applied causal ML methods on trial data to estimate $\mathrm{P}\left({\mathrm{Y}}_{\mathrm{i}}=1|\mathrm{T}=1,\mathrm{X}={\mathrm{x}}_{\mathrm{i}}\right)$ and $\mathrm{P}\left({\mathrm{Y}}_{\mathrm{i}}=1|\mathrm{T}=0,\mathrm{X}={\mathrm{x}}_{\mathrm{i}}\right)$. Current causal ML methods for learning ITE can be categorized into three groups^10^^,^^17^: metalearners (which combine standard prediction models to derive treatment contrasts), tree-based methods (which infer treatment effect heterogeneity through partitioning), and deep learning methods (which approximate counterfactual outcomes using neural architectures). In this study, we evaluated 17 causal ML methods, spanning all three categories, that have been used in medical research for identifying HTE. Specifically, we examined:

- Metalearners,^16^ including T-learners based on logistic regression,^11^ penalized logistic regression,^30^ random forest,^31^ support vector machine,^32^ and XGBoost^33^; S-learners using logistic regression, penalized logistic regression, XGBoost and BART^34^^,^^35^; X-learners with random forest and BART; DR-learners with random forest.^36^
- Tree-based methods,^37^ including causal forest,^38^^,^^39^ model-based recursive partitioning,^40^^,^^41^ and Bayesian causal forest.^42^
- Deep learning methods, represented by CVAE^43^ and GANITE.^44^

Model details are available in the Appendix S2.

### Evaluation metrics

Traditionally, ITE density plots and outcome prediction accuracy have been used to assess model validity.^11^^,^^13^^,^^15^ ITE density plots compare the distribution of estimated ITEs in the training data with those in the test data. Ideally, a well-calibrated model should yield the same distributions across training and test data, while large discrepancies suggest potential overfitting or lack of transportability. We utilized both visual-based and quantitative metrics to directly assess model reliability and the accuracy of ITE estimates. Visual-based metrics include outcome-ITE plots, subgroup ATE-ITE plots, and benefit-harm density plots, while quantitative validation metrics include c-for-benefit,^18^ model-based concordance probability for benefit (mbcb),^19^ and calibration-based pseudo R-squared.^20^

#### Visual-based metrics

We used outcome-ITE plots (see Figure 2a as an example) to examine the relationship between estimated ITE values and observed outcomes under different treatment conditions. Logistic regression lines with 95% confidence intervals were used to illustrate the predicted probability of outcomes as a function of ITE, stratified by treatment groups. Additionally, we conducted subgroup treatment effects trend analyses and designed benefit-harm effects density plots (see Figure 2b and 2c as an example), where we plotted ATEs across subgroups delineated by the estimated ITEs. The benefit group includes individuals with a negative estimated ITE, while the harm group consists of those with null or positive ITEs. (Here, the estimated ITE is conditioned on the patient’s baseline characteristics, distinguishing it from the contrast between potential outcomes.) Similar ideas using strata defined by clinical cut-offs for predicted treatment benefits have been used in previous research.^11^^,^^15^^,^^20^^,^^45^ Ideally, the trend in ATEs should mirror that of ITEs. Besides, calibration curves and receiver operating characteristic (ROC) curves were used to evaluate the performance of outcome predictions. Further discussions can be found in Appendix S3.

#### Quantitative metrics

To evaluate how well individualized treatment effect models distinguish between patients who benefit more or less from the treatment, we applied the c-for-benefit and mbcb metrics. c-for-benefit is motivated by the conventional c-statistics used in risk prediction, but instead evaluates a model’s ability to discriminate treatment benefit rather than outcome risk.^18^ The core concept is built on matched pairs of patients who share the same predicted ITE but differ in their treatment assignments. For each pair, the observed treatment effect is defined as the treated patient’s outcome minus the control patient’s outcome.^18^ In the binary outcome setting described in Section Heterogeneous treatment effect modeling, this results in three possible observed treatment effect values: +1 referring to treatment harm, 0 referring to no treatment effect, and -1 referring to treatment benefit. The c-for-benefit is then the probability that, when comparing two such patient pairs with distinct observed treatment effects, the pair with the larger observed treatment effect also corresponds to the higher estimated ITE.^18^ The mbcb builds on the same principle but avoids dependence on how pairs are formed, and it replaces the observed outcomes with predicted outcomes.^19^ Specifically, it relies on models that predict both potential outcomes (${Y}^{(0)}\ and\ {Y}^{(1)}$) for each individual. These model-based predictions are then used to define the treatment effect within the pair and evaluate concordance of estimated ITEs.^19^ Therefore, mbcb can only be used for models that provide explicit potential outcome predictions under both treatment and control. It is not applicable to methods that estimate ITEs directly without outcome modeling. There are also other variations of c-for-benefit^46^^,^^47^; however, due to the focus of our study, we do not cover all of them.

There exist several metrics assessing how well a model captures heterogeneous treatment effects compared with a constant model by evaluating outcomes across quantiles of the estimated ITEs.^20^^,^^48^ Here, we used one representative approach, the Calibration-based pseudo R-squared.^20^ Consider partitioning the sample $S$ into $K$ disjoint bins $\left\{{S}_1,{S}_2,\dots, {S}_K\right\}$: $S={\bigcup}_{k=1}^K{S}_k$ by the quantiles of estimated ITEs. Denote the size of the *k*-th bin as ${n}_k$ and the total sample size is ${n}_S$. Then the calibration-based pseudo R-squared is defined as: $1-\frac{calibration\ score}{standardized\ score}$, where the calibration score is ${\sum}_{k=1}^K\frac{n_k}{n_S}\times \big|{\overline{ITE}}_{S_k}-{\overline{ATE}}_{S_k}\big|$, and the standardized score is ${\sum}_{k=1}^K\frac{n_k}{n_S}\times \big|{\overline{ATE}}_S-{\overline{ATE}}_{S_k}\big|$. ${\overline{ITE}}_{S_k}$represents the average estimated ITE in ${S}_k$. ${\overline{ATE}}_S$ and ${\overline{ATE}}_{S_k}$ are the sample estimates of ATE defined in Section Heterogeneous treatment effect modeling.

For all these metrics, a quantitative score closer to 1 indicates a better fit.

### Validation strategy

**Figure 1:**
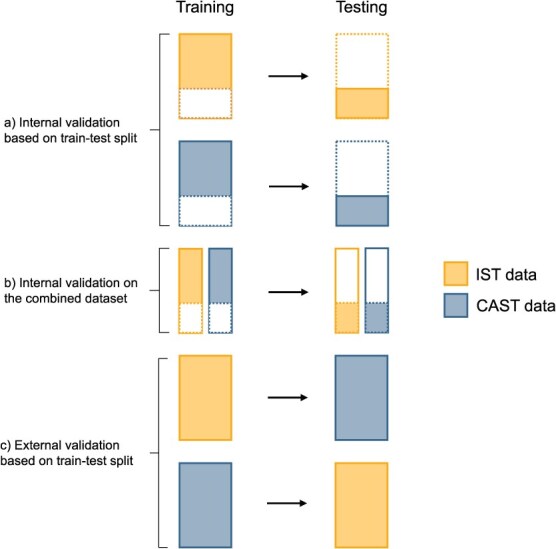
Illustration of internal and external validation strategies using IST and CAST datasets. **a**, internal validation based on the train-test split, where the IST and CAST datasets are each divided into training and test data for model development and evaluation respectively. **b**, internal validation on the combined IST and CAST dataset, where the combined dataset is divided into training and test data. **c**, external validation across datasets, where both the IST and CAST datasets are alternately used as training and test data. Abbreviations: IST: The international stroke trial, CAST: The Chinese acute stroke trial.

Similar to the sample splitting validation approach,^49^ our validation framework used standard internal-external validation parts to test the robustness and generalizability of the models (see Figure 1). Internal validation uses a train-test split within the same dataset, allowing us to evaluate model performance on unseen data from the same source. External validation applies the models to a new dataset, providing an additional layer and evaluating the models’ applicability to distribution shift scenarios.

#### Internal validation

We first conducted internal validation within the IST and CAST dataset separately (Figure 1, Panel a). Patients were randomly allocated to either training or test data using a 2:1 ratio. The training data was utilized for fine-tuning the causal ML models, and we subsequently evaluated the trained models on the test data. Besides random allocation, we also performed a geographical split (Europe for training; non-Europe for testing) within the IST dataset. Furthermore, we merged the data from the two trials into a unified dataset, which was then split into training and test data for analysis, akin to the previous approach (see Figure 1, Panel b). To eliminate the impact of a single random split, we further validated the model by aggregating results across 100 or 1000 random training-test splits.

#### External validation

We conducted external validations using both the IST and CAST datasets, leveraging similarities and differences in their populations. More specifically, we trained the models on the IST dataset and validated them on the CAST dataset, followed by the reverse process to ensure a comprehensive evaluation (see Figure 1, Panel c).

### Outcomes and covariates

For internal validation within the two trials, we considered dependency or death at 6 months for the IST dataset and dependency or death at 4 weeks for the CAST dataset as the primary outcome variables. To train the causal ML models, we used 16 baseline covariates common to both datasets: age, sex, delay in hours from onset to randomization, conscious state, CT scan results, presence of atrial fibrillation, systolic blood pressure, and neurological deficits (including 8 types of deficits). For the IST dataset, we included three additional variables: geographical information, aspirin history within three days prior to randomization, and stroke type. Internal validation on the combined dataset and external validation required aligning the outcome and covariates between the two trial datasets. Poststroke period is typically categorized into hyperacute (0-24 hours), acute (1-7 days), subacute, and chronic stages.^50^ Despite the lack of consensus in the literature regarding the distinction between subacute and chronic periods, durations of 14 days and 4 weeks are commonly grouped together. Therefore, we selected death within 14 days as the outcome for IST and death within 4 weeks as the outcome for CAST. For baseline covariates, we utilized the interaction set comprising the 16 variables identified in the internal validation of the IST and CAST datasets.

### Simulation study

To further investigate the strengths and limitations of causal ML models in estimating heterogeneous treatment effects, we designed simulations that mimic the setting of the stroke RCTs (IST and CAST) while allowing full control over the data generation mechanisms. More details of the simulation design are provided in the Appendix S4, and all code is publicly available in our open GitLab repository.

## Results

The IST dataset comprised 19 435 patients with 9720 treated with aspirin and 9715 untreated. 5.97% of individuals had incomplete data for the 6-month death or dependency outcome, and 5.19% of individuals had incomplete data for the 14-day death outcome. Following data cleaning and resolution of dataset inconsistencies, the CAST trial included 20 614 patients, with 10 313 treated with aspirin and 10 301 untreated. Incomplete data rates were 3.57% for the death or dependency outcome and 3.29% for death. Given the RCT design, both treated and control groups exhibited balanced distributions across training and test data.

### ITE estimation

The causal ML estimation in both the IST and CAST datasets revealed heterogeneity among patients (see Figure S1–S3.10.2). The effect of aspirin treatment varied among patients, with some benefitting from the treatment (ie, ITE < 0) and others not benefiting or even being harmed (ie, ITE ≥ 0). For most models, we observed that this heterogeneity of ITE was consistent across training and internal test datasets, as well as between treatment and control groups. This is also supported by discrimination and calibration plots of predicted and observed outcomes (see Figure S2.6.1–S2.9.2, Figure S3.6.1–S3.9.2).

### Internal validation

**Figure 2:**
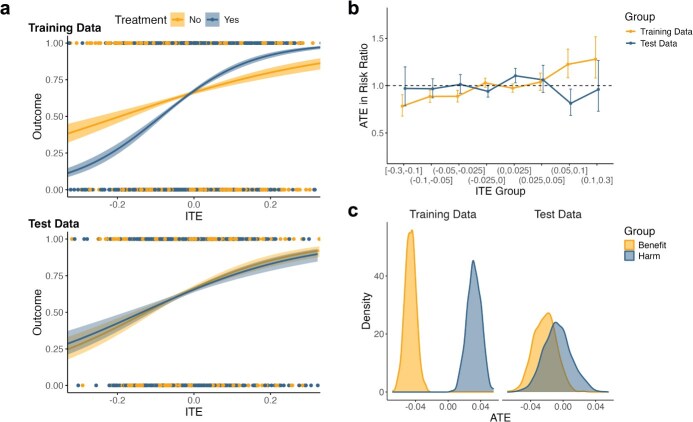
Comparative analysis of T-learner logistic regression-based individualized treatment effects in IST. **a**, comparing patient outcomes against estimated ITE values between training data and test data. Orange dots and lines represent the true and fitted outcome probabilities for the control group, while blue dots and lines indicate the true and fitted outcome probabilities for the treatment group. **b**, ATE in risk ratio within different ITE subgroups, confidence intervals at 95% level. Orange represents the training data, and blue indicates the test data. The horizontal dashed line at 1.0 means no treatment effects. **c**, density distributions of ATE in training data and test data stratified by benefit (orange) and harm (blue) groups. The distribution of ATE comes from 1000 random train-test split experiments. Benefit means negative ITE values and harm means positive ITE values. The outcome variable used in **a-c** is death or dependency at 6 months. Abbreviations: IST: The international stroke trial, ITE: Individualized treatment effect, ATE: Average treatment effect.

Internal validation uncovered significant disparities in model performance. Figure 2 presents the results of the T-learner logistic regression model for the 6-month death or dependency outcome in the IST dataset. Figure 2a indicates that, in the training data, individuals predicted to experience harmful treatment effects have a higher risk of death in the aspirin group compared to the control group. In contrast, for those predicted to benefit from the treatment, the risk is lower in the aspirin group than in the control group. However, this pattern does not hold in the internal test data. This is also evident from the subgroup ITE analysis as shown in Figure 2b. ATE in risk ratio increases at higher ITEs in the training but not in the test data. Repeated experiments shown in Figure 2c confirm these discrepancies across multiple splits when stratified by benefit and harm groups. Penalized logistic regression lowered the discriminatory accuracy on the training data between treatment and control groups but did not lead to consistent results on the test data. Alternatively, using more complex models such as random forest, XGBoost, and causal forest drew a more pronounced separation between the treatment and control group predictions in the training data, but also failed to replicate in the test data. Table 1 summarizes the quantitative validation metrics for the training and test data across the 17 causal ML models evaluated on the IST dataset. None of the models consistently achieved similar metrics close to 1 across training and test datasets. Additionally, geographical validation within the IST dataset, internal validation using the CAST dataset, and sensitivity analyses highlighted similar discrepancies in model performance. Detailed visual and quantitative validation results are provided in Figures S1–S3.10.2 and Table S1.

**Table 1:** Summary of quantitative validation metrics on IST training and test dataset of 17 causal machine learning methods with 6 months’ death or dependency outcome.

		**c-for-benefit (training)**	**c-for-benefit (test)**	**mbcb (training)**	**mbcb (test)**	**Calibration-based pseudo R-squared (training)**	**Calibration-based pseudo R-squared (test)**
**T-learner**	Logistic Regression	0.541	0.502	0.553	0.551	0.685	−1.701
	Random Forest	0.972	0.491	0.718	0.616	0.245	−5.855
	Support Vector Machine	0.537	0.503	0.552	0.552	0.574	−2.761
	XGBoost	0.657	0.491	0.615	0.613	0.590	−3.348
	Penalized Logistic Regression	0.540	0.500	0.551	0.550	0.516	−2.564
**S-learner**	Logistic Regression	0.507	0.496	0.512	0.511	0.144	−0.641
	Penalized Logistic Regression	0.531	0.497	0.520	0.520	0.508	−1.697
	XGBoost	0.682	0.493	0.571	0.566	0.315	−3.066
	BART	0.533	0.500	0.531	0.531	0.419	−1.980
**X-learner**	Random Forest	0.890	0.496	0.673	0.583	0.204	−4.563
	BART	0.551	0.522	0.547	0.546	0.430	0.426
**DR-learner**	Random Forest	0.849	0.496	0.501	0.498	0.538	−5.362
**Tree-based methods**	Causal Forest	0.593	0.507	-	-	0.083	0.053
	Bayesian Causal Forest	0.524	0.524	-	-	0.062	−0.014
	Model-based Recursive Partitioning	0.794	0.502	-	-	-	-
**Deep Learning**	CVAE	0.522	0.491	0.531	0.532	0.622	−0.879
	GANITE	0.519	0.512	0.539	0.540	−0.647	−1.870

*Abbreviation*: IST: the International Stroke Trial.

Notes: Consistent values across training and test data that are closer to 1 indicate a better model fit. The dash symbol indicates instances where the validation metric could not be assessed due to the model’s mechanism (mbcb can be only used for models that provide explicit potential outcomes predictions under both treatment and control).

In the internal validation on the merged dataset, we observed generally lower risks in the outcomes (see Figure S4.1.1–S4.10.2). Besides, the estimated individualized treatment effects had narrower scales compared to long-term outcomes and the inclusion of dependency data. Similarly, discrepancies in model behavior between training and test groups persisted across all causal ML models (see also Table S2).

### External validation

Cross-dataset external validation reiterated these findings (see Figure S5.1.1–S6.9.2, Table S3, S4). Regardless of which trial dataset served as the training or test set, we consistently observed the same disparity in model behavior between the training and test groups across all the causal ML models we applied.

### Simulation study

Simulation results (see Figure S7.1.1–S7.4.2) indicated that, under the correct model specification, logistic regression methods—including T-learner logistic regression, T-learner penalized logistic regression, and S-learner penalized logistic regression—as well as T-learner support vector machine, accurately modeled ITEs. In contrast, the causal forest and T-learner random forest performed poorly. All of our evaluation plots revealed discrepancies between training and test data for poorly performing methods but not for well-performing ones. These findings were consistent across both low- and high-dimensional settings and regardless of whether the models included a mix of discrete and continuous variables or only continuous variables. However, when models were constructed using variables with negligible treatment-covariate interactions, causal ML models failed to accurately capture ITEs.

## Discussion

Our study found that identification and estimation in commonly used ML methods for heterogeneous treatment effects fail to replicate even at internal validation stages in our non-simulated scenarios. We arrive at this result after fitting 17 ML methods applied to two large RCTs and evaluating them using three visual-based metrics and three quantitative metrics of treatment effect heterogeneity.

Causal ML methods’ utility resides in their ability to predict counterfactual treatment outcomes and estimate treatment effects at the individual level. Currently, ITE density plots are often used to assess the model’s ability to capture heterogeneity in treatment effects across patients.^11^^,^^13^^,^^15^ Outcome predictive discriminatory accuracy has also been used to justify ITE estimations.^10^^,^^13^ In our application, all tested causal ML methods produced ITE estimates with substantial variability (see Figure S2.1.1–S2.3.2, S2.6.1–S2.9.2, Figure S3.1.1–S3.3.2, S3.6.1–S3.9.2). And our experiments on IST and CAST datasets showed that even simple methods, such as T-learner logistic regression, precisely predicted outcomes. These findings were consistent with previous research results.^13^^,^^15^

However, the primary goal should be to validate ITE estimates rather than predict outcomes.^51^ And variability in estimated ITEs does not, by itself, establish the presence of true underlying treatment effect heterogeneity. Therefore, evaluation metrics, including visual outcome-ITE plots, subgroup ATE-ITE plots, benefit-harm density plots, and quantitative measures such as c-for-benefit, mbcb, and calibration-based pseudo R-squared, are crucial for assessing estimates of individualized and subgroup treatment effects. We assessed ITEs on both absolute and relative scales.^17^^,^^52^ There are other evaluation metrics proposed in the literature, such as the precision in estimation of heterogeneous effect (PEHE).^34^ It is used to evaluate the accuracy of ITE estimates by calculating the mean squared error between the predicted and true ITE. However, in real-world data, true ITE values are not directly observable, making PEHE impractical for direct assessment in our study. Some recent work has explored the use of influence functions to efficiently estimate the loss function of causal inference models without requiring access to true causal effects.^53^ While these methods offer promising alternatives, we did not include them in our study due to computational complexity and left them for future research.

Internal validation assesses ITEs within the same population. A significant challenge in real-world RCTs is the unknown underlying data-generating mechanism, making the true individualized treatment effect unrecoverable. While ITEs cannot be directly validated, it is expected of causal ML models to perform consistently across training and test data under random splits. Our internal validation results indicated that all tested causal ML models exhibit significant discrepancies between training and test data. These findings suggest that current causal ML models might struggle to reliably estimate ITEs even within the same populations they were trained on, highlighting issues with the assumptions required for the models to yield unbiased ITEs.

External validation is crucial because an ideal causal ML model should remain robust and resist distribution shifts. Patients enrolled in the IST and CAST trials exhibited distinct demographic characteristics, encompassing variations in geographical backgrounds, age distributions, and stroke severity profiles. Additionally, clinical subtypes of stroke prevalence differed between Asian and Western populations. Our external validation experiments showed that none of the models generalize well to the test data, regardless of which trial dataset is used for training or testing.

Validating ITE estimates is crucial when true treatment effect heterogeneity exists. In cases where treatment effect heterogeneity is minimal or absent in the target population, observed variability in ITE point estimates from ML models can not inherently confirm the existence of heterogeneity. The challenge lies in the fact that treatment effect heterogeneity is often identified through exploratory post-hoc analyses, which lack the statistical power of prospectively specified subgroup analyses in clinical trials.^54^ When it comes to the secondary prevention of aspirin, which is the case of our trial data, research has shown that aspirin may increase the risk of intracranial hemorrhage and is less effective in inhibiting platelet aggregation in postmenopausal women compared to men with a history of stroke or transient ischemic attack.^55^^,^^56^ This provides suggestive evidence that treatment effect heterogeneity exists in the general population. Our focus is not on any specific clinical context but on the broader methodological point: when there is no genuine heterogeneity, causal ML models may still detect spurious subgroup effects, and when true heterogeneity exists, different methods may yield inconsistent estimates even internally. In both cases, rigorous validation is essential.

There are still several limitations in our study. First, the baseline patient characteristics used to train the models were limited by the stroke trials’ designs. Including currently unavailable information that moderates the treatment effects could provide a more comprehensive patient profile in which the set of ML models tested could perform better. Second, the scope of our models and validation metrics cannot cover the vast set of potentially available ones. Methods designed for censored survival outcomes, such as Causal Survival Forests,^57^ and models that consider time-varying effects are also worth investigating. However, we believe to have included the most widely used causal ML methods in medical research, as shown in a recent scoping review on ML methods for ITE in randomized controlled trials.^17^^,^^23^^,^^24^ Moreover, our analysis focused on point estimates of ITEs. Quantifying the uncertainty of these estimators lies beyond the scope of this paper, but remains an important direction for future research. When lacking robust uncertainty quantification, rigorous validation is essential to distinguish true clinical signal from spurious modeling artifacts. Finally, our estimates have focused on the intention-to-treat causal contrast, while other contrasts might be of more clinical interest. We chose the contrast with the highest degree of internal validity.

Overall, our research exemplifies that blindly applying current heterogeneous causal ML methods to individual patients does not lead to reliable results and may result in undesired outcomes. Even in the absence of true effect heterogeneity, withholding treatment from patients predicted to experience net harm can induce avoidable undertreatment and yield worse outcomes than treating everyone. While systematic validation is essential for treatment effect heterogeneity estimation in randomized controlled trials, it is even more critical for observational studies, where confounding, data quality, and identifiability problems further complicate the analysis. Future work is necessary to advance the credibility and evaluation guidelines for counterfactual models that are suitable for broad studies. Considering the findings of this study, the gap between standard ATE treatment approaches and individualized treatment decisions driven by machine learning methods remains large at present.

## Ethics declarations

Because all patient data used in this study were fully anonymized prior to access and are available from public sources, no ethical approval was required.

## Supplementary Material

Web_Material_kwag065

## Data Availability

The code used for the analyses conducted in this study is available at GitLab (https://gitlab.uzh.ch/hongruyu.chen/causal_ml-for-ite). The individual patient data from IST has been made publicly available by the International Stroke Trial Collaborative Group (https://trialsjournal.biomedcentral.com/articles/10.1186/1745-6215-12-101#Sec8). And the patient entry and discharge forms from CAST were provided by the University of Oxford upon request.

## References

[1] Kent DM, Steyerberg E, van Klaveren D. Personalized evidence based medicine: predictive approaches to heterogeneous treatment effects. *BMJ.* 2018;363:k4245. 10.1136/bmj.k4245 30530757 PMC6889830

[2] Schork NJ . Personalized medicine: time for one-person trials. *Nature.* 2015;520(7549):609-611. 10.1038/520609a 25925459

[3] Kosorok MR, Laber EB. Precision medicine. *Ann Rev Stat Appl*. 2019;6(1):263-286. 10.1146/annurev-statistics-030718-105251 31073534 PMC6502478

[4] Kent DM . Overall average treatment effects from clinical trials, one-variable-at-a-time subgroup analyses and predictive approaches to heterogeneous treatment effects: toward a more patient-centered evidence-based medicine. *Clin Trials*. 2023;20(4):328-337. 10.1177/17407745231171897 37148125

[5] Deaton A, Cartwright N. Understanding and misunderstanding randomized controlled trials. *Soc Sci Med*. 2018;210:2-21. 10.1016/j.socscimed.2017.12.005 29331519 PMC6019115

[6] Subramanian SV, Kim R, Christakis NA. The “average” treatment effect: a construct ripe for retirement. A commentary on Deaton and Cartwright. *Soc Sci Med*. 2018;210:77-82. 10.1016/j.socscimed.2018.04.027 29724462

[7] Bica I, Alaa AM, Lambert C, et al. From real-world patient data to individualized treatment effects using machine learning: current and future methods to address underlying challenges. *Clin Pharmacol Ther*. 2021;109(1):87-100. 10.1002/cpt.1907 32449163

[8] Kaddour J, Lynch A, Liu Q, Kusner MJ, Silva R. Causal machine learning: a survey and open problems. Found Trends Optim. 2025;9(1-2):1-247. 10.1561/2400000052

[9] Curth A, Peck RW, McKinney E, et al. Using machine learning to individualize treatment effect estimation: challenges and opportunities. *Clin Pharmacol Ther*. 2024;115(4):710-719. 10.1002/cpt.3159 38124482

[10] Feuerriegel S, Frauen D, Melnychuk V, et al. Causal machine learning for predicting treatment outcomes. *Nat Med*. 2024;30(4):958-968. 10.1038/s41591-024-02902-1 38641741

[11] Hoogland J, IntHout J, Belias M, et al. A tutorial on individualized treatment effect prediction from randomized trials with a binary endpoint. *Stat Med*. 2021;40(26):5961-5981. 10.1002/sim.9154 34402094 PMC9291969

[12] Nie X, Wager S. Quasi-oracle estimation of heterogeneous treatment effects. *Biometrika.* 2021;108(2):299-319. 10.1093/biomet/asaa076

[13] Nguyen TL, Collins GS, Landais P, et al. Counterfactual clinical prediction models could help to infer individualized treatment effects in randomized controlled trials—an illustration with the international stroke trial. *J Clin Epidemiol*. 2020;125:47-56. 10.1016/j.jclinepi.2020.05.022 32464321

[14] Msaouel P, Lee J, Karam JA, et al. A causal framework for making individualized treatment decisions in oncology. *Cancers (Basel)*. 2022;14(16):3923. 10.3390/cancers14163923 36010916 PMC9406391

[15] Venkatasubramaniam A, Mateen BA, Shields BM, et al. Comparison of causal forest and regression-based approaches to evaluate treatment effect heterogeneity: an application for type 2 diabetes precision medicine. *BMC Med Inform Decis Mak*. 2023;23(1):110. 10.1186/s12911-023-02207-2 37328784 PMC10276367

[16] Künzel SR, Sekhon JS, Bickel PJ, et al. Metalearners for estimating heterogeneous treatment effects using machine learning. *Proc Natl Acad Sci*. 2019;116(10):4156-4165. 10.1073/pnas.1804597116 30770453 PMC6410831

[17] Inoue K, Adomi M, Efthimiou O, et al. Machine learning approaches to evaluate heterogeneous treatment effects in randomized controlled trials: a scoping review. *J Clin Epidemiol*. 2024;176:111538. 10.1016/j.jclinepi.2024.111538 39305940

[18] van Klaveren D, Steyerberg EW, Serruys PW, et al. The proposed ‘concordance-statistic for benefit’ provided a useful metric when modeling heterogeneous treatment effects. *J Clin Epidemiol*. 2018;94:59-68. 10.1016/j.jclinepi.2017.10.021 29132832 PMC7448760

[19] Hoogland J, Efthimiou O, Nguyen TL, et al. Evaluating individualized treatment effect predictions: a model-based perspective on discrimination and calibration assessment. *Stat Med*. 2024;43(23):4481-4498. 10.1002/sim.10186 39090523

[20] Dwivedi R, Tan YS, Park B, et al. Stable discovery of interpretable subgroups via calibration in causal studies. *Int Stat Rev*. 2020;88(S1):S135-S178. 10.1111/insr.12427

[21] Lu M, Sadiq S, Feaster DJ, et al. Estimating individual treatment effect in observational data using random Forest methods. *J Comput Graph Stat*. 2018;27(1):209-219. 10.1080/10618600.2017.1356325 29706752 PMC5920646

[22] Booth CM, Tannock IF. Randomised controlled trials and population-based observational research: partners in the evolution of medical evidence. *Br J Cancer*. 2014;110(3):551-555. 10.1038/bjc.2013.725 24495873 PMC3915111

[23] The international stroke trial (IST): a randomised trial of aspirin, subcutaneous heparin, both, or neither among 19 435 patients with acute ischaemic stroke. *Lancet.* 1997;349(9065):1569-1581. 10.1016/S0140-6736(97)04011-7 9174558

[24] Chen ZM . CAST: randomised placebo-controlled trial of early aspirin use in 20 000 patients with acute ischaemic stroke. *Lancet*. 1997;349(9066):1641-1649. 10.1016/S0140-6736(97)04010-5 9186381

[25] Chen Z, Sandercock P, Pan H, et al. Indications for early aspirin use in acute ischemic stroke: a combined analysis of 40 000 randomized patients from the Chinese acute stroke trial and the international stroke trial. *Stroke.* 2000;31(6):1240-1249. 10.1161/01.STR.31.6.1240 10835439

[26] the International Stroke Trial Collaborative Group, Sandercock PA, Niewada M, et al. The international stroke trial database. *Trials*. 2011;12(1):101. 10.1186/1745-6215-12-101 21510853 PMC3104487

[27] R Core Team . R: A Language and Environment for Statistical Computing. 2024. Accessed July 29, 2024. https://www.r-project.org/

[28] Rubin DB . Causal inference using potential outcomes: design, modeling, decisions. *J Am Stat Assoc*. 2005;100(469):322-331. 10.1198/016214504000001880

[29] Colnet B, Josse J, Varoquaux G, et al. Risk ratio, odds ratio, risk difference... Which causal measure is easier to generalize? arXiv. 2025. 10.48550/arXiv.2303.16008

[30] Zou H, Hastie T. Regularization and variable selection via the elastic net. *J R Stat Soc Series B Stat Methodology*. 2005;67(2):301-320. 10.1111/j.1467-9868.2005.00503.x

[31] TK Ho . Random decision forests. In: *Proceedings of 3rd International Conference on Document Analysis and Recognition*. *IEEE*. Vol 1. 1995:278-282.

[32] Cortes C, Vapnik V. Support-vector networks. *Mach Learn*. 1995;20(3):273-297. 10.1007/BF00994018

[33] T Chen, C Guestrin. XGBoost: A Scalable Tree Boosting System. In: *Proceedings of the 22nd ACM SIGKDD International Conference on Knowledge Discovery and Data Mining*. KDD ‘16. *Association for Computing Machinery*; 2016:785-794.

[34] Hill JL . Bayesian nonparametric modeling for causal inference. *J Comput Graph Stat*. 2011;20(1):217-240. 10.1198/jcgs.2010.08162

[35] Sparapani R, Spanbauer C, McCulloch R. Nonparametric machine learning and efficient computation with Bayesian additive regression trees: the BART R package. *J Stat Softw*. 2021;97(1):1-66. 10.18637/jss.v097.i01

[36] Kennedy EH . Towards optimal doubly robust estimation of heterogeneous causal effects. *Electron J Stat*. 2023;17(2):3008-3049. 10.1214/23-EJS2157

[37] Athey S, Imbens G. Recursive partitioning for heterogeneous causal effects. *Proc Natl Acad Sci U S A*. 2016;113(27):7353-7360. 10.1073/pnas.1510489113 27382149 PMC4941430

[38] Wager S, Athey S. Estimation and inference of heterogeneous treatment effects using random forests. *J Am Stat Assoc*. 2018;113(523):1228-1242. 10.1080/01621459.2017.1319839

[39] Jawadekar N, Kezios K, Odden MC, et al. Practical guide to honest causal forests for identifying heterogeneous treatment effects. *Am J Epidemiol*. 2023;192(7):1155-1165. 10.1093/aje/kwad043 36843042

[40] Seibold H, Zeileis A, Hothorn T. Model-based recursive partitioning for subgroup analyses. *Int J Biostat*. 2016;12(1):45-63. 10.1515/ijb-2015-0032 27227717

[41] Seibold H, Zeileis A, Hothorn T. model4you: An R package for personalised treatment effect estimation. J Open Res Softw 2019;7(1):17. doi:10.5334/jors.219

[42] Hahn PR, Murray JS, Carvalho CM. Bayesian regression tree models for causal inference: regularization, confounding, and heterogeneous effects (with discussion). *Bayesian Anal*. 2020;15(3):965-1056. 10.1214/19-BA1195

[43] K Sohn, H Lee, X Yan. Learning Structured Output Representation using Deep Conditional Generative Models. In: *Advances in Neural Information Processing Systems*. Vol 28. Curran Associates, Inc.; 2015. Accessed December 9, 2024. https://papers.nips.cc/paper_files/paper/2015/hash/8d55a249e6baa5c06772297520da2051-Abstract.html

[44] J Yoon, J Jordon, van der M Schaar . GANITE: estimation of individualized treatment effects using generative adversarial nets. 2018. Accessed December 9, 2024. https://openreview.net/forum?id=ByKWUeWA-

[45] Imai K, Li ML. Statistical inference for heterogeneous treatment effects discovered by generic machine learning in randomized experiments. *Journal of Business & Economic Statistics*. 2025;43(1):256-268. 10.1080/07350015.2024.2358909

[46] Maas CCHM, Kent DM, Hughes MC, et al. Performance metrics for models designed to predict treatment effect. *BMC Med Res Methodol*. 2023;23(1):165. 10.1186/s12874-023-01974-w 37422647 PMC10329397

[47] Efthimiou O, Hoogland J, Debray TPA, et al. Measuring the performance of prediction models to personalize treatment choice. *Stat Med*. 2023;42(8):1188-1206. 10.1002/sim.9665 36700492 PMC7615726

[48] Yadlowsky S, Fleming S, Shah N, et al. Evaluating treatment prioritization rules via rank-weighted average treatment effects. *J Am Stat Assoc*. 2025;120(549):38-51. 10.1080/01621459.2024.2393466 40248684 PMC12002561

[49] Chernozhukov V, Chetverikov D, Demirer M, et al. Double/Debiased/Neyman machine learning of treatment effects. *Am Econ Rev*. 2017;107(5):261-265. 10.1257/aer.p20171038

[50] Bernhardt J, Hayward KS, Kwakkel G, et al. Agreed definitions and a shared vision for new standards in stroke recovery research: the stroke recovery and rehabilitation roundtable taskforce. *Int J Stroke*. 2017;12(5):444-450. 10.1177/1747493017711816 28697708

[51] Prosperi M, Guo Y, Sperrin M, et al. Causal inference and counterfactual prediction in machine learning for actionable healthcare. *Nat Mach Intell*. 2020;2(7):369-375. 10.1038/s42256-020-0197-y

[52] Kent DM, Paulus JK, van Klaveren D, et al. The predictive approaches to treatment effect heterogeneity (PATH) statement. *Ann Intern Med*. 2020;172(1):35-45. 10.7326/M18-3667 31711134 PMC7531587

[53] A Alaa, Schaar MVD. Validating Causal Inference Models via Influence Functions. In: Proceedings of the 36th International Conference on Machine Learning. PMLR; 2019;97:191-201. Accessed December 1, 2024. https://proceedings.mlr.press/v97/alaa19a.html

[54] Lipkovich I, Dmitrienko A, D’Agostino RD Sr. Tutorial in biostatistics: data-driven subgroup identification and analysis in clinical trials. *Stat Med*. 2017;36(1):136-196. 10.1002/sim.7064 27488683

[55] Cavallari LH, Helgason CM, Brace LD, et al. Sex difference in the antiplatelet effect of aspirin in patients with stroke. *Ann Pharmacother*. 2006;40(5):812-817. 10.1345/aph.1G569 16608908

[56] He J, Whelton PK, Vu B, et al. Aspirin and risk of hemorrhagic stroke a meta-analysis of randomized controlled trials. *JAMA.* 1998;280(22):1930-1935. 10.1001/jama.280.22.1930 9851479

[57] Cui Y, Kosorok MR, Sverdrup E, et al. Estimating heterogeneous treatment effects with right-censored data via causal survival forests. *J R Stat Soc Ser B Stat Methodol*. 2023;85(2):179-211. 10.1093/jrsssb/qkac001

