# Supplementary material for: Machine learning methods for estimating personalized treatment effects—insights on validity from two large trials
Source: Am J Epidemiol. 2026 Mar 20;195(8):2263–72. doi: 10.1093/aje/kwag065 (PMC13437088; doi:10.1093/aje/kwag065)
Supplement: Supplementary file 1 [file web_material_kwag065.zip › supp_materials_Final.pdf]

Appendix: Machine Learning Methods for Estimating Personalized Treatment Effects - Insights on validity from two large trials

Hongruyu Chen[1], Helena Aebersold[1], Milo Alan Puhan[1,2], Miquel Serra-Burriel[1]

[1] Epidemiology, Biostatistics and Prevention Institute, University of Zurich, Zurich, Switzerland

[2] Department of Epidemiology, Bloomberg School of Public Health, Johns Hopkins University, Baltimore, Maryland, USA

Appendix

S1. Software and Packages……………………………………………………………………………2
S2. Causal Machine Learning Methods…………………………………………………………….....5
S3. ITE Evaluation Metrics……………………………………………………………………...6
S4. Simulation study……………………………………………………………………………......7
Appendix Tables: Table S1 – Table S4 ….……………..…………...……..……………………………..10
Appendix Figures: Figure S1 – Figure S7.4.2. …………………………..……………………………14

S1. Software and Packages

Main analyses were performed in R (version 4.3.3). Below we provide a reference list of the main software packages used. All citations are given in the format provided by the respective R package.

1. R Core Team. (2024). *R: A Language and Environment for Statistical Computing.* R Foundation for Statistical Computing, Vienna, Austria. Retrieved from https://www.R-project.org/
2. Bates, D., Maechler, M., & Jagan, M. (2024). *Matrix: Sparse and Dense Matrix Classes and Methods.* R package version 1.6-5. Retrieved from https://CRAN.R-project.org/package=Matrix
3. Chen, T., He, T., Benesty, M., Khotilovich, V., Tang, Y., Cho, H., Chen, K., Mitchell, R., Cano, I., Zhou, T., Li, M., Xie, J., Lin, M., Geng, Y., Li, Y., & Yuan, J. (2024). *xgboost: Extreme Gradient Boosting.* R package version 1.7.8.1. Retrieved from https://github.com/dmlc/xgboost
4. Comtois, D. (2022). *summarytools: Tools to Quickly and Neatly Summarize Data.* R package version 1.0.1. Retrieved from https://github.com/dcomtois/summarytools
5. Csárdi, G., & FitzJohn, R. (2023). *progress: Terminal Progress Bars.* R package version 1.2.3. Retrieved from https://CRAN.R-project.org/package=progress
6. Friedman, J., Tibshirani, R., & Hastie, T. (2010). Regularization paths for generalized linear models via coordinate descent. *Journal of Statistical Software, 33*(1), 1–22. https://doi.org/10.18637/jss.v033.i01
7. Simon, N., Friedman, J., Tibshirani, R., & Hastie, T. (2011). Regularization paths for Cox's proportional hazards model via coordinate descent. *Journal of Statistical Software, 39*(5), 1–13. https://doi.org/10.18637/jss.v039.i05
8. Tay, J. K., Narasimhan, B., & Hastie, T. (2023). Elastic net regularization paths for all generalized linear models. *Journal of Statistical Software, 106*(1), 1–31. https://doi.org/10.18637/jss.v106.i01
9. Hahn, P., Murray, J., & Carvalho, C. (2020). Bayesian regression tree models for causal inference: Regularization, confounding, and heterogeneous effects. *Bayesian Analysis, 15*(3), 965–1056. https://doi.org/10.1214/19-BA1195
10. Harrell, F. E. Jr. (2024). *Hmisc: Harrell Miscellaneous.* R package version 5.2-1. Retrieved from https://hbiostat.org/R/Hmisc/
11. Harrell, F. E. Jr. (2024). *rms: Regression Modeling Strategies.* R package version 6.8-0. Retrieved from https://hbiostat.org/R/rms/
12. Hoogland, J. (2024). *iteval: Individualized Treatment Effects Validation.* R package version 0.1.1. Retrieved from https://github.com/jeroenhoogland/iteval
13. Hothorn, T., & Winell, H. (2023). *libcoin: Linear Test Statistics for Permutation Inference.* R package version 1.0-10
14. Hothorn, T., Zeileis, A., Hornik, K. (2006). Unbiased recursive partitioning: A conditional inference framework. *Journal of Computational and Graphical Statistics, 15*(3), 651–674. https://doi.org/10.1198/106186006X133933
15. Hothorn, T., Zeileis, A. (2015). partykit: A modular toolkit for recursive partitioning in R. *Journal of Machine Learning Research, 16,* 3905–3909. Retrieved from https://jmlr.org/papers/v16/hothorn15a.html
16. Zeileis, A., Hothorn, T., & Hornik, K. (2008). Model-based recursive partitioning. *Journal of Computational and Graphical Statistics, 17*(2), 492–514. https://doi.org/10.1198/106186008X319331
17. Kassambara, A. (2023). *ggpubr: 'ggplot2' Based Publication Ready Plots.* R package version 0.6.0. Retrieved from https://rpkgs.datanovia.com/ggpubr/
18. Liaw, A., & Wiener, M. (2002). Classification and regression by randomForest. *R News, 2*(3), 18–22. Retrieved from https://CRAN.R-project.org/doc/Rnews/
19. Lim, M., & Hastie, T. (2021). *glinternet: Learning Interactions via Hierarchical Group-Lasso Regularization.* R package version 1.0.12. Retrieved from http://web.stanford.edu/~hastie/Papers/glinternet_jcgs.pdf

20. Makowski, D., Lüdecke, D., Patil, I., Thériault, R., Ben-Shachar, M., & Wiernik, B. (2023). Automated results reporting as a practical tool to improve reproducibility and methodological best practices adoption. *CRAN.* Retrieved from https://easystats.github.io/report/
21. Meyer, D., Dimitriadou, E., Hornik, K., Weingessel, A., & Leisch, F. (2023). *e1071: Misc Functions of the Department of Statistics, Probability Theory Group (Formerly: E1071), TU Wien.* R package version 1.7-14
22. Müller, K. (2020). *here: A Simpler Way to Find Your Files.* R package version 1.0.1. Retrieved from https://CRAN.R-project.org/package=here
23. Müller, K., & Wickham, H. (2023). *tibble: Simple Data Frames.* R package version 3.2.1. Retrieved from https://CRAN.R-project.org/package=tibble
24. Pinheiro, J., Bates, D., & R Core Team. (2023). *nlme: Linear and Nonlinear Mixed Effects Models.* R package version 3.1-164. Retrieved from https://CRAN.R-project.org/package=nlme
Pinheiro, J. C., & Bates, D. M. (2000). *Mixed-Effects Models in S and S-PLUS.* Springer, New York. https://doi.org/10.1007/b98882
25. Robinson, D., Hayes, A., & Couch, S. (2023). *broom: Convert Statistical Objects into Tidy Tibbles.* R package version 1.0.5. Retrieved from https://CRAN.R-project.org/package=broom
26. Seibold, H., Zeileis, A., & Hothorn, T. (2021). *model4you: Stratified and Personalised Models Based on Model-Based Trees and Forests.* R package version 0.9-7
27. Sparapani, R., Spanbauer, C., & McCulloch, R. (2021). Nonparametric machine learning and efficient computation with Bayesian additive regression trees: The BART R package. *Journal of Statistical Software, 97*(1), 1–66. https://doi.org/10.18637/jss.v097.i01
28. Therneau, T. (2024). *A Package for Survival Analysis in R.* R package version 3.5-8. Retrieved from https://CRAN.R-project.org/package=survival
29. Therneau, T. M., & Grambsch, P. M. (2000). *Modeling Survival Data: Extending the Cox Model.* Springer, New York. ISBN 0-387-98784-3
30. Tibshirani, J., Athey, S., Sverdrup, E., & Wager, S. (2024). *grf: Generalized Random Forests.* R package version 2.3.2. Retrieved from https://github.com/grf-labs/grf
31. Van Calster, B., Nieboer, D., Vergouwe, Y., De Cock, B., Pencina, M., & Steyerberg, E. (2016). A calibration hierarchy for risk models was defined: From utopia to empirical data. *Journal of Clinical Epidemiology, 74,* 167–176. https://doi.org/10.1016/j.jclinepi.2015.12.005
32. De Cock, B., Nieboer, D., Van Calster, B., Steyerberg, E., & Vergouwe, Y. (2023). *CalibrationCurves: Assessing the agreement between observed outcomes and predictions.* R package version 2.0.1. Retrieved from https://cran.r-project.org/package=CalibrationCurves
33. De Cock Campo, B. (2023). Towards reliable predictive analytics: A generalized calibration framework. *arXiv preprint,* arXiv:2309.08559. https://arxiv.org/abs/2309.08559
34. Warnes, G., Bolker, B., Lumley, T., Magnusson, A., Venables, B., Ryodan, G., & Moeller, S. (2023). *gtools: Various R Programming Tools.* R package version 3.9.5. Retrieved from https://github.com/r-gregmisc/gtools
35. Wickham, H. (2007). Reshaping data with the reshape package. *Journal of Statistical Software, 21*(12), 1–20. Retrieved from http://www.jstatsoft.org/v21/i12/
36. Wickham, H. (2016). *ggplot2: Elegant Graphics for Data Analysis.* Springer-Verlag New York. ISBN 978-3-319-24277-4
37. Wickham, H. (2023). *forcats: Tools for Working with Categorical Variables (Factors).* R package version 1.0.0. Retrieved from https://CRAN.R-project.org/package=forcats
38. Wickham, H. (2023). *stringr: Simple, Consistent Wrappers for Common String Operations.* R package version 1.5.1. Retrieved from https://CRAN.R-project.org/package=stringr
39. Wickham, H., Averick, M., Bryan, J., Chang, W., McGowan, L. D., François, R., Grolemund, G., Hayes, A., Henry, L., Hester, J., Kuhn, M., Pedersen, T. L., Miller, E., Bache, S. M., Müller, K., Ooms, J., Robinson, D., Seidel, D. P., Spinu, V., Takahashi, K., Vaughan, D., Wilke, C., Woo, K., & Yutani, H. (2019). Welcome to the tidyverse. *Journal of Open Source Software, 4*(43), 1686. https://doi.org/10.21105/joss.01686

40. Wickham, H., François, R., Henry, L., Müller, K., & Vaughan, D. (2023). *dplyr: A Grammar of Data Manipulation.* R package version 1.1.4. Retrieved from https://CRAN.R-project.org/package=dplyr
41. Wickham, H., & Henry, L. (2023). *purrr: Functional Programming Tools.* R package version 1.0.2. Retrieved from https://CRAN.R-project.org/package=purrr
42. Wickham, H., Hester, J., & Bryan, J. (2024). *readr: Read Rectangular Text Data.* R package version 2.1.5. Retrieved from https://CRAN.R-project.org/package=readr
43. Wickham, H., Pedersen, T., & Seidel, D. (2023). *scales: Scale Functions for Visualization.* R package version 1.3.0. Retrieved from https://CRAN.R-project.org/package=scales
44. Wickham, H., Vaughan, D., & Girlich, M. (2024). *tidyr: Tidy Messy Data.* R package version 1.3.1. Retrieved from https://CRAN.R-project.org/package=tidyr
45. Wright, M. N., & Ziegler, A. (2017). ranger: A fast implementation of random forests for high dimensional data in C++ and R. *Journal of Statistical Software, 77*(1), 1–17. https://doi.org/10.18637/jss.v077.i01
46. Yoshida, K., & Bartel, A. (2022). *tableone: Create "Table 1" to Describe Baseline Characteristics with or without Propensity Score Weights.* R package version 0.13.2. Retrieved from https://github.com/kaz-yos/tableone

S2. Causal Machine Learning Methods

Metalearners

Metalearners are flexible, model-agnostic algorithms designed to estimate ITE by decomposing the estimation process into multiple prediction tasks, each solvable by machine learning models[1,2]. Notable examples include the T-learner, S-learner, X-learner and DR-learner. T-learner models train two prediction models separately for the treatment ($P(Y_i = 1|T = 1, X = x_i)$) and control groups ($P(Y_i = 1|T = 0, X = x_i)$). T-learners allow for the use of different models in the treated and control groups. However, to facilitate efficient model comparison, researchers typically constrain both arms to use the same type of model. S-learners incorporate the treatment assignment variable into the covariates list and train a single model. To account for potential variations in treatment effects among individuals, one way is to introduce interaction terms between treatment and covariates for the linear model or directly apply nonlinear models. Additionally, spline functions and penalization techniques are applied to address potential model misspecifications. X-learner first models outcomes for each group, imputes counterfactual outcomes to estimate treatment effects, and then refines these estimates through weighted averaging[2]. DR-learner combines pseudo-outcome regression and propensity score models using a double robust approach to correct for selection bias between treatment groups[3,4].

Tree-based methods

Tree-based methods, such as causal tree[5], causal forest[6], model-based recursive partitioning[7,8], and Bayesian causal forest[9], are designed to partition the covariate space in such a way that the resulting subgroups exhibit heterogeneous treatment effects. Causal forest extends random forest and integrates an honesty mechanism in the training process to overcome potential over-fitting issues[10]. Model-based recursive partitioning automatically identifies patient subgroups with differential treatment effects by segmenting a model of the overall treatment effect and linking subgroups to predictive factors using a decision tree approach. Bayesian causal forest incorporates an estimate of the propensity function into the response model and separately regularizes treatment effect heterogeneity. It can address biases from confounding and improves control over effect estimation. Causal survival forests extend generalized random forest to handle right-censored survival outcomes, enabling estimation of heterogeneous treatment effects for survival probabilities at a fixed horizon or restricted mean survival time[11]. Our study focused on validating widely used causal ML methods for fixed-time outcomes (death or death/dependency) and therefore did not require survival modeling. Nevertheless, causal survival forests could be applied to the same data to explore treatment effect heterogeneity across survival curves. Future work may consider such approaches to complement fixed-time analyses and assess whether survival-based methods improve generalizability.

Deep learning methods

Deep learning methods model the relationship between covariates and outcomes using neural networks. Conditional Variational Autoencoders (CVAE) learns a latent variable representation of treatment and covariates to estimate individualized treatment effects[12]. Generative Adversarial Nets for inference of Individualized Treatment Effects (GANITE)[13] uses adversarial learning to capture the uncertainty in counterfactual outcomes.

S3. ITE Evaluation Metrics

Standard diagnostics, such as ITE density plots and outcome calibration curves, provide limited insight into the validity of ITE estimates because they do not directly evaluate the causal estimation process. The density plots summarize only the distribution of the model-predicted ITEs without incorporating outcomes. And stability across random splits does not imply that the estimated ITEs correspond to true treatment-effect heterogeneity. Outcome calibration curves assess predictive performance for outcomes, not the validity of effect heterogeneity. Therefore, both metrics cannot reveal whether the estimated ITEs reflect the treatment–outcome relationship present in the randomized data.

To address these limitations, we proposed evaluation tools that directly link the estimated ITEs to the empirical treatment effects in the trial data: the outcome–ITE plot, the subgroup ATE–ITE plot, and the benefit–harm density plot. In the outcome–ITE plot, the fitted outcome curves under treatment and control represent the observed probabilities as a function of the estimated ITE. The vertical separation between these curves is an empirical treatment effect curve, which should align with the estimated ITEs on the x-axis. This allows a direct assessment of whether the estimated ITEs are supported by the observed treatment–outcome pattern at the individual level, enabling researchers to identify for whom the estimates appear plausible and for whom they may not.

The subgroup ATE–ITE plot groups individuals by estimated ITEs and compares the grouping with empirical average treatment effects. If the model produces valid estimates, the observed ATEs should increase as the estimated ITE increases. The benefit–harm density plots address the instability that may arise from a single training–test split. By repeatedly fitting the model and computing empirical ATEs for groups predicted to benefit or be harmed, these plots assess whether the sign of the estimated ITE is consistently supported by the data. When treatment is beneficial (ITE < 0), the corresponding empirical ATEs should also be negative, and vice versa. The quantitative validation metrics are similarly more informative than ITE density plots, as they provide numerical summaries that reflect the same causal and empirical consistency principles.

These visual and quantitative ITE evaluation metrics, complement traditional density and calibration metrics. They directly evaluate whether the estimated ITEs are consistent with other causal estimates, such as the ATEs. They also assess the empirical consistency of the estimates across training and test datasets obtained from random train–test splits. These tools contribute to ITE evaluation that links model-based estimates to empirical treatment effects, enables assessment at both the individual and subgroup levels, and improves the interpretability of heterogeneous treatment effect analyses.

S4. Simulation study

We generated simulated data to systematically evaluate the performance of causal ML models in estimating heterogeneous treatment effects. The simulation was designed to mimic the scale and structure of the stroke RCTs while ensuring a fully known data-generating process.

We assumed binary outcome and treatment, where the log-odds of the outcome was modeled as a function of covariates and treatment, incorporating both a global treatment effect and covariate-interactive treatment effects. We explored scenarios involving continuous covariates alone, a mix of continuous and discrete covariates, as well as both high- and low-dimensional covariate settings. By construction, treatment was randomized, eliminating confounding. Effect modifiers were explicitly introduced through interaction terms, whereas non-modifying covariates served as prognostic factors.

A sample size of 20,000 simulated observations was chosen to reflect the scale of the stroke trials. To check model validity, we applied the causal ML methods that were tested above and compared their performance through internal validation using training-test splits. The internal validation process was repeated for both metalearners and tree-based methods using the simulated data. Additionally, to assess the impact of variable selection, we validated the models using only variables with near-zero treatment-covariate interaction coefficients (i.e., non-modifying variables). This enabled us to examine how prior knowledge of variables influences the stability and validity of HTE estimation. The controlled data-generating process ensured the true individualized treatment effects were known and provided a gold standard for performance evaluation.

Design and covariates. Each simulated individual was assigned to a binary treatment arm ($T = 0$ or 1) with equal probability (0.5), reflecting randomization. Baseline covariates (X) were generated depending on the simulation setting. Continuous covariates were drawn from a multivariate normal distribution with mean zero and a compound symmetric covariance structure ($\rho = 0.1, \sigma = 1$). Discrete covariates were generated as categorical variables with 4 possible values and randomly assigned probabilities. A fixed random seed ($set.seed(1234)$) was used to ensure reproducibility.

Outcome generation. Outcomes were binary and modeled through a logistic regression framework:

$$logit\big(P(Y=1)\big) = \beta_0 + \beta_T T + \sum_j \beta_j X^j + \sum_j \beta_{Tj}\big(TX^j\big),$$

with coefficients randomly drawn from prespecified ranges ($\beta_0 \sim U[-1,1]$, $\beta_T \sim U[-0.5,0.5]$, $\beta_j \sim U[-1,1]$, $\beta_{Tj} \sim U[-0.5,0.5]$). Outcomes were sampled from a Bernoulli distribution with probability given by the logistic function.

True treatment effects. For each individual, potential outcomes under treatment ($Y^{(1)}$) and control ($Y^{(0)}$) were computed directly from the logistic model, holding treatment fixed at 1 or 0, respectively. The true individualized treatment effect (ITE) was then defined as $\text{ITE}_i^{\text{true}} = P\left(Y_i^{(1)} = 1 |\, X_i\right) - P\left(Y_i^{(0)} = 1 |\, X_i\right)$. This ensured that the ground truth for individualized treatment effects was fully known.

Simulation settings. We investigated four distinct settings to evaluate model performance under different data structures.

- **Setting I:** 20 continuous covariates, binary treatment, and binary outcome. All covariates were used to model ITEs, with true ITEs determined by a logistic function incorporating a global treatment effect and covariate–treatment interactions. Results are presented in Figure S7.1.1 and Figure S7.1.2.
- **Setting II:** 20 continuous covariates, binary treatment, and binary outcome. Only six covariates with minimal influence on treatment effect heterogeneity were used to train the causal ML models, simulating variable selection challenges. The true ITEs were generated as in Setting I. Results are presented in Figure S7.2.1 and Figure S7.2.2.
- **Setting III:** 10 continuous covariates and 10 discrete covariates, binary treatment, and binary outcome. All covariates contributed to modeling ITEs, again using a logistic function with global and interaction effects. Results are presented in Figure S7.3.1 and Figure S7.3.2.
- **Setting IV:** 20 continuous covariates, binary treatment, and binary outcome. All covariates were used to model ITEs. The true ITEs were modelled by logistic function with a near-zero global treatment effect and no covariates-interactive treatment effects. This setting is identical to Setting I, except that the covariate–treatment interaction terms were excluded. Results are presented in Figure S7.4.1 and Figure S7.4.2.

Implementation. All simulations were implemented in R, with random number generation controlled by a fixed seed (1234) to ensure reproducibility. Full code is publicly available in our open GitLab repository (https://gitlab.uzh.ch/hongruyu.chen/causal_ml-for-ite).

References

1. Feuerriegel S, Frauen D, Melnychuk V, et al. Causal machine learning for predicting treatment outcomes. *Nat Med*. 2024;30(4):958-968. doi:10.1038/s41591-024-02902-1
2. Künzel SR, Sekhon JS, Bickel PJ, Yu B. Metalearners for estimating heterogeneous treatment effects using machine learning. *Proceedings of the National Academy of Sciences*. 2019;116(10):4156-4165. doi:10.1073/pnas.1804597116
3. Nie X, Wager S. Quasi-oracle estimation of heterogeneous treatment effects. *Biometrika*. 2021;108(2):299-319. doi:10.1093/biomet/asaa076
4. Kennedy EH. Towards optimal doubly robust estimation of heterogeneous causal effects. *Electronic Journal of Statistics*. 2023;17(2):3008-3049. doi:10.1214/23-EJS2157
5. Athey S, Imbens G. Recursive partitioning for heterogeneous causal effects. *Proc Natl Acad Sci USA*. 2016;113(27):7353-7360. doi:10.1073/pnas.1510489113
6. Wager S, Athey S. Estimation and Inference of Heterogeneous Treatment Effects using Random Forests. *Journal of the American Statistical Association*. 2018;113(523):1228-1242. doi:10.1080/01621459.2017.1319839
7. Seibold H, Zeileis A, Hothorn T. Model-Based Recursive Partitioning for Subgroup Analyses. *The International Journal of Biostatistics*. 2016;12(1):45-63. doi:10.1515/ijb-2015-0032
8. Seibold H, Zeileis A, Hothorn T. model4you: An R Package for Personalised Treatment Effect Estimation. *Journal of Open Research Software*. 2019;7(1). doi:10.5334/jors.219
9. Hahn PR, Murray JS, Carvalho CM. Bayesian Regression Tree Models for Causal Inference: Regularization, Confounding, and Heterogeneous Effects (with Discussion). *Bayesian Analysis*. 2020;15(3):965-1056. doi:10.1214/19-BA1195
10. Jawadekar N, Kezios K, Odden MC, et al. Practical Guide to Honest Causal Forests for Identifying Heterogeneous Treatment Effects. *Am J Epidemiol*. 2023;192(7):1155-1165. doi:10.1093/aje/kwad043
11. Cui Y, Kosorok MR, Sverdrup E, Wager S, Zhu R. Estimating heterogeneous treatment effects with right-censored data via causal survival forests. *J R Stat Soc Ser B Stat Methodol*. 2023;85(2):179-211. doi:10.1093/jrsssb/qkac001
12. Sohn K, Lee H, Yan X. Learning Structured Output Representation using Deep Conditional Generative Models. In: *Advances in Neural Information Processing Systems*. Vol 28. Curran Associates, Inc.; 2015. Accessed December 9, 2024. https://proceedings.neurips.cc/paper_files/paper/2015/file/8d55a249e6baa5c06772297520da2051-Paper.pdf
13. Yoon J, Jordon J, Schaar M van der. GANITE: Estimation of Individualized Treatment Effects using Generative Adversarial Nets. In: 2018. Accessed December 9, 2024. https://openreview.net/forum?id=ByKWUeWA-

Table S1. Summary of quantitative validation metrics on CAST training and test dataset of 17 causal machine learning methods with 4 weeks' death or dependency outcome.

		**c-for-benefit (training)**	**c-for-benefit (test)**	**mbcb (training)**	**mbcb (test)**	**Calibration-based pseudo R-squared (training)**	**Calibration-based pseudo R-squared (test)**
**T-learner**	Logistic Regression	0.539	0.505	0.542	0.542	0.925	-1.102
	Random Forest	0.963	0.494	0.714	0.633	0.255	-3.034
	Support Vector Machine	0.512	0.495	0.578	0.577	-3.525	-5.675
	XGBoost	0.649	0.500	0.600	0.600	0.590	-4.154
	Penalized Logistic Regression	0.529	0.514	0.533	0.533	0.780	0.083
**S-learner**	Logistic Regression	0.502	0.494	0.505	0.505	0.125	-0.088
	Penalized Logistic Regression	0.520	0.504	0.517	0.517	0.611	0.077
	XGBoost	0.660	0.499	0.558	0.556	0.322	-4.402
	BART	0.538	0.506	0.521	0.521	0.367	-0.758
**X-learner**	Random Forest	0.901	0.502	0.660	0.598	0.216	-18.731
	BART	0.549	0.506	0.541	0.540	0.592	-2.660
**DR-learner**	Random Forest	0.837	0.492	0.502	0.501	0.638	-8.926
**Tree-based methods**	Causal Forest	0.569	0.504	-	-	0.112	-0.065
	Bayesian Causal Forest	0.526	0.509	-	-	0.092	0.014
	Model-based Recursive Partitioning	0.757	0.510	-	-	-	-
**Deep Learning**	CVAE	0.530	0.496	0.533	0.532	0.540	-1.103
	GANITE	0.519	0.492	0.525	0.525	0.290	-1.243

Notes: Consistent values across training and test data that are closer to 1 indicate a better model fit. The dash symbol indicates instances where the validation metric could not be assessed due to the model's mechanism (mbcb can be only used for models that provide explicit potential outcomes predictions under both treatment and control).
Abbreviations: CAST: the Chinese Acute Stroke Trial.

Table S2. Summary of quantitative validation metrics on the combined IST and CAST dataset of 17 causal machine learning methods. The outcome variable is death at 14 days for IST and death at 4 weeks for CAST.

		**c-for-benefit (training)**	**c-for-benefit (test)**	**mbcb (training)**	**mbcb (test)**	**Calibration-based pseudo R-squared (training)**	**Calibration-based pseudo R-squared (test)**
**T-learner**	Logistic Regression	0.544	0.515	0.540	0.539	0.668	-1.565
	Random Forest	0.998	0.480	0.785	0.657	0.216	-3.684
	Support Vector Machine	0.540	0.496	0.554	0.554	0.578	-1.579
	XGBoost	0.734	0.498	0.620	0.617	0.528	-2.591
	Penalized Logistic Regression	0.500	0.501	-	-	-	-
**S-learner**	Logistic Regression	0.517	0.501	0.514	0.514	0.625	-0.413
	Penalized Logistic Regression	0.500	0.500	-	-	-	-
	XGBoost	0.742	0.507	0.564	0.561	0.316	-2.114
	BART	0.553	0.511	0.529	0.528	0.404	-0.545
**X-learner**	Random Forest	0.986	0.479	0.755	0.643	0.139	-2.297
	BART	0.612	0.505	0.547	0.546	0.395	-0.669
**DR-learner**	Random Forest	0.793	0.506	0.494	0.497	0.715	-5.365
**Tree-based methods**	Causal Forest	0.551	0.492	-	-	0.112	-0.177
	Bayesian Causal Forest	0.531	0.496	-	-	0.131	-0.134
	Model-based Recursive Partitioning	0.825	0.489	-	-	-	-
**Deep Learning**	CVAE	0.516	0.484	0.535	0.536	-0.263	-1.488
	GANITE	0.508	0.484	0.531	0.531	-40.604	-21.871

Notes: Consistent values across training and test data that are closer to 1 indicate a better model fit. The dash symbol indicates instances where:

- The validation metric could not be assessed due to the model's mechanism.
- The model failed to estimate valid individualized treatment effects.
- The metric was not applicable to the model.

Abbreviations: IST: the International Stroke Trial, CAST: the Chinese Acute Stroke Trial.

Table S3. Summary of quantitative validation metrics on external datasets (IST as training data and CAST as test data) of 17 causal machine learning methods. The outcome variable is death at 14 days for IST and death at 4 weeks for CAST.

		**c-for-benefit (training)**	**c-for-benefit (test)**	**mbcb (training)**	**mbcb (test)**	**Calibration-based pseudo R-squared (training)**	**Calibration-based pseudo R-squared (test)**
**T-learner**	Logistic Regression	0.538	0.504	0.546	0.547	0.684	-2.740
	Random Forest	0.998	0.486	0.773	0.672	0.223	-11.254
	Support Vector Machine	0.534	0.514	0.607	0.615	-0.976	-8.961
	XGBoost	0.717	0.480	0.622	0.627	0.546	-4.505
	Penalized Logistic Regression	0.500	0.500	-	-	-	-
**S-learner**	Logistic Regression	0.503	0.529	0.509	0.508	-0.401	0.326
	Penalized Logistic Regression	0.500	0.500	-	-	-	-
	XGBoost	0.724	0.502	0.564	0.566	0.272	-4.479
	BART	0.551	0.488	0.523	0.527	0.299	-0.496
**X-learner**	Random Forest	0.976	0.484	0.745	0.655	0.142	-3.295
	BART	0.602	0.492	0.547	0.557	0.394	-1.678
**DR-learner**	Random Forest	0.813	0.485	0.499	0.514	0.657	-7.144
**Tree-based methods**	Causal Forest	0.665	0.484	-	-	0.094	-1.374
	Bayesian Causal Forest	0.515	0.485	-	-	0.072	-0.118
	Model-based Recursive Partitioning	0.802	0.481	-	-	-	-
**Deep Learning**	CVAE	0.527	0.523	0.525	0.525	0.467	0.492
	GANITE	0.495	0.515	0.532	0.528	-17.126	-21.885

Notes: Consistent values across training and test data that are closer to 1 indicate a better model fit. The dash symbol indicates instances where:

- The validation metric could not be assessed due to the model's mechanism.
- The model failed to estimate valid individualized treatment effects.
- The metric was not applicable to the model.

Abbreviations: IST: the International Stroke Trial, CAST: the Chinese Acute Stroke Trial.

Table S4. Summary of quantitative validation metrics on external datasets (CAST as training data and IST as test data) of 17 causal machine learning methods. The outcome variable is death at 14 days for IST and death at 4 weeks for CAST.

		**c-for-benefit (training)**	**c-for-benefit (test)**	**mbcb (training)**	**mbcb (test)**	**Calibration-based pseudo R-squared (training)**	**Calibration-based pseudo R-squared (test)**
**T-learner**	Logistic Regression	0.576	0.498	0.572	0.592	0.635	-3.824
	Random Forest	0.993	0.497	0.806	0.655	0.270	-8.679
	Support Vector Machine	0.523	0.497	0.578	0.580	-0.349	-3.363
	XGBoost	0.803	0.504	0.628	0.679	0.482	-9.664
	Penalized Logistic Regression	0.500	0.500	-	-	-	-
**S-learner**	Logistic Regression	0.530	0.500	0.525	0.528	0.635	-1.047
	Penalized Logistic Regression	0.500	0.500	-	-	-	-
	XGBoost	0.790	0.493	0.566	0.593	0.325	-3.351
	BART	0.564	0.500	0.548	0.561	0.683	-4.418
**X-learner**	Random Forest	0.994	0.504	0.766	0.642	0.188	-2.441
	BART	0.644	0.492	0.561	0.567	0.534	-3.154
**DR-learner**	Random Forest	0.776	0.518	0.495	0.495	0.778	-4.909
**Tree-based methods**	Causal Forest	0.600	0.499	-	-	0.190	-0.116
	Bayesian Causal Forest	0.570	0.493	-	-	0.640	-1.839
	Model-based Recursive Partitioning	-	-	-	-	-	-
**Deep Learning**	CVAE	0.523	0.492	0.562	0.551	-0.747	-3.190
	GANITE	0.518	0.488	0.531	0.532	-20.976	-13.067

Notes: Consistent values across training and test data that are closer to 1 indicate a better model fit. The dash symbol indicates instances where:

- The validation metric could not be assessed due to the model's mechanism.
- The model failed to estimate valid individualized treatment effects.
- The metric was not applicable to the model.

Abbreviations: IST: the International Stroke Trial, CAST: the Chinese Acute Stroke Trial.

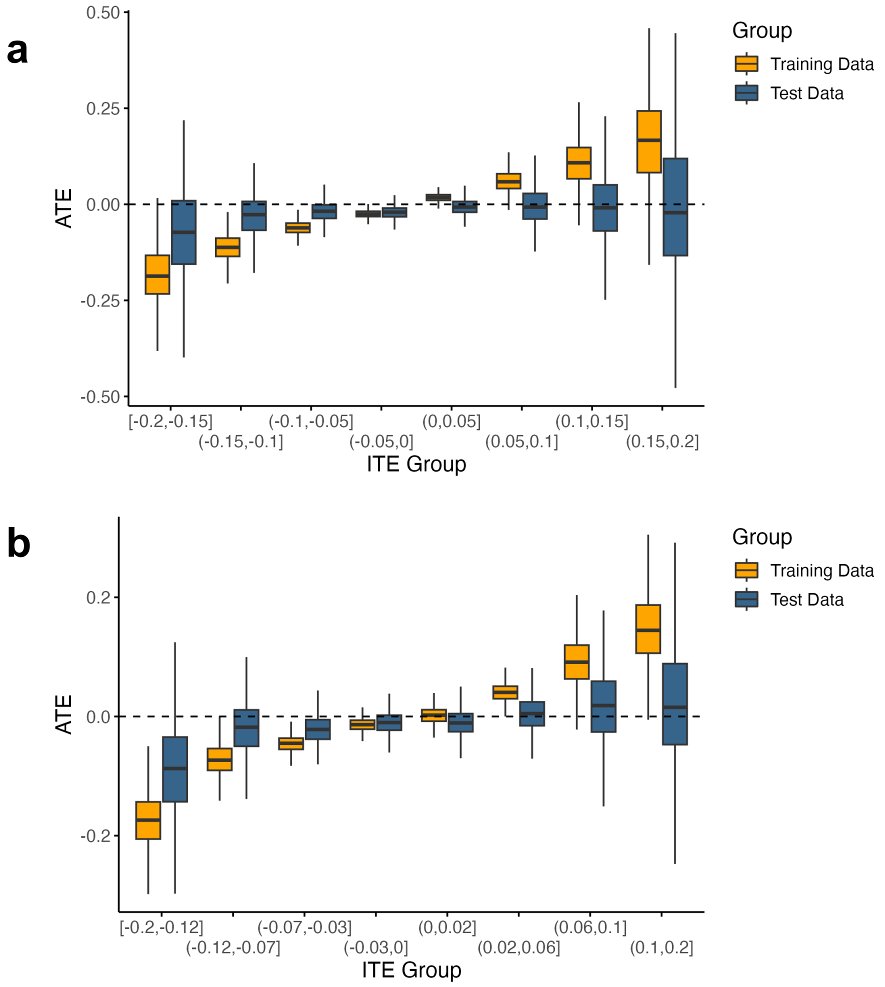

Figure S1. Box plots of ATE in risk ratio within different ITE subgroups from 1000 random train-test split experiments. It compares the trends of ATE between training data (orange bars) and test data (blue bars) across various ITE ranges. The horizontal dashed line at 0 indicates no treatment effects. **A**, ITE estimated by T-learner logistic regression model on the IST dataset with the outcome variable being death or dependency at 6 months. **B**, ITE estimated by T-learner logistic regression model on the CAST dataset with the outcome variable being death or dependency at 4 weeks. Abbreviations: IST: the International Stroke Trial, CAST: the Chinese Acute Stroke Trial, ITE: individualized treatment effect, ATE: average treatment effect.

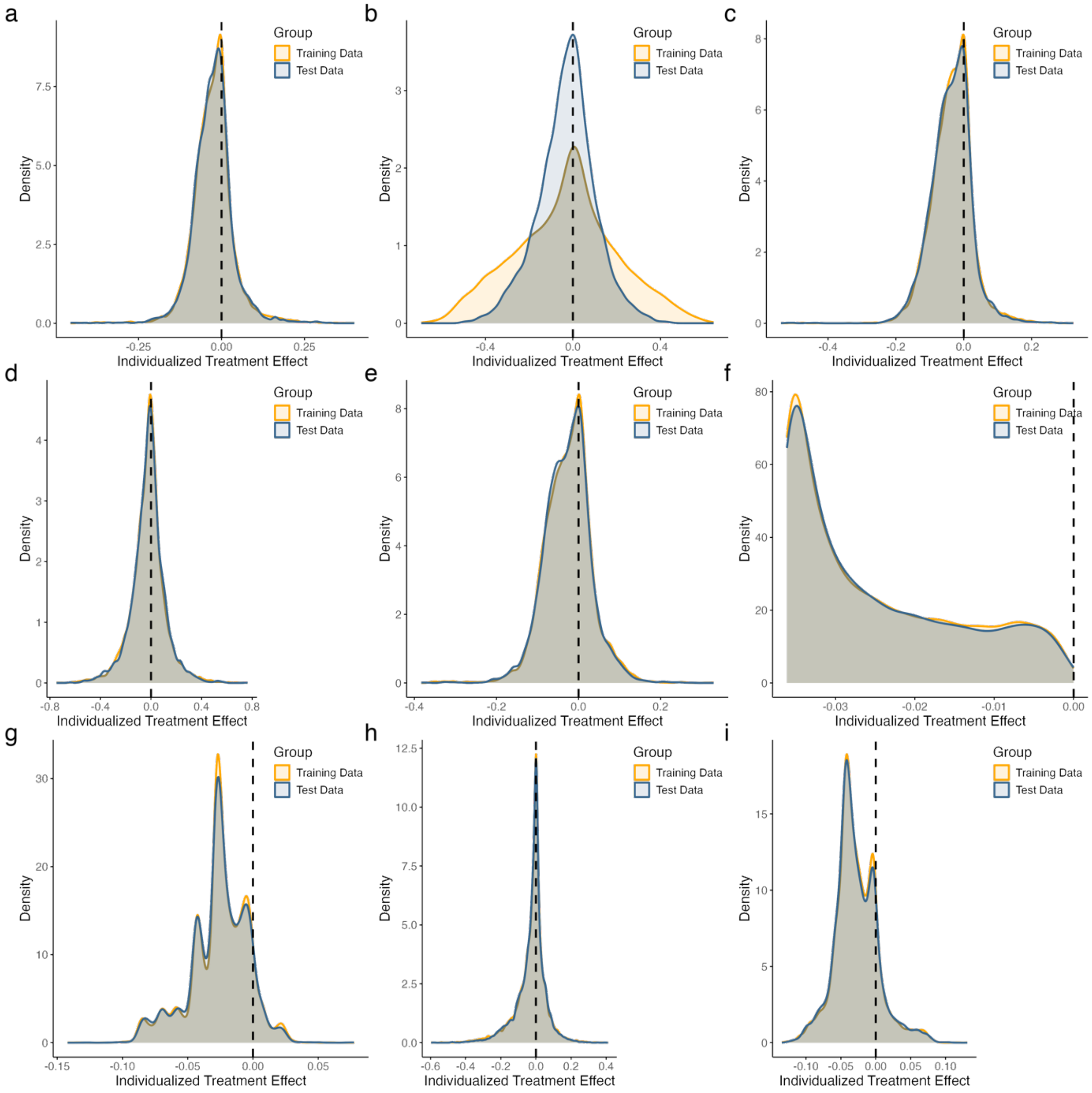

Figure S2.1.1. Internal validation on the IST dataset: density plots of causal machine learning-based individualized treatment effects. Orange represents the training data, and blue indicates the test data. The outcome variable is death or dependency at 6 months. **a**, T-learner Logistic Regression. **b**, T-learner Random Forest. **c**, T-learner Support Vector Machine. **d**, T-learner XGBoost. **e**, T-learner Penalized Logistic Regression. **f**, S-learner Logistic Regression. **g**, S-learner Penalized Logistic Regression. **h**, S-learner XGBoost. **i**, S-learner BART. Abbreviations: IST: the International Stroke Trial, ITE: individualized treatment effect.

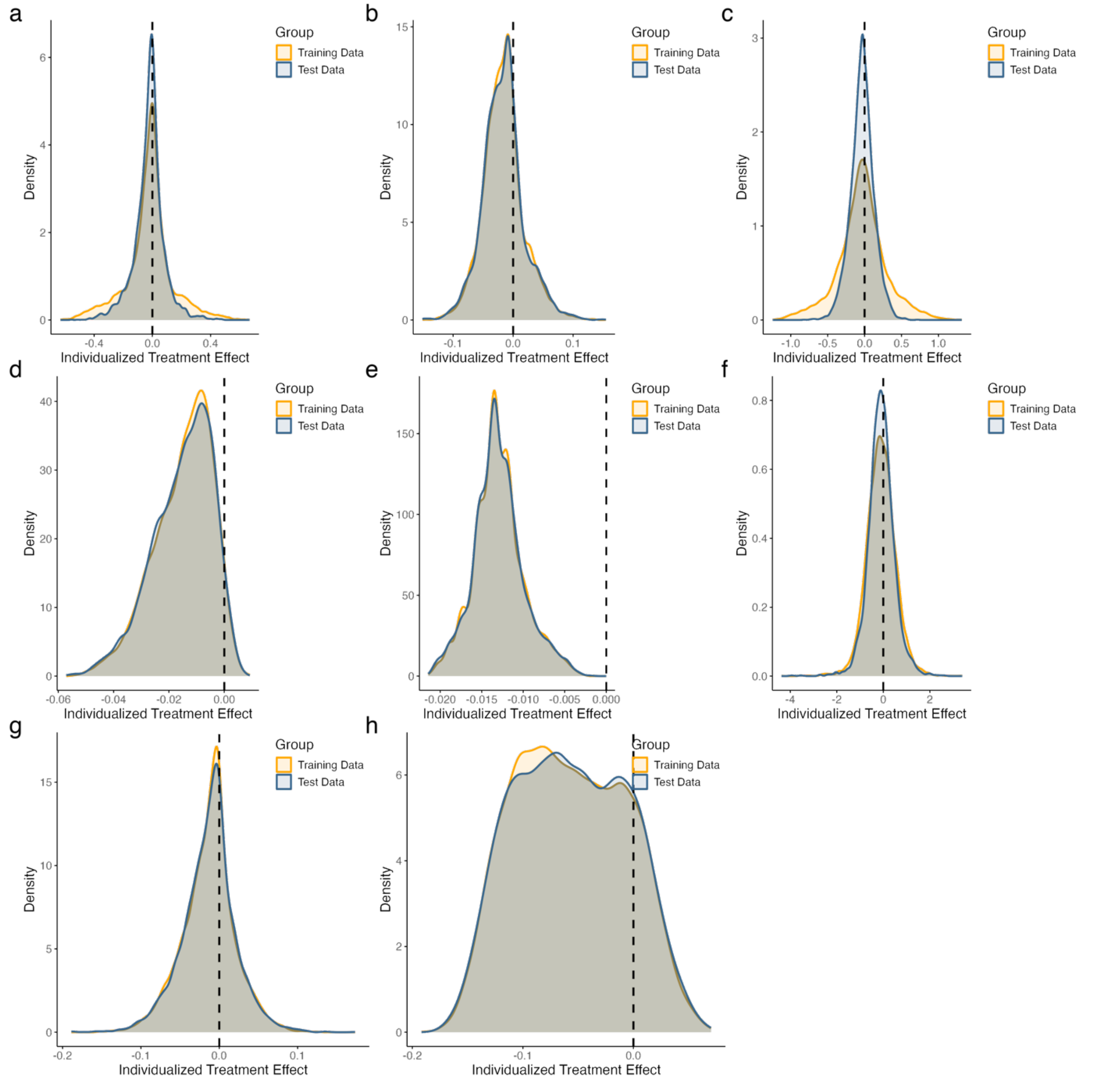

Figure S2.1.2. Internal validation on the IST dataset: density plots of causal machine learning-based individualized treatment effects. Orange represents the training data, and blue indicates the test data. The outcome variable is death or dependency at 6 months. **a**, X-learner Random Forest. **b**, X-learner BART. **c**, DR-learner Random Forest. **d**, Causal Forest. **e**, Bayesian Causal Forest. **f**, Model-based Recursive Partitioning. **g**, CVAE. **h**, GANITE. Abbreviations: IST: the International Stroke Trial, ITE: individualized treatment effect.

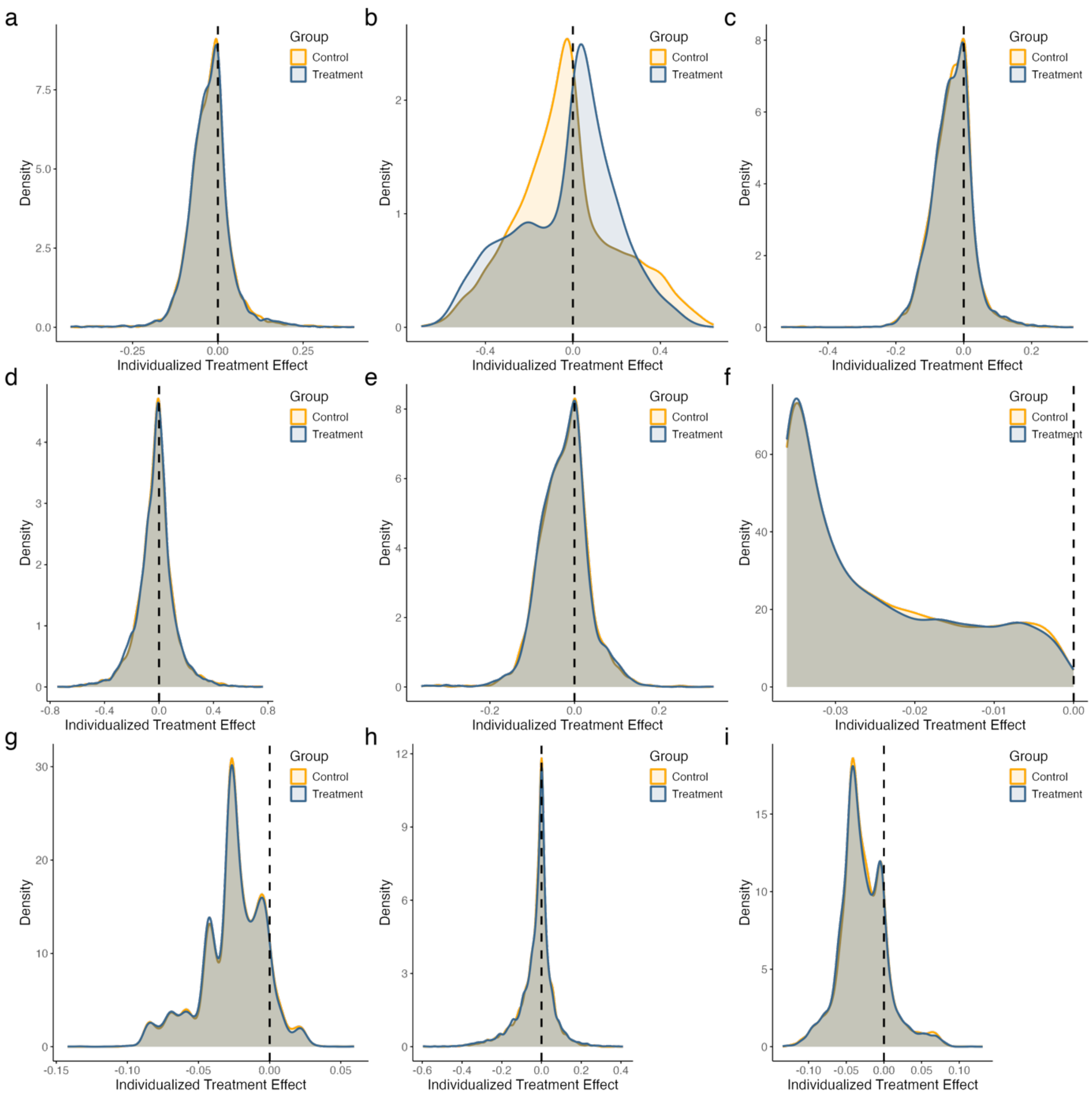

Figure S2.2.1. Internal validation on the IST training dataset: density plots of causal machine learning-based individualized treatment effects. Orange represents the control group, and blue indicates the treatment group. The outcome variable is death or dependency at 6 months. **a**, T-learner Logistic Regression. **b**, T-learner Random Forest. **c**, T-learner Support Vector Machine. **d**, T-learner XGBoost. **e**, T-learner Penalized Logistic Regression. **f**, S-learner Logistic Regression. **g**, S-learner Penalized Logistic Regression. **h**, S-learner XGBoost. **i**, S-learner BART. Abbreviations: IST: the International Stroke Trial, ITE: individualized treatment effect.

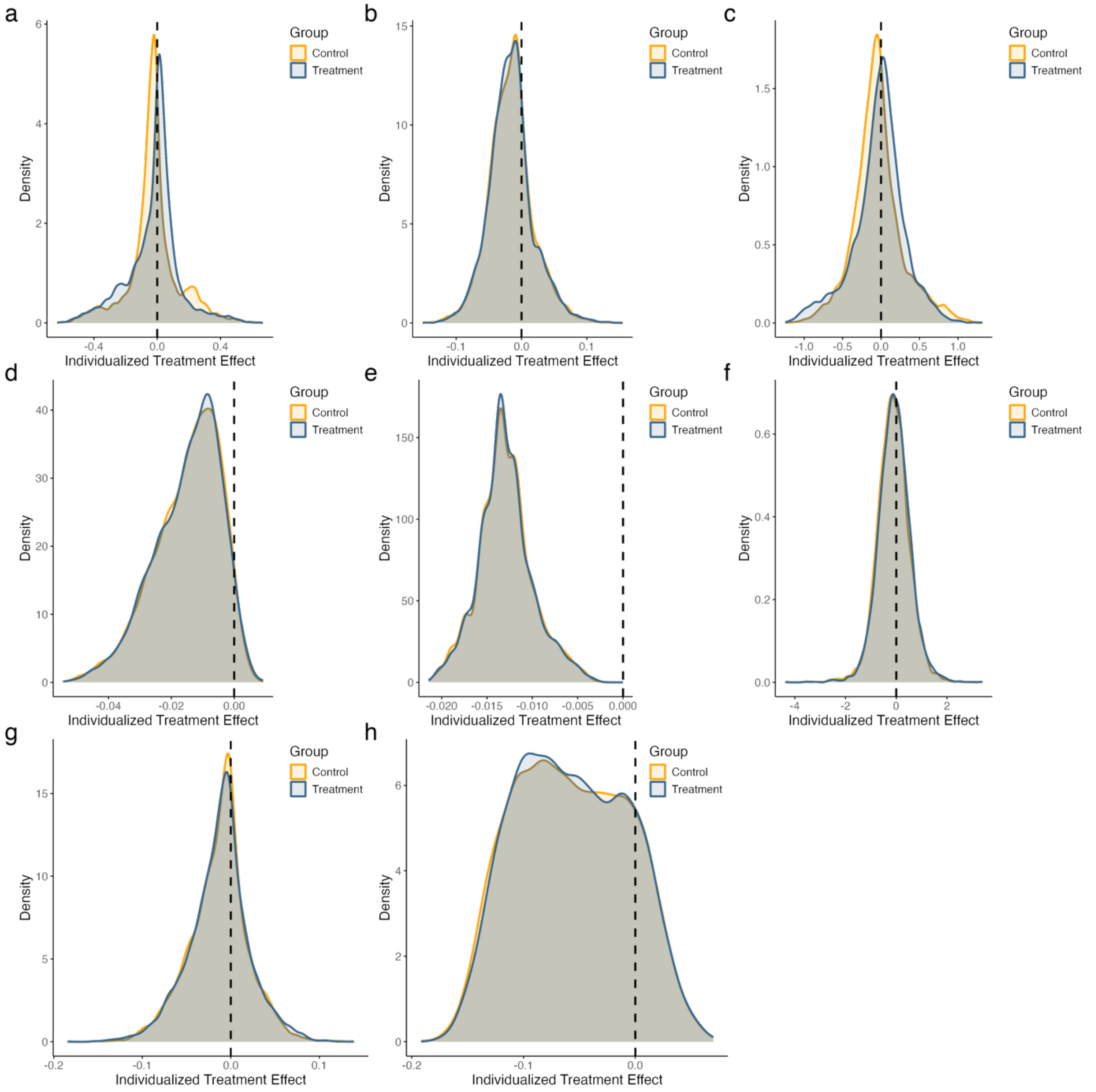

Figure S2.2.2. Internal validation on the IST training dataset: density plots of causal machine learning-based individualized treatment effects. Orange represents the control group, and blue indicates the treatment group. The outcome variable is death or dependency at 6 months. **a**, X-learner Random Forest. **b**, X-learner BART. **c**, DR-learner Random Forest. **d**, Causal Forest. **e**, Bayesian Causal Forest. **f**, Model-based Recursive Partitioning. **g**, CVAE. **h**, GANITE. Abbreviations: IST: the International Stroke Trial, ITE: individualized treatment effect.

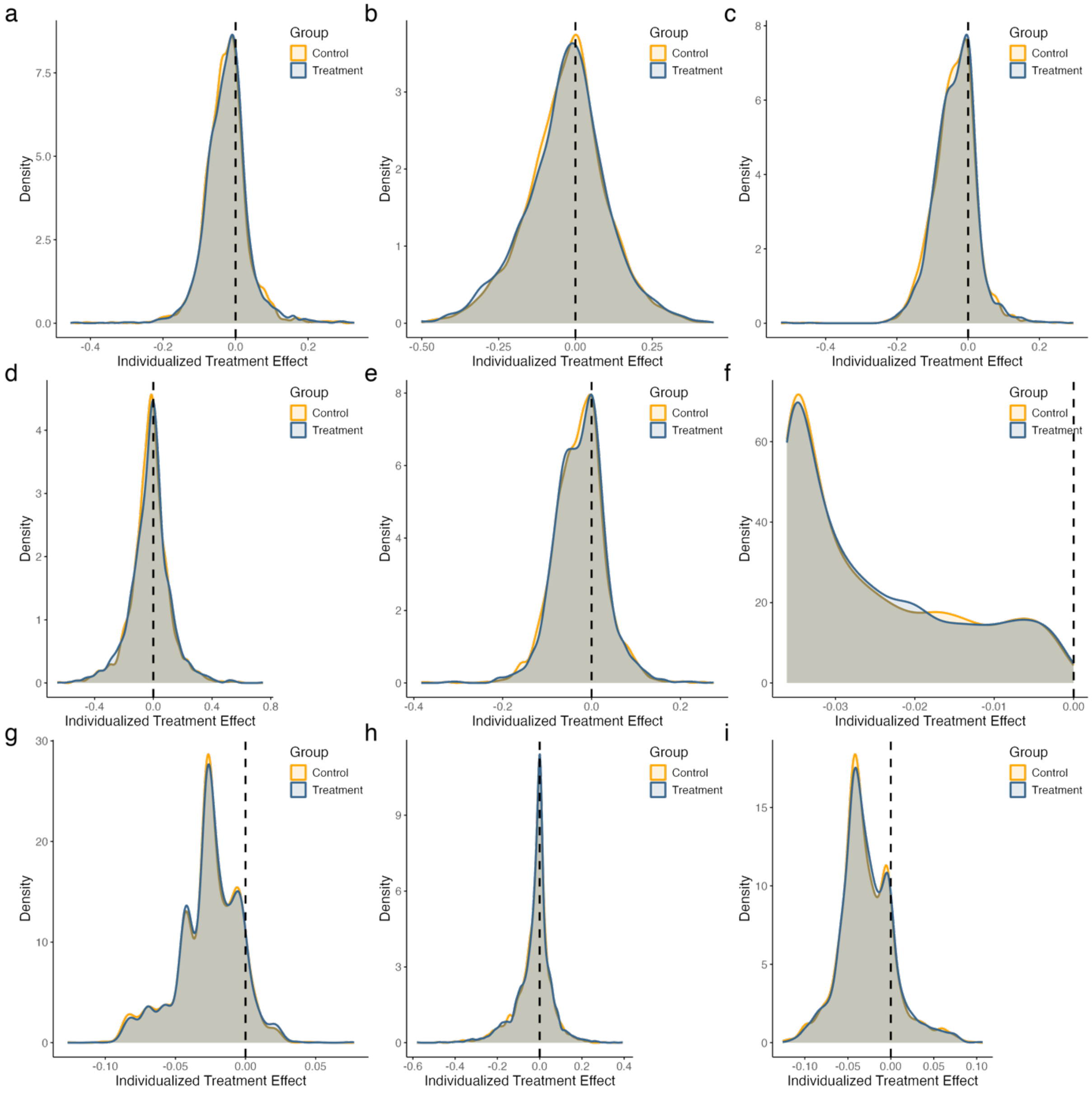

Figure S2.3.1. Internal validation on the IST test dataset: density plots of causal machine learning-based individualized treatment effects. Orange represents the control group, and blue indicates the treatment group. The outcome variable is death or dependency at 6 months. **a**, T-learner Logistic Regression. **b**, T-learner Random Forest. **c**, T-learner Support Vector Machine. **d**, T-learner XGBoost. **e**, T-learner Penalized Logistic Regression. **f**, S-learner Logistic Regression. **g**, S-learner Penalized Logistic Regression. **h**, S-learner XGBoost. **i**, S-learner BART. Abbreviations: IST: the International Stroke Trial, ITE: individualized treatment effect.

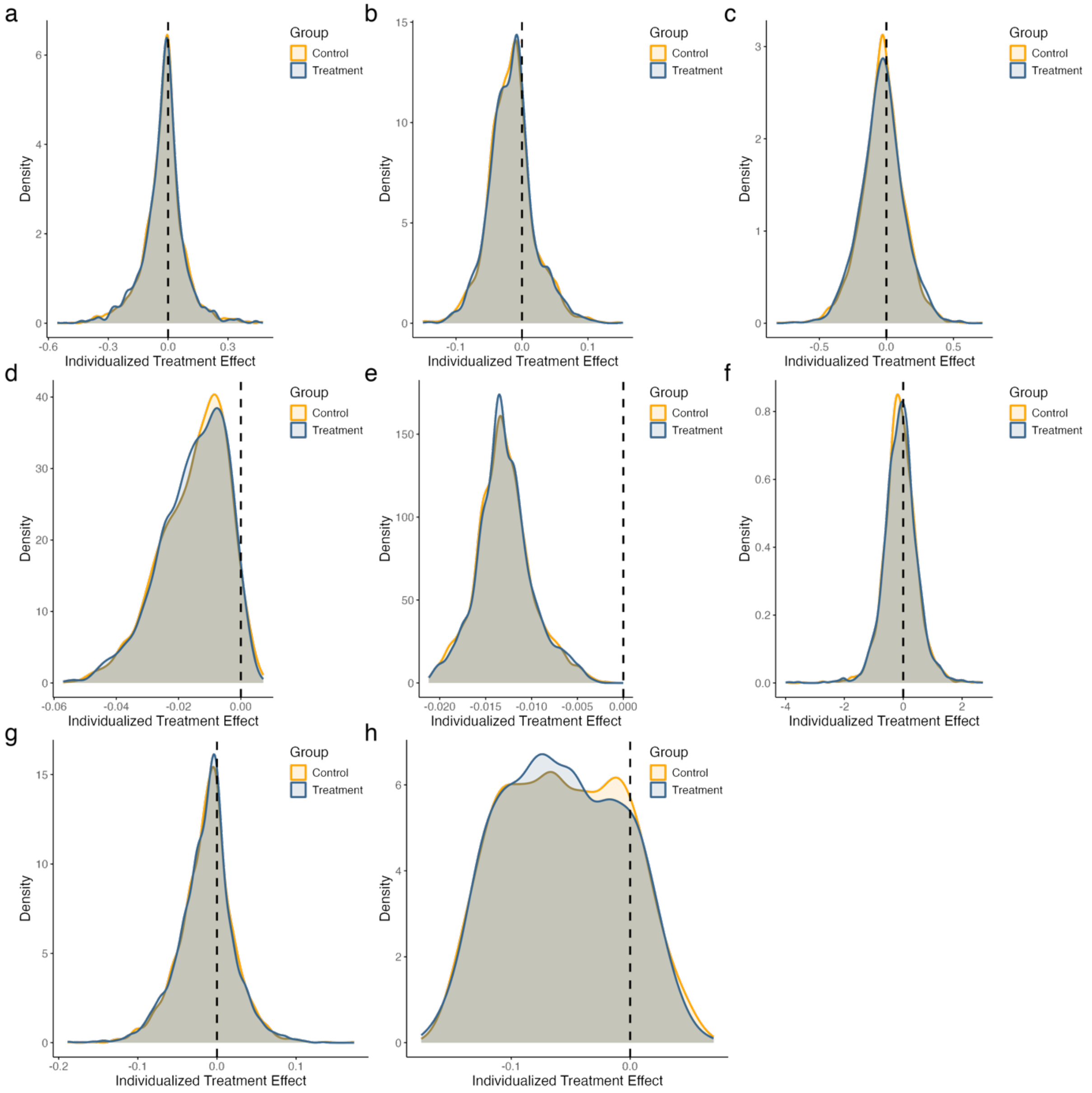

Figure S2.3.2. Internal validation on the IST test dataset: density plots of causal machine learning-based individualized treatment effects. Orange represents the control group, and blue indicates the treatment group. The outcome variable is death or dependency at 6 months. **a**, X-learner Random Forest. **b**, X-learner BART. **c**, DR-learner Random Forest. **d**, Causal Forest. **e**, Bayesian Causal Forest. **f**, Model-based Recursive Partitioning. **g**, CVAE. **h**, GANITE. Abbreviations: IST: the International Stroke Trial, ITE: individualized treatment effect.

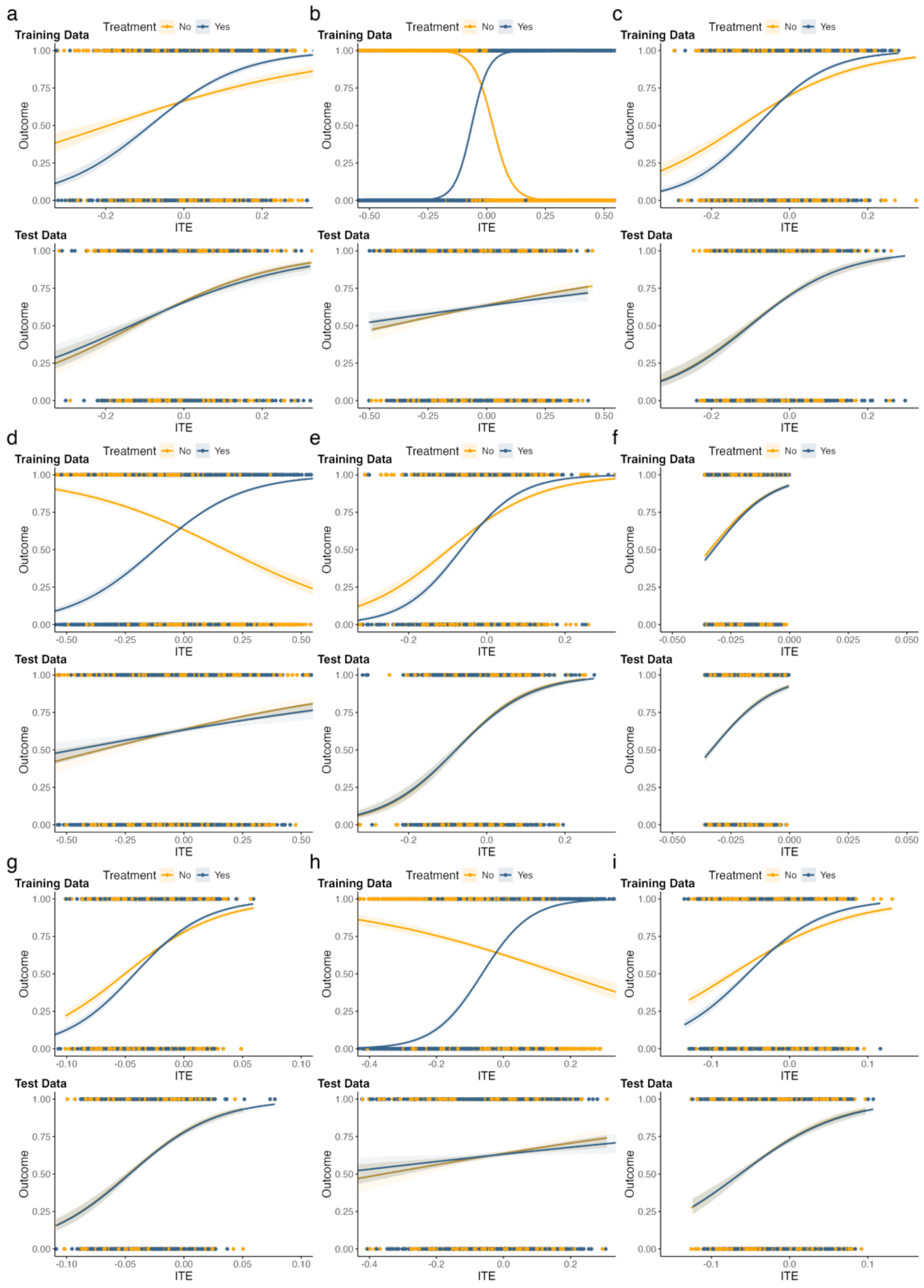

Figure S2.4.1. Internal validation on the IST dataset: outcome-ITE comparative analysis. Dot plots and line plots depict the true and fitted patient outcomes against estimated ITE values between training data and test data. Orange represents the control group, and blue indicates the treatment group. The outcome variable is death or dependency at 6 months. **a**, T-learner Logistic Regression. **b**, T-learner Random Forest. **c**, T-learner Support Vector Machine. **d**, T-learner XGBoost. **e**, T-learner Penalized Logistic Regression. **f**, S-learner Logistic Regression. **g**, S-learner Penalized Logistic Regression. **h**, S-learner XGBoost. **i**, S-learner BART. Abbreviations: IST: the International Stroke Trial, ITE: individualized treatment effect.

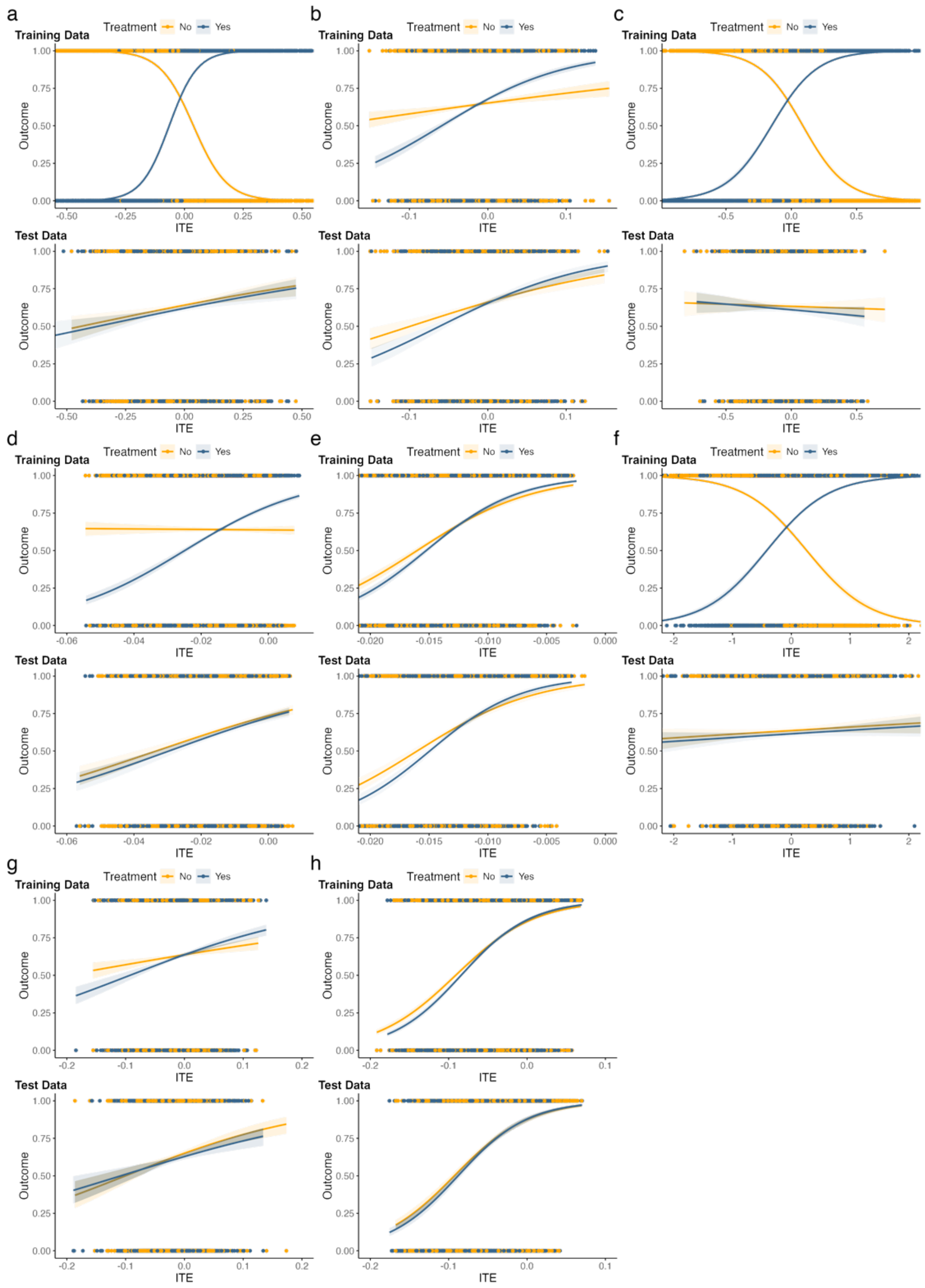

Figure S2.4.2. Internal validation on the IST dataset: outcome-ITE comparative analysis. Dot plots and line plots depict the true and fitted patient outcomes against estimated ITE values between training data and test data. Orange represents the control group, and blue indicates the treatment group. The outcome variable is death or dependency at 6 months. **a**, X-learner Random Forest. **b**, X-learner BART. **c**, DR-learner Random Forest. **d**, Causal Forest. **e**, Bayesian Causal Forest. **f**, Model-based Recursive Partitioning. **g**, CVAE. **h**, GANITE. Abbreviations: IST: the International Stroke Trial, ITE: individualized treatment effect.

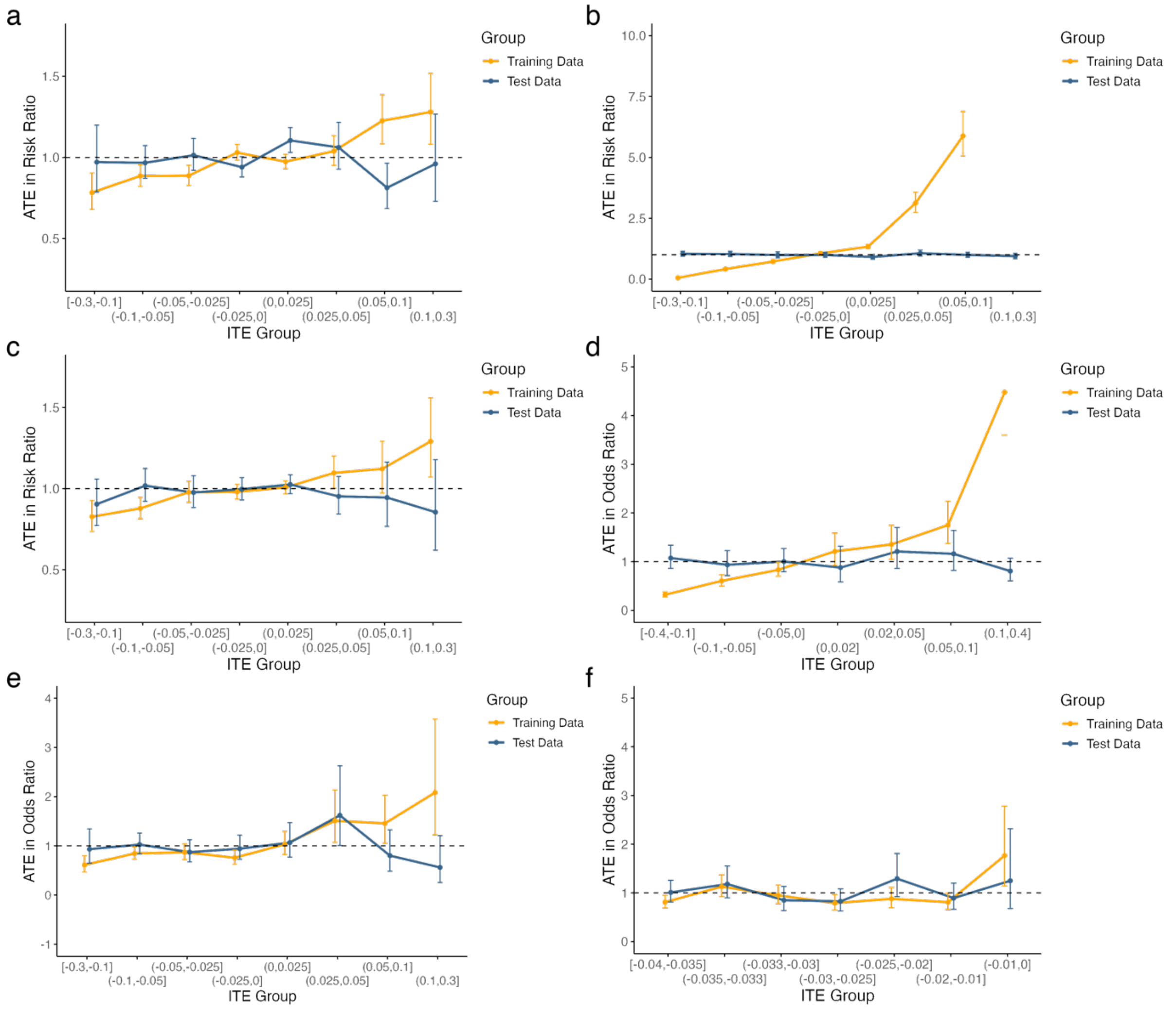

Figure S2.5.1. Internal validation on the IST dataset: ATE-ITE comparative analysis. Line plots depict ATE in risk ratio within different ITE subgroups and provide the confidence intervals at 95% level. Orange represents the training data, and blue indicates the test data. The horizontal dashed line at 1.0 means no treatment effects. The outcome variable is death or dependency at 6 months. **a**, T-learner Logistic Regression. **b**, T-learner Random Forest. **c**, T-learner Support Vector Machine. **d**, T-learner XGBoost. **e**, T-learner Penalized Logistic Regression. **f**, S-learner Logistic Regression. Abbreviations: IST: the International Stroke Trial, ITE: individualized treatment effect, ATE: average treatment effect.

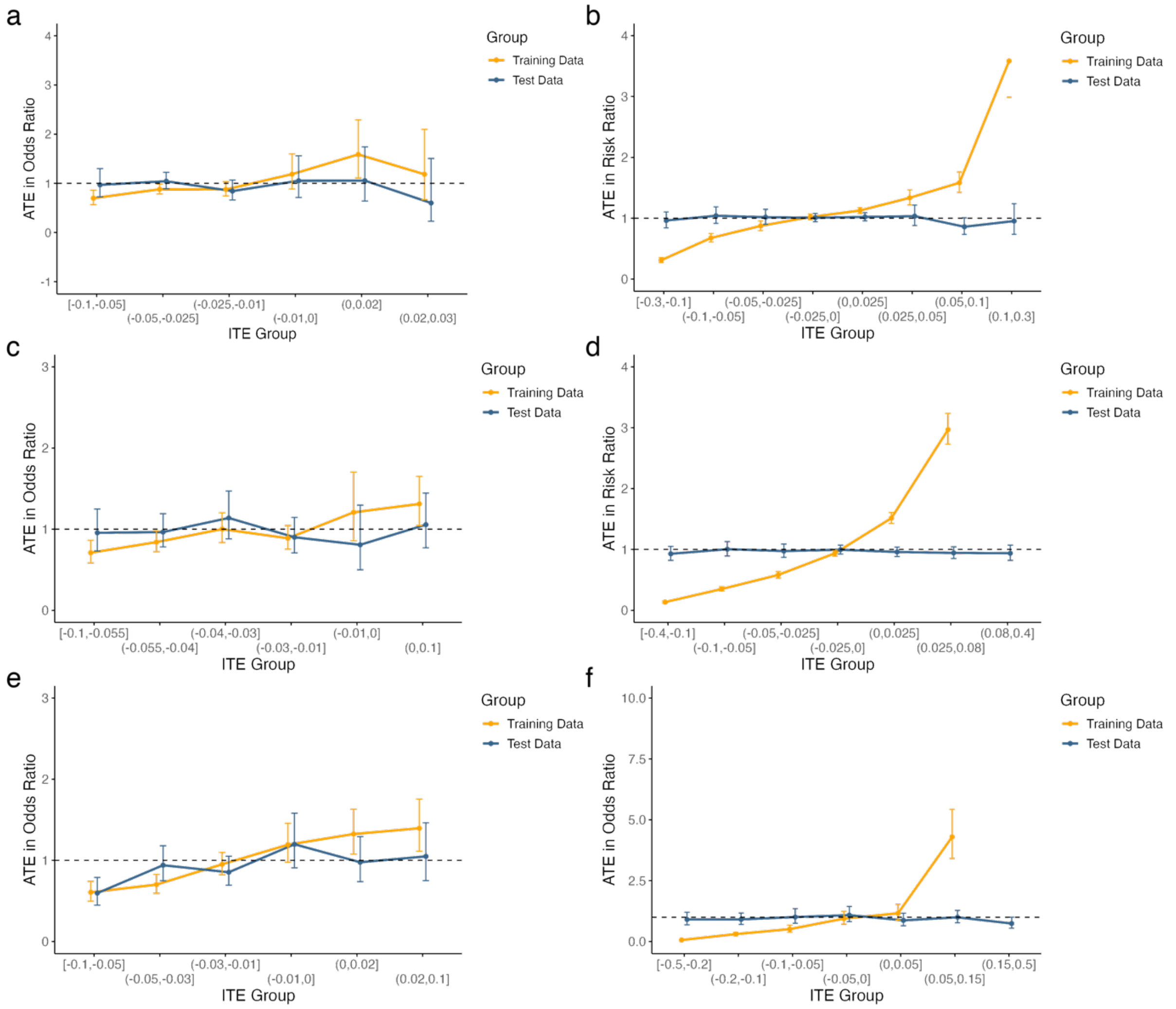

Figure S2.5.2. Internal validation on the IST dataset: ATE-ITE comparative analysis. Line plots depict ATE in risk ratio within different ITE subgroups and provide the confidence intervals at 95% level. Orange represents the training data, and blue indicates the test data. The horizontal dashed line at 1.0 means no treatment effects. The outcome variable is death or dependency at 6 months. **a**, S-learner Penalized Logistic Regression. **b**, S-learner XGBoost. **c**, S-learner BART. **d**, X-learner Random Forest. **e**, X-learner BART. **f**, DR-learner Random Forest. Abbreviations: IST: the International Stroke Trial, ITE: individualized treatment effect, ATE: average treatment effect.

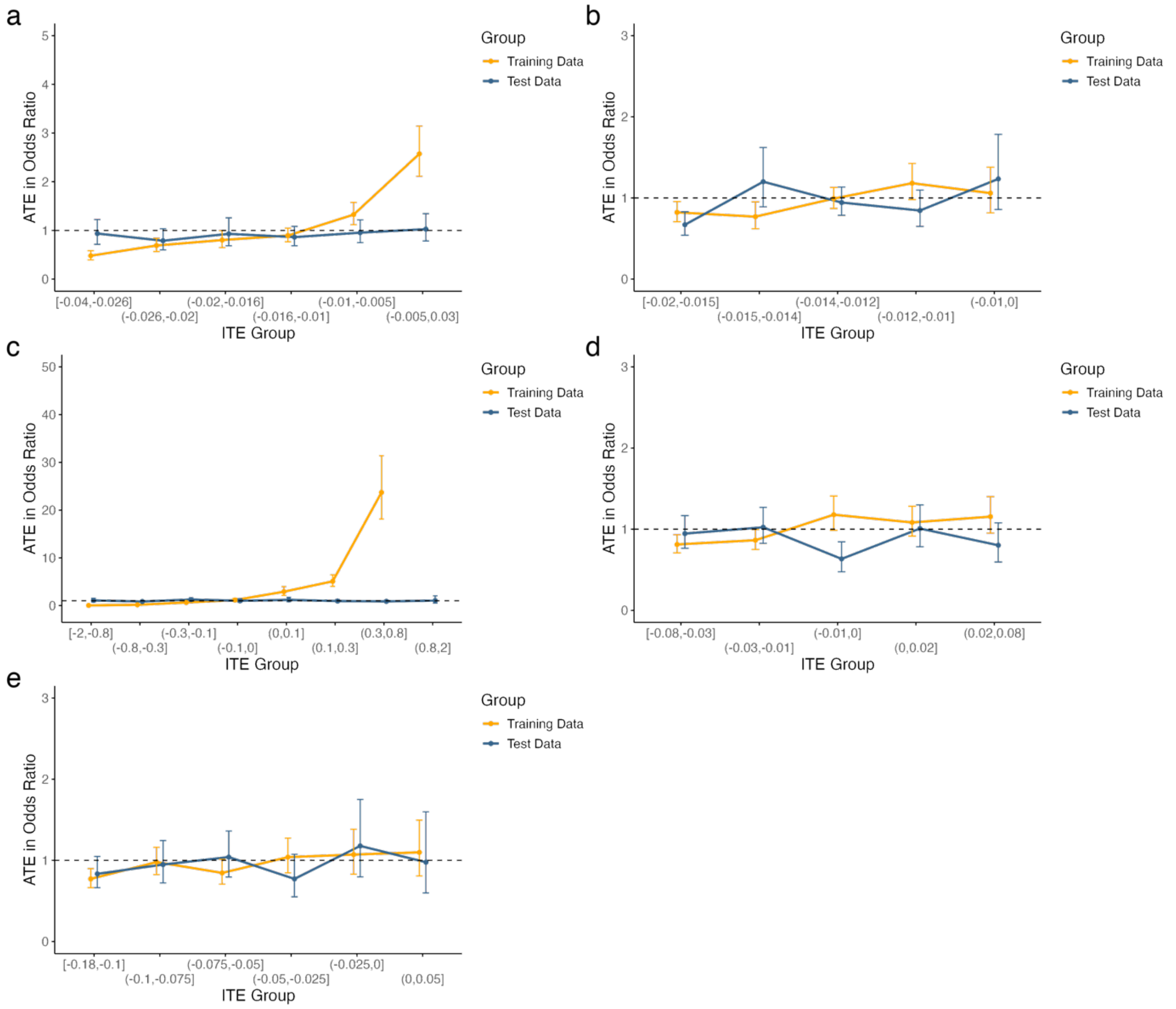

Figure S2.5.2. Internal validation on the IST dataset: ATE-ITE comparative analysis. Line plots depict ATE in risk ratio within different ITE subgroups and provide the confidence intervals at 95% level. Orange represents the training data, and blue indicates the test data. The horizontal dashed line at 1.0 means no treatment effects. The outcome variable is death or dependency at 6 months. **a**, Causal Forest. **b**, Bayesian Causal Forest. **c**, Model-based Recursive Partitioning. **d**, CVAE. **e**, GANITE. Abbreviations: IST: the International Stroke Trial, ITE: individualized treatment effect, ATE: average treatment effect.

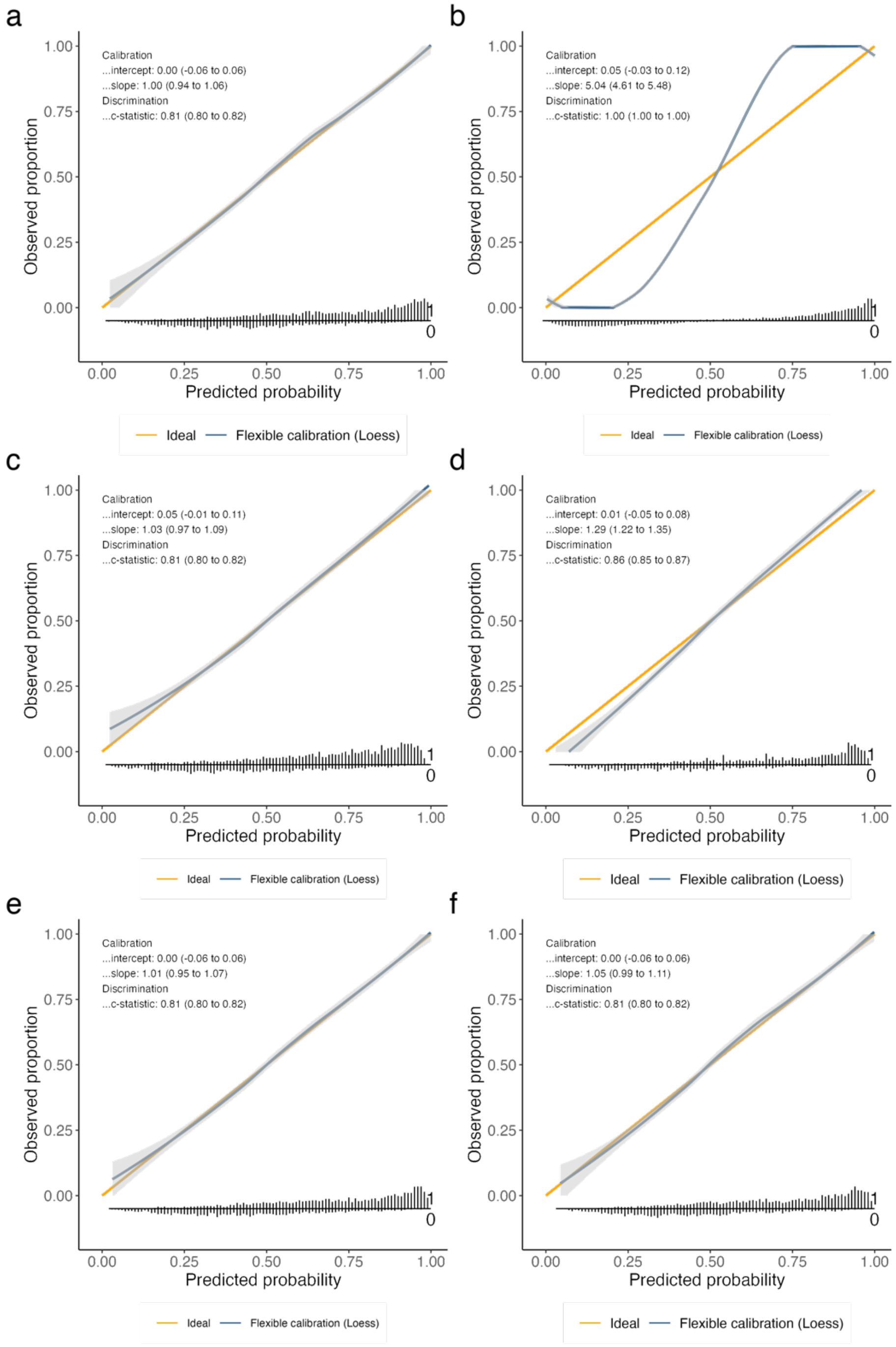

Figure S2.6.1. Internal validation on the IST training dataset: calibration plot of predicted outcome in the treatment group. The orange line indicates ideal calibration. The outcome variable is death or dependency at 6 months. **a**, T-learner Logistic Regression. **b**, T-learner Random Forest. **c**, T-learner Support Vector Machine. **d**, T-learner XGBoost. **e**, T-learner Penalized Logistic Regression. **f**, S-learner Logistic Regression. Abbreviations: IST: the International Stroke Trial.

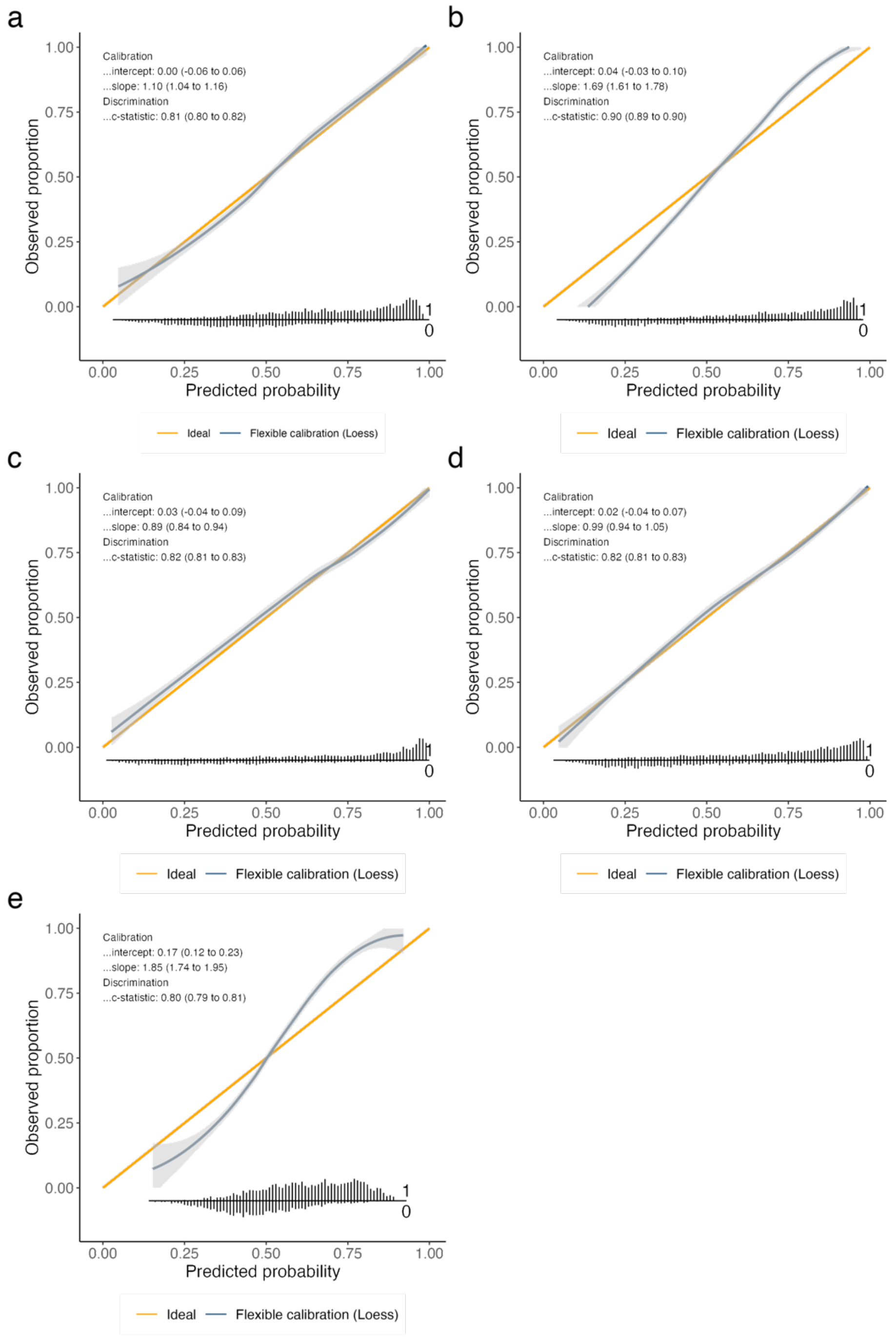

Figure S2.6.2. Internal validation on the IST training dataset: calibration plot of predicted outcome in the treatment group. The orange line indicates ideal calibration. The outcome variable is death or dependency at 6 months. **a**, S-learner Penalized Logistic Regression. **b**, S-learner XGBoost **c**, S-learner BART. **d**, CVAE. **e**, GANITE. Abbreviations: IST: the International Stroke Trial.

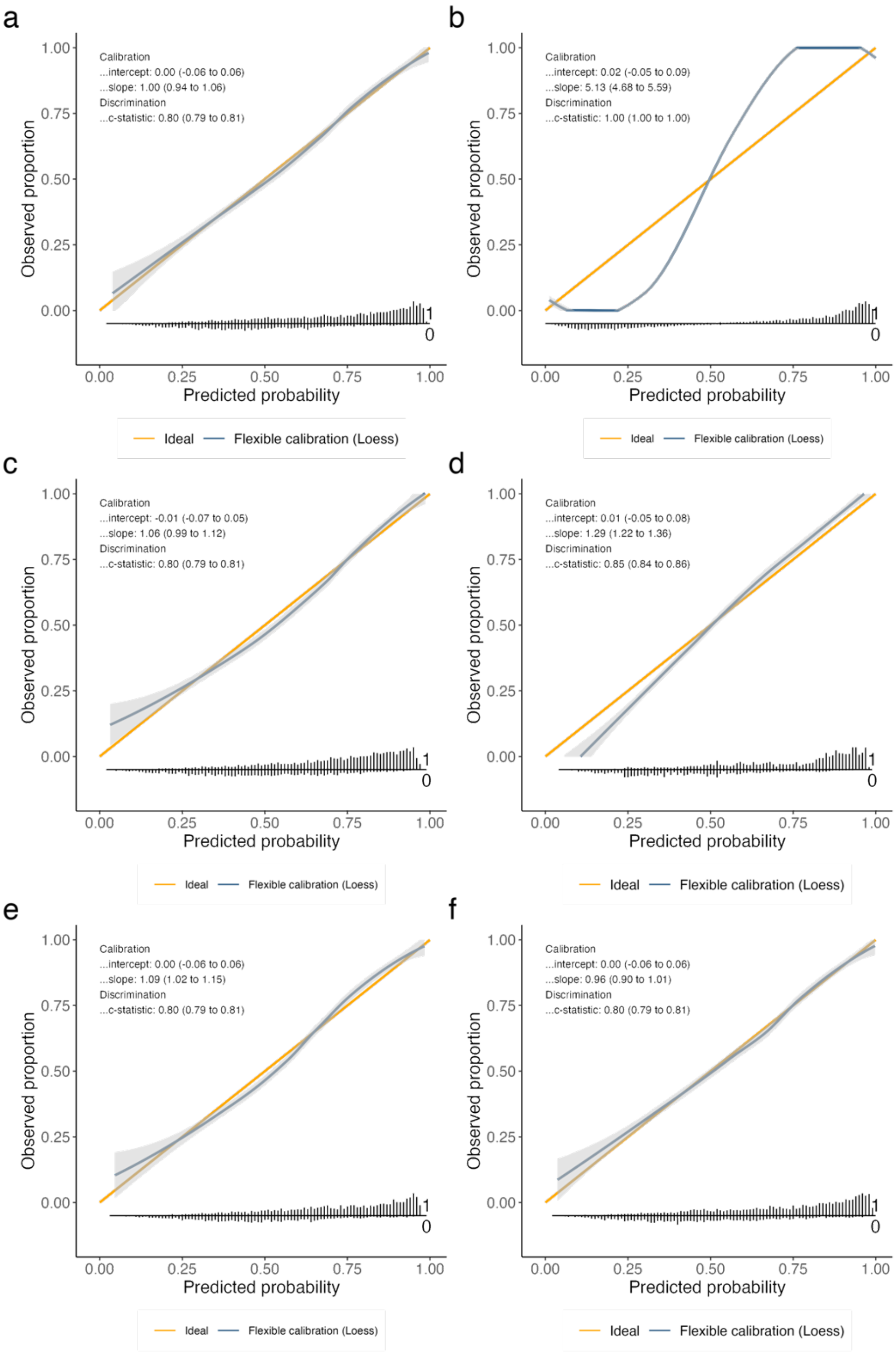

Figure S2.7.1. Internal validation on the IST training dataset: calibration plot of predicted outcome in the control group. The orange line indicates ideal calibration. The outcome variable is death or dependency at 6 months. **a**, T-learner Logistic Regression. **b**, T-learner Random Forest. **c**, T-learner Support Vector Machine. **d**, T-learner XGBoost. **e**, T-learner Penalized Logistic Regression. **f**, S-learner Logistic Regression. Abbreviations: IST: the International Stroke Trial.

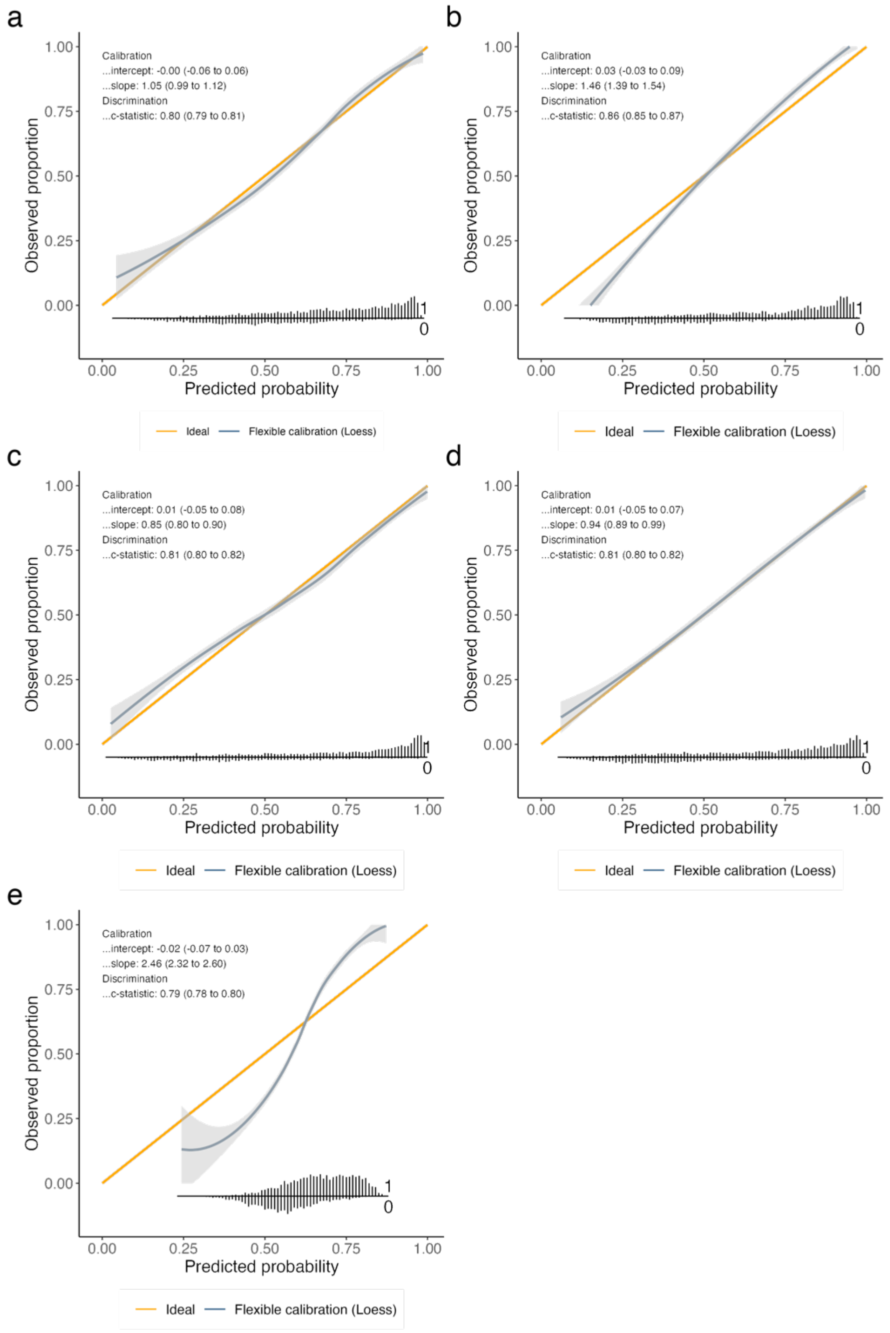

Figure S2.7.2. Internal validation on the IST training dataset: calibration plot of predicted outcome in the control group. The orange line indicates ideal calibration. The outcome variable is death or dependency at 6 months. **a**, S-learner Penalized Logistic Regression. **b**, S-learner XGBoost **c**, S-learner BART. **d**, CVAE. **e**, GANITE. Abbreviations: IST: the International Stroke Trial.

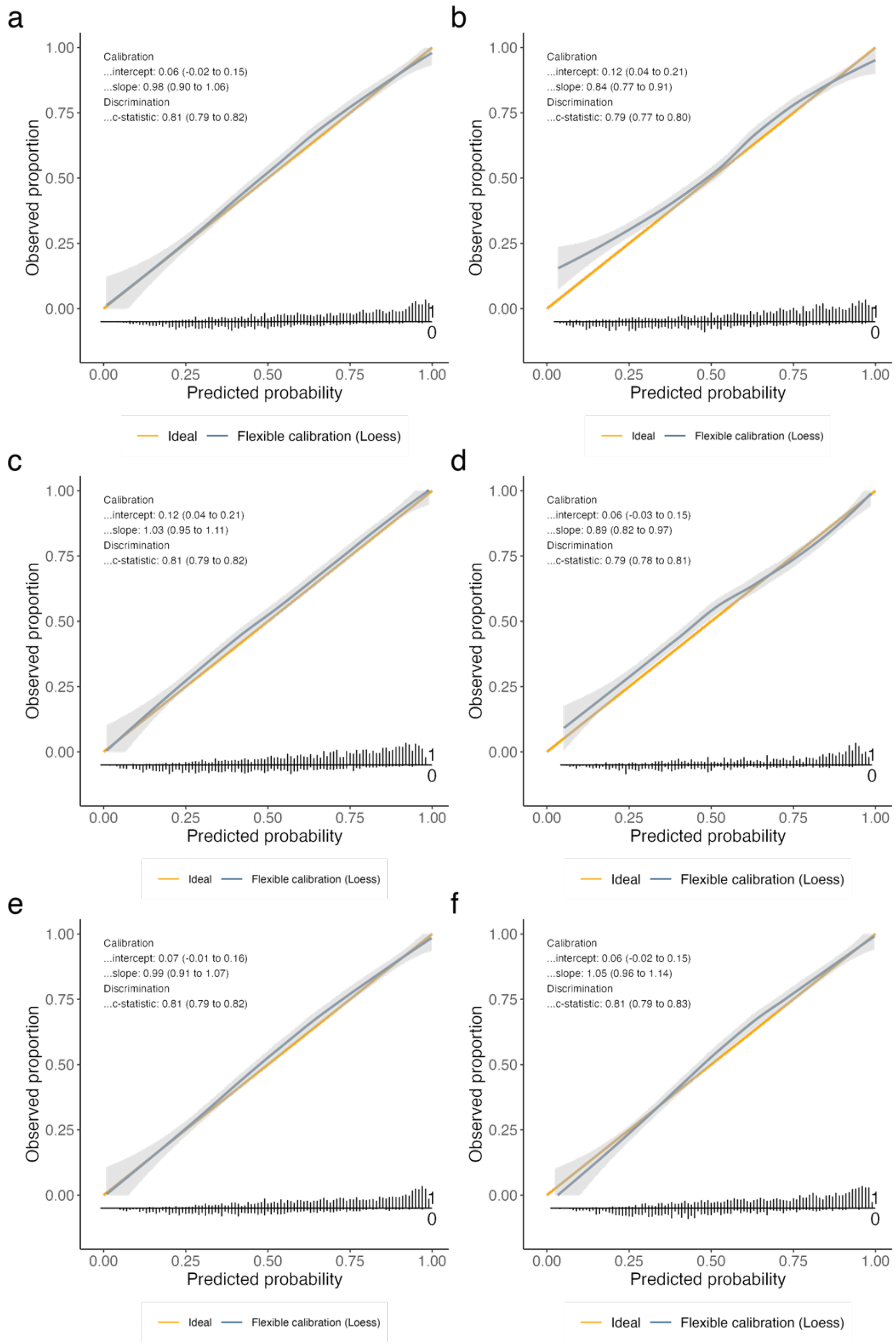

Figure S2.8.1. Internal validation on the IST test dataset: calibration plot of predicted outcome in the treatment group. The orange line indicates ideal calibration. The outcome variable is death or dependency at 6 months. **a**, T-learner Logistic Regression. **b**, T-learner Random Forest. **c**, T-learner Support Vector Machine. **d**, T-learner XGBoost. **e**, T-learner Penalized Logistic Regression. **f**, S-learner Logistic Regression. Abbreviations: IST: the International Stroke Trial.

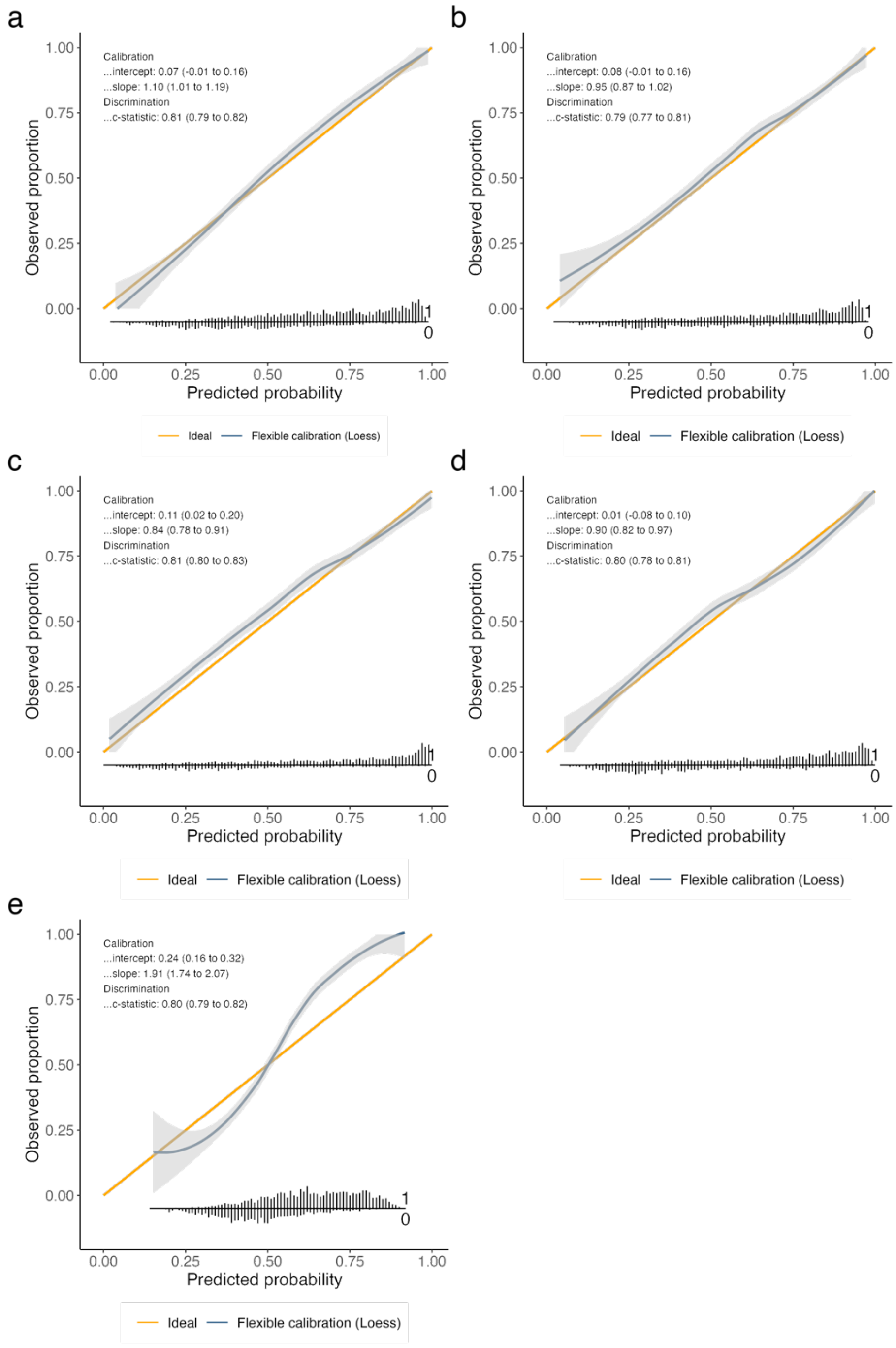

Figure S2.8.2. Internal validation on the IST test dataset: calibration plot of predicted outcome in the treatment group. The orange line indicates ideal calibration. The outcome variable is death or dependency at 6 months. **a**, S-learner Penalized Logistic Regression. **b**, S-learner XGBoost **c**, S-learner BART. **d**, CVAE. **e**, GANITE. Abbreviations: IST: the International Stroke Trial.

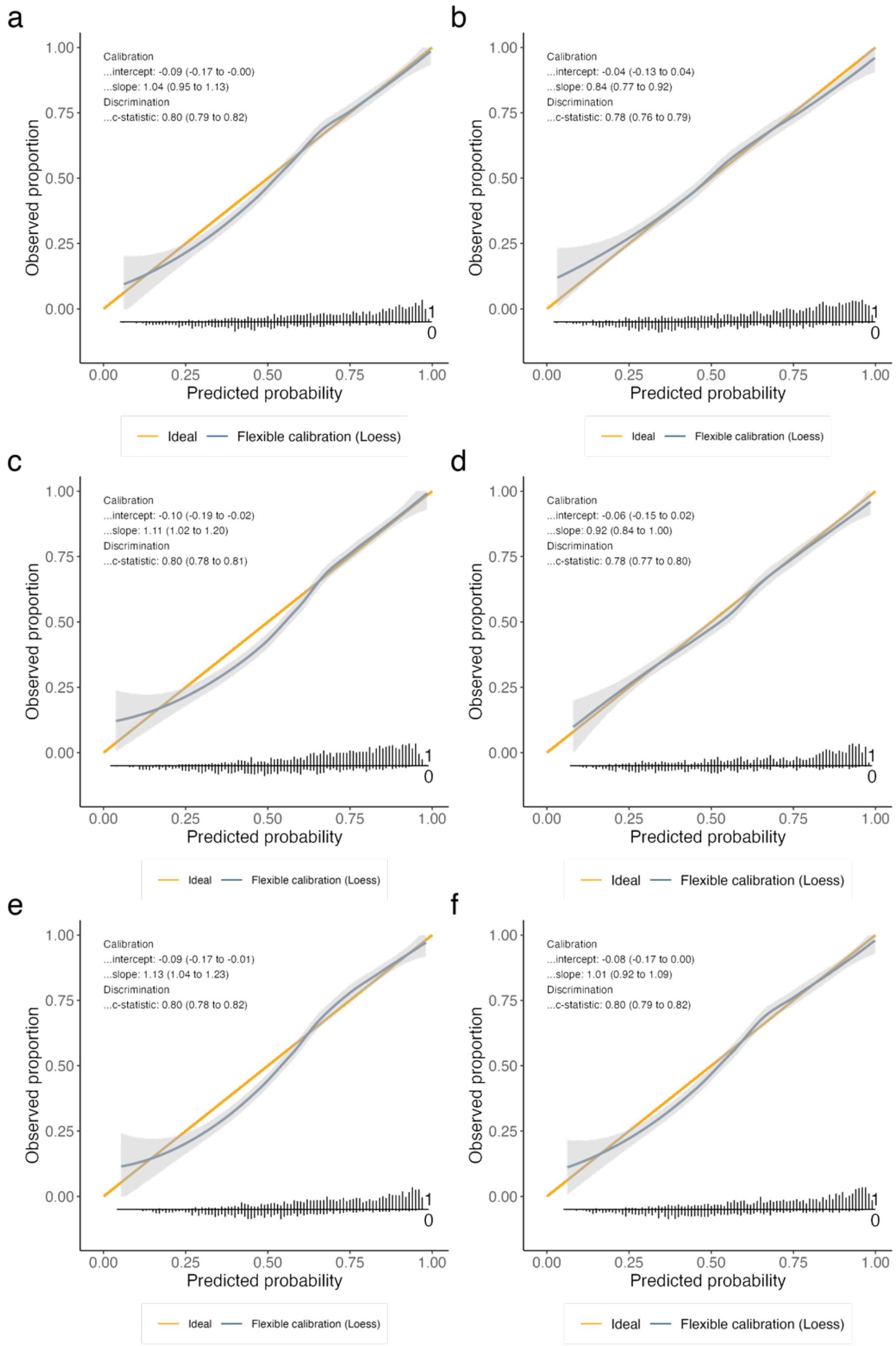

Figure S2.9.1. Internal validation on the IST test dataset: calibration plot of predicted outcome in the control group. The orange line indicates ideal calibration. The outcome variable is death or dependency at 6 months. **a**, T-learner Logistic Regression. **b**, T-learner Random Forest. **c**, T-learner Support Vector Machine. **d**, T-learner XGBoost. **e**, T-learner Penalized Logistic Regression. **f**, S-learner Logistic Regression. Abbreviations: IST: the International Stroke Trial.

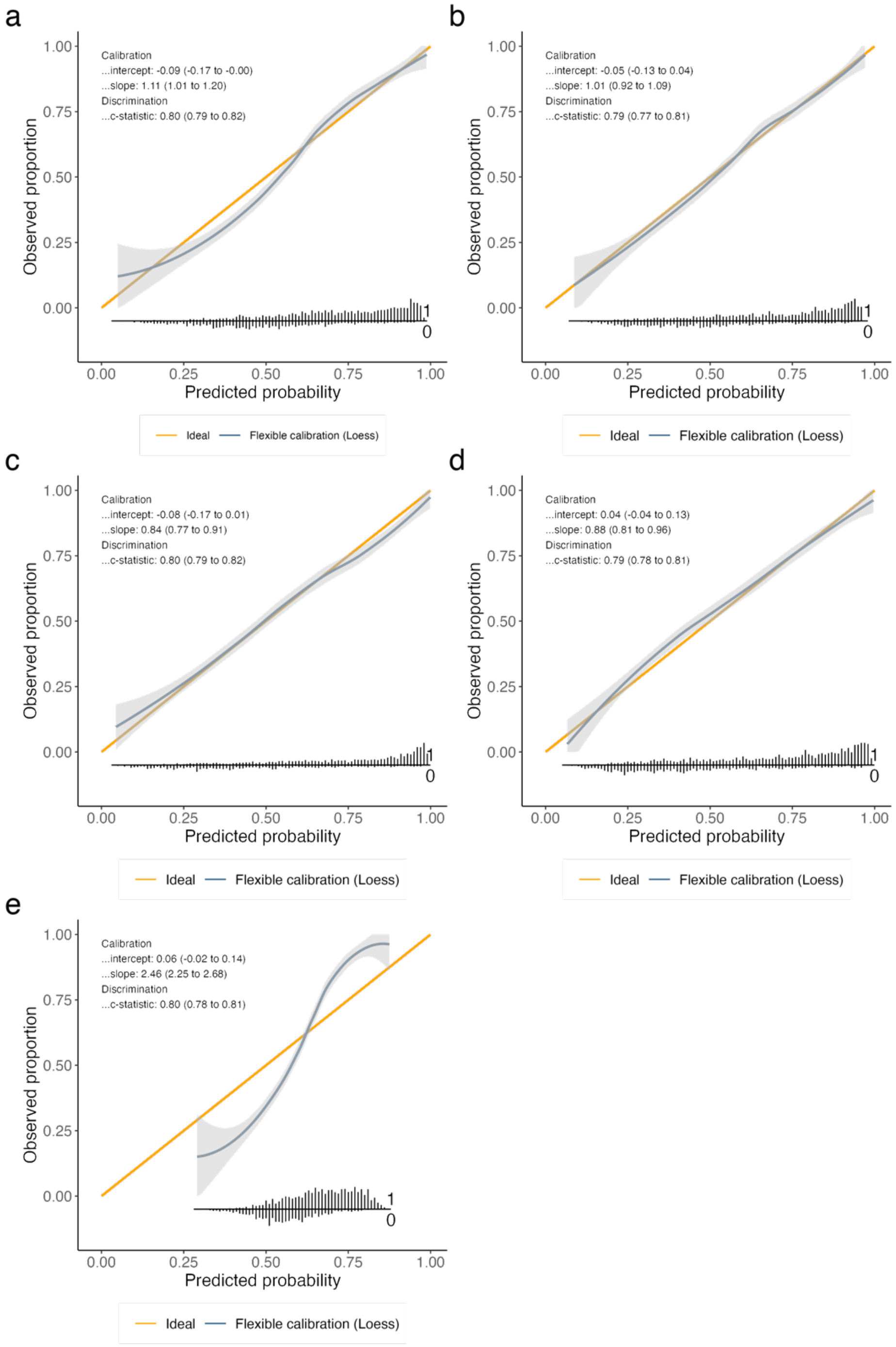

Figure S2.9.2. Internal validation on the IST test dataset: calibration plot of predicted outcome in the control group. The orange line indicates ideal calibration. The outcome variable is death or dependency at 6 months. **a**, S-learner Penalized Logistic Regression. **b**, S-learner XGBoost **c**, S-learner BART. **d**, CVAE. **e**, GANITE. Abbreviations: IST: the International Stroke Trial.

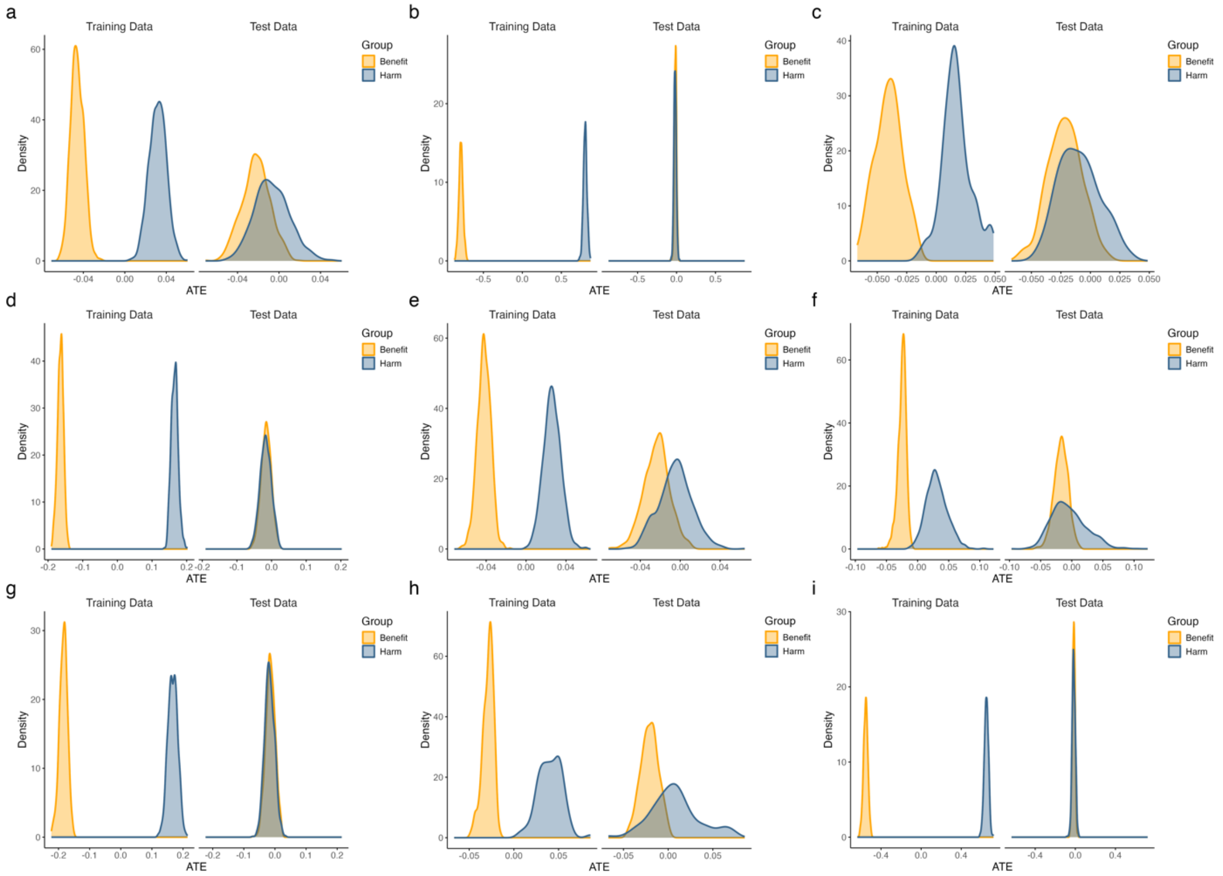

Figure S2.10.1. Internal validation on the IST dataset: density comparative analysis of causal machine learning-based individualized treatment effects. Density plots depict the distributions of ATE in training data and test data stratified by benefit (orange) and harm (blue) groups. The distribution of ATE comes from 1000 (**a, b, d, e, f, g, i**) or 100 (**c, h**) random train-test splits experiments. Benefit means negative ITE values and harm means positive ITE values. The outcome variable is death or dependency at 6 months. **a**, T-learner Logistic Regression. **b**, T-learner Random Forest. **c**, T-learner Support Vector Machine. **d**, T-learner XGBoost. **e**, T-learner Penalized Logistic Regression. **f**, S-learner Penalized Logistic Regression. **g**, S-learner XGBoost. **h**, S-learner BART. **i**, X-learner Random Forest. Abbreviations: IST: the International Stroke Trial, ITE: individualized treatment effect, ATE: average treatment effect.

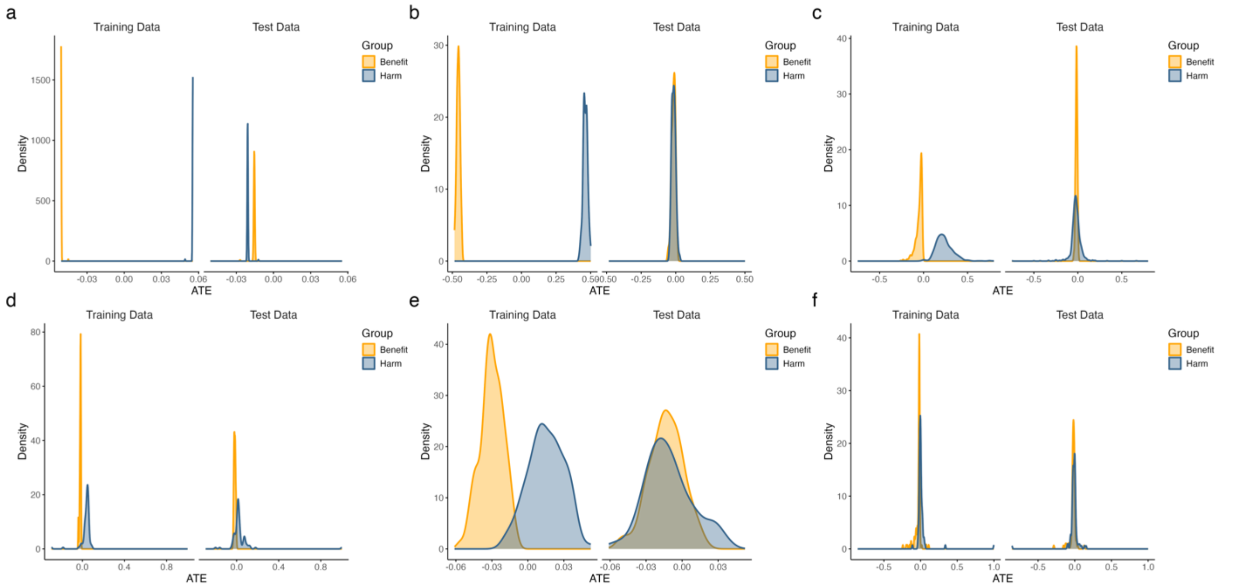

Figure S2.10.2. Internal validation on the IST dataset: density comparative analysis of causal machine learning-based individualized treatment effects. Density plots depict the distributions of ATE in training data and test data stratified by benefit (orange) and harm (blue) groups. The distribution of ATE comes from 1000 (**b**) or 100 (**a, c, d, e, f**) random train-test splits experiments. Benefit means negative ITE values and harm means positive ITE values. The outcome variable is death or dependency at 6 months. **a**, X-learner BART. **b**, DR-learner Random Forest. **c**, Causal Forest. **d**, Bayesian Causal Forest. **e**, CVAE. **f**, GANITE. Abbreviations: IST: the International Stroke Trial, ITE: individualized treatment effect, ATE: average treatment effect.

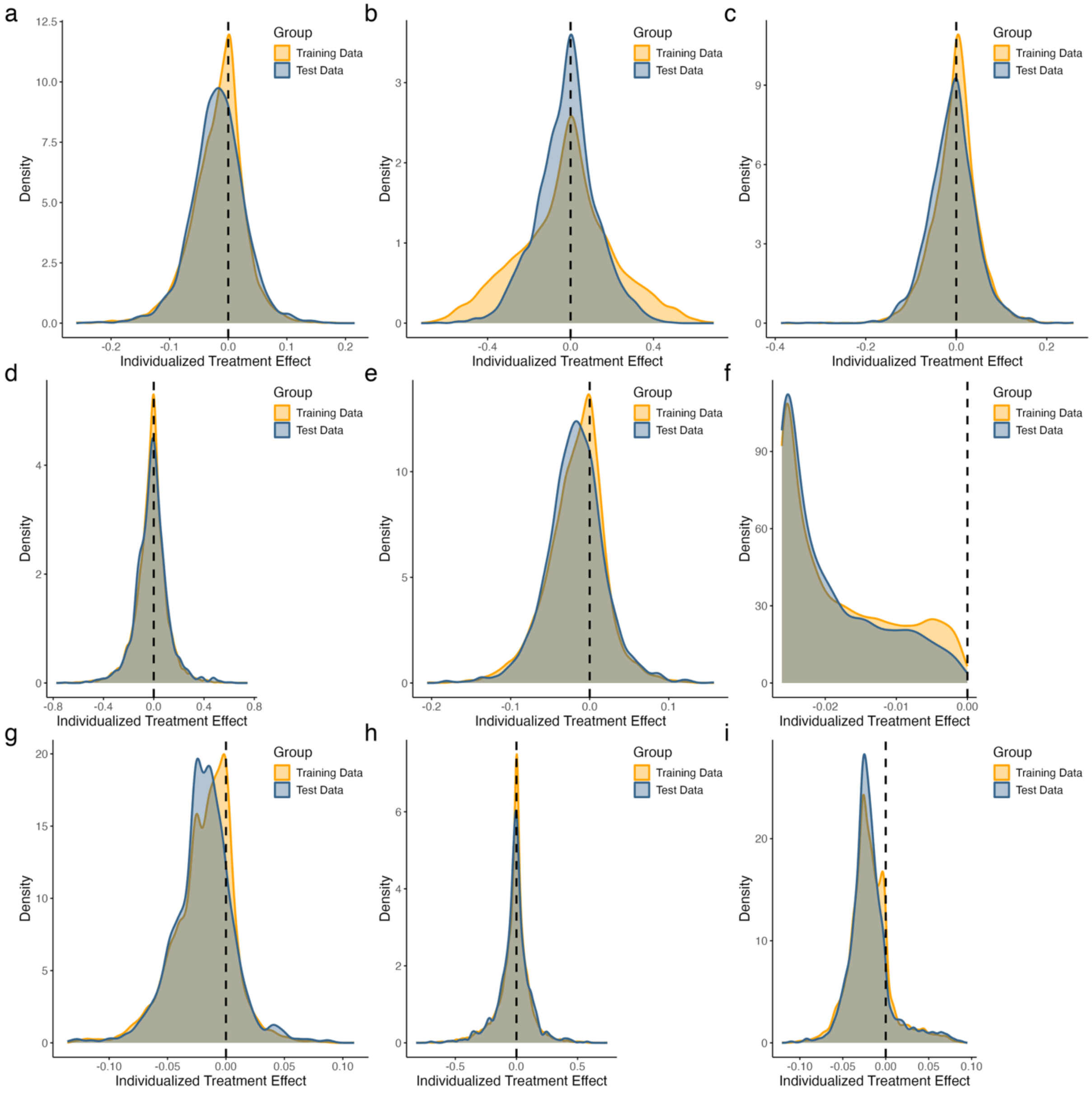

Figure S2.11.1. Internal validation on the IST dataset based on geographical split: density plots of causal machine learning-based individualized treatment effects. Orange represents the training data, and blue indicates the test data. The outcome variable is death or dependency at 6 months. **a**, T-learner Logistic Regression. **b**, T-learner Random Forest. **c**, T-learner Support Vector Machine. **d**, T-learner XGBoost. **e**, T-learner Penalized Logistic Regression. **f**, S-learner Logistic Regression. **g**, S-learner Penalized Logistic Regression. **h**, S-learner XGBoost. **i**, S-learner BART. Abbreviations: IST: the International Stroke Trial, ITE: individualized treatment effect.

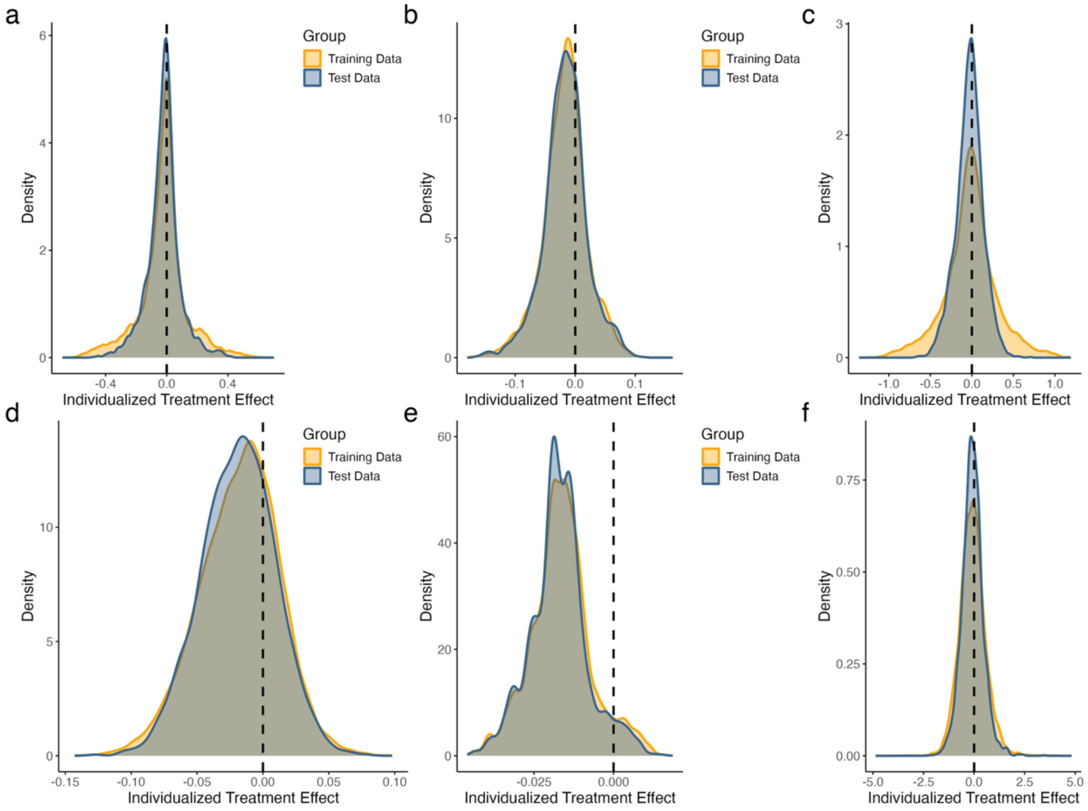

Figure S2.11.2. Internal validation on the IST dataset based on geographical split: density plots of causal machine learning-based individualized treatment effects. Orange represents the training data, and blue indicates the test data. The outcome variable is death or dependency at 6 months. **a**, X-learner Random Forest. **b**, X-learner BART. **c**, DR-learner Random Forest. **d**, Causal Forest. **e**, Bayesian Causal Forest. **f**, Model-based Recursive Partitioning. Abbreviations: IST: the International Stroke Trial, ITE: individualized treatment effect.

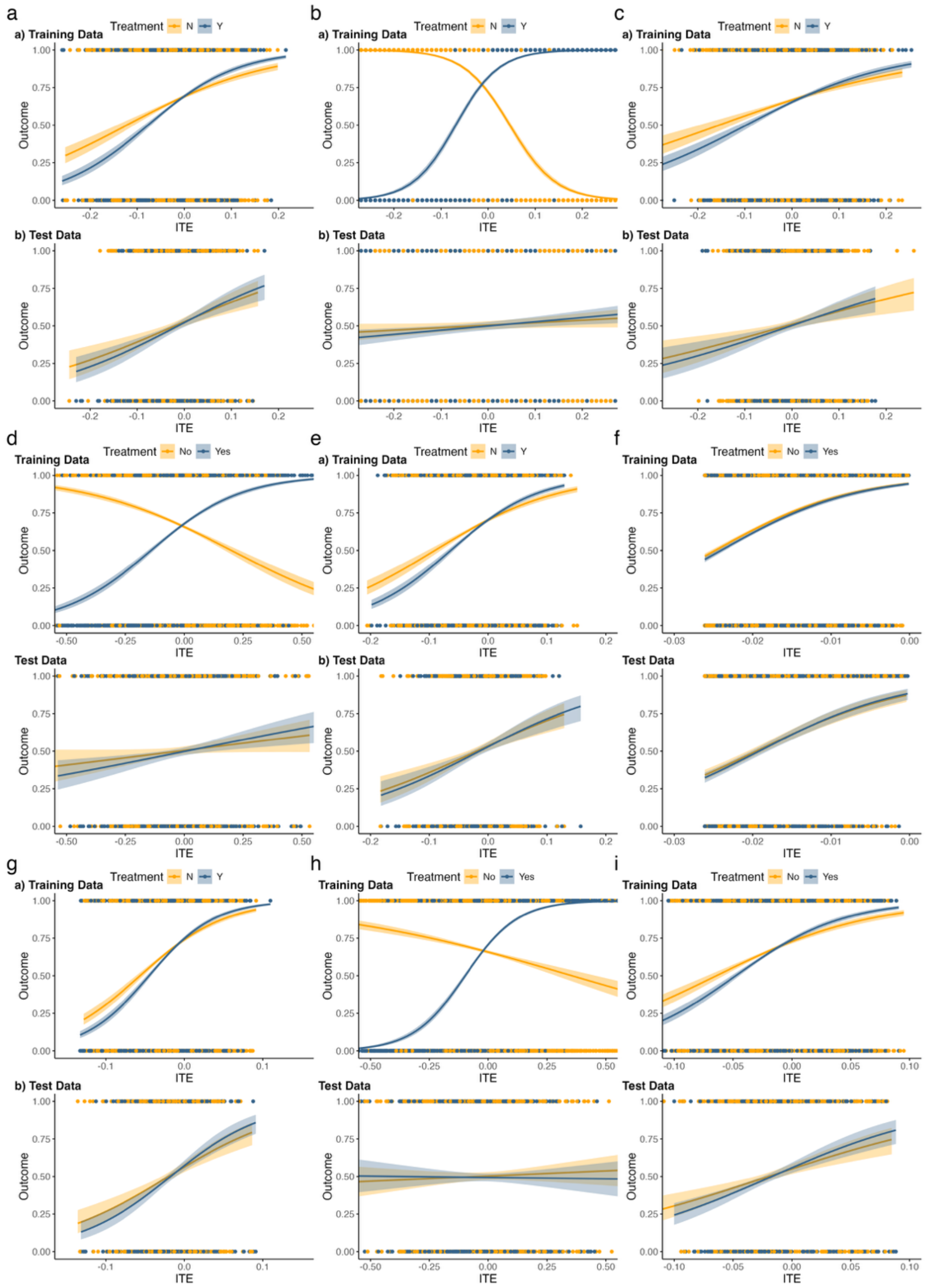

Figure S2.12.1. Internal validation on the IST dataset based on geographical split: outcome-ITE comparative analysis. Dot plots and line plots depict the true and fitted patient outcomes against estimated ITE values between training data and test data. Orange represents the control group, and blue indicates the treatment group. The outcome variable is death or dependency at 6 months. **a**, T-learner Logistic Regression. **b**, T-learner Random Forest. **c**, T-learner Support Vector Machine. **d**, T-learner XGBoost. **e**, T-learner Penalized Logistic Regression. **f**, S-learner Logistic Regression. **g**, S-learner Penalized Logistic Regression. **h**, S-learner XGBoost. **i**, S-learner BART. Abbreviations: IST: the International Stroke Trial, ITE: individualized treatment effect.

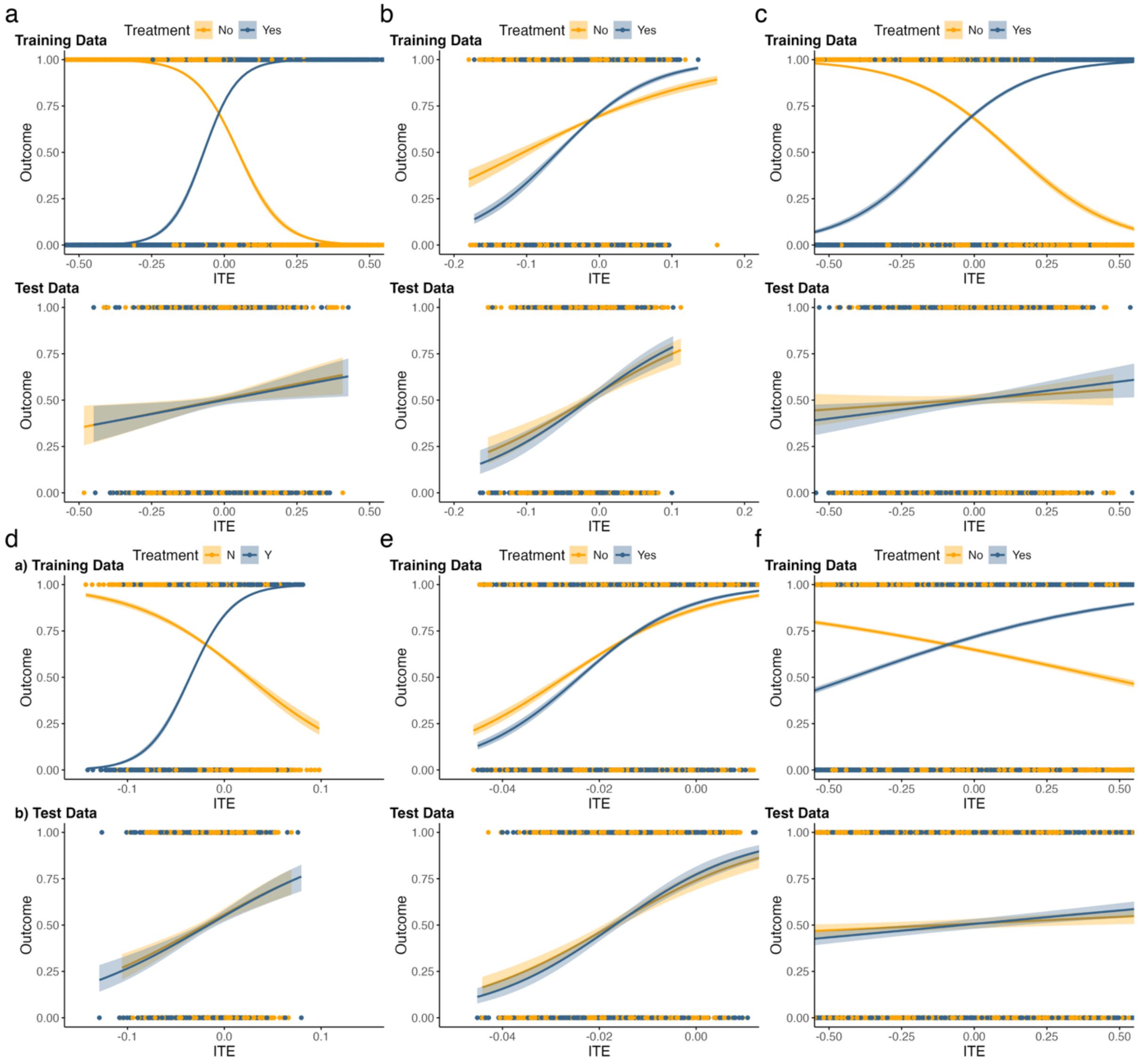

Figure S2.12.2. Internal validation on the IST dataset based on geographical split: outcome-ITE comparative analysis. Dot plots and line plots depict the true and fitted patient outcomes against estimated ITE values between training data and test data. Orange represents the control group, and blue indicates the treatment group. The outcome variable is death or dependency at 6 months. **a**, X-learner Random Forest. **b**, X-learner BART. **c**, DR-learner Random Forest. **d**, Causal Forest. **e**, Bayesian Causal Forest. **f**, Model-based Recursive Partitioning. Abbreviations: IST: the International Stroke Trial, ITE: individualized treatment effect.

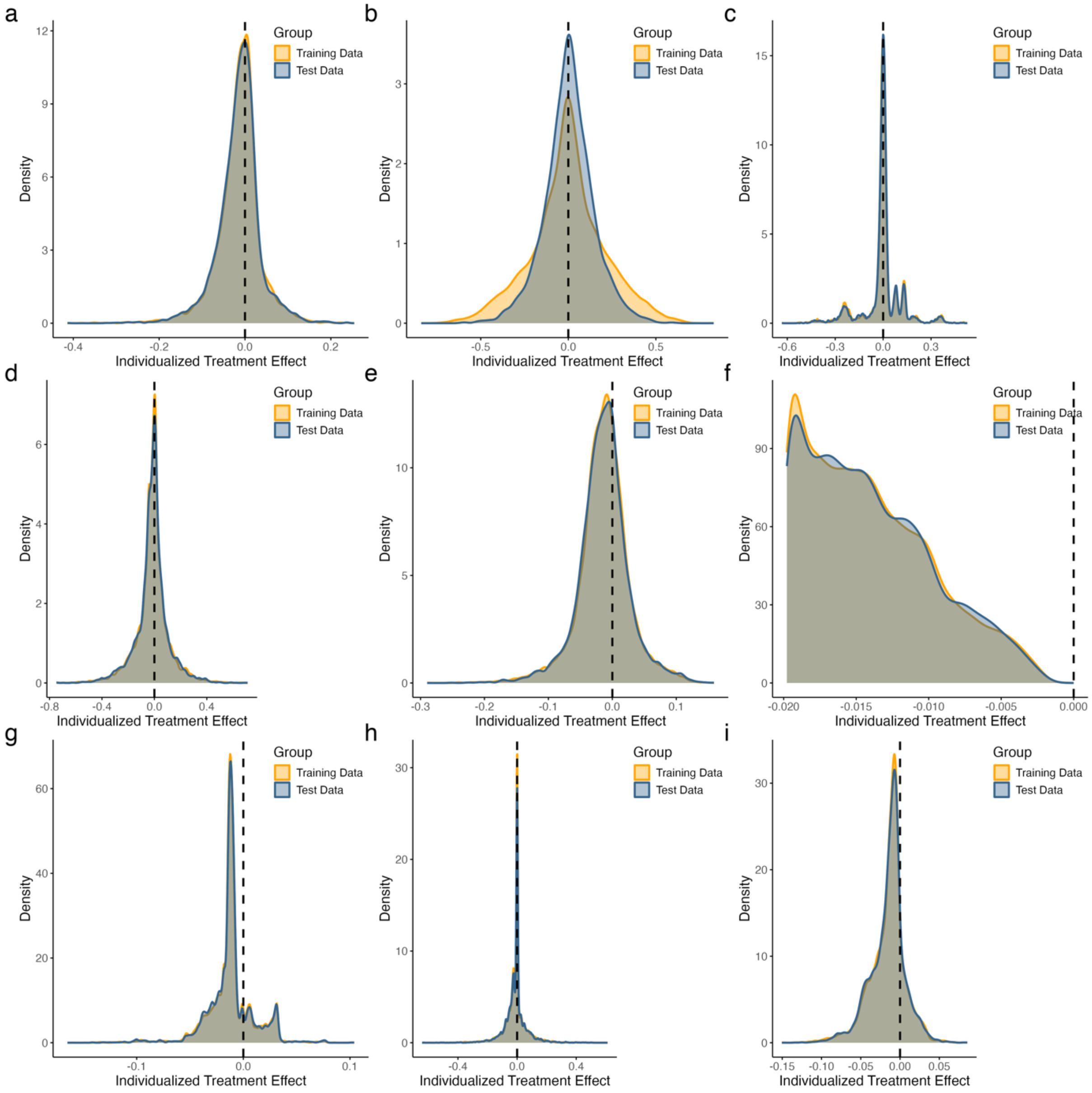

Figure S3.1.1. Internal validation on the CAST dataset: density plots of causal machine learning-based individualized treatment effects. Orange represents the training data, and blue indicates the test data. The outcome variable is death or dependency at 4 weeks. **a**, T-learner Logistic Regression. **b**, T-learner Random Forest. **c**, T-learner Support Vector Machine. **d**, T-learner XGBoost. **e**, T-learner Penalized Logistic Regression. **f**, S-learner Logistic Regression. **g**, S-learner Penalized Logistic Regression. **h**, S-learner XGBoost. **i**, S-learner BART. Abbreviations: CAST: the Chinese Acute Stroke Trial, ITE: individualized treatment effect.

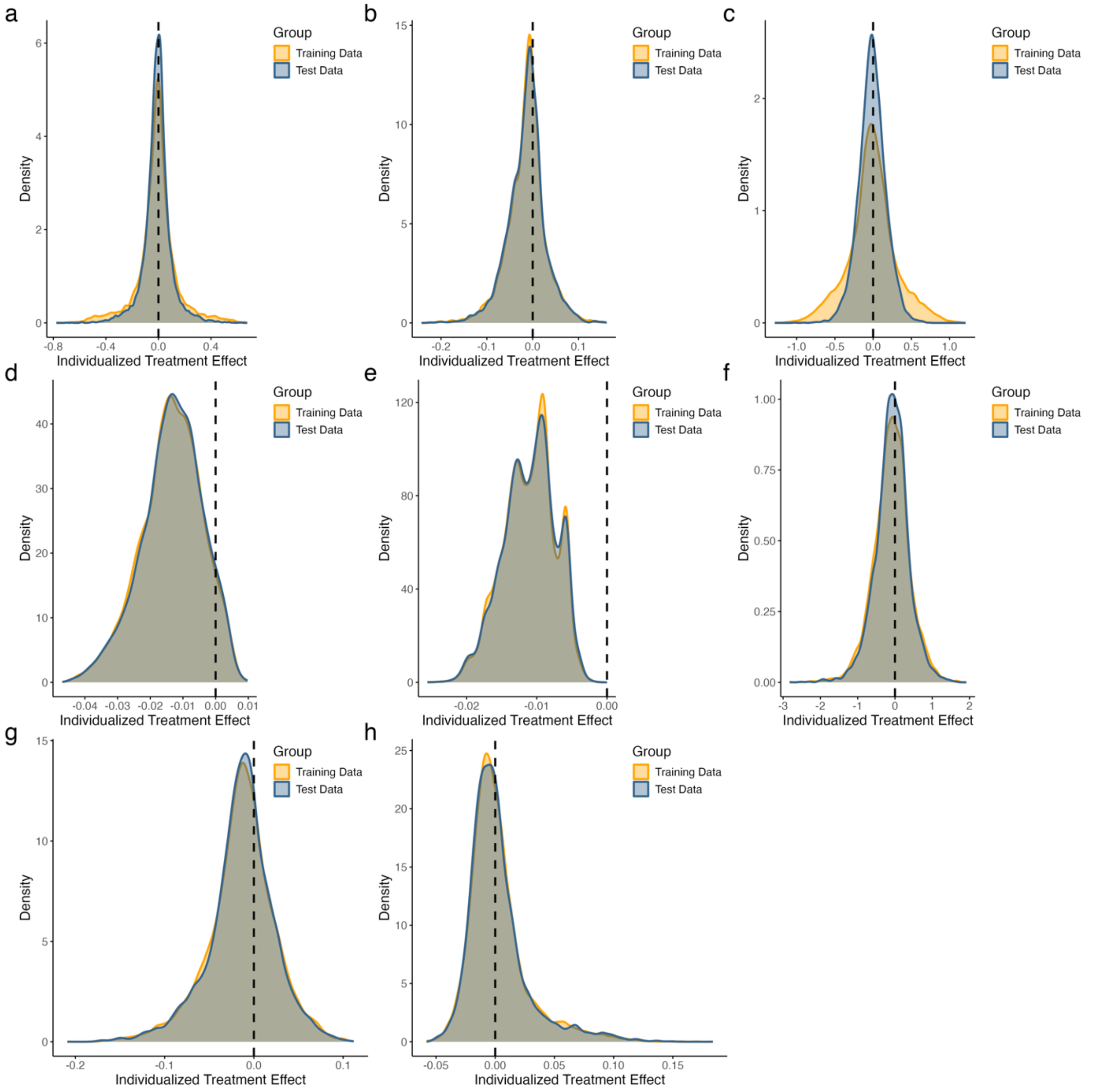

Figure S3.1.2. Internal validation on the CAST dataset: density plots of causal machine learning-based individualized treatment effects. Orange represents the training data, and blue indicates the test data. The outcome variable is death or dependency at 4 weeks. **a**, X-learner Random Forest. **b**, X-learner BART. **c**, DR-learner Random Forest. **d**, Causal Forest. **e**, Bayesian Causal Forest. **f**, Model-based Recursive Partitioning. **g**, CVAE. **h**, GANITE. Abbreviations: CAST: the Chinese Acute Stroke Trial, ITE: individualized treatment effect.

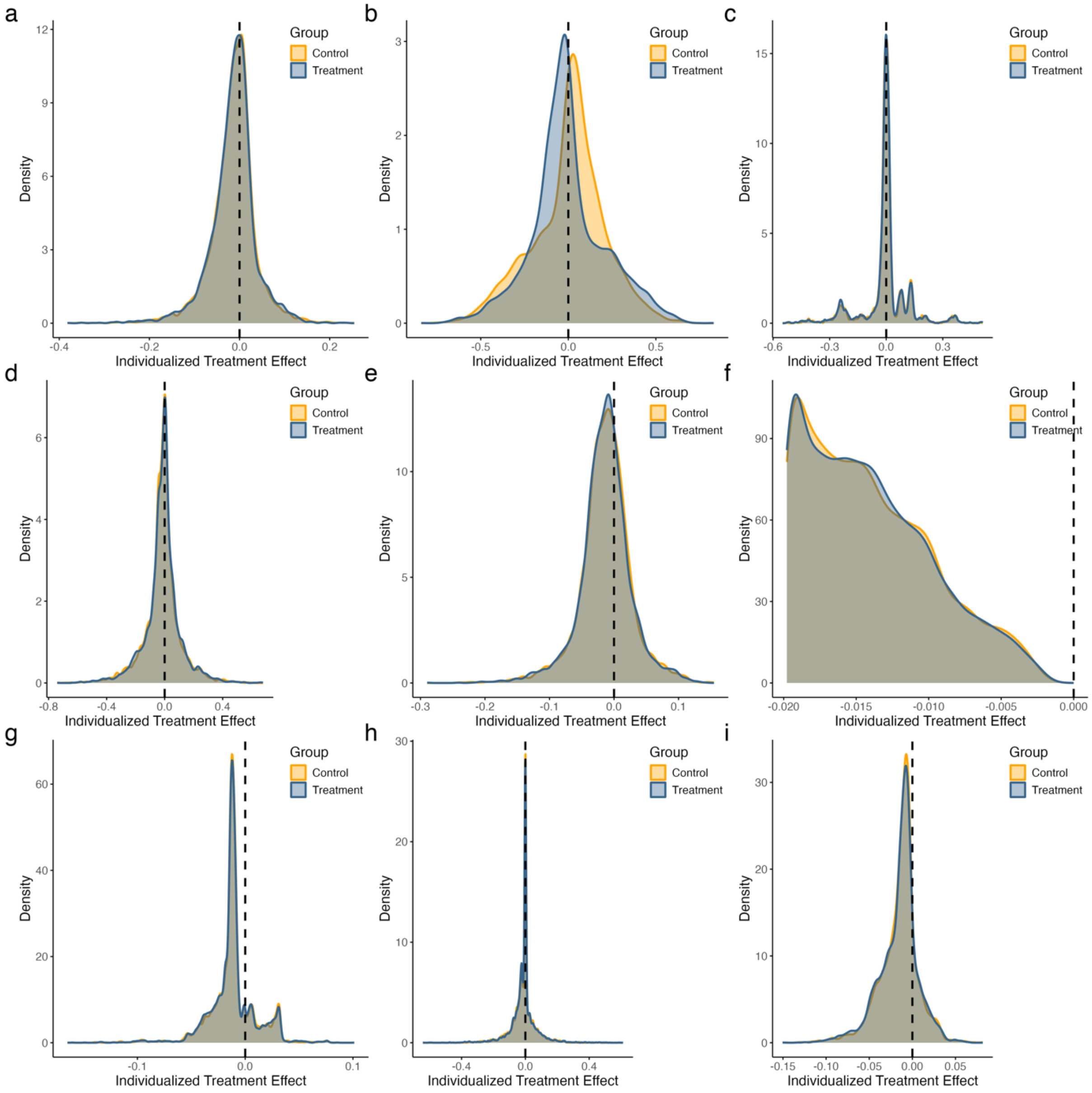

Figure S3.2.1. Internal validation on the CAST training dataset: density plots of causal machine learning-based individualized treatment effects. Orange represents the control group, and blue indicates the treatment group. The outcome variable is death or dependency at 4 weeks. **a**, T-learner Logistic Regression. **b**, T-learner Random Forest. **c**, T-learner Support Vector Machine. **d**, T-learner XGBoost. **e**, T-learner Penalized Logistic Regression. **f**, S-learner Logistic Regression. **g**, S-learner Penalized Logistic Regression. **h**, S-learner XGBoost. **i**, S-learner BART. Abbreviations: CAST: the Chinese Acute Stroke Trial, ITE: individualized treatment effect.

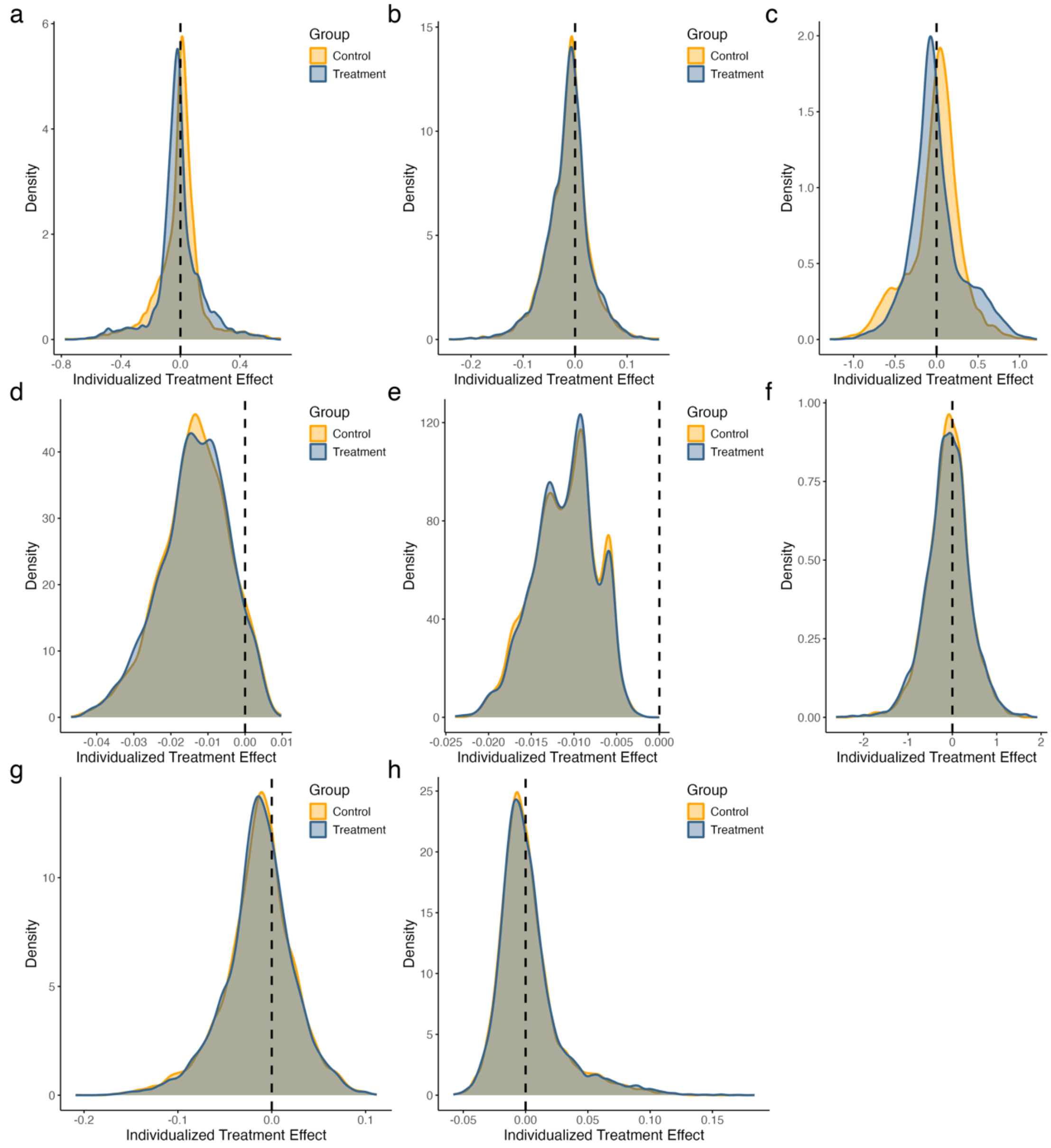

Figure S3.2.2. Internal validation on the CAST training dataset: density plots of causal machine learning-based individualized treatment effects. Orange represents the control group, and blue indicates the treatment group. The outcome variable is death or dependency at 4 weeks. **a**, X-learner Random Forest. **b**, X-learner BART. **c**, DR-learner Random Forest. **d**, Causal Forest. **e**, Bayesian Causal Forest. **f**, Model-based Recursive Partitioning. **g**, CVAE. **h**, GANITE. Abbreviations: CAST: the Chinese Acute Stroke Trial, ITE: individualized treatment effect.

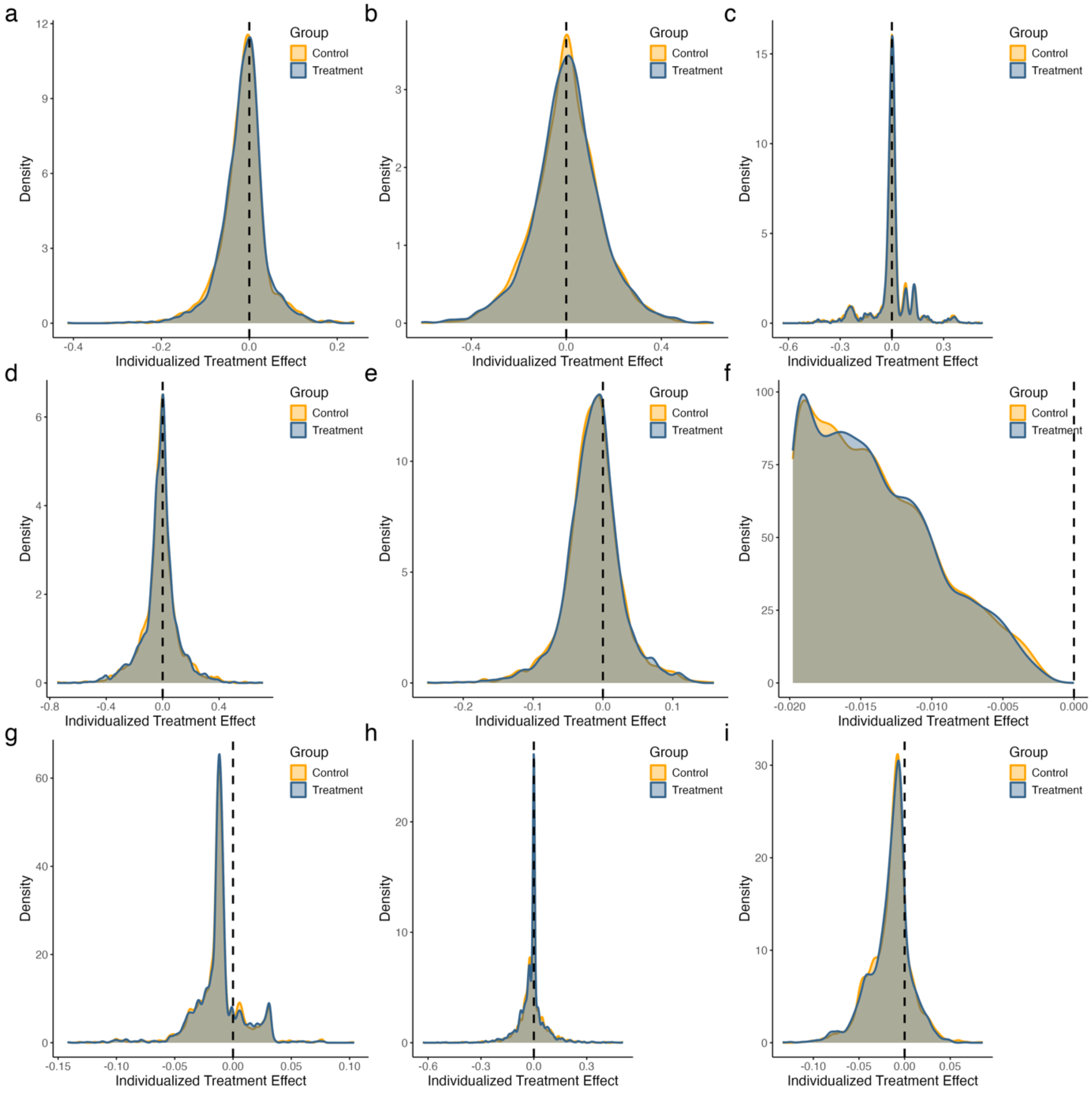

Figure S3.3.1. Internal validation on the CAST test dataset: density plots of causal machine learning-based individualized treatment effects. Orange represents the control group, and blue indicates the treatment group. The outcome variable is death or dependency at 4 weeks. **a**, T-learner Logistic Regression. **b**, T-learner Random Forest. **c**, T-learner Support Vector Machine. **d**, T-learner XGBoost. **e**, T-learner Penalized Logistic Regression. **f**, S-learner Logistic Regression. **g**, S-learner Penalized Logistic Regression. **h**, S-learner XGBoost. **i**, S-learner BART. Abbreviations: CAST: the Chinese Acute Stroke Trial, ITE: individualized treatment effect.

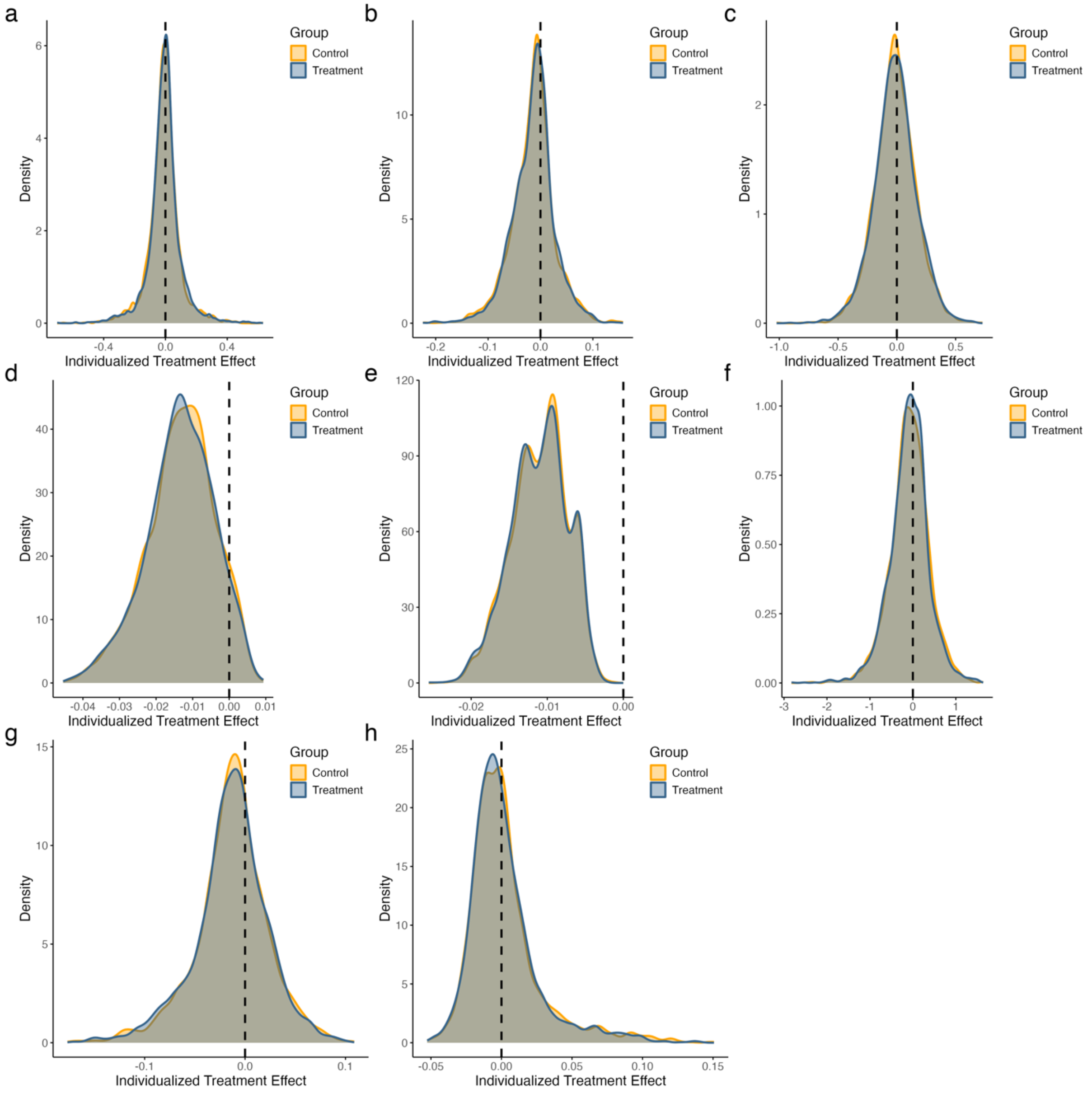

Figure S3.3.2. Internal validation on the CAST test dataset: density plots of causal machine learning-based individualized treatment effects. Orange represents the control group, and blue indicates the treatment group. The outcome variable is death or dependency at 4 weeks. **a**, X-learner Random Forest. **b**, X-learner BART. **c**, DR-learner Random Forest. **d**, Causal Forest. **e**, Bayesian Causal Forest. **f**, Model-based Recursive Partitioning. **g**, CVAE. **h**, GANITE. Abbreviations: CAST: the Chinese Acute Stroke Trial, ITE: individualized treatment effect.

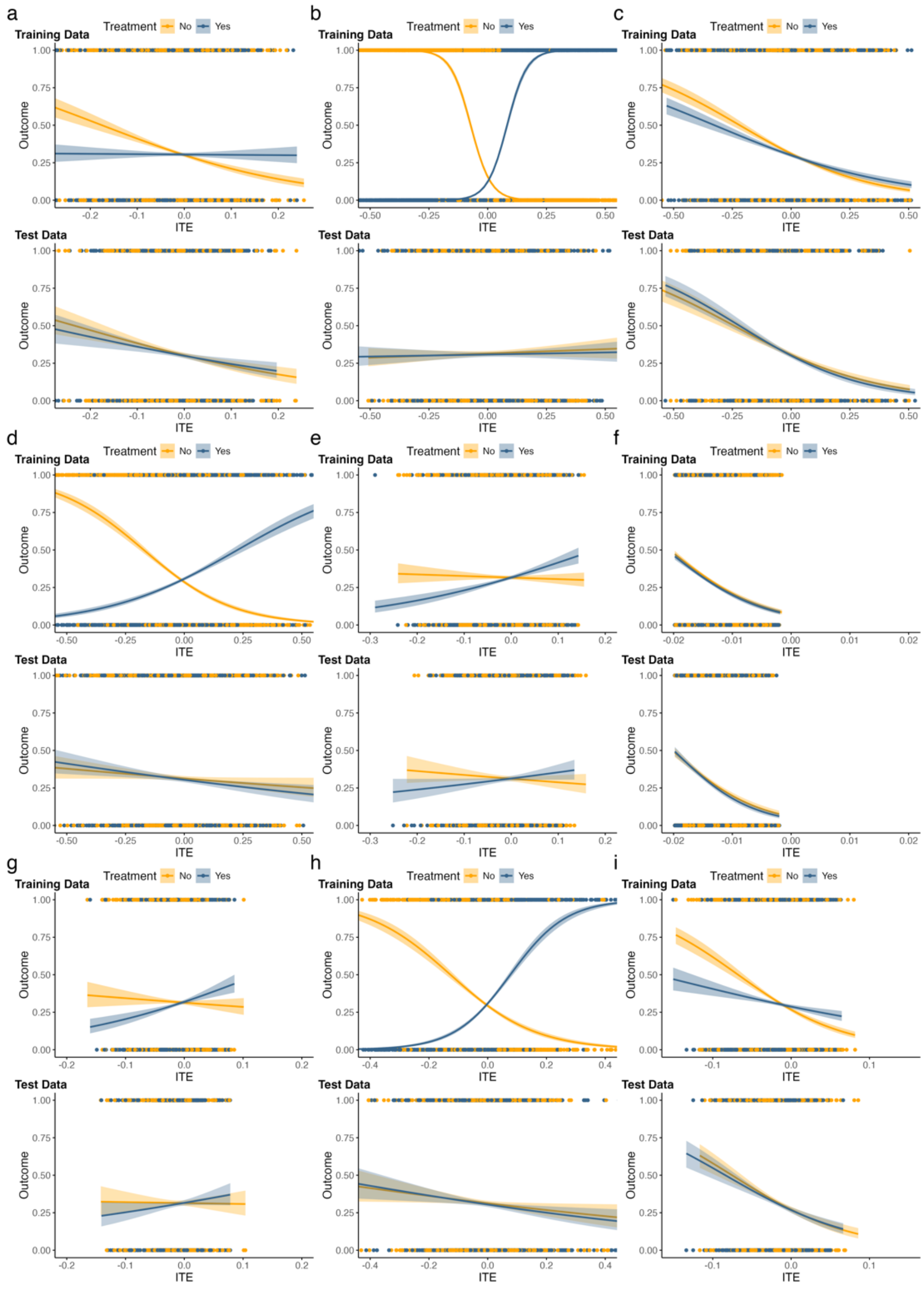

Figure S3.4.1. Internal validation on the CAST dataset: outcome-ITE comparative analysis. Dot plots and line plots depict the true and fitted patient outcomes against estimated ITE values between training data and test data. Orange represents the control group, and blue indicates the treatment group. The outcome variable is death or dependency at 4 weeks. **a**, T-learner Logistic Regression. **b**, T-learner Random Forest. **c**, T-learner Support Vector Machine. **d**, T-learner XGBoost. **e**, T-learner Penalized Logistic Regression. **f**, S-learner Logistic Regression. **g**, S-learner Penalized Logistic Regression. **h**, S-learner XGBoost. **i**, S-learner BART. Abbreviations: CAST: the Chinese Acute Stroke Trial, ITE: individualized treatment effect.

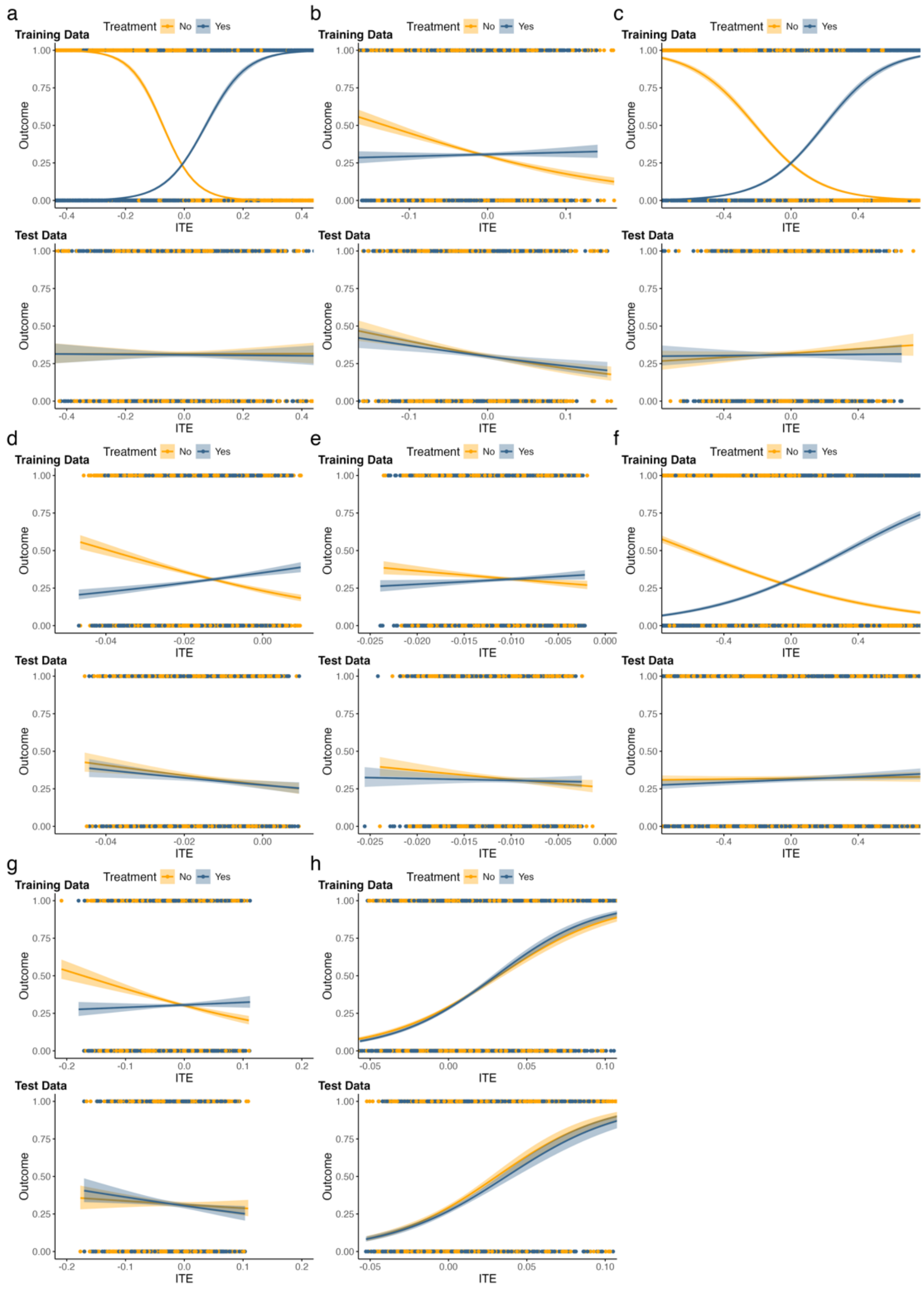

Figure S3.4.2. Internal validation on the CAST dataset: outcome-ITE comparative analysis. Dot plots and line plots depict the true and fitted patient outcomes against estimated ITE values between training data and test data. Orange represents the control group, and blue indicates the treatment group. The outcome variable is death or dependency at 4 weeks. **a**, X-learner Random Forest. **b**, X-learner BART. **c**, DR-learner Random Forest. **d**, Causal Forest. **e**, Bayesian Causal Forest. **f**, Model-based Recursive Partitioning. **g**, CVAE. **h**, GANITE. Abbreviations: CAST: the Chinese Acute Stroke Trial, ITE: individualized treatment effect.

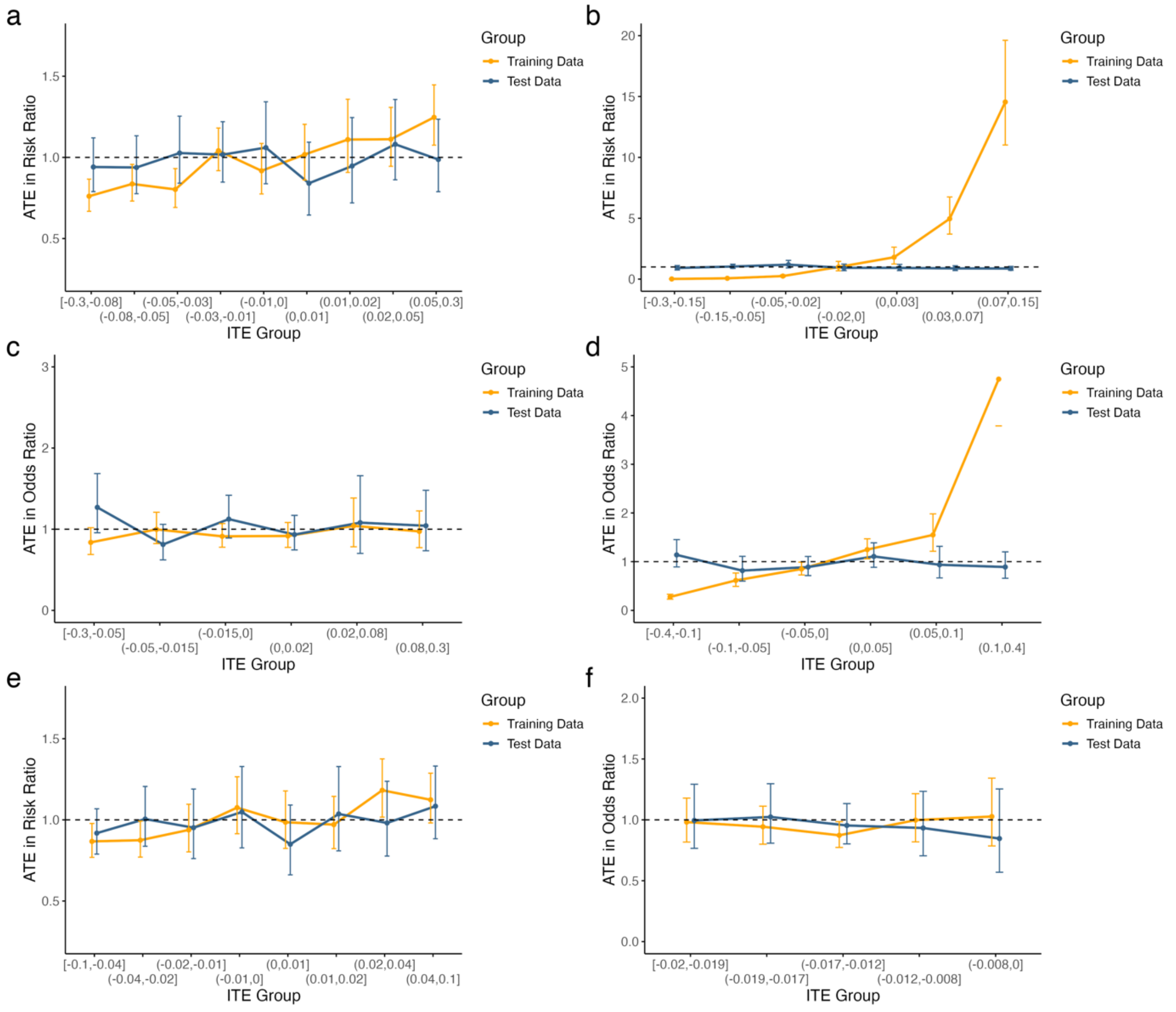

Figure S3.5.1. Internal validation on the CAST dataset: ATE-ITE comparative analysis. Line plots depict ATE in risk ratio within different ITE subgroups and provide the confidence intervals at 95% level. Orange represents the training data, and blue indicates the test data. The horizontal dashed line at 1.0 means no treatment effects. The outcome variable is death or dependency at 4 weeks. **a**, T-learner Logistic Regression. **b**, T-learner Random Forest. **c**, T-learner Support Vector Machine. **d**, T-learner XGBoost. **e**, T-learner Penalized Logistic Regression. **f**, S-learner Logistic Regression. Abbreviations: CAST: the Chinese Acute Stroke Trial, ITE: individualized treatment effect, ATE: average treatment effect.

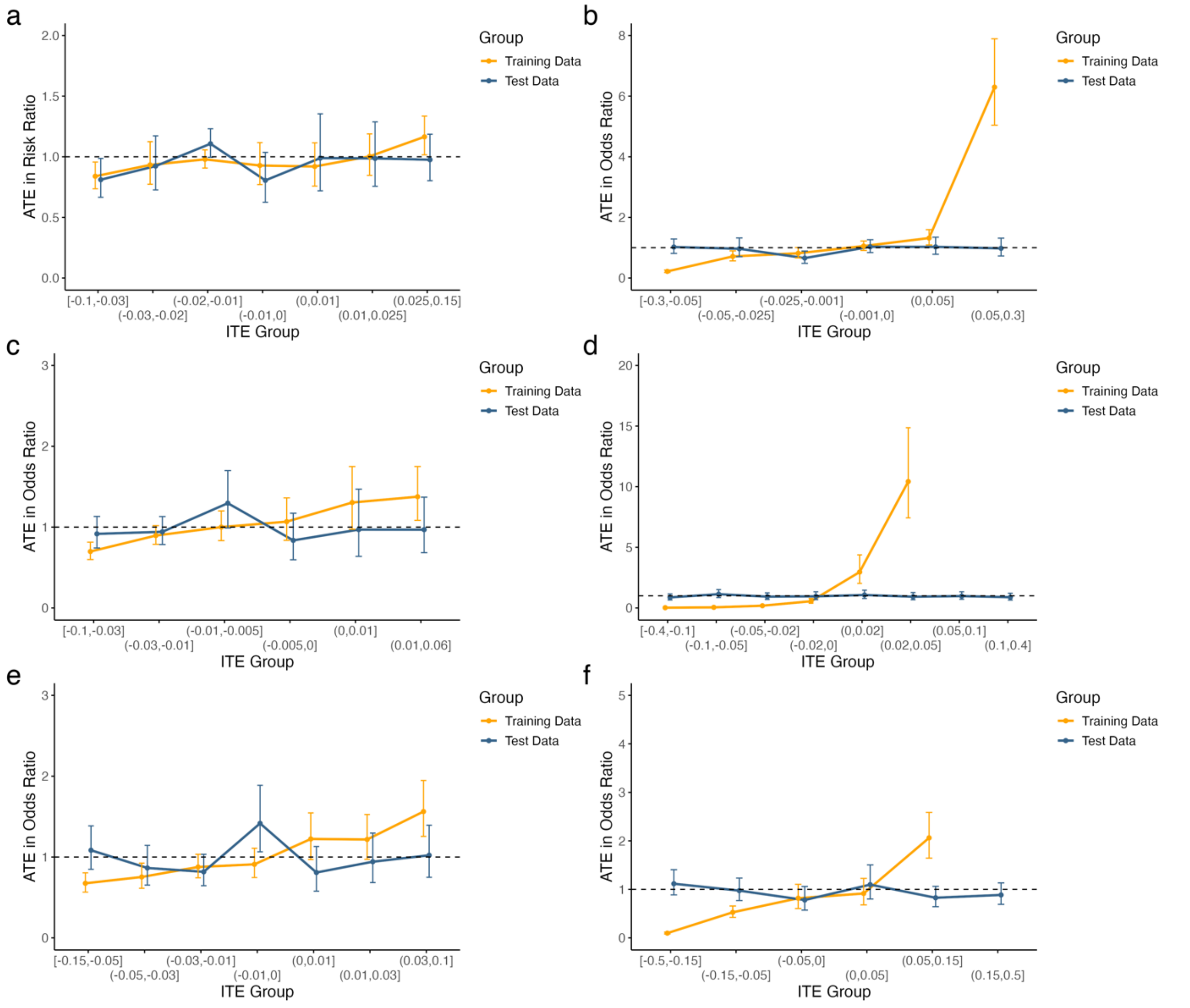

Figure S3.5.2. Internal validation on the CAST dataset: ATE-ITE comparative analysis. Line plots depict ATE in risk ratio within different ITE subgroups and provide the confidence intervals at 95% level. Orange represents the training data, and blue indicates the test data. The horizontal dashed line at 1.0 means no treatment effects. The outcome variable is death or dependency at 4 weeks. **a**, S-learner Penalized Logistic Regression. **b**, S-learner XGBoost. **c**, S-learner BART. **d**, X-learner Random Forest. **e**, X-learner BART. **f**, DR-learner Random Forest. Abbreviations: CAST: the Chinese Acute Stroke Trial, ITE: individualized treatment effect, ATE: average treatment effect.

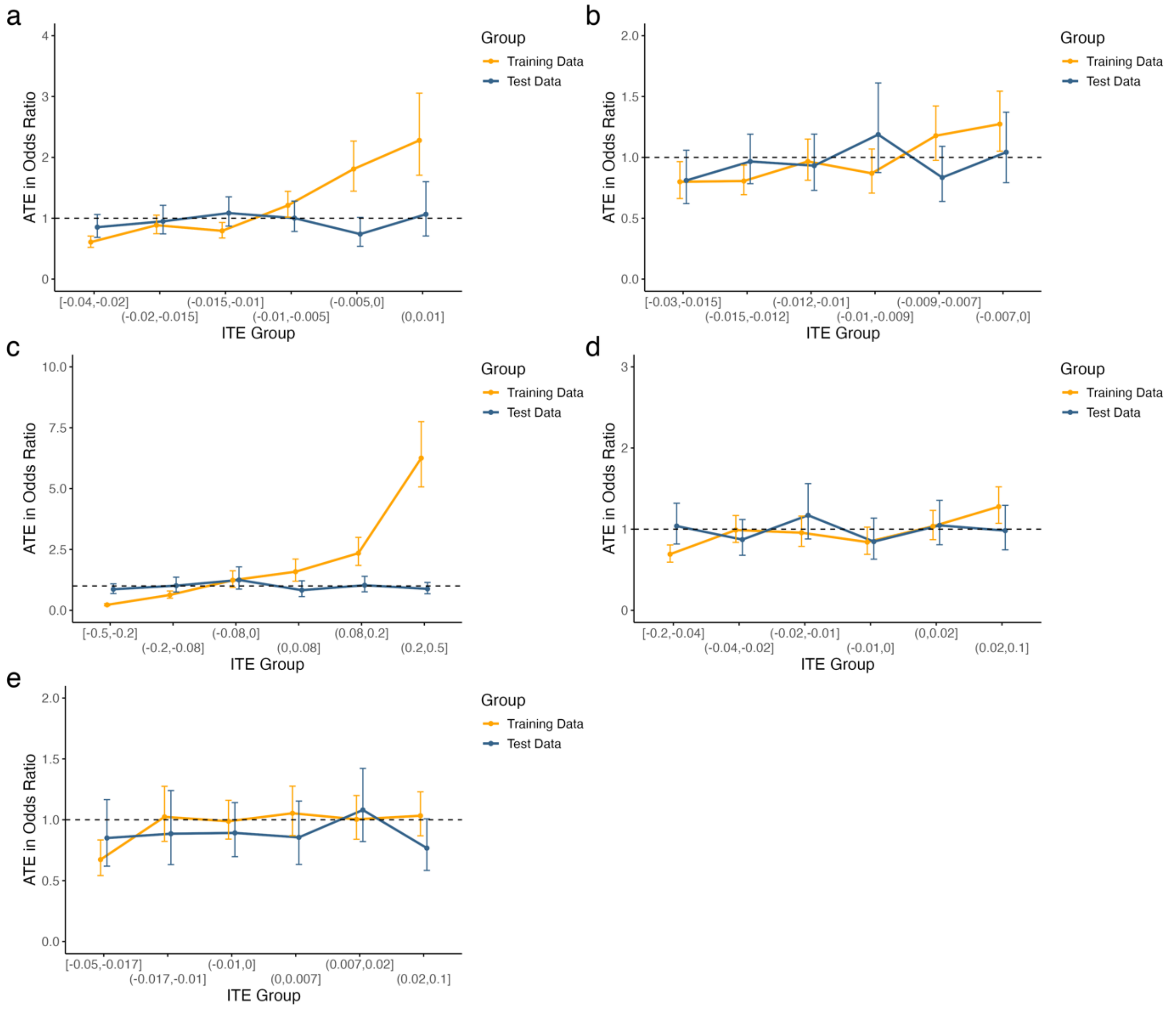

Figure S3.5.2. Internal validation on the CAST dataset: ATE-ITE comparative analysis. Line plots depict ATE in risk ratio within different ITE subgroups and provide the confidence intervals at 95% level. Orange represents the training data, and blue indicates the test data. The horizontal dashed line at 1.0 means no treatment effects. The outcome variable is death or dependency at 4 weeks. **a**, Causal Forest. **b**, Bayesian Causal Forest. **c**, Model-based Recursive Partitioning. **d**, CVAE. **e**, GANITE. Abbreviations: CAST: the Chinese Acute Stroke Trial, ITE: individualized treatment effect, ATE: average treatment effect.

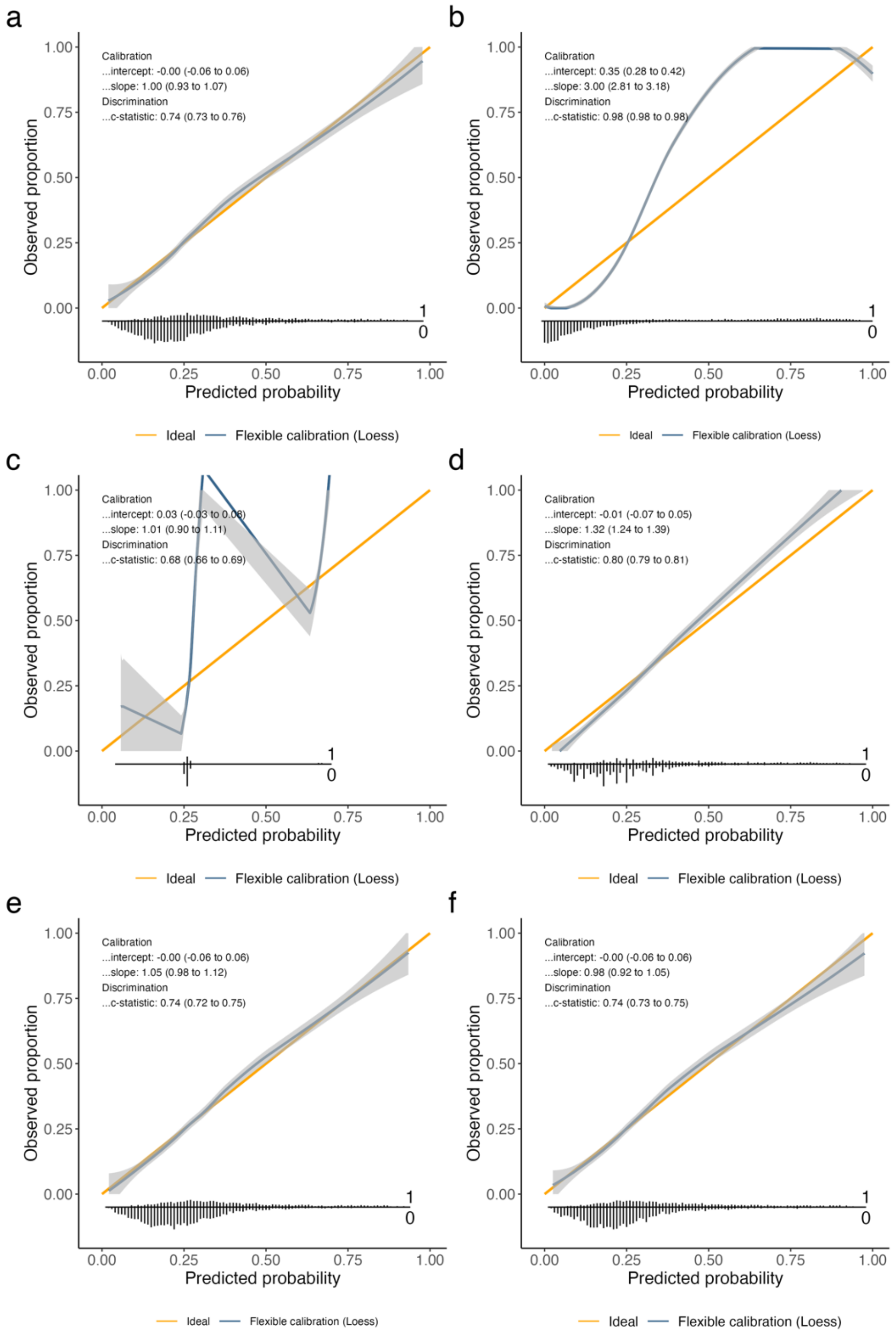

Figure S3.6.1. Internal validation on the CAST training dataset: calibration plot of predicted outcome in the treatment group. The orange line indicates ideal calibration. The outcome variable is death or dependency at 4 weeks. **a**, T-learner Logistic Regression. **b**, T-learner Random Forest. **c**, T-learner Support Vector Machine. **d**, T-learner XGBoost. **e**, T-learner Penalized Logistic Regression. **f**, S-learner Logistic Regression. Abbreviations: CAST: the Chinese Acute Stroke Trials.

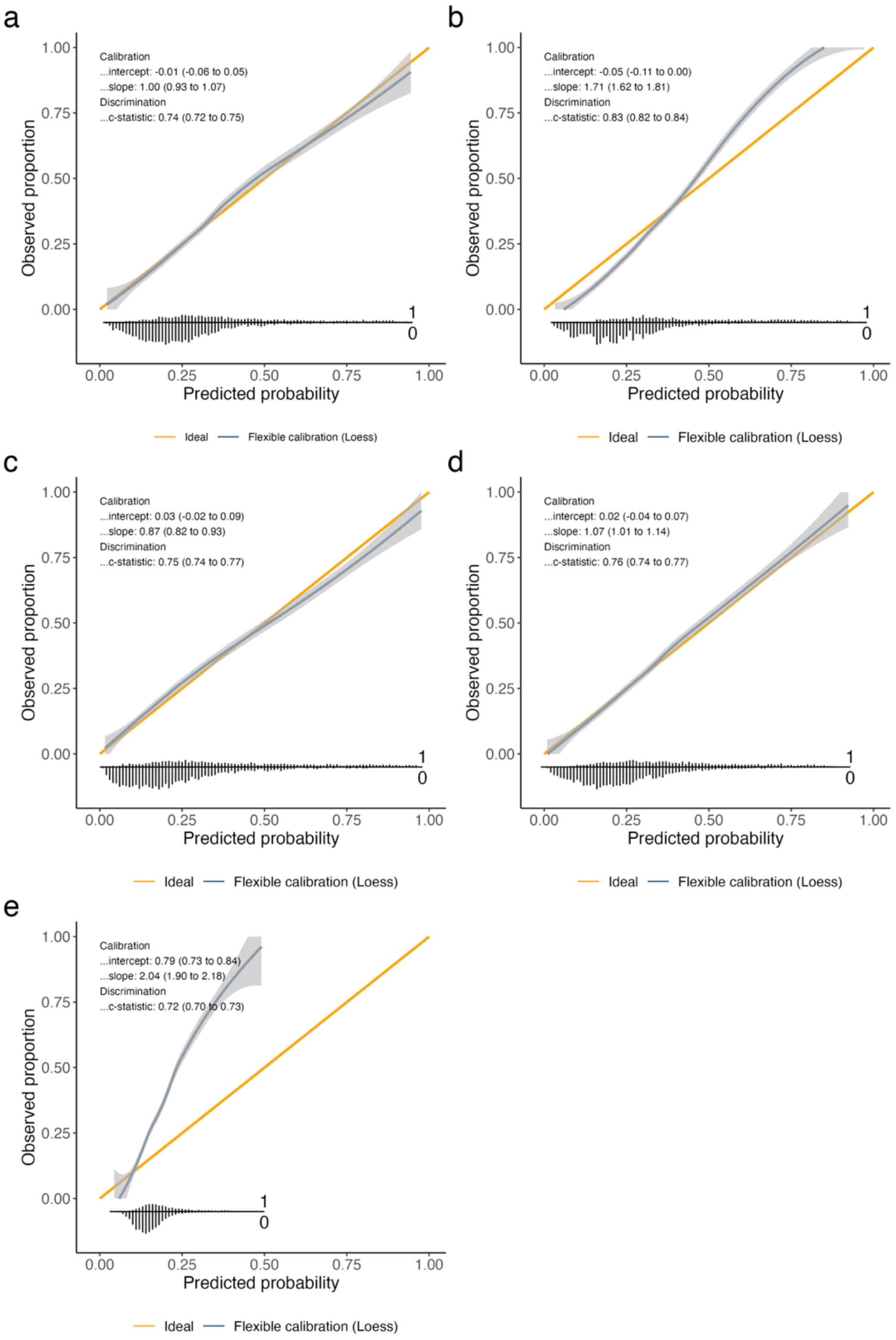

Figure S3.6.2. Internal validation on the CAST training dataset: calibration plot of predicted outcome in the treatment group. The orange line indicates ideal calibration. The outcome variable is death or dependency at 4 weeks. **a**, S-learner Penalized Logistic Regression. **b**, S-learner XGBoost **c**, S-learner BART. **d**, CVAE. **e**, GANITE. Abbreviations: CAST: the Chinese Acute Stroke Trial.

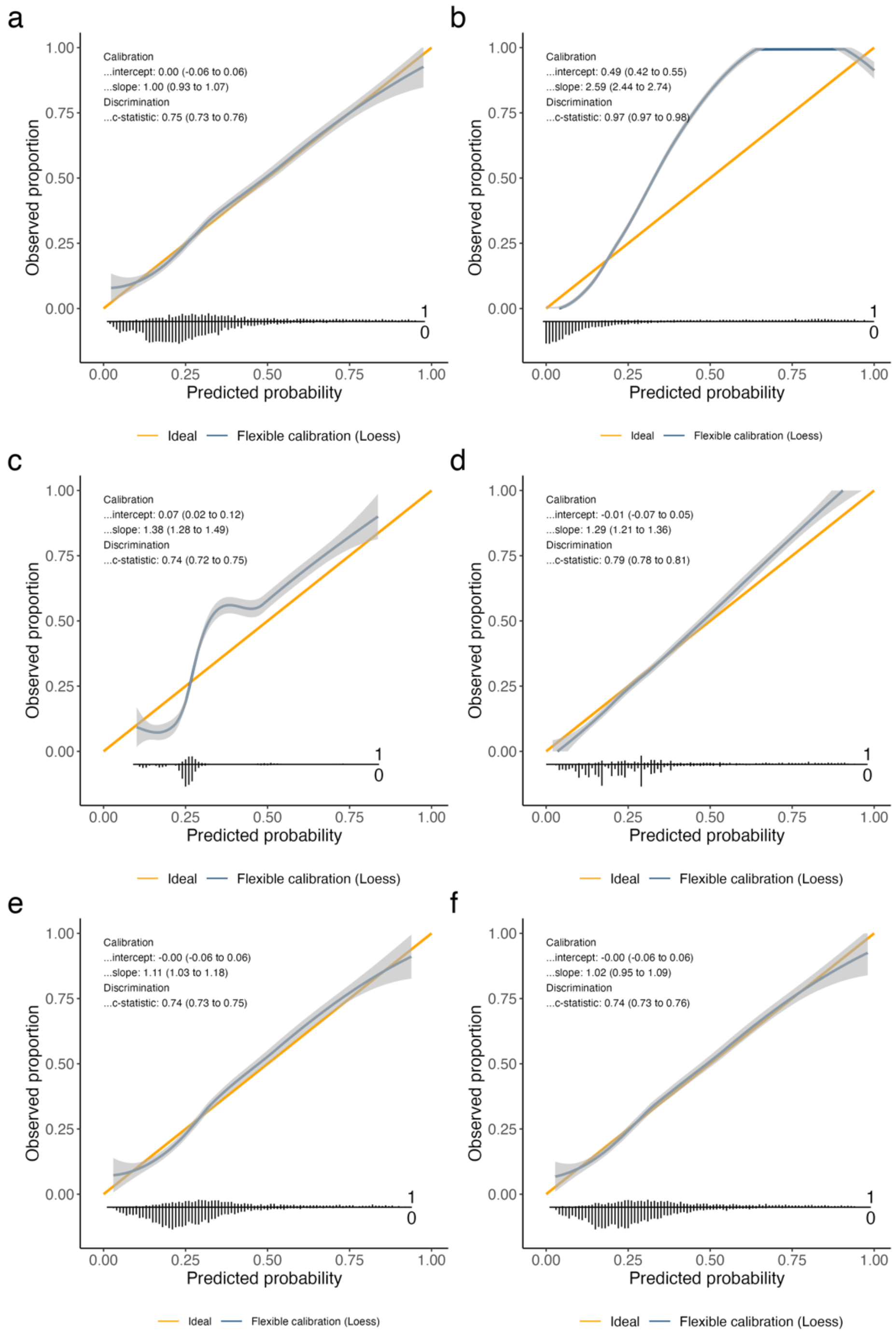

Figure S3.7.1. Internal validation on the CAST training dataset: calibration plot of predicted outcome in the control group. The orange line indicates ideal calibration. The outcome variable is death or dependency at 4 weeks. **a**, T-learner Logistic Regression. **b**, T-learner Random Forest. **c**, T-learner Support Vector Machine. **d**, T-learner XGBoost. **e**, T-learner Penalized Logistic Regression. **f**, S-learner Logistic Regression. Abbreviations: CAST: the Chinese Acute Stroke Trial.

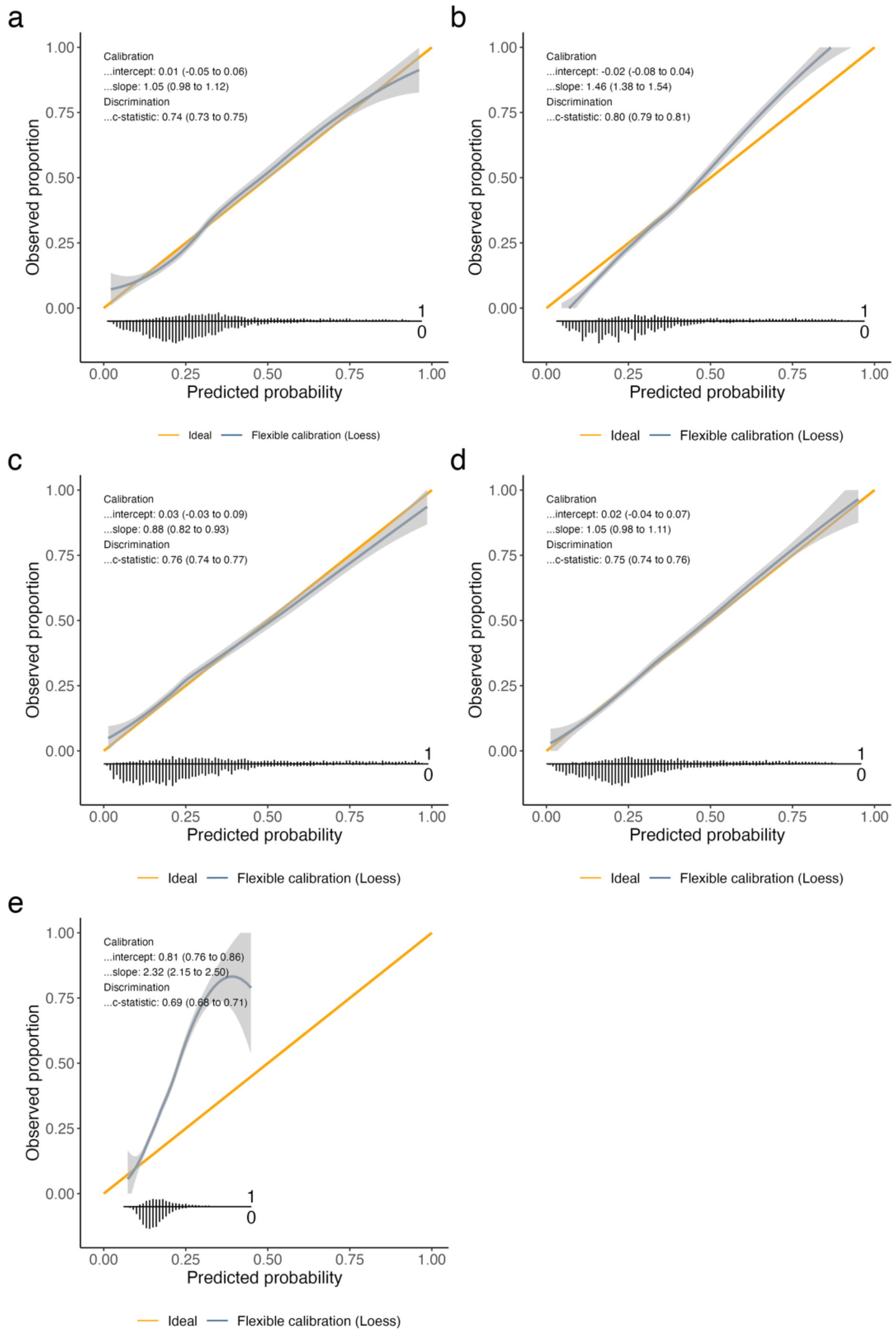

Figure S3.7.2. Internal validation on the CAST training dataset: calibration plot of predicted outcome in the control group. The orange line indicates ideal calibration. The outcome variable is death or dependency at 4 weeks. **a**, S-learner Penalized Logistic Regression. **b**, S-learner XGBoost **c**, S-learner BART. **d**, CVAE. **e**, GANITE. Abbreviations: CAST: the Chinese Acute Stroke Trial.

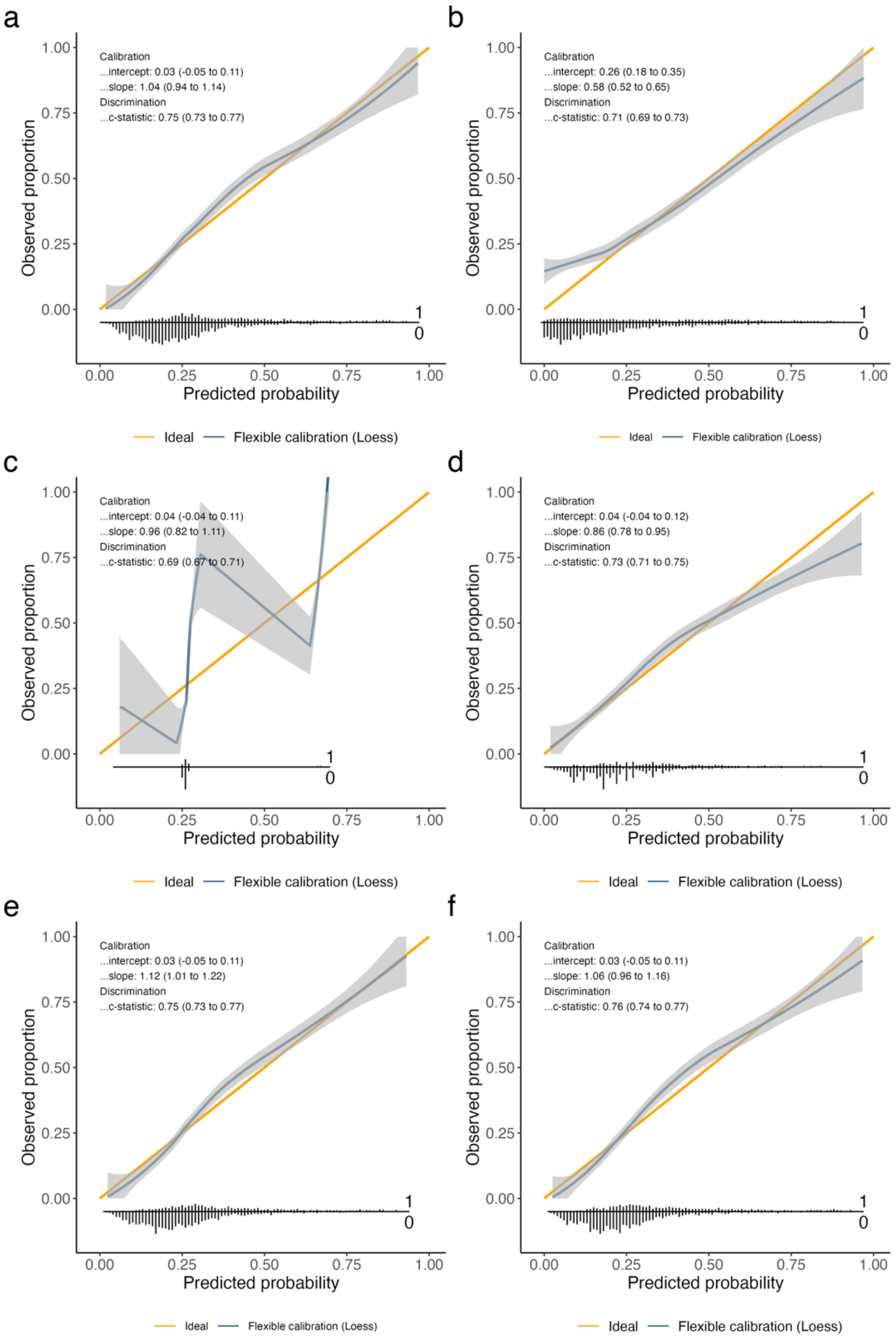

Figure S3.8.1. Internal validation on the CAST test dataset: calibration plot of predicted outcome in the treatment group. The orange line indicates ideal calibration. The outcome variable is death or dependency at 4 weeks. **a**, T-learner Logistic Regression. **b**, T-learner Random Forest. **c**, T-learner Support Vector Machine. **d**, T-learner XGBoost. **e**, T-learner Penalized Logistic Regression. **f**, S-learner Logistic Regression. Abbreviations: CAST: the Chinese Acute Stroke Trial.

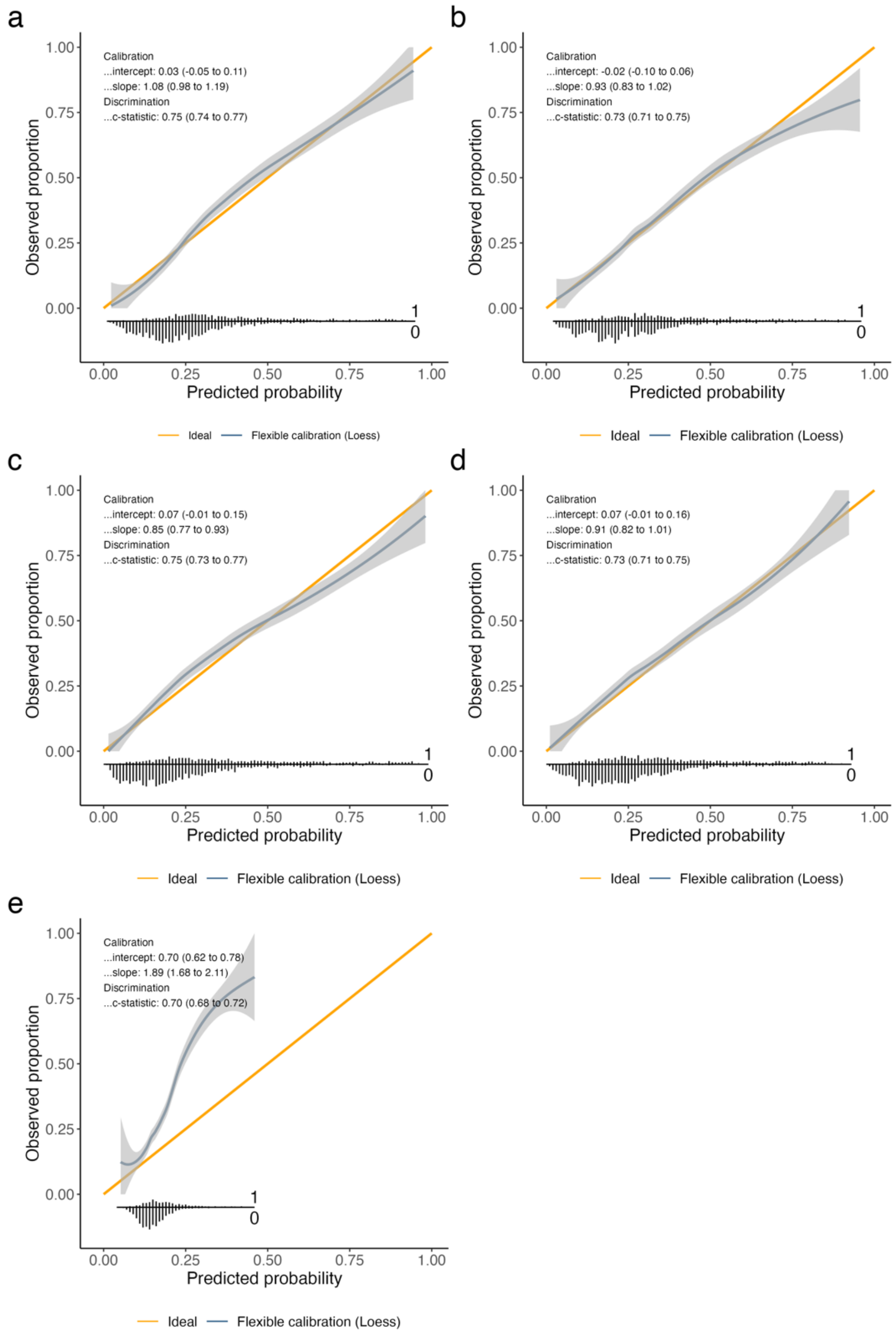

Figure S3.8.2. Internal validation on the CAST test dataset: calibration plot of predicted outcome in the treatment group. The orange line indicates ideal calibration. The outcome variable is death or dependency at 4 weeks. **a**, S-learner Penalized Logistic Regression. **b**, S-learner XGBoost **c**, S-learner BART. **d**, CVAE. **e**, GANITE. Abbreviations: CAST: the Chinese Acute Stroke Trial.

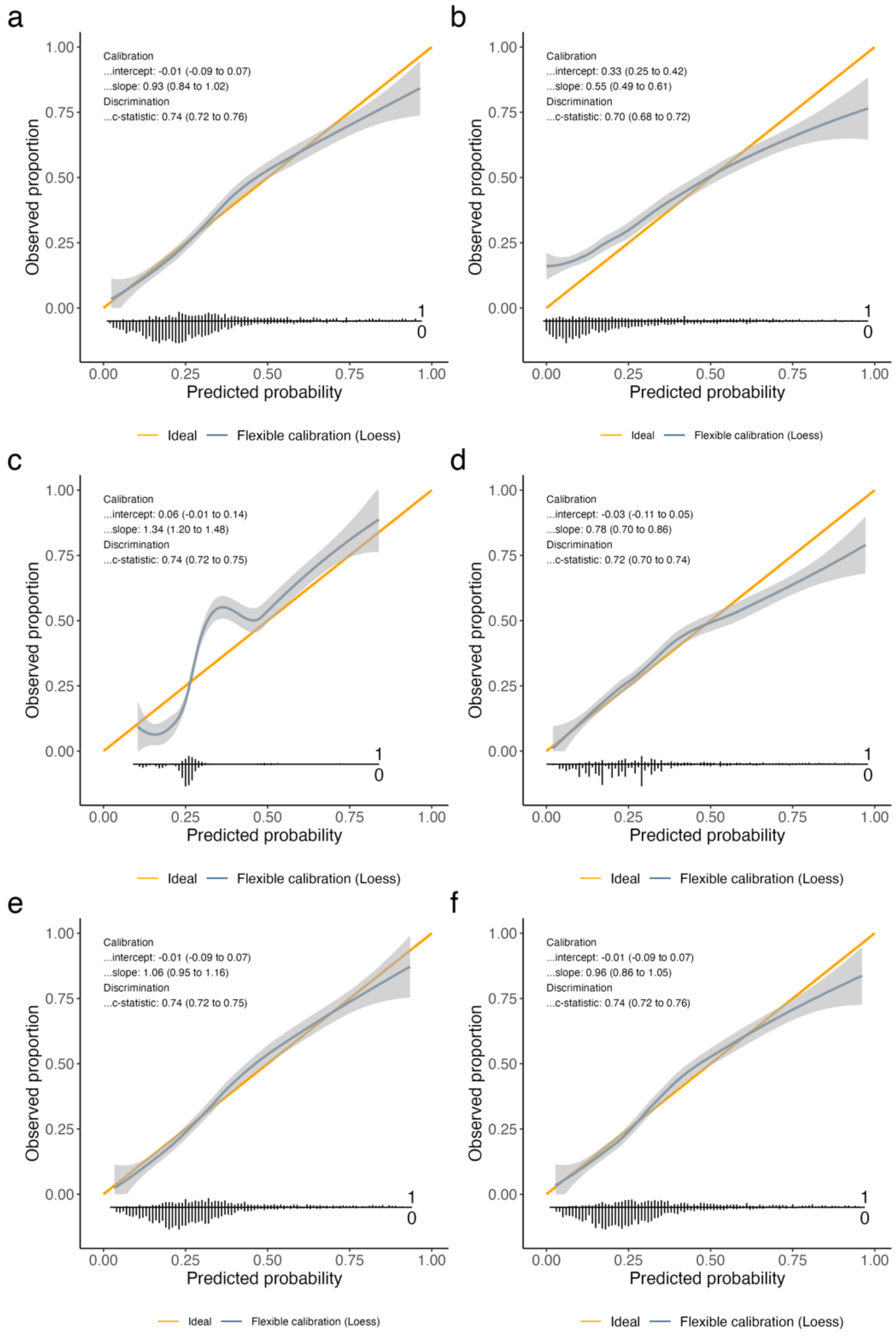

Figure S3.9.1. Internal validation on the CAST test dataset: calibration plot of predicted outcome in the control group. The orange line indicates ideal calibration. The outcome variable is death or dependency at 4 weeks. **a**, T-learner Logistic Regression. **b**, T-learner Random Forest. **c**, T-learner Support Vector Machine. **d**, T-learner XGBoost. **e**, T-learner Penalized Logistic Regression. **f**, S-learner Logistic Regression. Abbreviations: CAST: the Chinese Acute Stroke Trial.

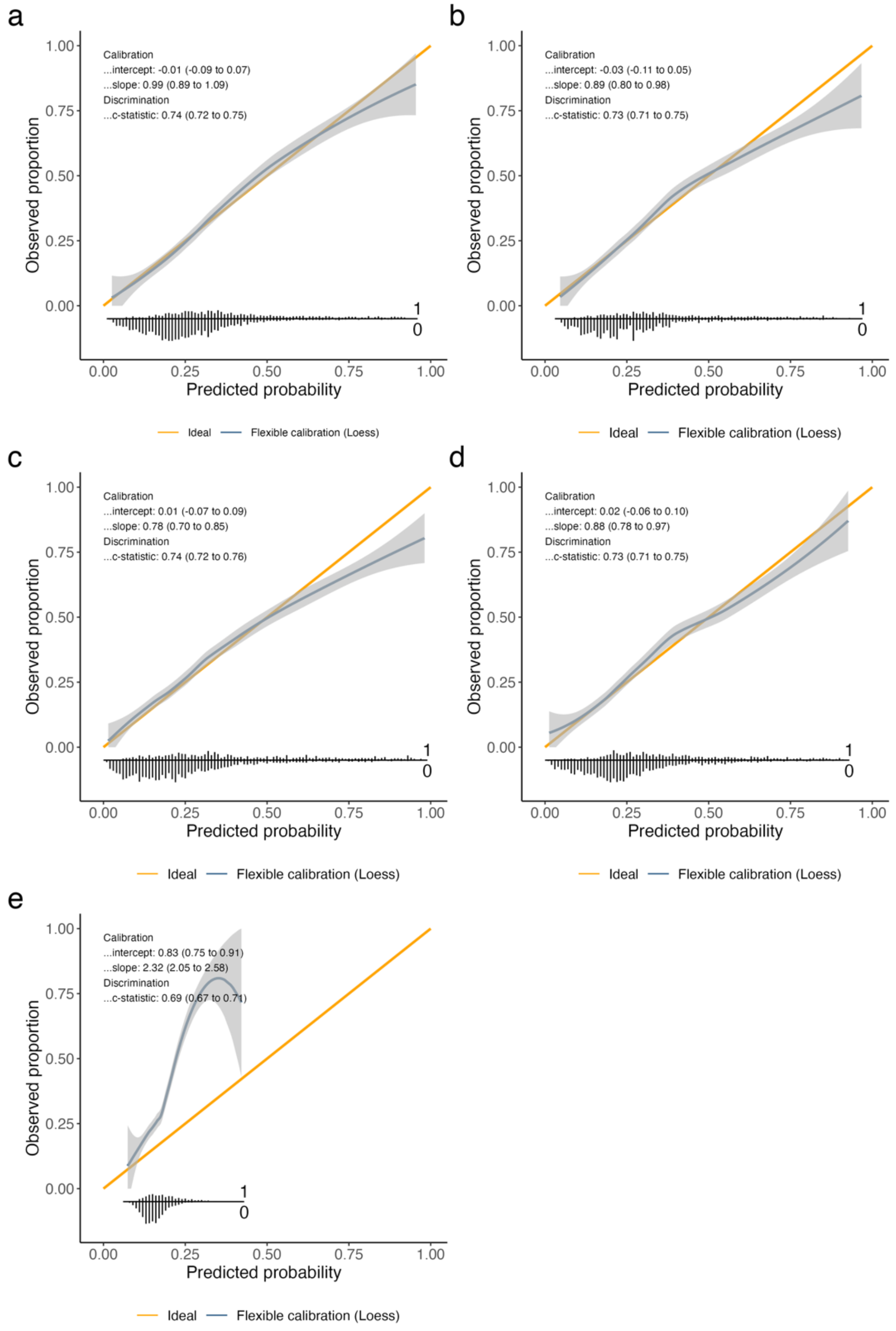

Figure S3.9.2. Internal validation on the CAST test dataset: calibration plot of predicted outcome in the control group. The orange line indicates ideal calibration. The outcome variable is death or dependency at 4 weeks. **a**, S-learner Penalized Logistic Regression. **b**, S-learner XGBoost **c**, S-learner BART. **d**, CVAE. **e**, GANITE. Abbreviations: CAST: the Chinese Acute Stroke Trial.

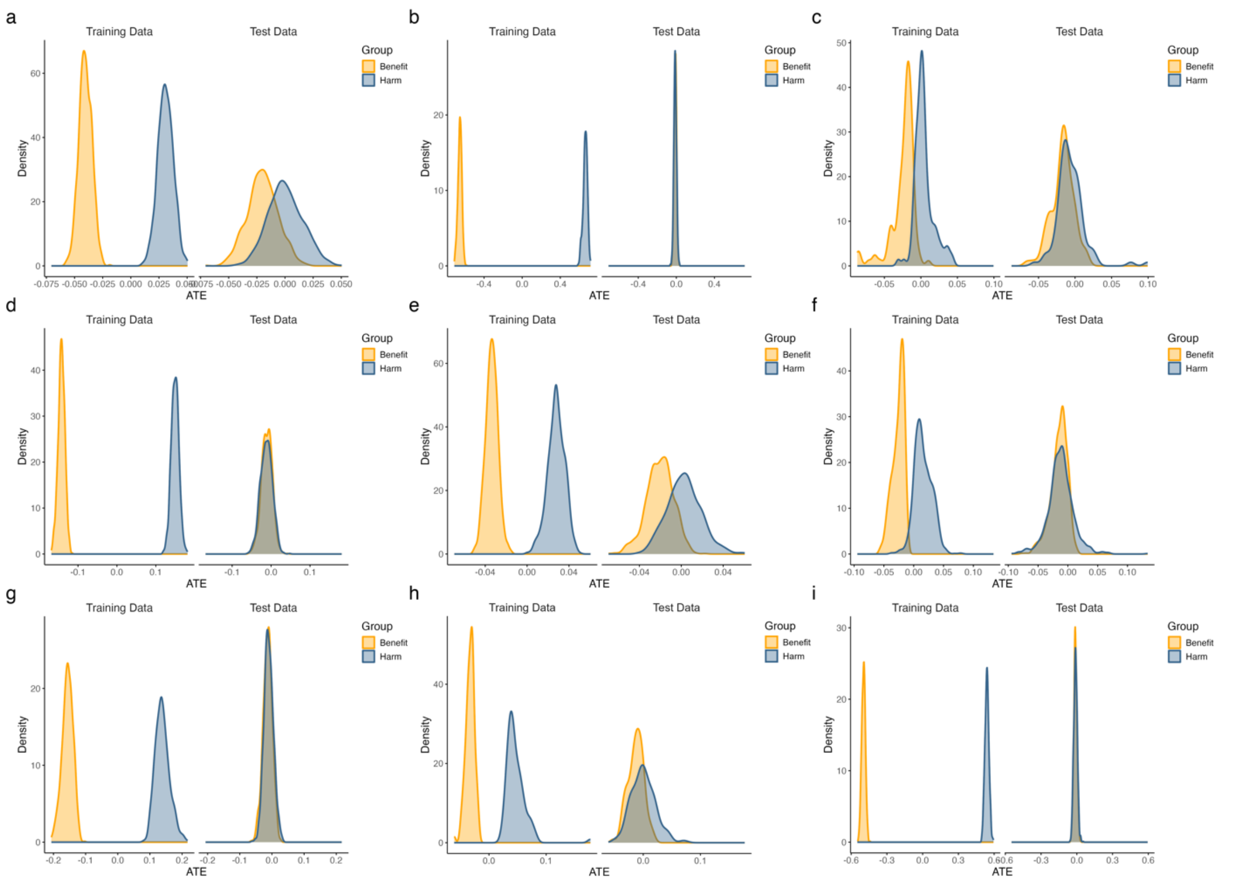

Figure S3.10.1. Internal validation on the CAST dataset: density comparative analysis of causal machine learning-based individualized treatment effects. Density plots depict the distributions of ATE in training data and test data stratified by benefit (orange) and harm (blue) groups. The distribution of ATE comes from 1000 (**a, b, d, e, f, g, i**) or 100 (**c, h**) random train-test splits experiments. Benefit means negative ITE values and harm means positive ITE values. The outcome variable is death or dependency at 4 weeks. **a**, T-learner Logistic Regression. **b**, T-learner Random Forest. **c**, T-learner Support Vector Machine. **d**, T-learner XGBoost. **e**, T-learner Penalized Logistic Regression. **f**, S-learner Penalized Logistic Regression. **g**, S-learner XGBoost. **h**, S-learner BART. **i**, X-learner Random Forest. Abbreviations: CAST: the Chinese Acute Stroke Trial, ITE: individualized treatment effect, ATE: average treatment effect.

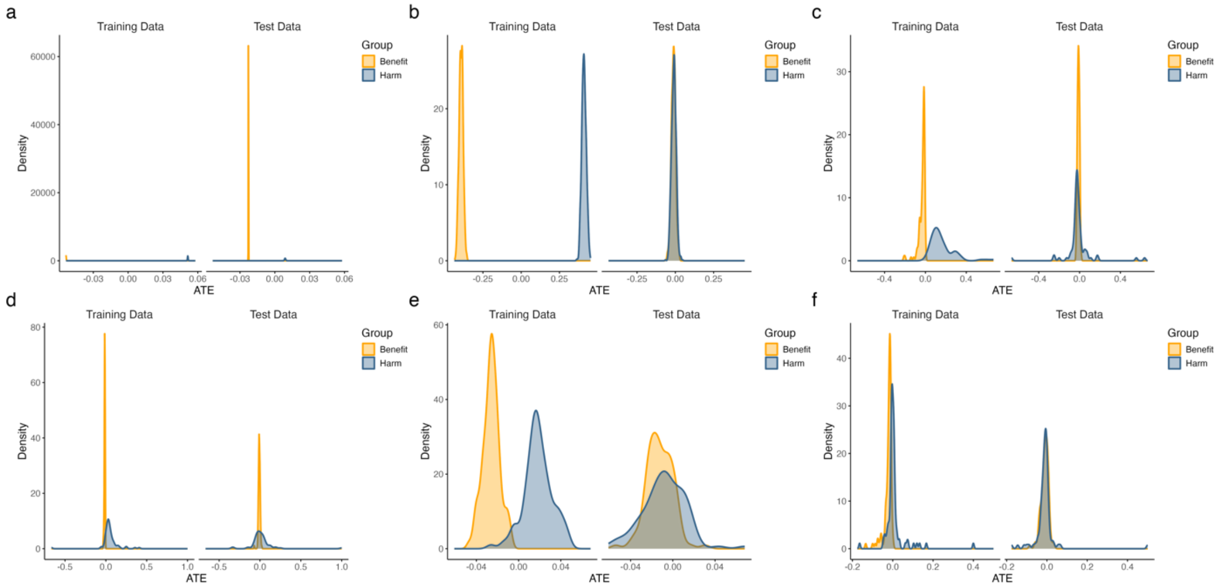

Figure S3.10.2. Internal validation on the CAST dataset: density comparative analysis of causal machine learning-based individualized treatment effects. Density plots depict the distributions of ATE in training data and test data stratified by benefit (orange) and harm (blue) groups. The distribution of ATE comes from 1000 (**b**) or 100 (**a, c, d, e, f**) random train-test splits experiments. Benefit means negative ITE values and harm means positive ITE values. The outcome variable is death or dependency at 4 weeks. **a**, X-learner BART. **b**, DR-learner Random Forest. **c**, Causal Forest. **d**, Bayesian Causal Forest. **e**, CVAE. **f**, GANITE. Abbreviations: CAST: the Chinese Acute Stroke Trial, ITE: individualized treatment effect, ATE: average treatment effect.

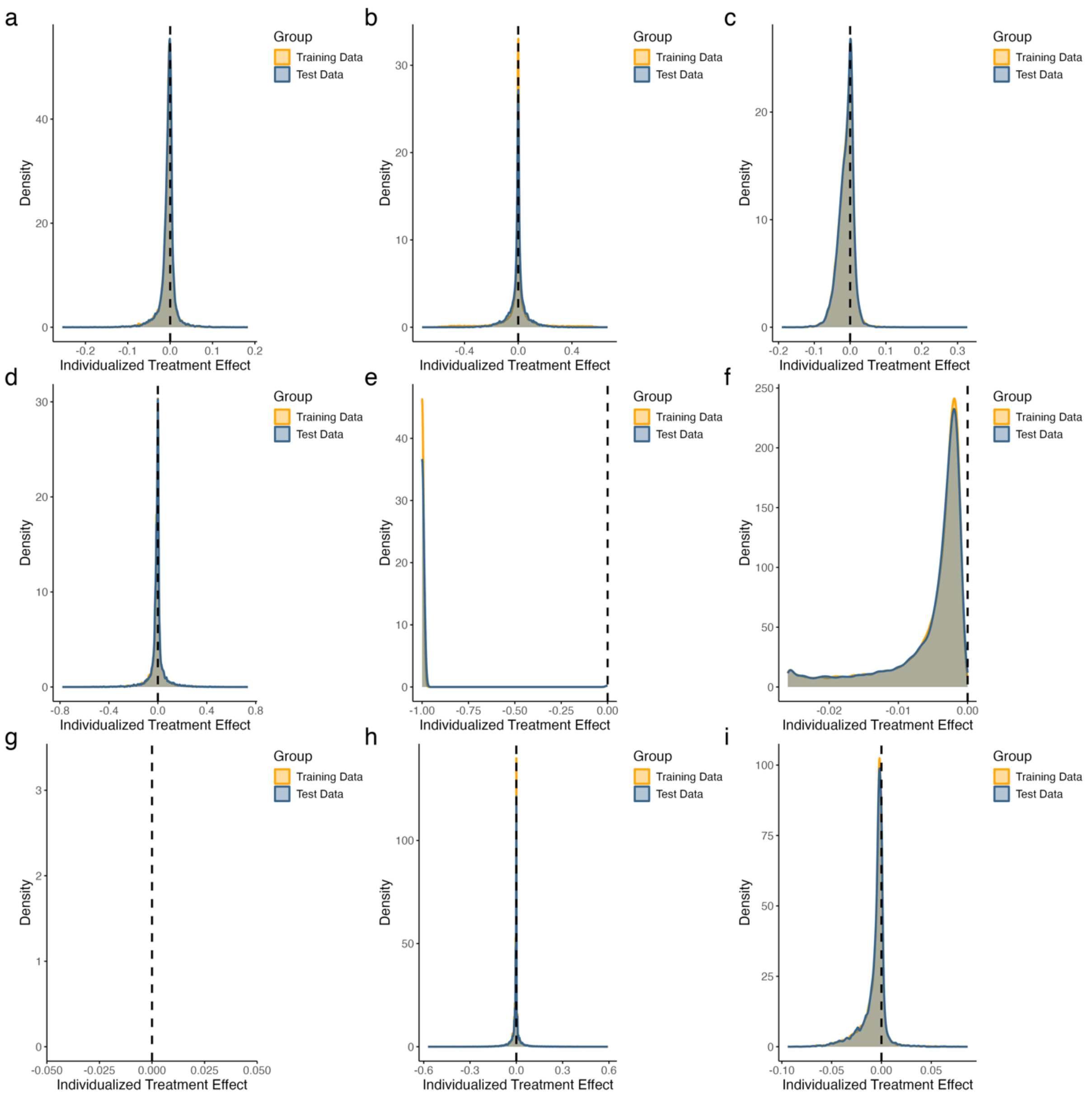

Figure S4.1.1. Internal validation on the combined IST and CAST dataset: density plots of causal machine learning-based individualized treatment effects. Orange represents the training data, and blue indicates the test data. The outcome variable is death at 14 days for IST and death at 4 weeks for CAST. **a**, T-learner Logistic Regression. **b**, T-learner Random Forest. **c**, T-learner Support Vector Machine. **d**, T-learner XGBoost. **e**, T-learner Penalized Logistic Regression. **f**, S-learner Logistic Regression. **g**, S-learner Penalized Logistic Regression. **h**, S-learner XGBoost. **i**, S-learner BART. Abbreviations: IST: the International Stroke Trial, CAST: the Chinese Acute Stroke Trial, ITE: individualized treatment effect.

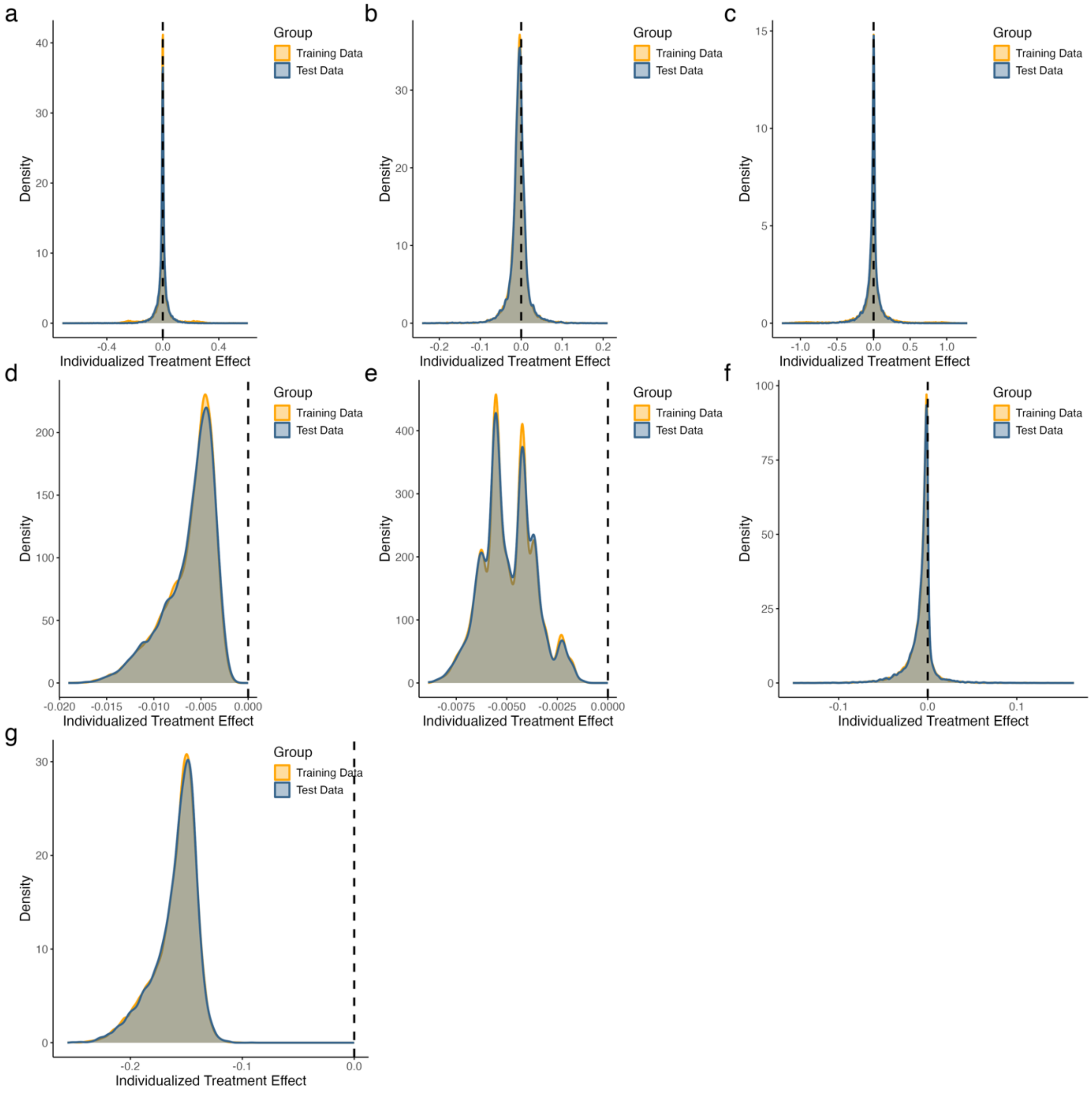

Figure S4.1.2. Internal validation on the combined IST and CAST dataset: density plots of causal machine learning-based individualized treatment effects. Orange represents the training data, and blue indicates the test data. The outcome variable is death at 14 days for IST and death at 4 weeks for CAST. **a**, X-learner Random Forest. **b**, X-learner BART. **c**, DR-learner Random Forest. **d**, Causal Forest. **e**, Bayesian Causal Forest. **f**, CVAE. **g**, GANITE. Abbreviations: IST: the International Stroke Trial, CAST: the Chinese Acute Stroke Trial, ITE: individualized treatment effect.

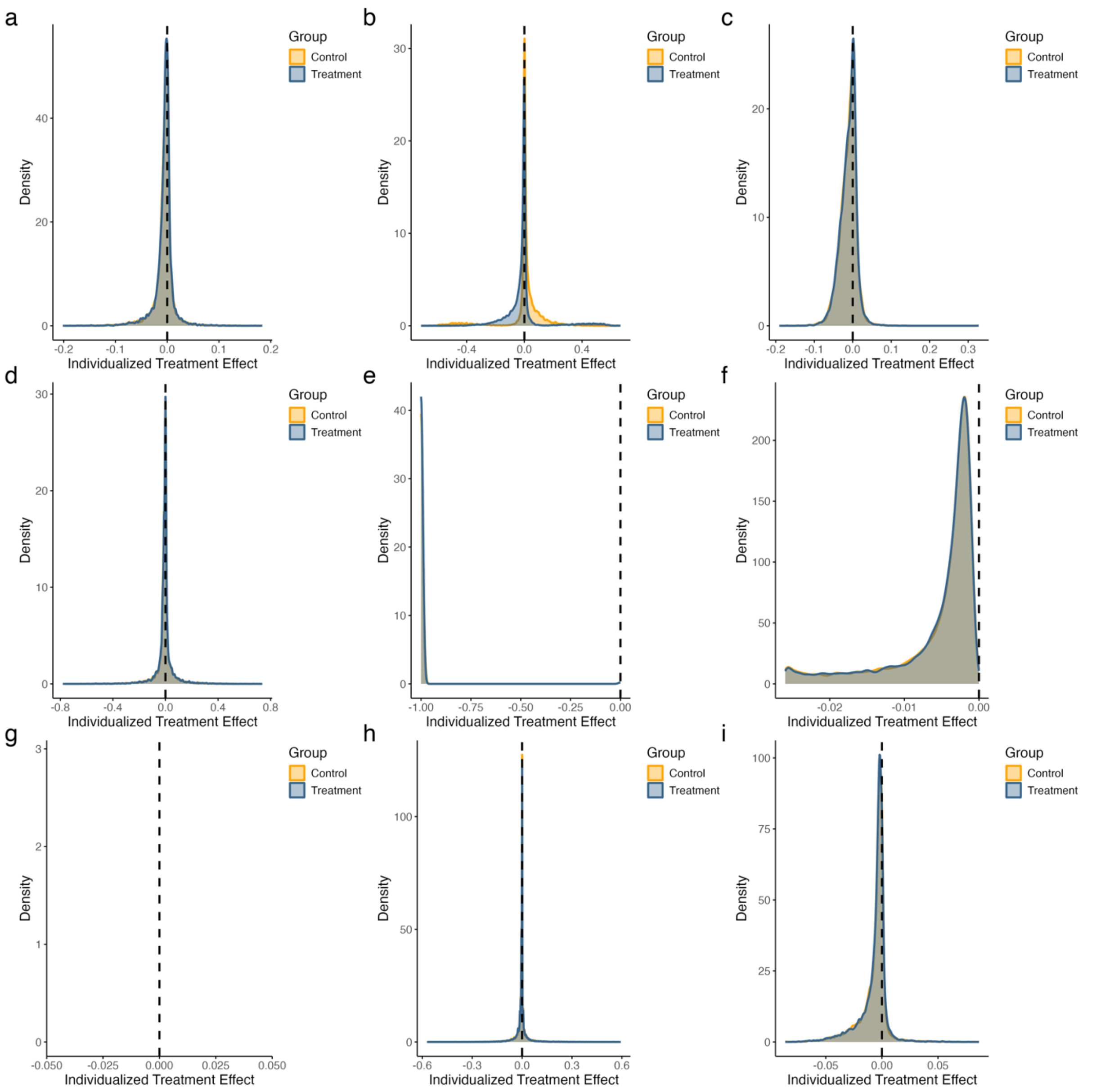

Figure S4.2.1. Internal validation on the combined IST and CAST training dataset: density plots of causal machine learning-based individualized treatment effects. Orange represents the control group, and blue indicates the treatment group. The outcome variable is death at 14 days for IST and death at 4 weeks for CAST. **a**, T-learner Logistic Regression. **b**, T-learner Random Forest. **c**, T-learner Support Vector Machine. **d**, T-learner XGBoost. **e**, T-learner Penalized Logistic Regression. **f**, S-learner Logistic Regression. **g**, S-learner Penalized Logistic Regression. **h**, S-learner XGBoost. **i**, S-learner BART. Abbreviations: IST: the International Stroke Trial, CAST: the Chinese Acute Stroke Trial, ITE: individualized treatment effect.

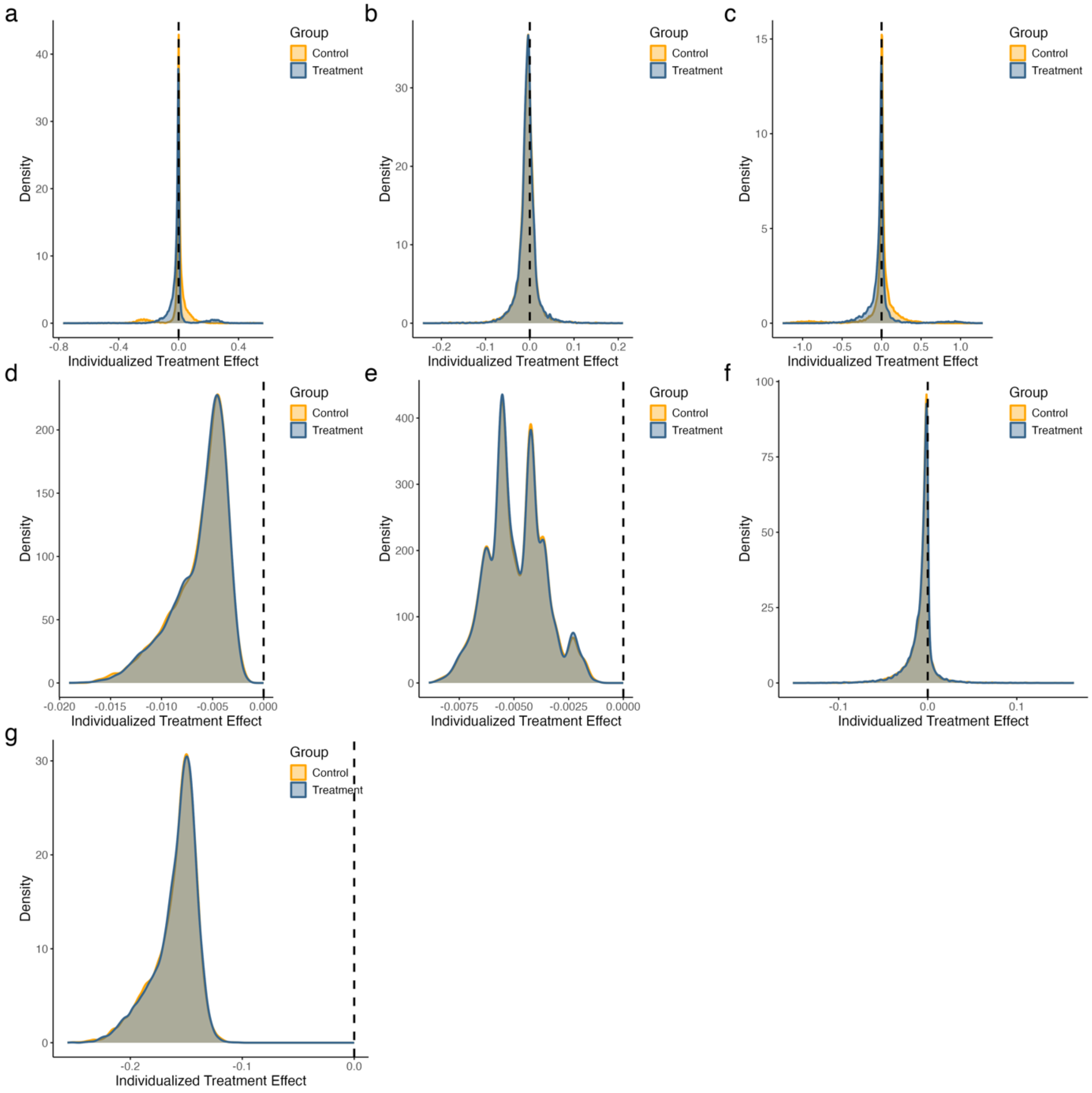

Figure S4.2.2. Internal validation on the combined IST and CAST training dataset: density plots of causal machine learning-based individualized treatment effects. Orange represents the control group, and blue indicates the treatment group. The outcome variable is death at 14 days for IST and death at 4 weeks for CAST. **a**, X-learner Random Forest. **b**, X-learner BART. **c**, DR-learner Random Forest. **d**, Causal Forest. **e**, Bayesian Causal Forest. **f**, CVAE. **g**, GANITE. Abbreviations: IST: the International Stroke Trial, CAST: the Chinese Acute Stroke Trial, ITE: individualized treatment effect.

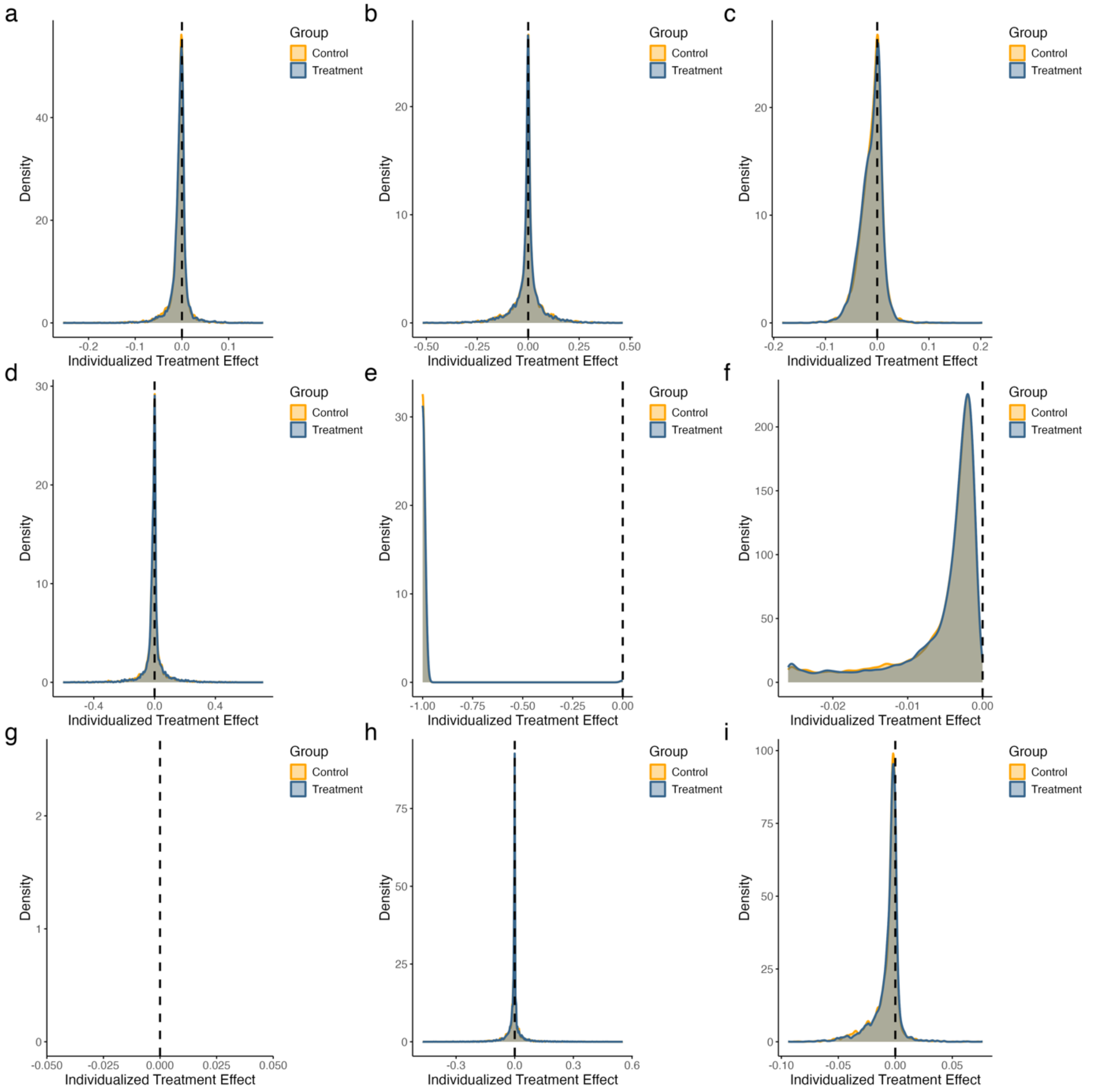

Figure S4.3.1. Internal validation on the combined IST and CAST test dataset: density plots of causal machine learning-based individualized treatment effects. Orange represents the control group, and blue indicates the treatment group. The outcome variable is death at 14 days for IST and death at 4 weeks for CAST. **a**, T-learner Logistic Regression. **b**, T-learner Random Forest. **c**, T-learner Support Vector Machine. **d**, T-learner XGBoost. **e**, T-learner Penalized Logistic Regression. **f**, S-learner Logistic Regression. **g**, S-learner Penalized Logistic Regression. **h**, S-learner XGBoost. **i**, S-learner BART. Abbreviations: IST: the International Stroke Trial, CAST: the Chinese Acute Stroke Trial, ITE: individualized treatment effect.

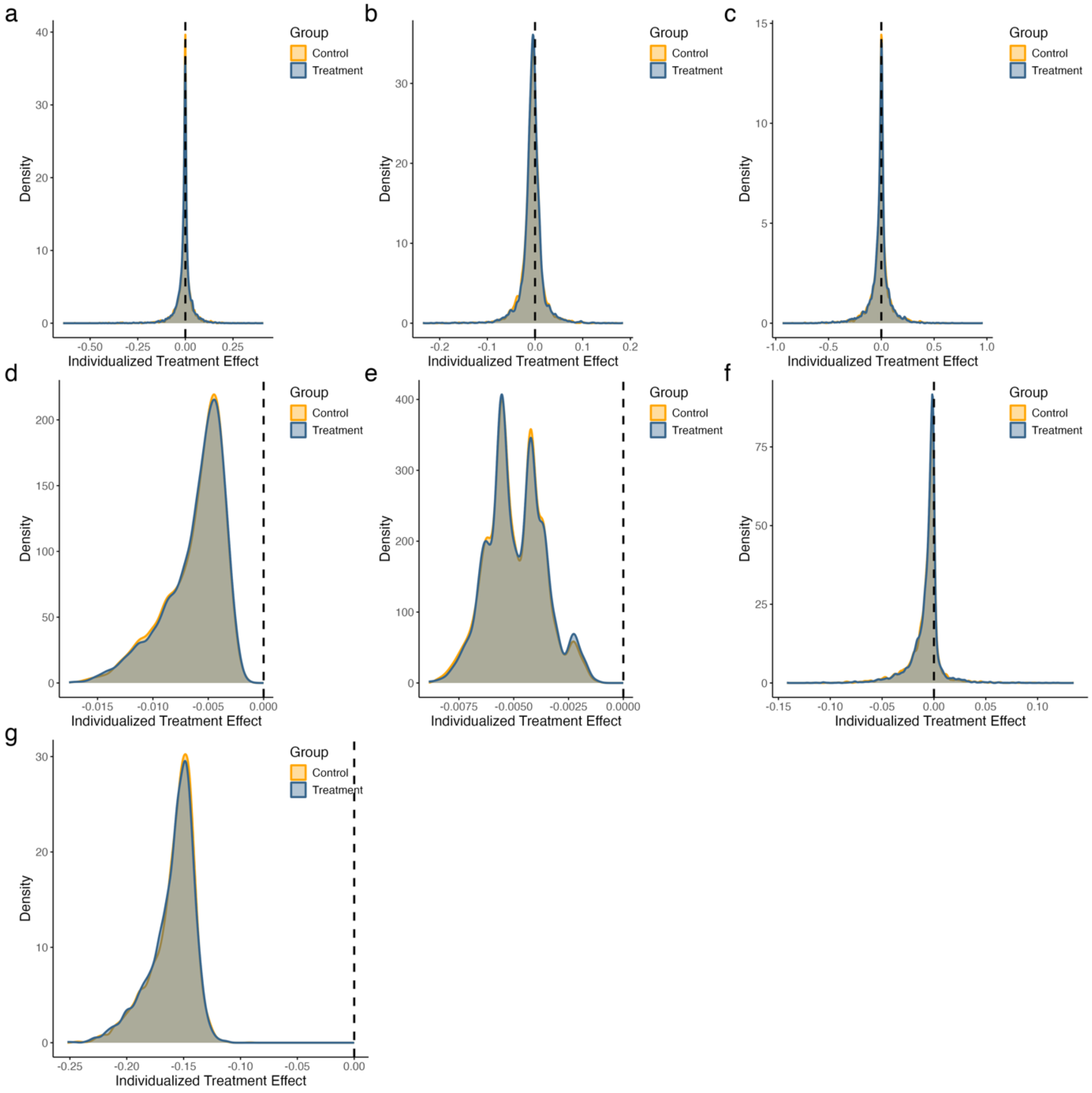

Figure S4.3.2. Internal validation on the combined IST and CAST test dataset: density plots of causal machine learning-based individualized treatment effects. Orange represents the control group, and blue indicates the treatment group. The outcome variable is death at 14 days for IST and death at 4 weeks for CAST. **a**, X-learner Random Forest. **b**, X-learner BART. **c**, DR-learner Random Forest. **d**, Causal Forest. **e**, Bayesian Causal Forest. **f**, CVAE. **g**, GANITE. Abbreviations: IST: the International Stroke Trial, CAST: the Chinese Acute Stroke Trial, ITE: individualized treatment effect.

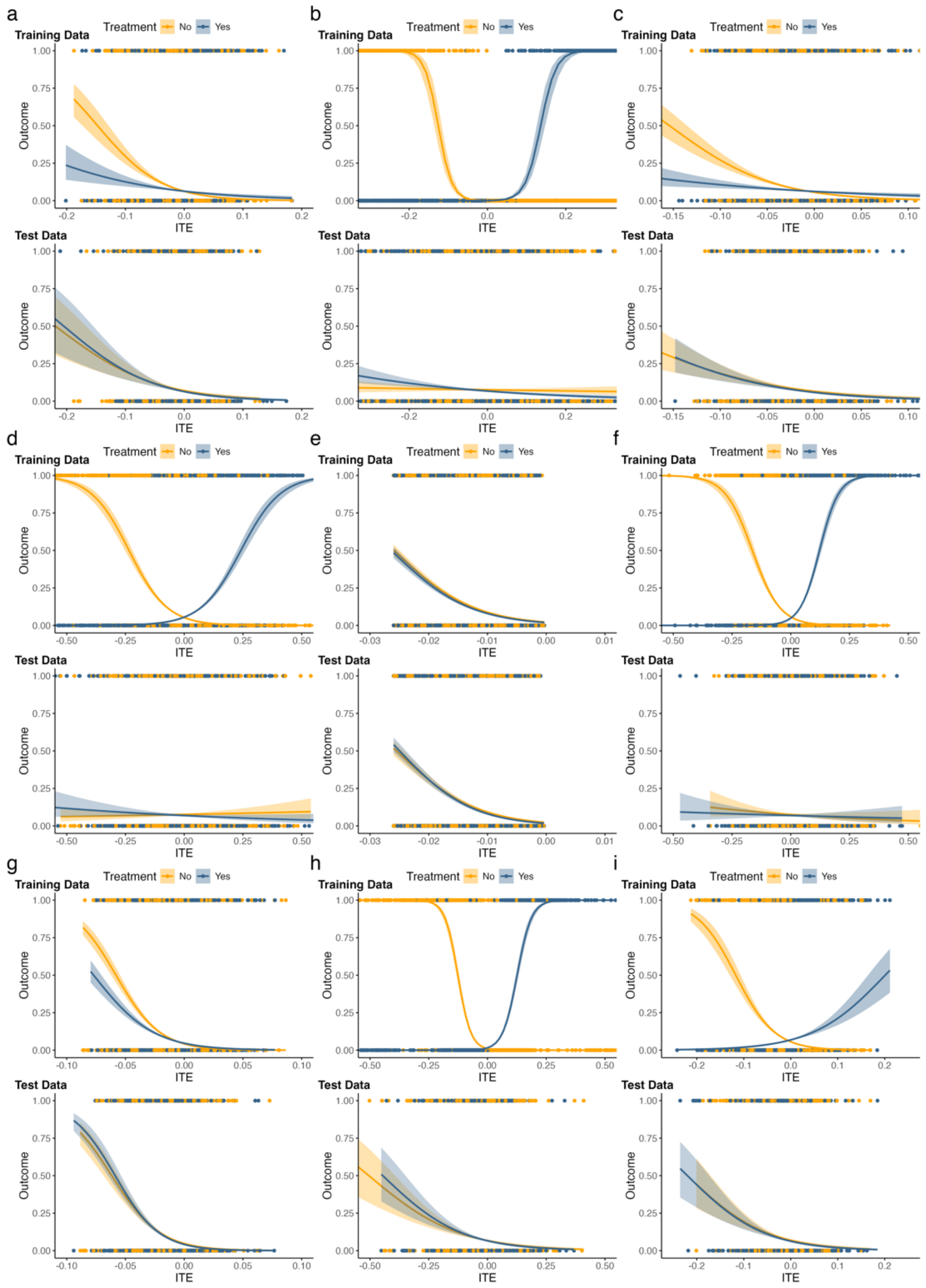

Figure S4.4.1. Internal validation on the combined IST and CAST dataset: outcome-ITE comparative analysis. Dot plots and line plots depict the true and fitted patient outcomes against estimated ITE values between training data and test data. Orange represents the control group, and blue indicates the treatment group. The outcome variable is death at 14 days for IST and death at 4 weeks for CAST. **a**, T-learner Logistic Regression. **b**, T-learner Random Forest. **c**, T-learner Support Vector Machine. **d**, T-learner XGBoost. **e**, S-learner Logistic Regression. **f**, S-learner XGBoost. **g**, S-learner BART. **h**, X-learner Random Forest. **i**, X-learner BART. Abbreviations: IST: the International Stroke Trial, CAST: the Chinese Acute Stroke Trial, ITE: individualized treatment effect.

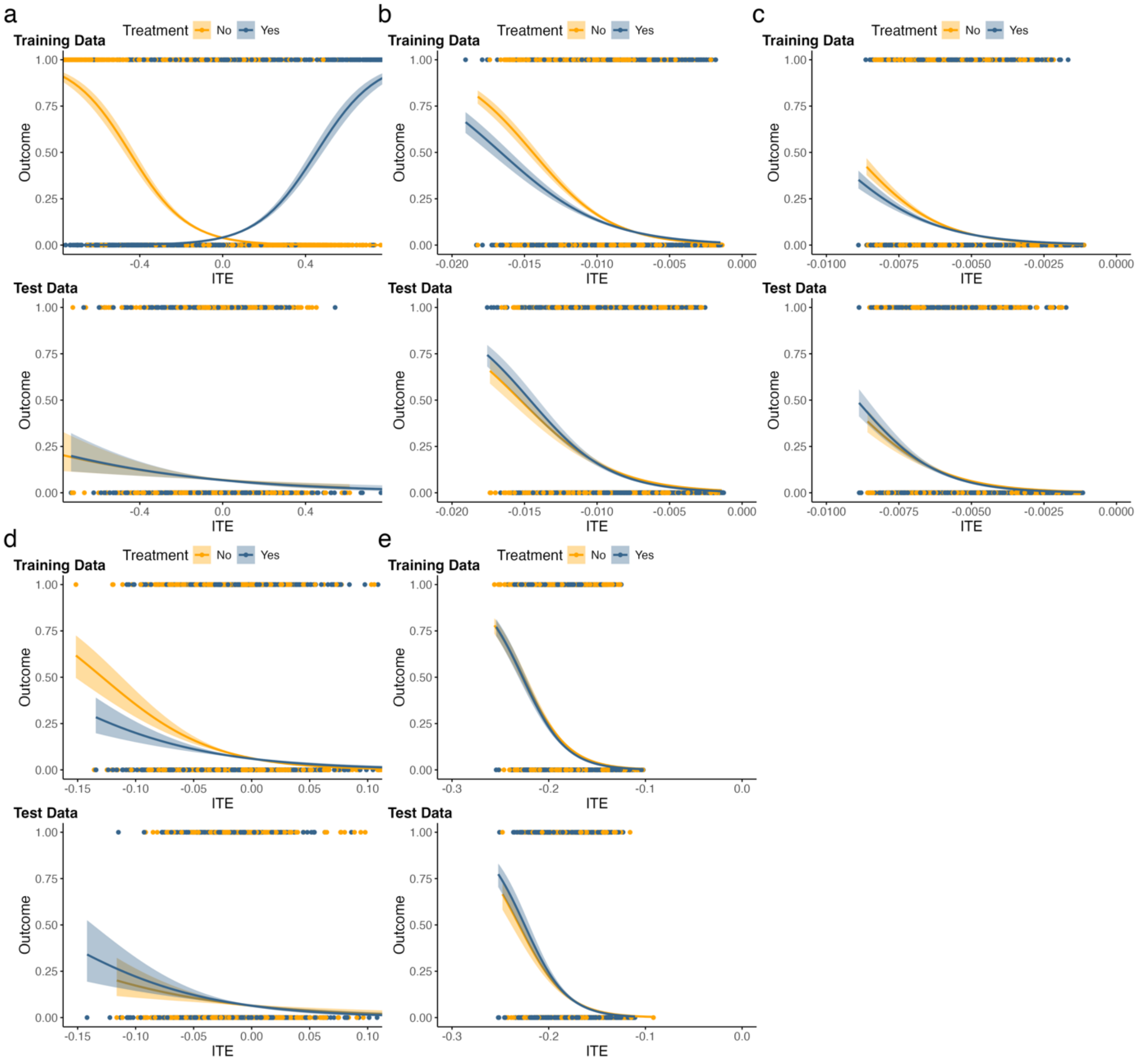

Figure S4.4.2. Internal validation on the combined IST and CAST dataset: outcome-ITE comparative analysis. Dot plots and line plots depict the true and fitted patient outcomes against estimated ITE values between training data and test data. Orange represents the control group, and blue indicates the treatment group. The outcome variable is death at 14 days for IST and death at 4 weeks for CAST. **a**, DR-learner Random Forest. **b**, Causal Forest. **c**, Bayesian Causal Forest. **d**, CVAE. **e**, GANITE. Abbreviations: IST: the International Stroke Trial, CAST: the Chinese Acute Stroke Trial, ITE: individualized treatment effect.

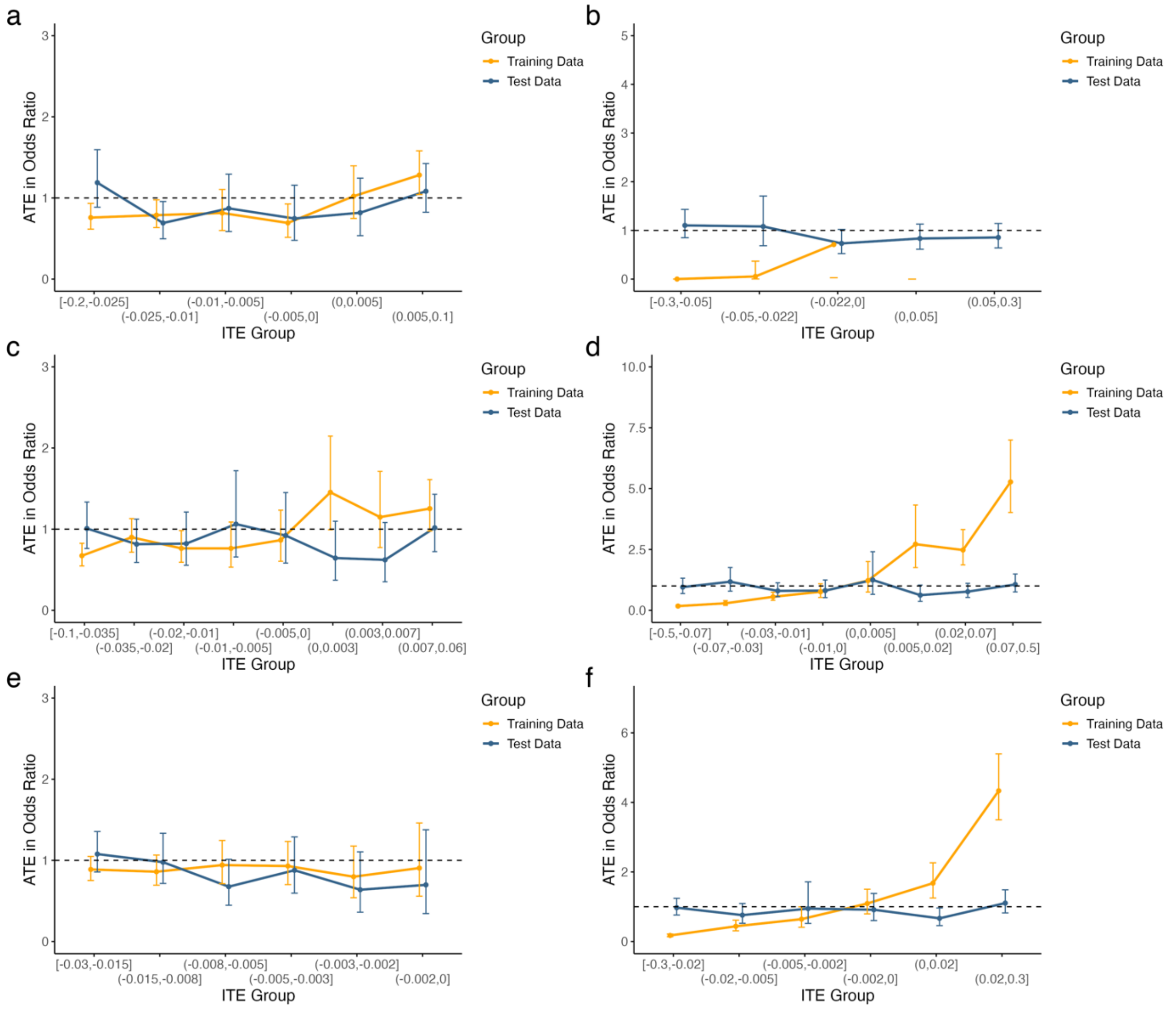

Figure S4.5.1. Internal validation on the combined IST and CAST dataset: ATE-ITE comparative analysis. Line plots depict ATE in risk ratio within different ITE subgroups and provide the confidence intervals at 95% level. Orange represents the training data, and blue indicates the test data. The horizontal dashed line at 1.0 means no treatment effects. The outcome variable is death at 14 days for IST and death at 4 weeks for CAST. **a**, T-learner Logistic Regression. **b**, T-learner Random Forest. **c**, T-learner Support Vector Machine. **d**, T-learner XGBoost. **e**, S-learner Logistic Regression. **f**, S-learner XGBoost. Abbreviations: IST: the International Stroke Trial, CAST: the Chinese Acute Stroke Trial, ITE: individualized treatment effect, ATE: average treatment effect.

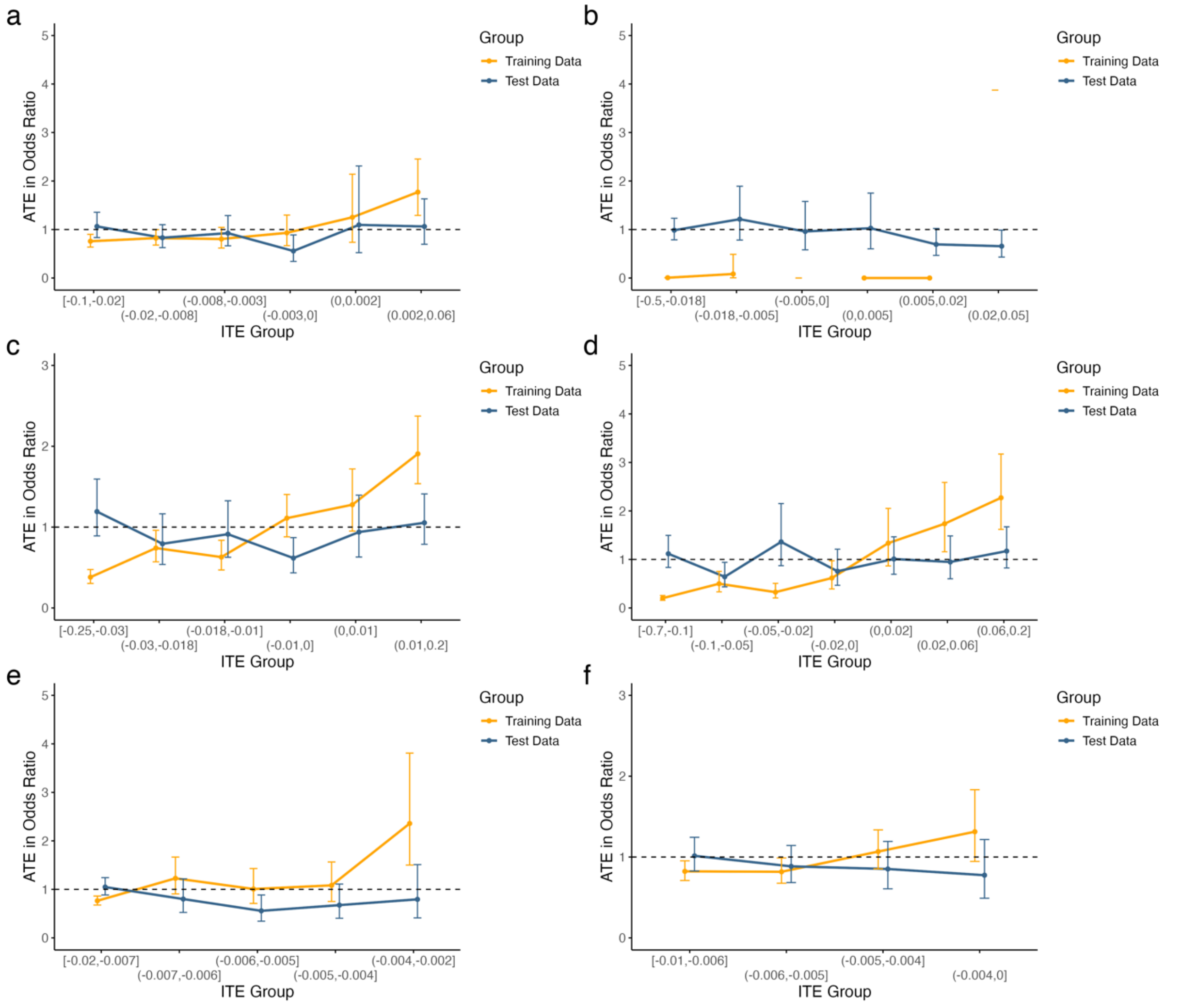

Figure S4.5.2. Internal validation on the combined IST and CAST dataset: ATE-ITE comparative analysis. Line plots depict ATE in risk ratio within different ITE subgroups and provide the confidence intervals at 95% level. Orange represents the training data, and blue indicates the test data. The horizontal dashed line at 1.0 means no treatment effects. The outcome variable is death at 14 days for IST and death at 4 weeks for CAST. **a**, S-learner BART. **b**, X-learner Random Forest. **c**, X-learner BART. **d**, DR-learner Random Forest. **e**, Causal Forest. **f**, Bayesian Causal Forest. Abbreviations: IST: the International Stroke Trial, CAST: the Chinese Acute Stroke Trial, ITE: individualized treatment effect, ATE: average treatment effect.

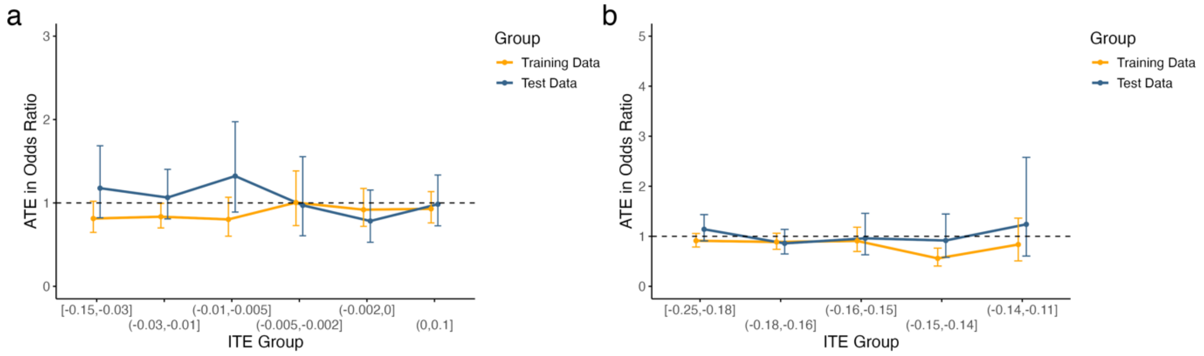

Figure S4.5.2. Internal validation on the combined IST and CAST dataset: ATE-ITE comparative analysis. Line plots depict ATE in risk ratio within different ITE subgroups and provide the confidence intervals at 95% level. Orange represents the training data, and blue indicates the test data. The horizontal dashed line at 1.0 means no treatment effects. The outcome variable is death at 14 days for IST and death at 4 weeks for CAST. **a**, CVAE. **b**, GANITE. Abbreviations: IST: the International Stroke Trial, CAST: the Chinese Acute Stroke Trial, ITE: individualized treatment effect, ATE: average treatment effect.

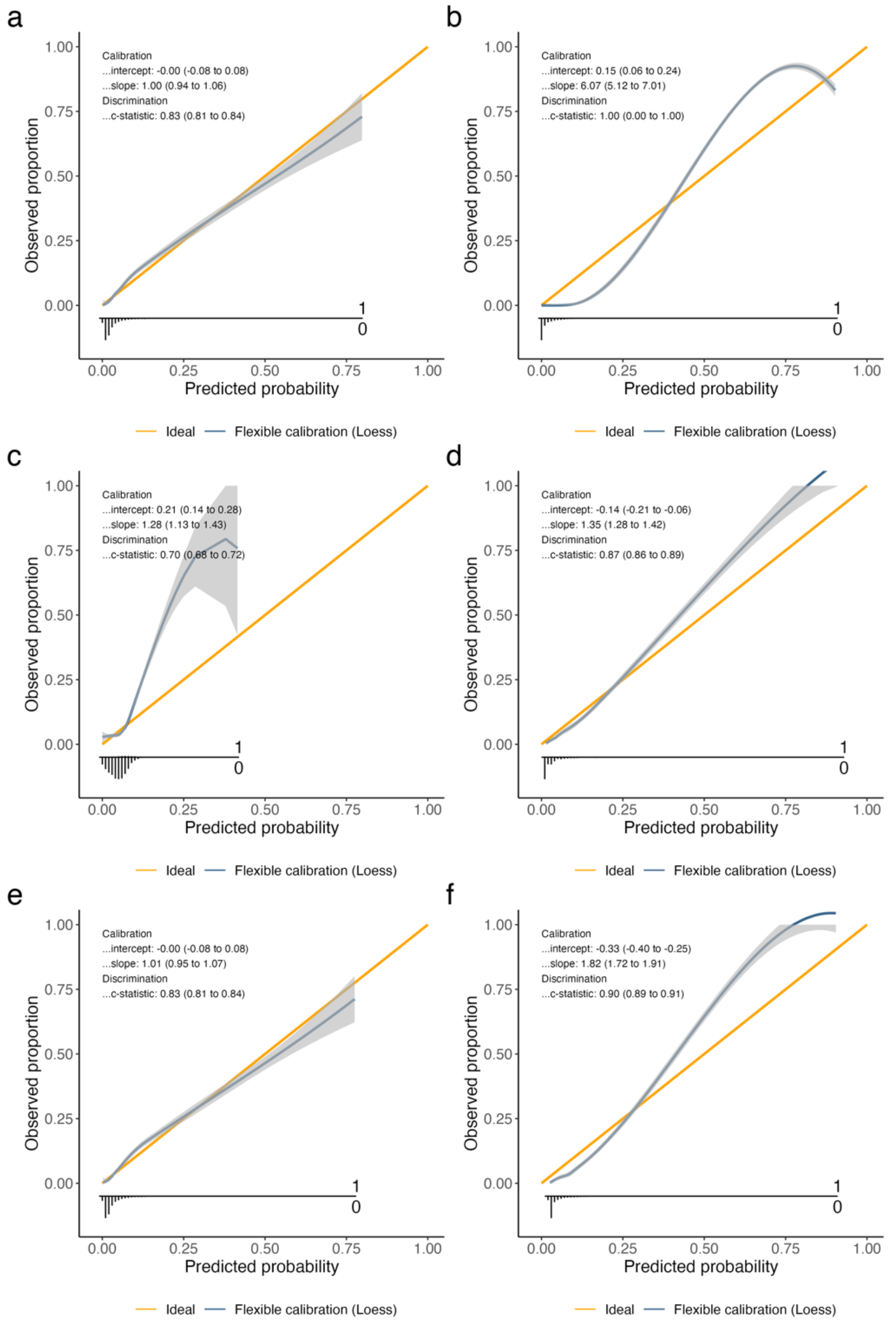

Figure S4.6.1. Internal validation on the combined IST and CAST training dataset: calibration plot of predicted outcome in the treatment group. The orange line indicates ideal calibration. The outcome variable is death at 14 days for IST and death at 4 weeks for CAST. **a**, T-learner Logistic Regression. **b**, T-learner Random Forest. **c**, T-learner Support Vector Machine. **d**, T-learner XGBoost. **e**, S-learner Logistic Regression. **f**, S-learner XGBoost. Abbreviations: IST: the International Stroke Trial, CAST: the Chinese Acute Stroke Trials.

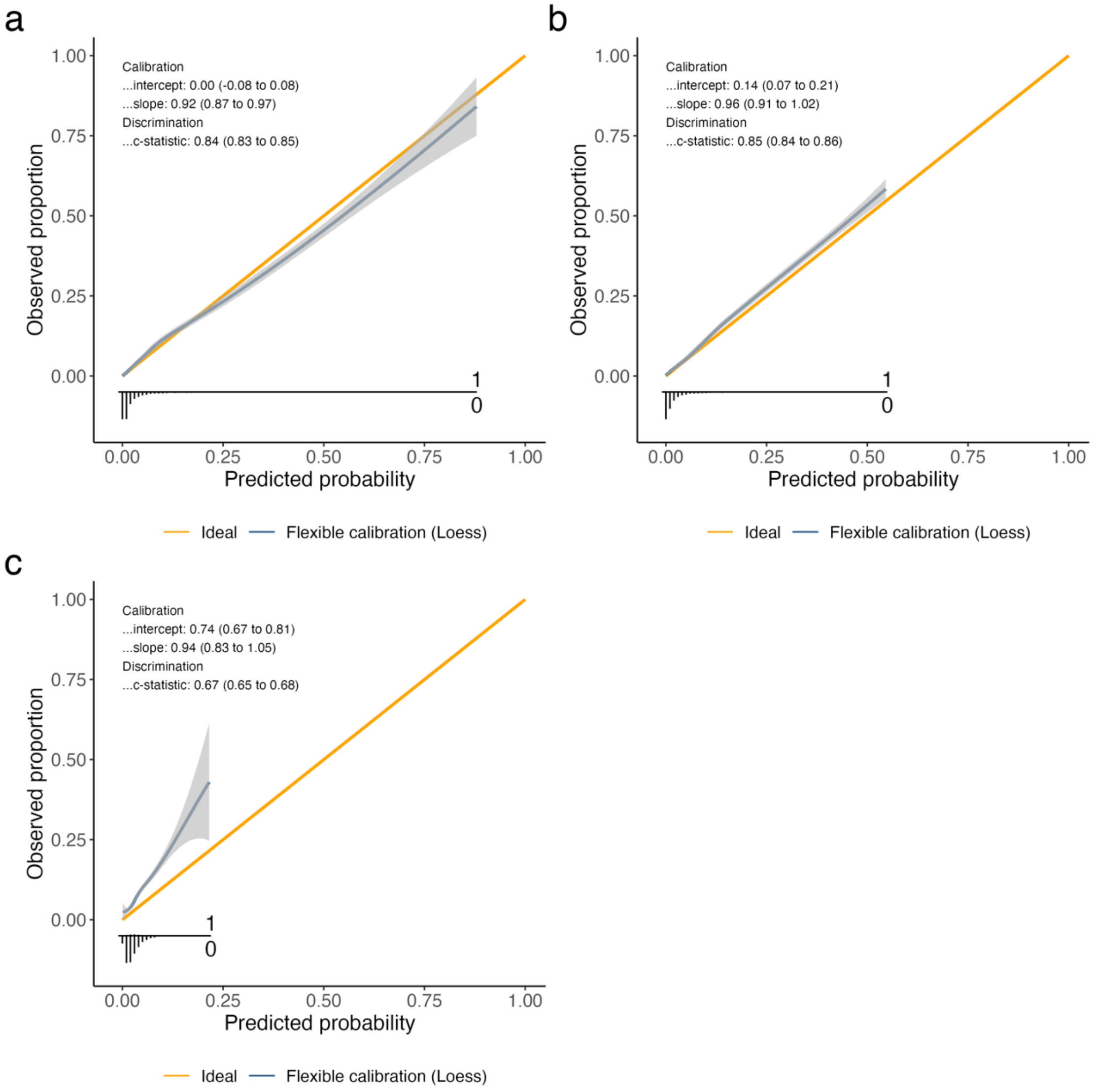

Figure S4.6.2. Internal validation on the combined IST and CAST training dataset: calibration plot of predicted outcome in the treatment group. The orange line indicates ideal calibration. The outcome variable is death at 14 days for IST and death at 4 weeks for CAST. **a**, S-learner BART. **b**, CVAE. **c**, GANITE. Abbreviations: IST: the International Stroke Trial, CAST: the Chinese Acute Stroke Trial.

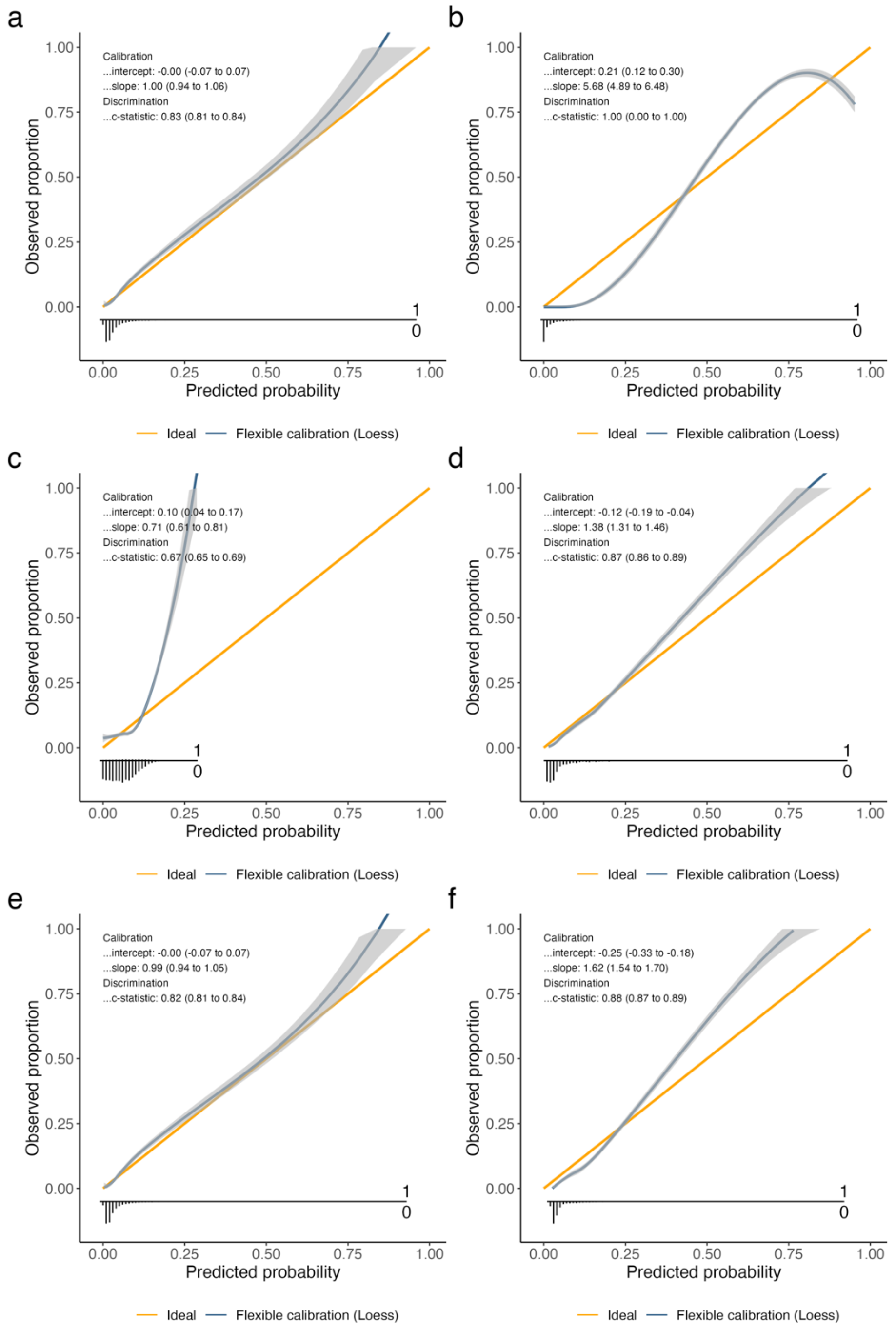

Figure S4.7.1. Internal validation on the combined IST and CAST training dataset: calibration plot of predicted outcome in the control group. The orange line indicates ideal calibration. The outcome variable is death at 14 days for IST and death at 4 weeks for CAST. **a**, T-learner Logistic Regression. **b**, T-learner Random Forest. **c**, T-learner Support Vector Machine. **d**, T-learner XGBoost. **e**, S-learner Logistic Regression. **f**, S-learner XGBoost. Abbreviations: IST: the International Stroke Trial, CAST: the Chinese Acute Stroke Trial.

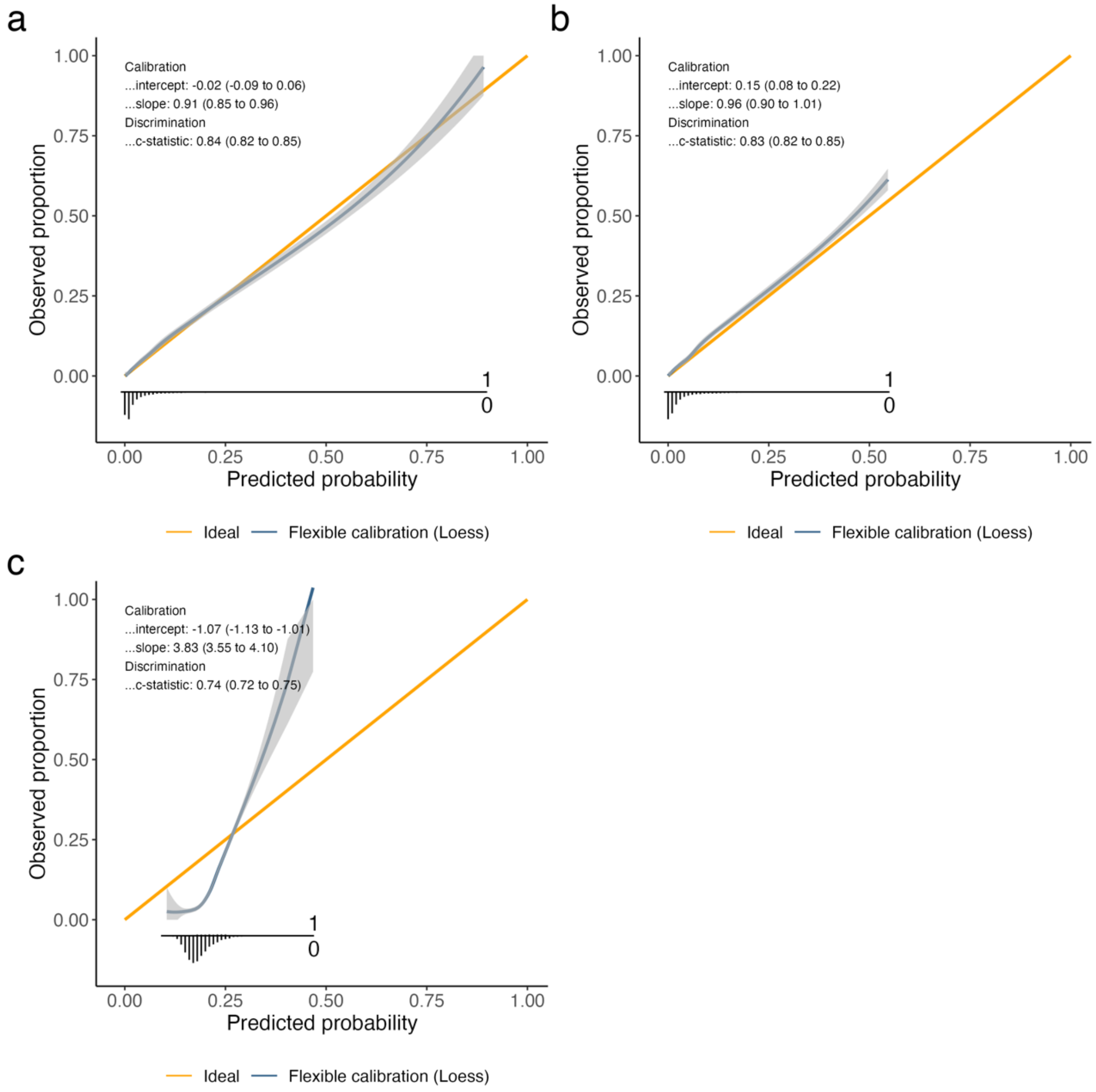

Figure S4.7.2. Internal validation on the combined IST and CAST training dataset: calibration plot of predicted outcome in the control group. The orange line indicates ideal calibration. The outcome variable is death at 14 days for IST and death at 4 weeks for CAST. **a**, S-learner BART. **b**, CVAE. **c**, GANITE. Abbreviations: IST: the International Stroke Trial, CAST: the Chinese Acute Stroke Trial.

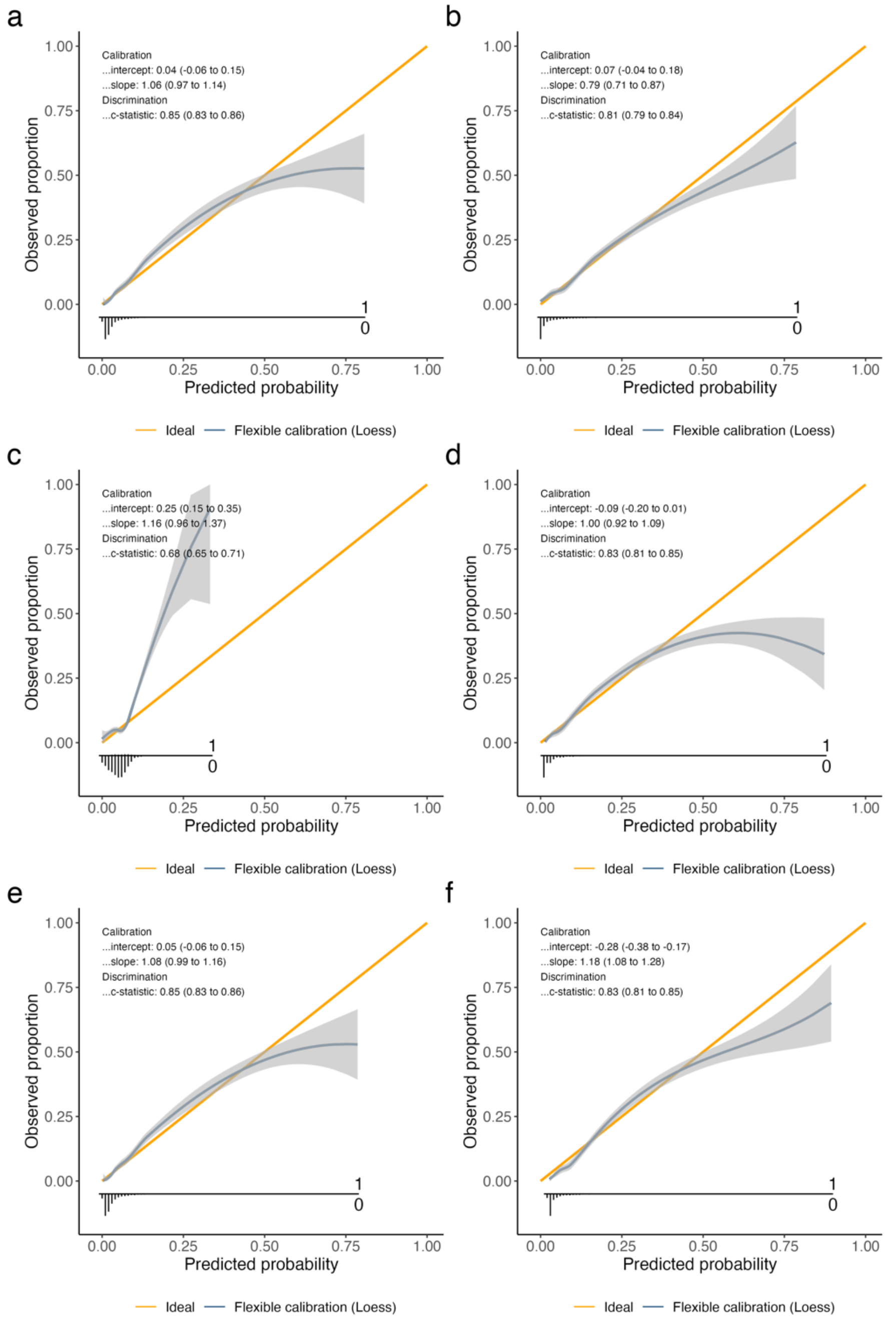

Figure S4.8.1. Internal validation on the combined IST and CAST test dataset: calibration plot of predicted outcome in the treatment group. The orange line indicates ideal calibration. The outcome variable is death at 14 days for IST and death at 4 weeks for CAST. **a**, T-learner Logistic Regression. **b**, T-learner Random Forest. **c**, T-learner Support Vector Machine. **d**, T-learner XGBoost. **e**, S-learner Logistic Regression. **f**, S-learner XGBoost. Abbreviations: IST: the International Stroke Trial, CAST: the Chinese Acute Stroke Trial.

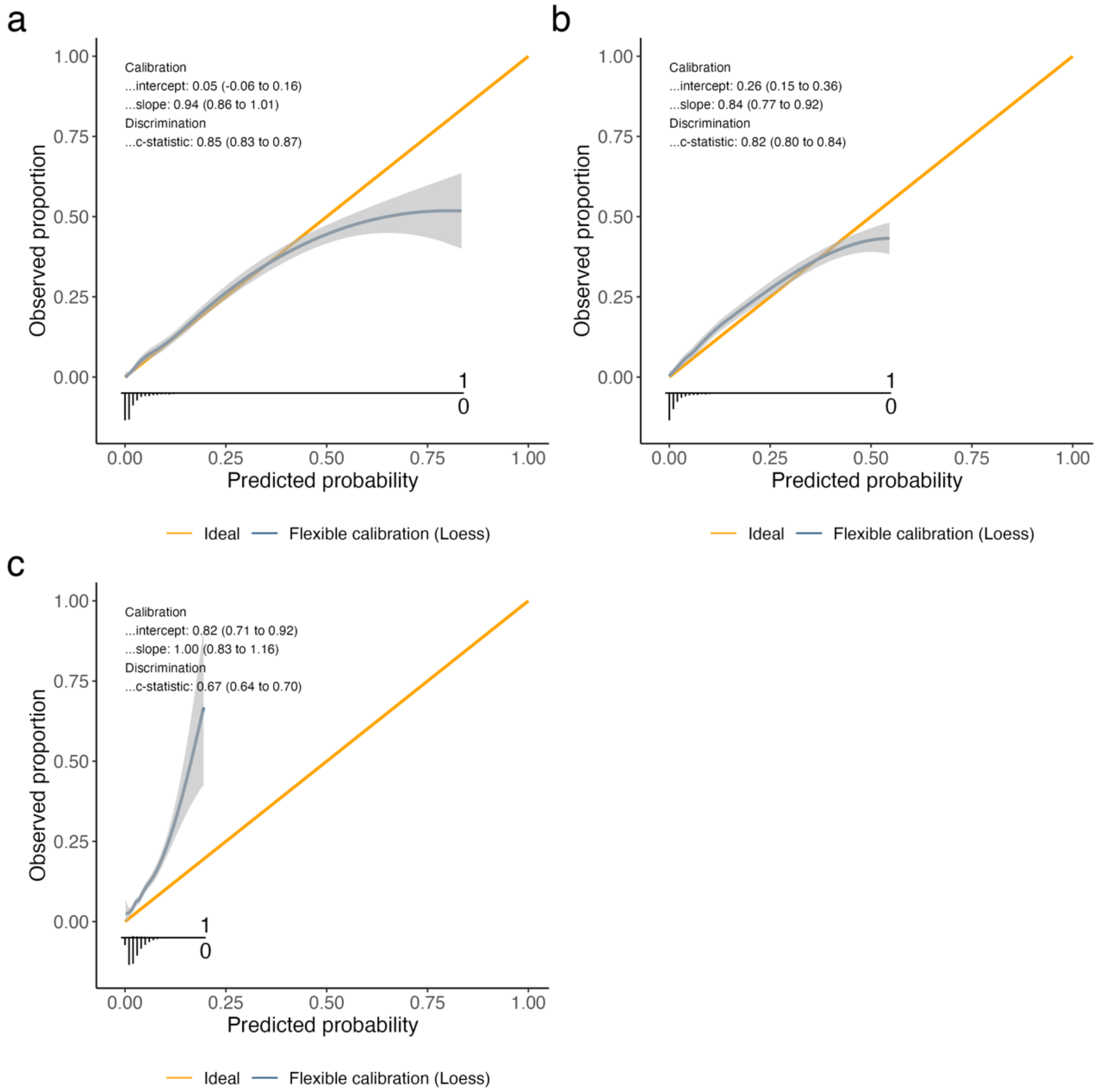

Figure S4.8.2. Internal validation on the combined IST and CAST test dataset: calibration plot of predicted outcome in the treatment group. The orange line indicates ideal calibration. The outcome variable is death at 14 days for IST and death at 4 weeks for CAST. **a**, S-learner BART. **b**, CVAE. **c**, GANITE. Abbreviations: IST: the International Stroke Trial, CAST: the Chinese Acute Stroke Trial.

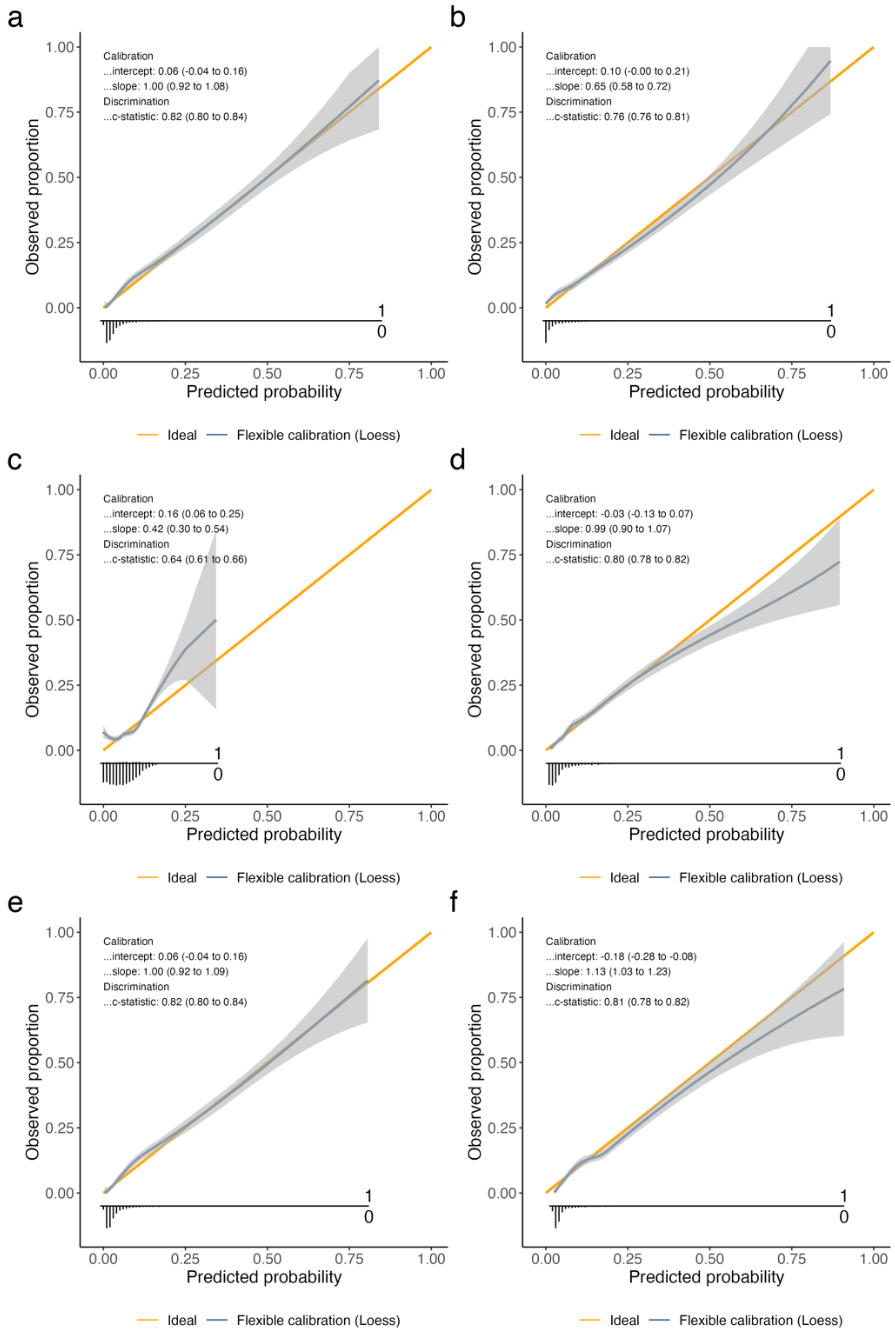

Figure S4.9.1. Internal validation on the combined IST and CAST test dataset: calibration plot of predicted outcome in the control group. The orange line indicates ideal calibration. The outcome variable is death at 14 days for IST and death at 4 weeks for CAST. **a**, T-learner Logistic Regression. **b**, T-learner Random Forest. **c**, T-learner Support Vector Machine. **d**, T-learner XGBoost. **e**, S-learner Logistic Regression. **f**, S-learner XGBoost. Abbreviations: IST: the International Stroke Trial, CAST: the Chinese Acute Stroke Trial.

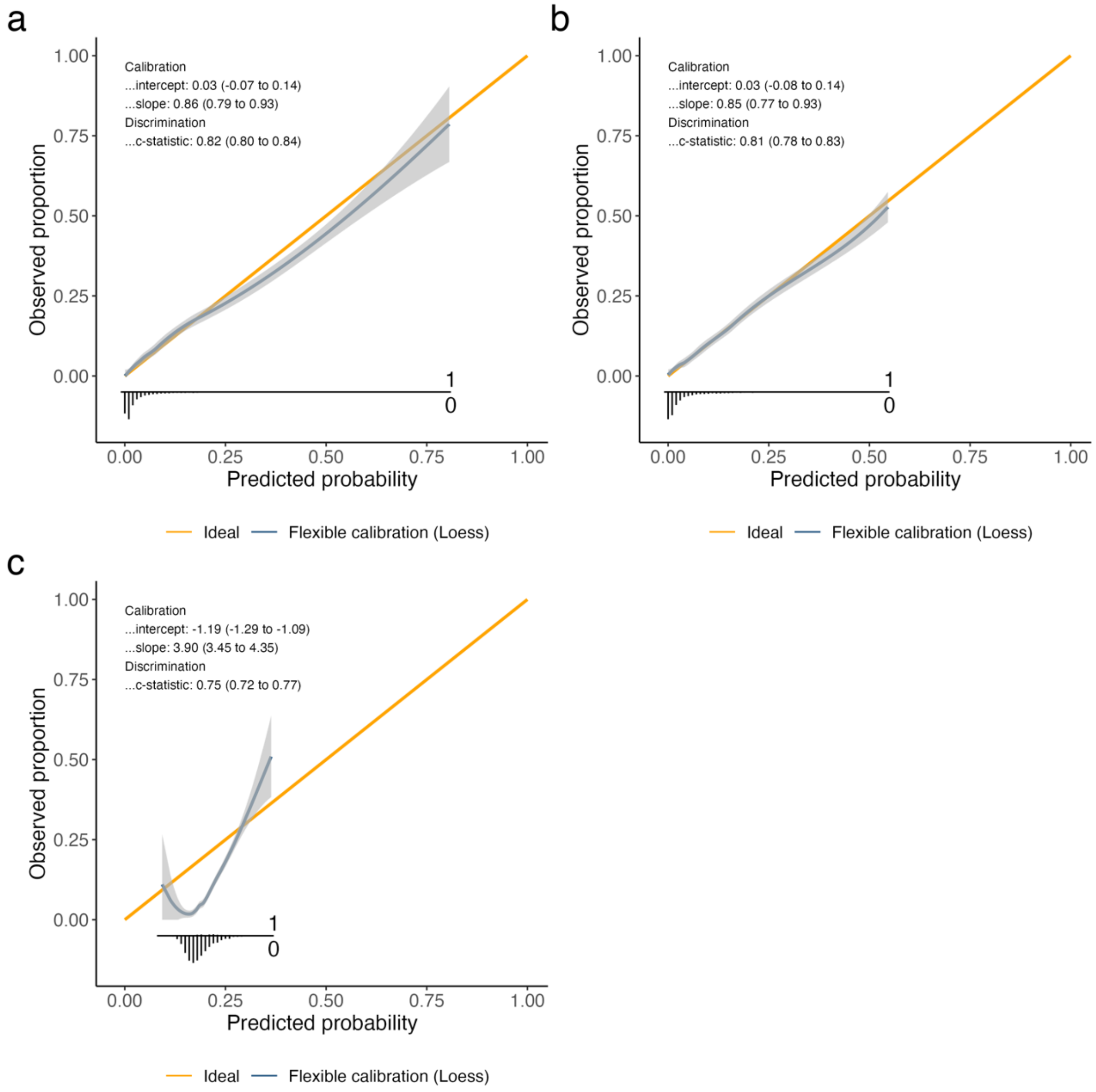

Figure S4.9.2. Internal validation on the combined IST and CAST test dataset: calibration plot of predicted outcome in the control group. The orange line indicates ideal calibration. The outcome variable is death at 14 days for IST and death at 4 weeks for CAST. **a**, S-learner BART. **b**, CVAE. **c**, GANITE. Abbreviations: IST: the International Stroke Trial, CAST: the Chinese Acute Stroke Trial.

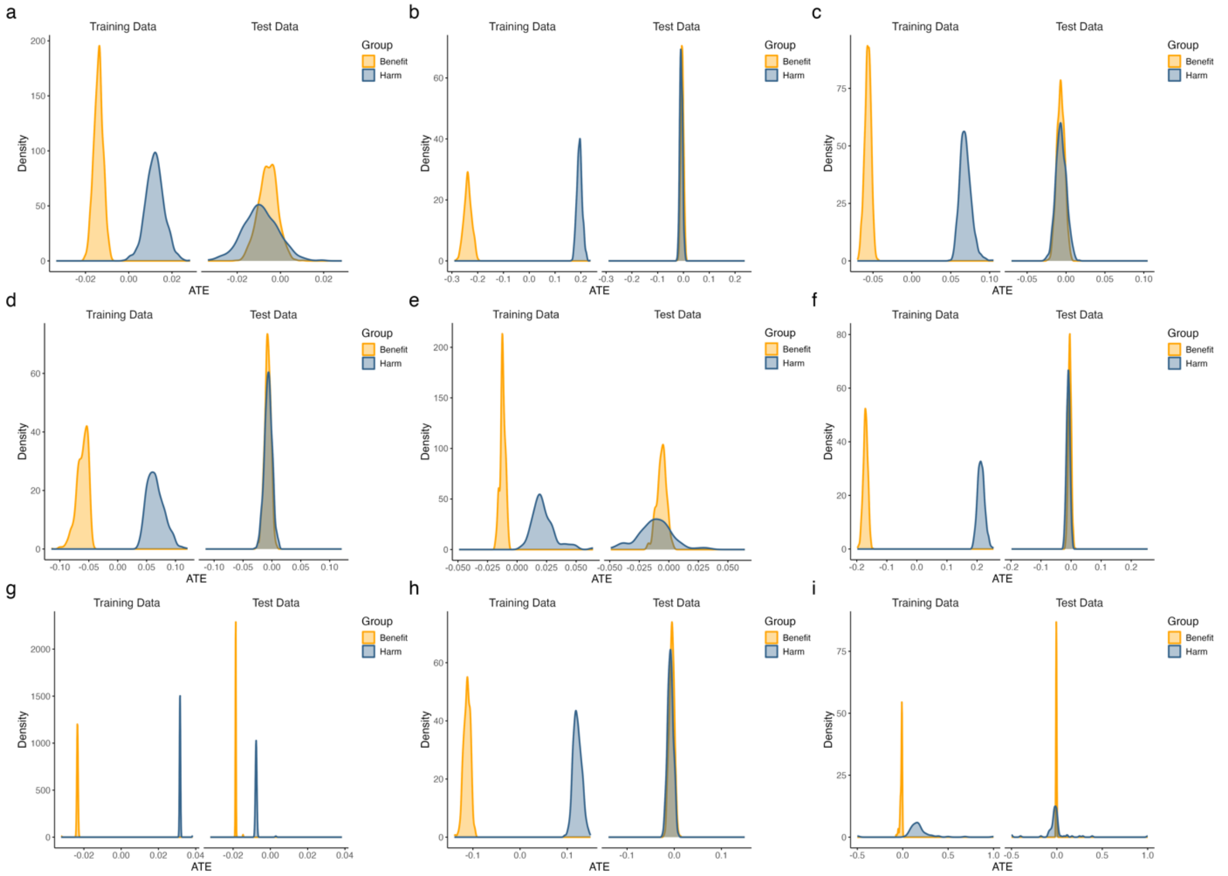

Figure S4.10.1. Internal validation on the combined IST and CAST dataset: density comparative analysis of causal machine learning-based individualized treatment effects. Density plots depict the distributions of ATE in training data and test data stratified by benefit (orange) and harm (blue) groups. The distribution of ATE comes from 1000 (**a, b, c, d, f, h**) or 100 (**e, g**) random train-test splits experiments. Benefit means negative ITE values and harm means positive ITE values. The outcome variable is death at 14 days for IST and death at 4 weeks for CAST. **a**, T-learner Logistic Regression. **b**, T-learner Random Forest. **c**, T-learner XGBoost. **d**, S-learner XGBoost. **e**, S-learner BART. **f**, X-learner Random Forest. **g**, X-learner BART. **h**, DR-learner Random Forest. **i**, Causal Forest. Abbreviations: IST: the International Stroke Trial, CAST: the Chinese Acute Stroke Trial, ITE: individualized treatment effect, ATE: average treatment effect.

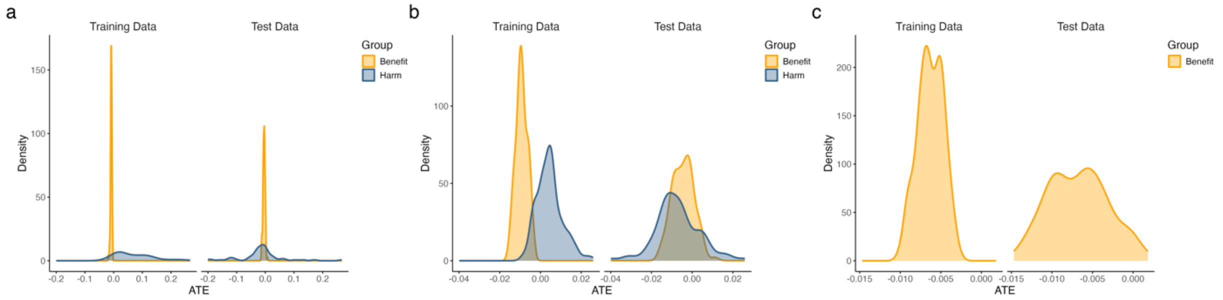

Figure S4.10.2. Internal validation on the combined IST and CAST dataset: density comparative analysis of causal machine learning-based individualized treatment effects. Density plots depict the distributions of ATE in training data and test data stratified by benefit (orange) and harm (blue) groups. The distribution of ATE comes from 100 random train-test splits experiments. Benefit means negative ITE values and harm means positive ITE values. The outcome variable is death at 14 days for IST and death at 4 weeks for CAST. **a**, Bayesian Causal Forest. **b**, CVAE. **c**, GANITE. Abbreviations: IST: the International Stroke Trial, CAST: the Chinese Acute Stroke Trial, ITE: individualized treatment effect, ATE: average treatment effect.

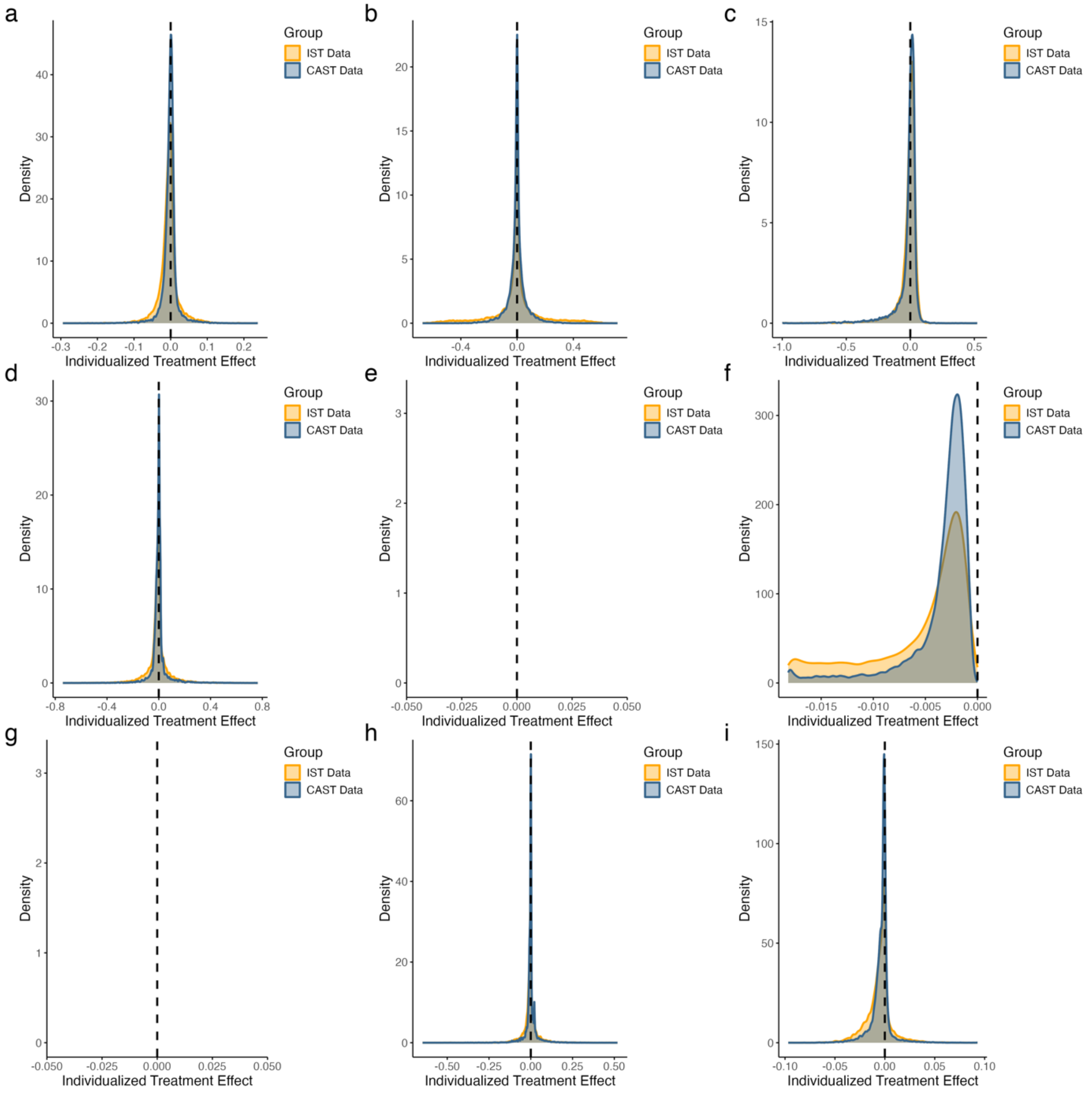

Figure S5.1.1. External validation with IST as training data and CAST as test data: density plots of causal machine learning-based individualized treatment effects. Orange represents the training data, and blue indicates the test data. The outcome variable is death at 14 days for IST and death at 4 weeks for CAST. **a**, T-learner Logistic Regression. **b**, T-learner Random Forest. **c**, T-learner Support Vector Machine. **d**, T-learner XGBoost. **e**, T-learner Penalized Logistic Regression. **f**, S-learner Logistic Regression. **g**, S-learner Penalized Logistic Regression. **h**, S-learner XGBoost. **i**, S-learner BART. Abbreviations: IST: the International Stroke Trial, CAST: the Chinese Acute Stroke Trial, ITE: individualized treatment effect.

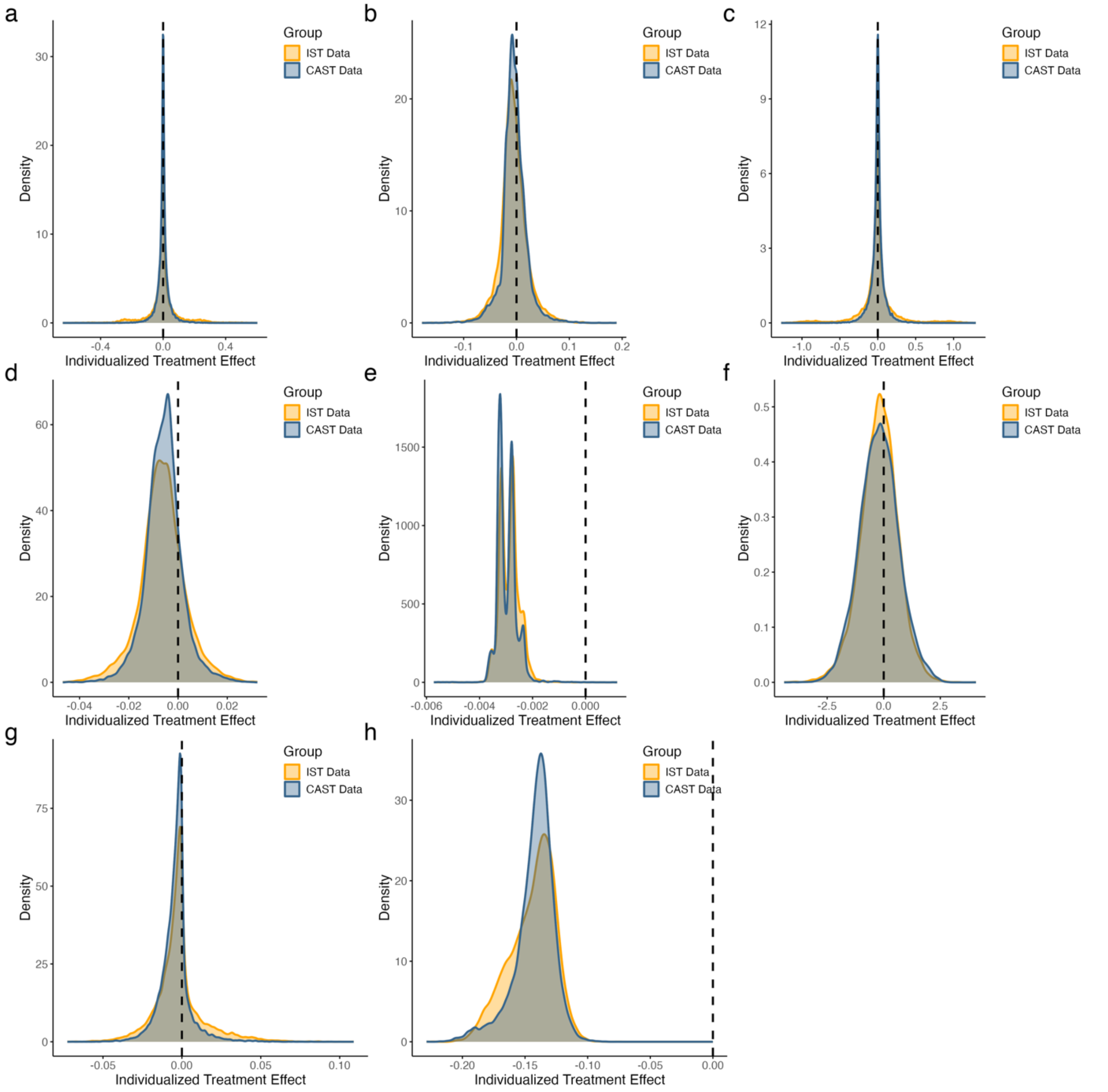

Figure S5.1.2. External validation with IST as training data and CAST as test data: density plots of causal machine learning-based individualized treatment effects. Orange represents the training data, and blue indicates the test data. The outcome variable is death at 14 days for IST and death at 4 weeks for CAST. **a**, X-learner Random Forest. **b**, X-learner BART. **c**, DR-learner Random Forest. **d**, Causal Forest. **e**, Bayesian Causal Forest. **f**, Model-based Recursive Partitioning. **g**, CVAE. **h**, GANITE. Abbreviations: IST: the International Stroke Trial, CAST: the Chinese Acute Stroke Trial, ITE: individualized treatment effect.

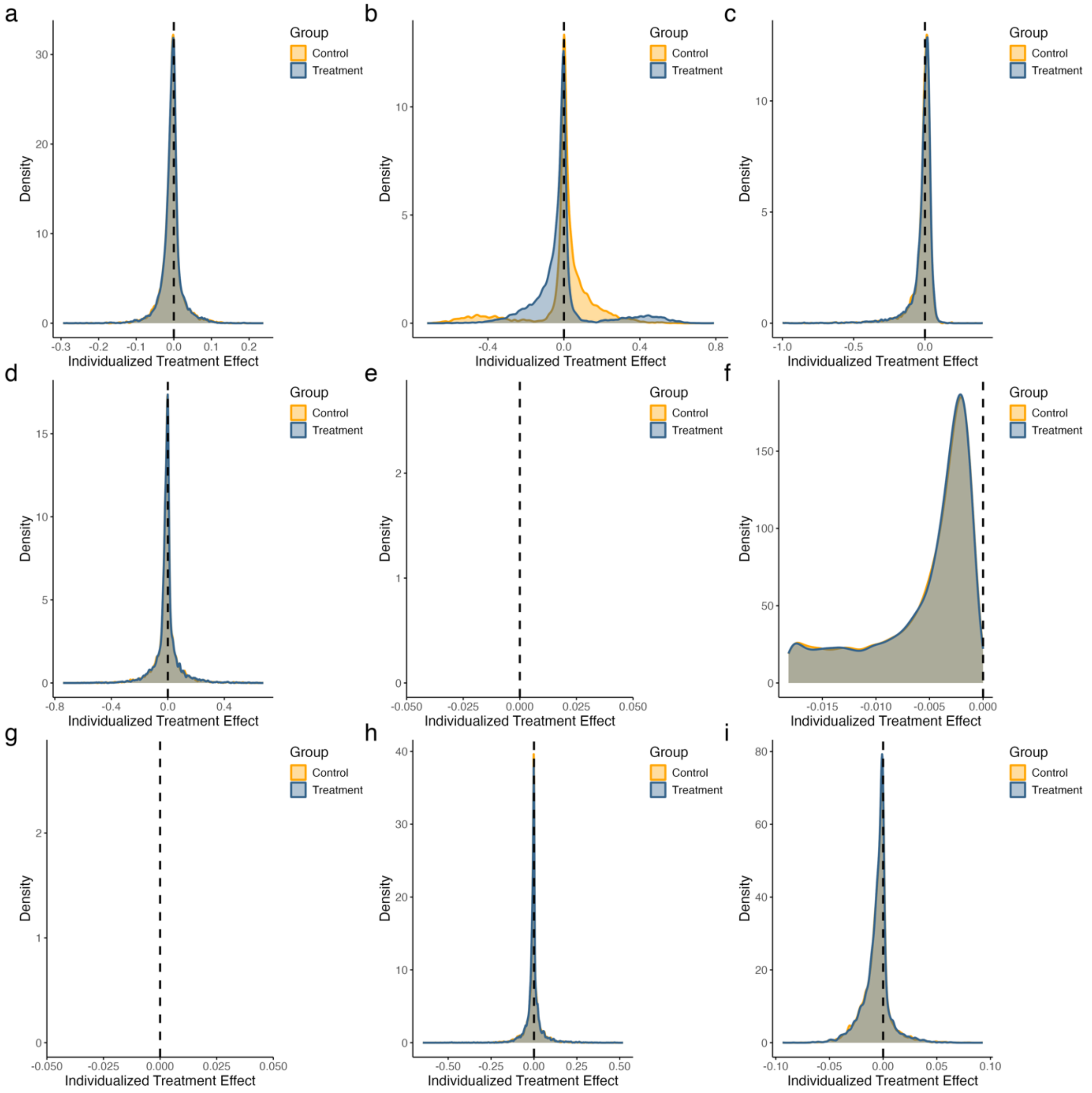

Figure S5.2.1. IST as training dataset: density plots of causal machine learning-based individualized treatment effects. Orange represents the control group, and blue indicates the treatment group. The outcome variable is death at 14 days for IST and death at 4 weeks for CAST. **a**, T-learner Logistic Regression. **b**, T-learner Random Forest. **c**, T-learner Support Vector Machine. **d**, T-learner XGBoost. **e**, T-learner Penalized Logistic Regression. **f**, S-learner Logistic Regression. **g**, S-learner Penalized Logistic Regression. **h**, S-learner XGBoost. **i**, S-learner BART. Abbreviations: IST: the International Stroke Trial, CAST: the Chinese Acute Stroke Trial, ITE: individualized treatment effect.

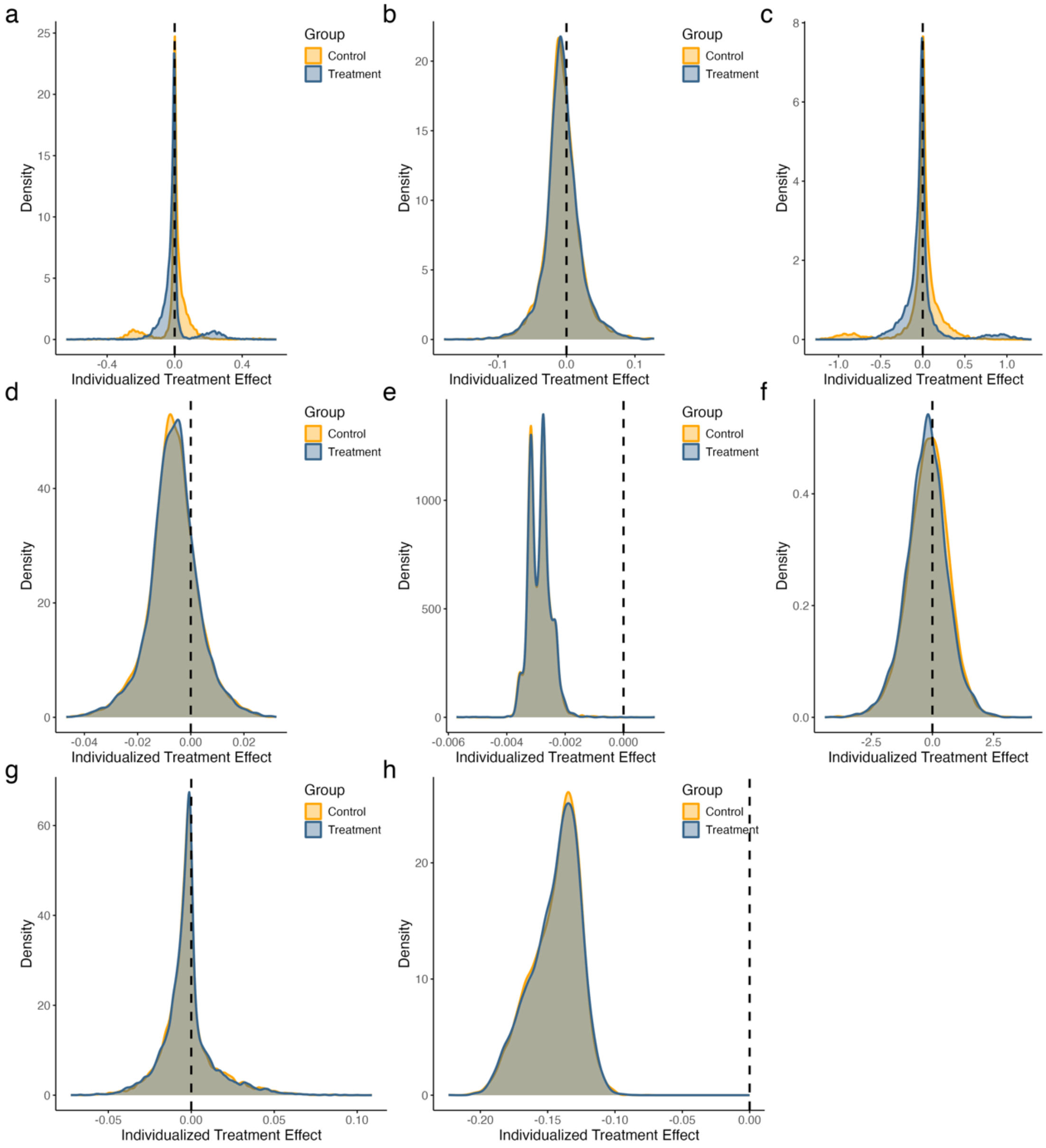

Figure S5.2.2. IST as training dataset: density plots of causal machine learning-based individualized treatment effects. Orange represents the control group, and blue indicates the treatment group. The outcome variable is death at 14 days for IST and death at 4 weeks for CAST. **a**, X-learner Random Forest. **b**, X-learner BART. **c**, DR-learner Random Forest. **d**, Causal Forest. **e**, Bayesian Causal Forest. **f**, Model-based Recursive Partitioning. **g**, CVAE. **h**, GANITE. Abbreviations: IST: the International Stroke Trial, CAST: the Chinese Acute Stroke Trial, ITE: individualized treatment effect.

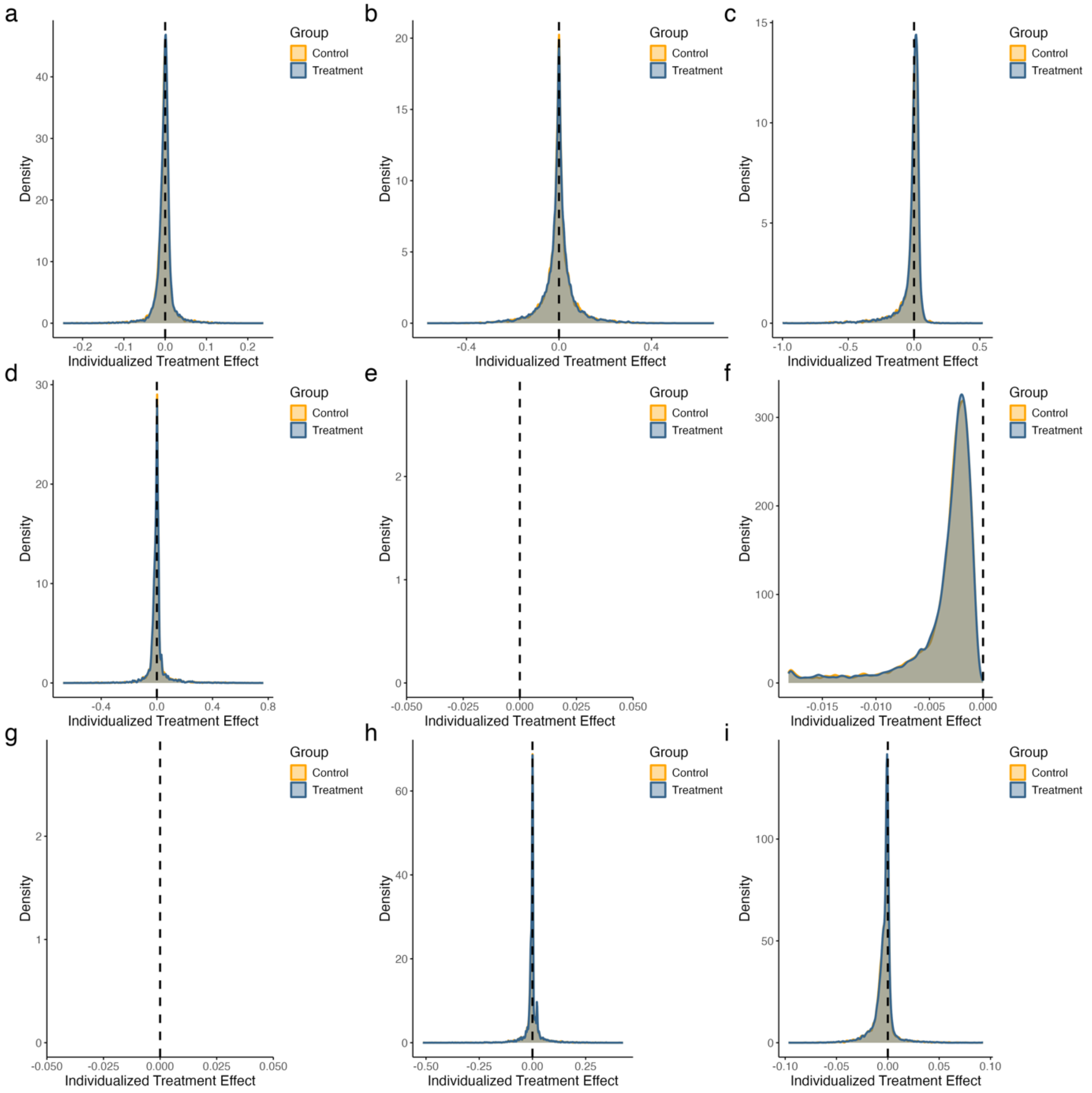

Figure S5.3.1. CAST as test dataset: density plots of causal machine learning-based individualized treatment effects. Orange represents the control group, and blue indicates the treatment group. The outcome variable is death at 14 days for IST and death at 4 weeks for CAST. **a**, T-learner Logistic Regression. **b**, T-learner Random Forest. **c**, T-learner Support Vector Machine. **d**, T-learner XGBoost. **e**, T-learner Penalized Logistic Regression. **f**, S-learner Logistic Regression. **g**, S-learner Penalized Logistic Regression. **h**, S-learner XGBoost. **i**, S-learner BART. Abbreviations: IST: the International Stroke Trial, CAST: the Chinese Acute Stroke Trial, ITE: individualized treatment effect.

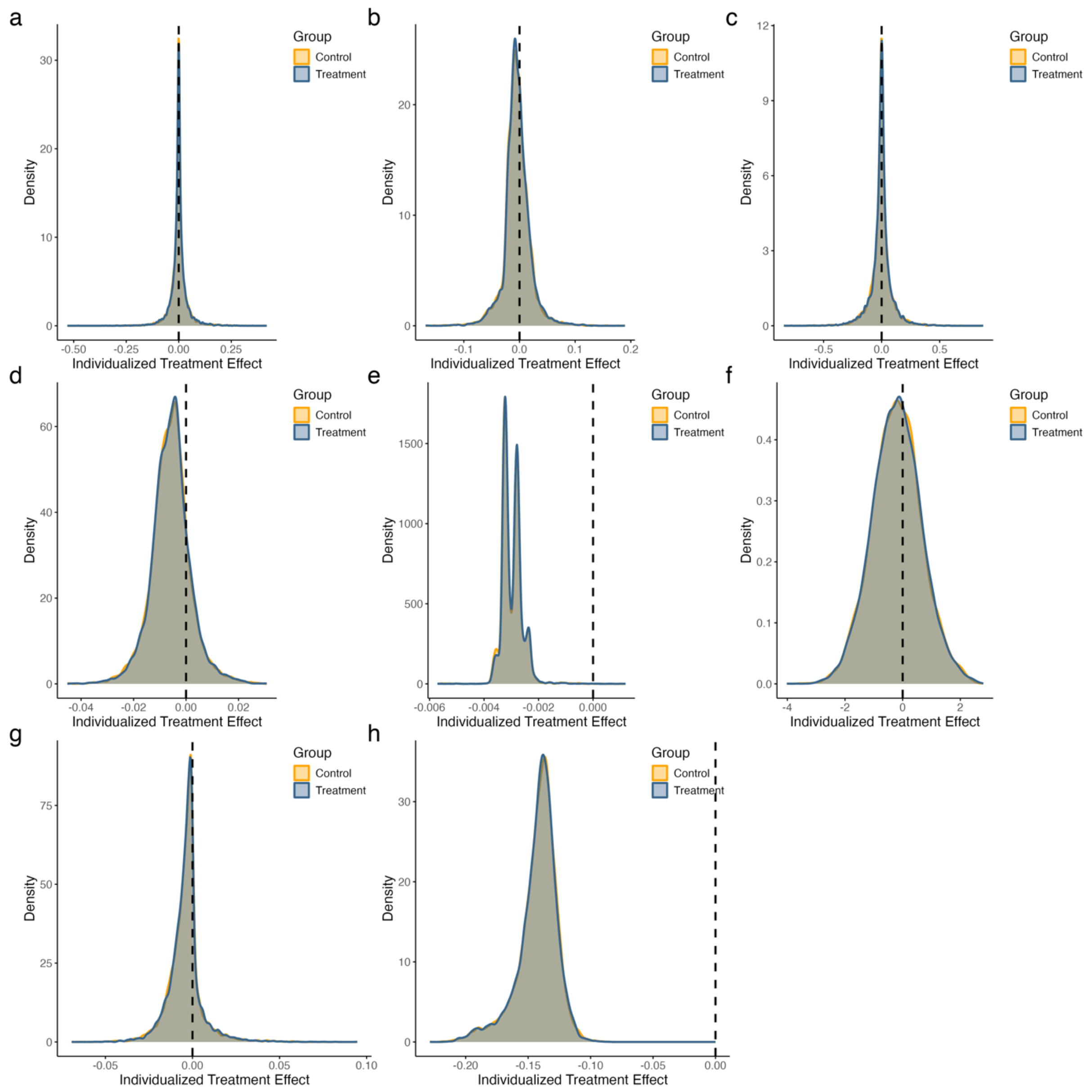

Figure S5.3.2. CAST as test dataset: density plots of causal machine learning-based individualized treatment effects. Orange represents the control group, and blue indicates the treatment group. The outcome variable is death at 14 days for IST and death at 4 weeks for CAST. **a**, X-learner Random Forest. **b**, X-learner BART. **c**, DR-learner Random Forest. **d**, Causal Forest. **e**, Bayesian Causal Forest. **f**, Model-based Recursive Partitioning. **g**, CVAE. **h**, GANITE. Abbreviations: IST: the International Stroke Trial, CAST: the Chinese Acute Stroke Trial, ITE: individualized treatment effect.

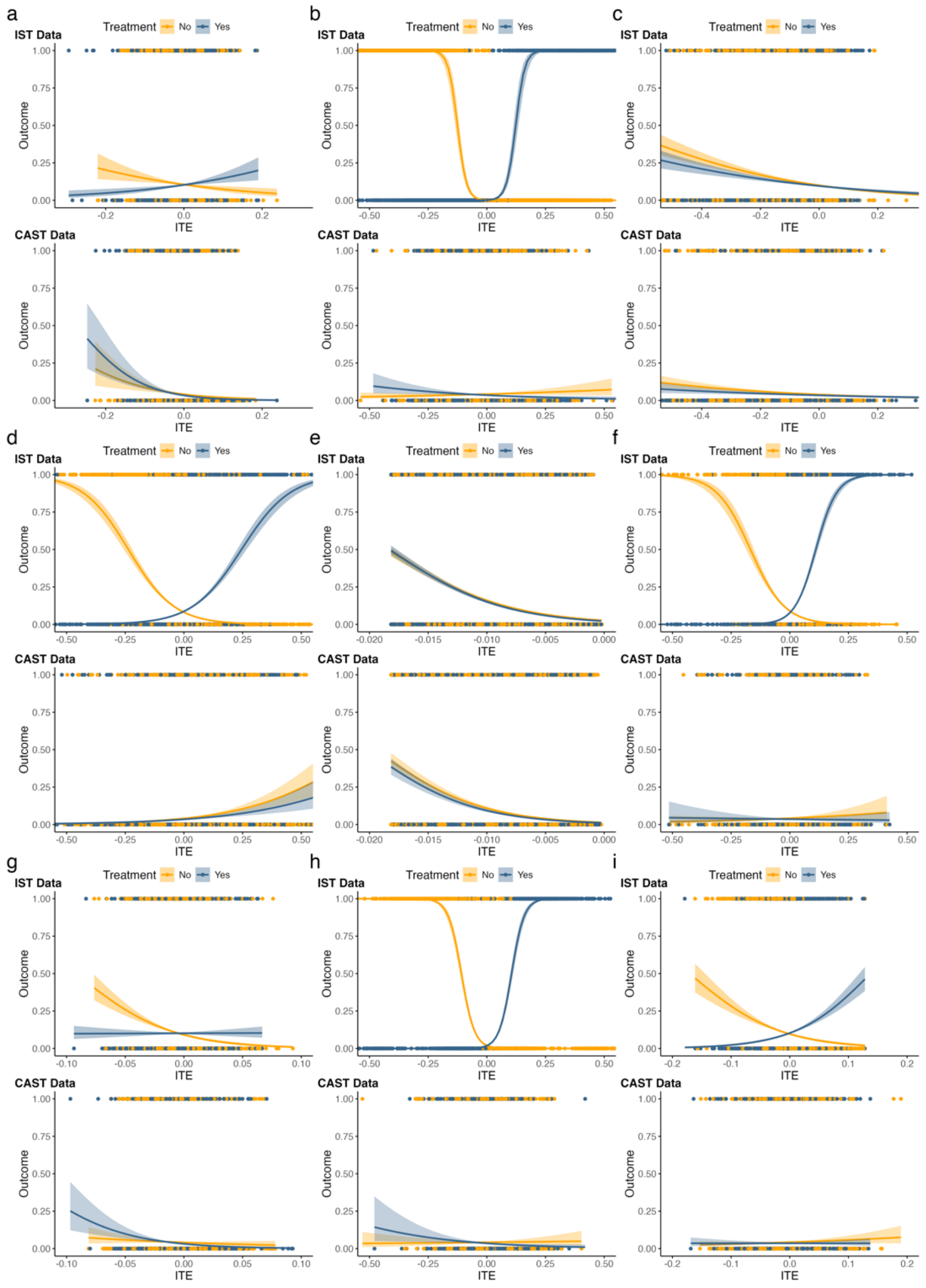

Figure S5.4.1. External validation with IST as training data and CAST as test data: outcome-ITE comparative analysis. Dot plots and line plots depict the true and fitted patient outcomes against estimated ITE values between training data and test data. Orange represents the control group, and blue indicates the treatment group. The outcome variable is death at 14 days for IST and death at 4 weeks for CAST. **a**, T-learner Logistic Regression. **b**, T-learner Random Forest. **c**, T-learner Support Vector Machine. **d**, T-learner XGBoost. **e**, S-learner Logistic Regression. **f**, S-learner XGBoost. **g**, S-learner BART. **h**, X-learner Random Forest. **i**, X-learner BART. Abbreviations: IST: the International Stroke Trial, CAST: the Chinese Acute Stroke Trial, ITE: individualized treatment effect.

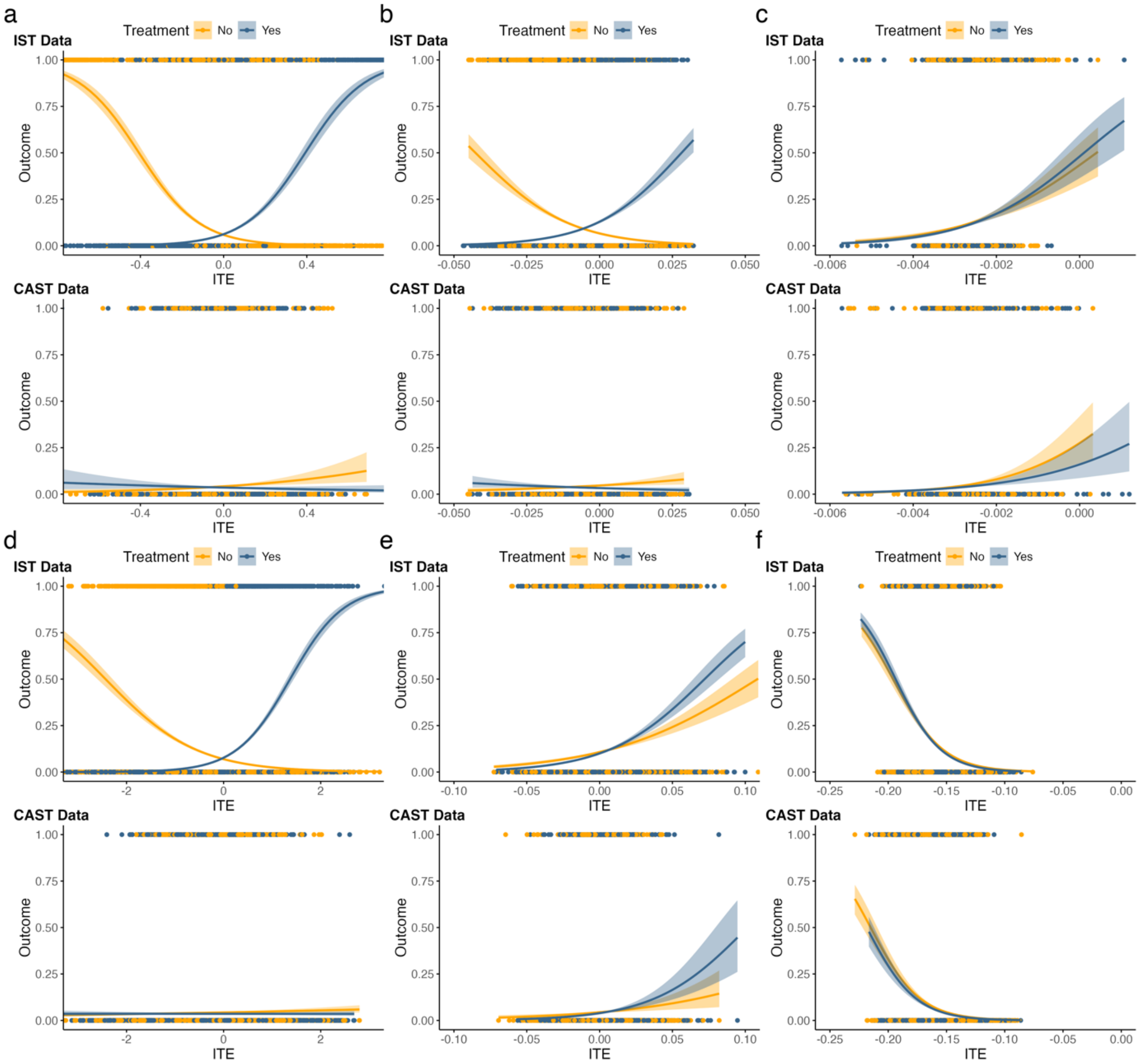

Figure S5.4.2. External validation with IST as training data and CAST as test data: outcome-ITE comparative analysis. Dot plots and line plots depict the true and fitted patient outcomes against estimated ITE values between training data and test data. Orange represents the control group, and blue indicates the treatment group. The outcome variable is death at 14 days for IST and death at 4 weeks for CAST. **a**, DR-learner Random Forest. **b**, Causal Forest. **c**, Bayesian Causal Forest. **d**, Model-based Recursive Partitioning. **e**, CVAE. **f**, GANITE. Abbreviations: IST: the International Stroke Trial, CAST: the Chinese Acute Stroke Trial, ITE: individualized treatment effect.

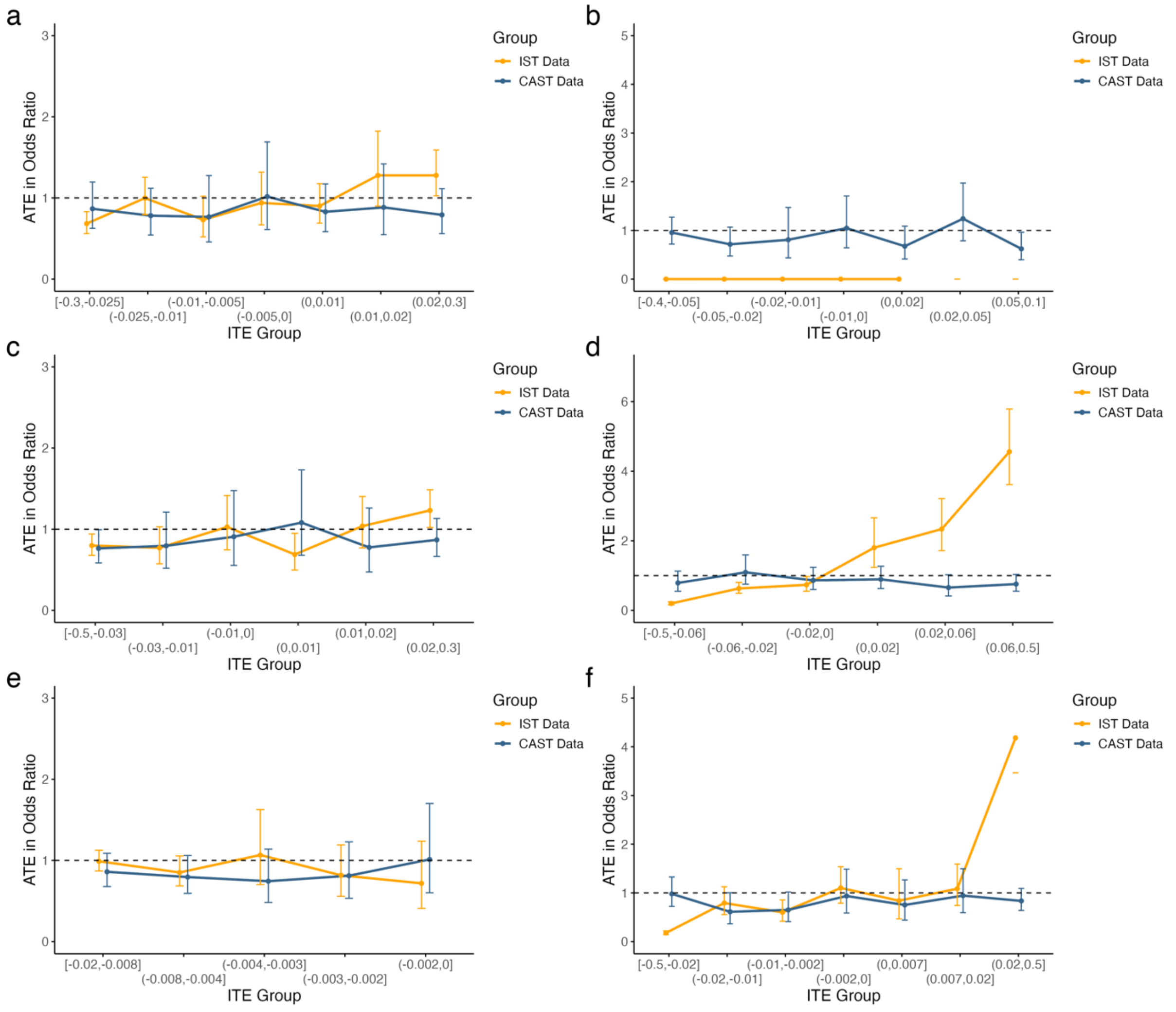

Figure S5.5.1. External validation with IST as training data and CAST as test data: ATE-ITE comparative analysis. Line plots depict ATE in risk ratio within different ITE subgroups and provide the confidence intervals at 95% level. Orange represents the training data, and blue indicates the test data. The horizontal dashed line at 1.0 means no treatment effects. The outcome variable is death at 14 days for IST and death at 4 weeks for CAST. **a**, T-learner Logistic Regression. **b**, T-learner Random Forest. **c**, T-learner Support Vector Machine. **d**, T-learner XGBoost. **e**, S-learner Logistic Regression. **f**, S-learner XGBoost. Abbreviations: IST: the International Stroke Trial, CAST: the Chinese Acute Stroke Trial, ITE: individualized treatment effect, ATE: average treatment effect.

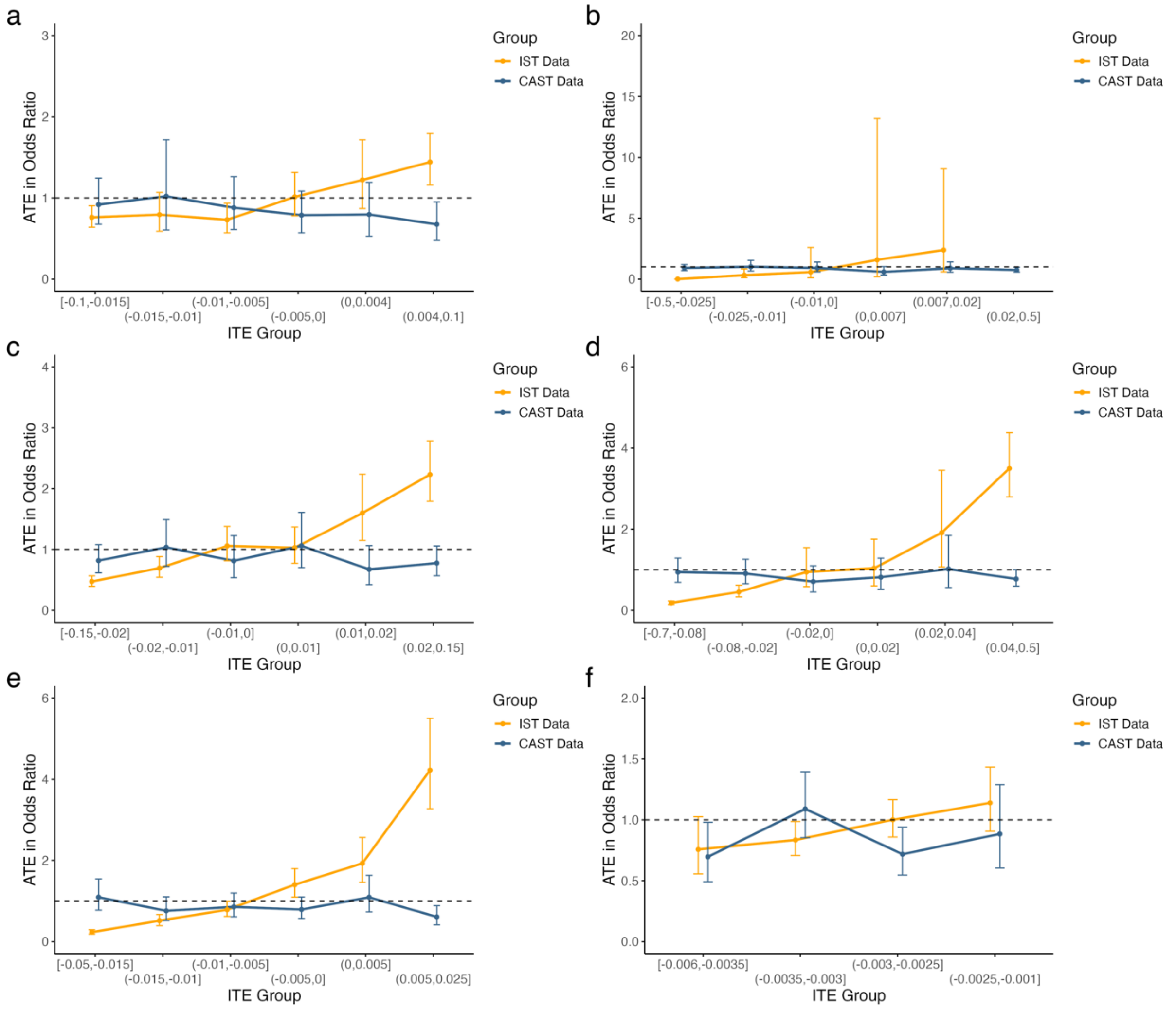

Figure S5.5.2. External validation with IST as training data and CAST as test data: ATE-ITE comparative analysis. Line plots depict ATE in risk ratio within different ITE subgroups and provide the confidence intervals at 95% level. Orange represents the training data, and blue indicates the test data. The horizontal dashed line at 1.0 means no treatment effects. The outcome variable is death at 14 days for IST and death at 4 weeks for CAST. **a**, S-learner BART. **b**, X-learner Random Forest. **c**, X-learner BART. **d**, DR-learner Random Forest. **e**, Causal Forest. **f**, Bayesian Causal Forest. Abbreviations: IST: the International Stroke Trial, CAST: the Chinese Acute Stroke Trial, ITE: individualized treatment effect, ATE: average treatment effect.

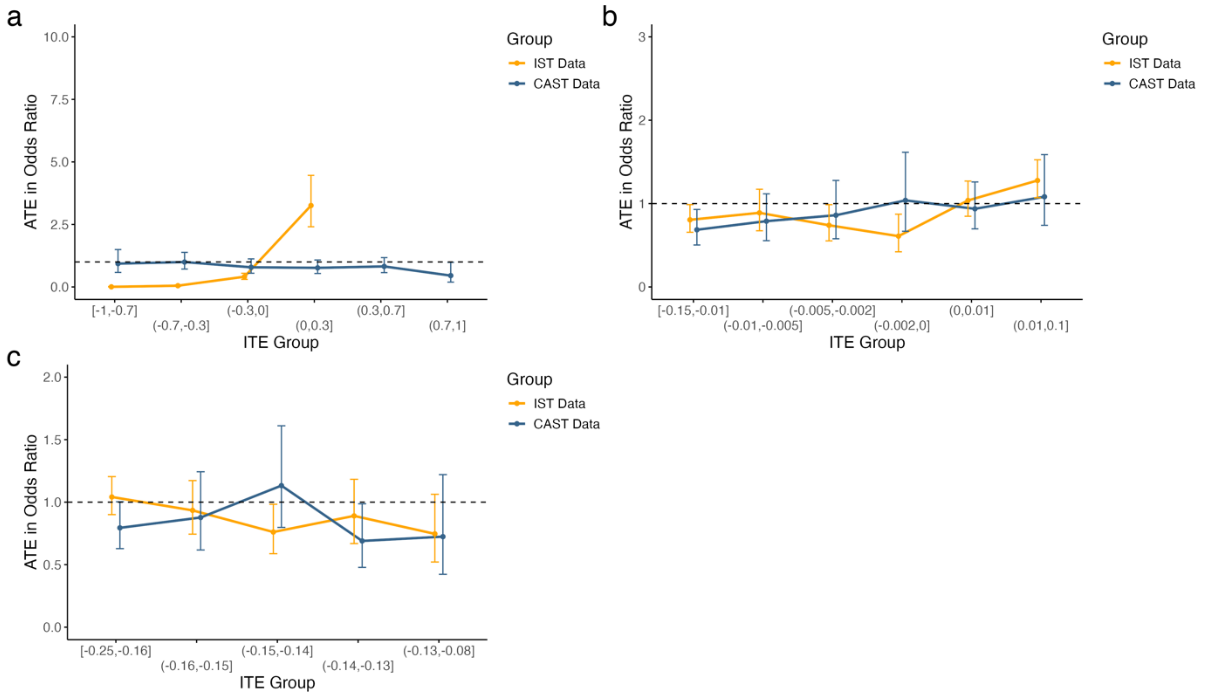

Figure S5.5.2. External validation with IST as training data and CAST as test data: ATE-ITE comparative analysis. Line plots depict ATE in risk ratio within different ITE subgroups and provide the confidence intervals at 95% level. Orange represents the training data, and blue indicates the test data. The horizontal dashed line at 1.0 means no treatment effects. The outcome variable is death at 14 days for IST and death at 4 weeks for CAST. **a**, Model-based Recursive Partitioning. **b**, CVAE. **c**, GANITE. Abbreviations: IST: the International Stroke Trial, CAST: the Chinese Acute Stroke Trial, ITE: individualized treatment effect, ATE: average treatment effect.

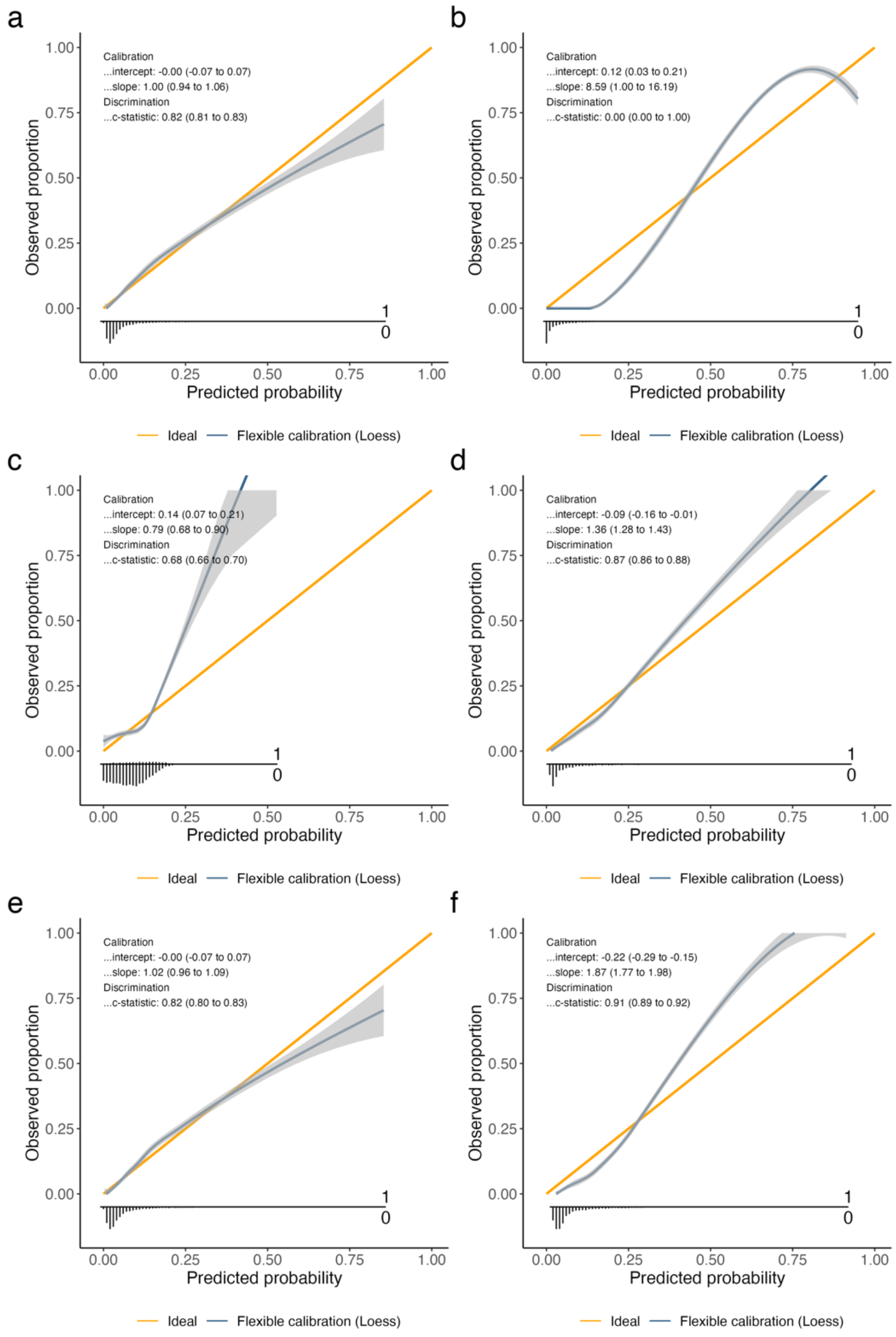

Figure S5.6.1. IST as training dataset: calibration plot of predicted outcome in the treatment group. The orange line indicates ideal calibration. The outcome variable is death at 14 days for IST and death at 4 weeks for CAST. **a**, T-learner Logistic Regression. **b**, T-learner Random Forest. **c**, T-learner Support Vector Machine. **d**, T-learner XGBoost. **e**, S-learner Logistic Regression. **f**, S-learner XGBoost. Abbreviations: IST: the International Stroke Trial.

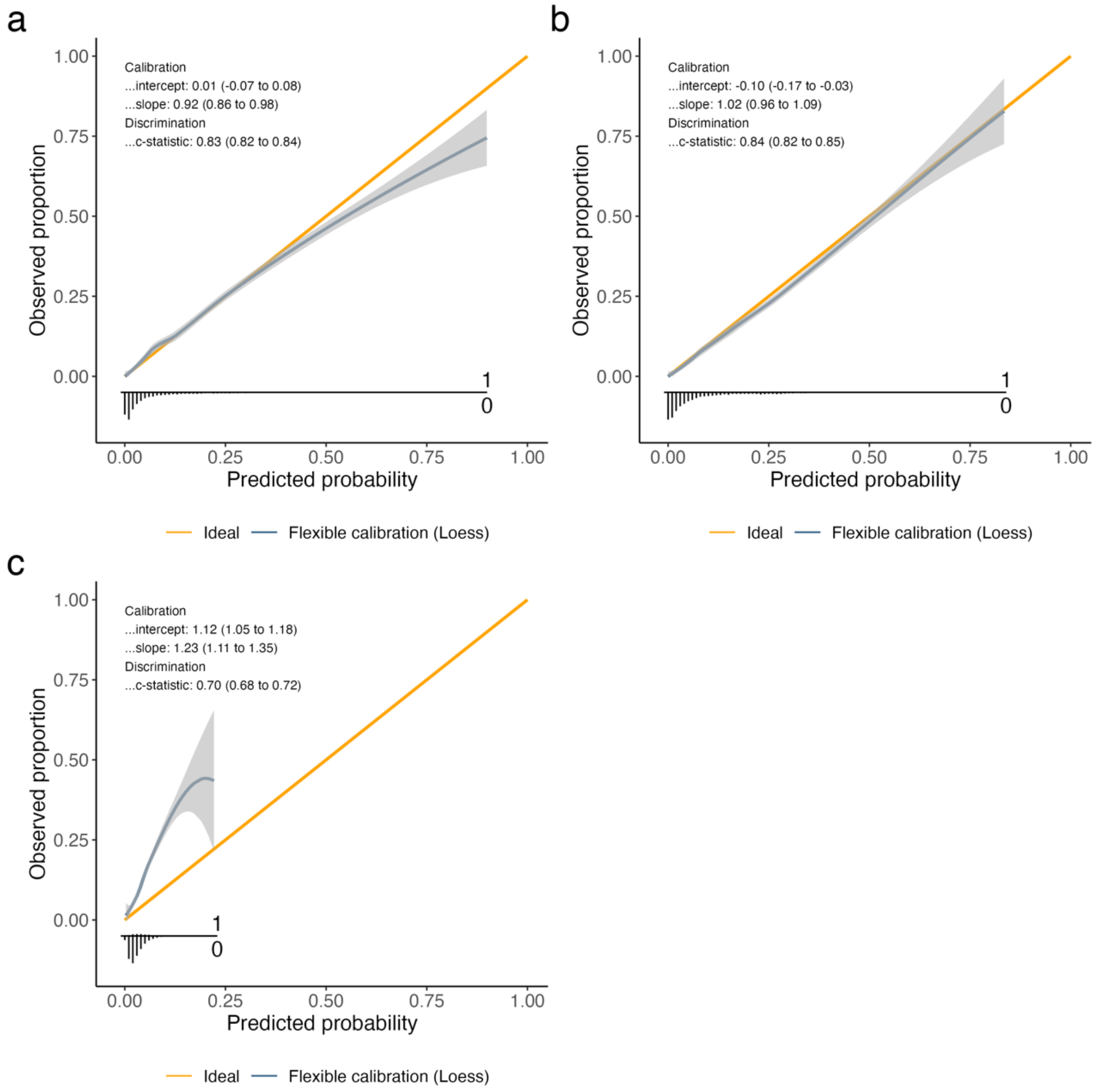

Figure S5.6.2. IST as training dataset: calibration plot of predicted outcome in the treatment group. The orange line indicates ideal calibration. The outcome variable is death at 14 days for IST and death at 4 weeks for CAST. **a**, S-learner BART. **b**, CVAE. **c**, GANITE. Abbreviations: IST: the International Stroke Trial.

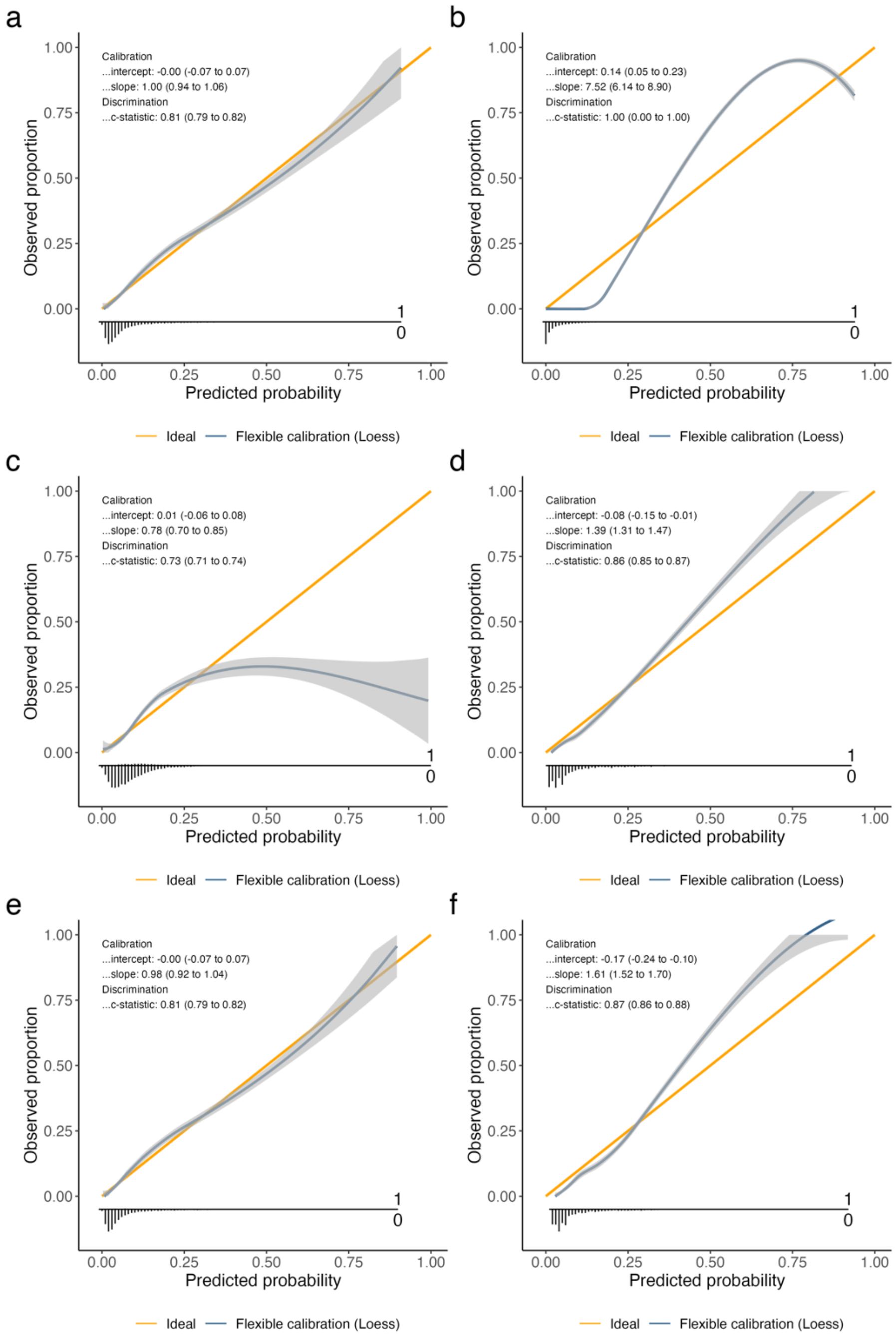

Figure S5.7.1. IST as training dataset: calibration plot of predicted outcome in the control group. The orange line indicates ideal calibration. The outcome variable is death at 14 days for IST and death at 4 weeks for CAST. **a**, T-learner Logistic Regression. **b**, T-learner Random Forest. **c**, T-learner Support Vector Machine. **d**, T-learner XGBoost. **e**, S-learner Logistic Regression. **f**, S-learner XGBoost. Abbreviations: IST: the International Stroke Trial.

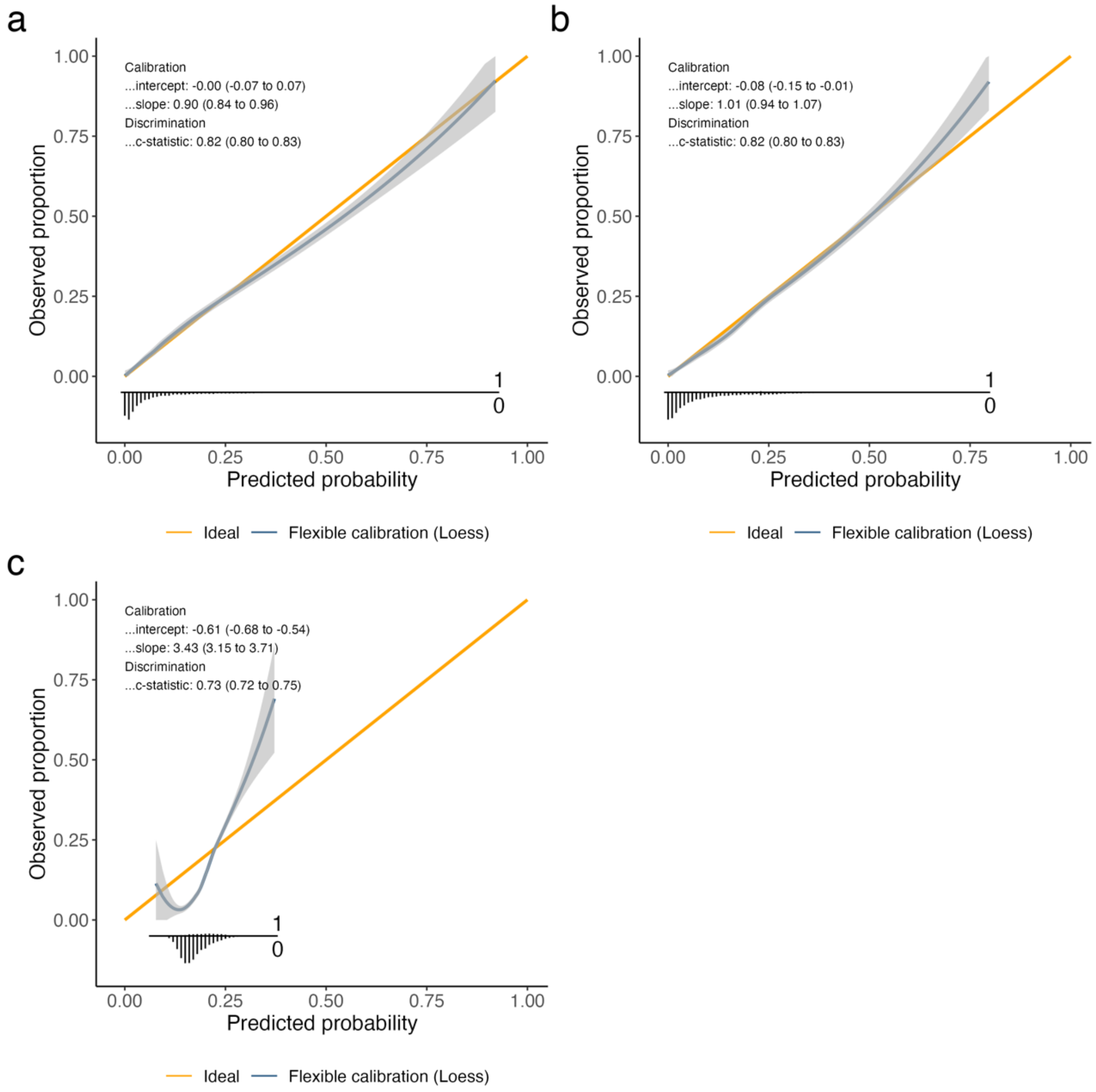

Figure S5.7.2. IST as training dataset: calibration plot of predicted outcome in the control group. The orange line indicates ideal calibration. The outcome variable is death at 14 days for IST and death at 4 weeks for CAST. **a**, S-learner BART. **b**, CVAE. **c**, GANITE. Abbreviations: IST: the International Stroke Trial.

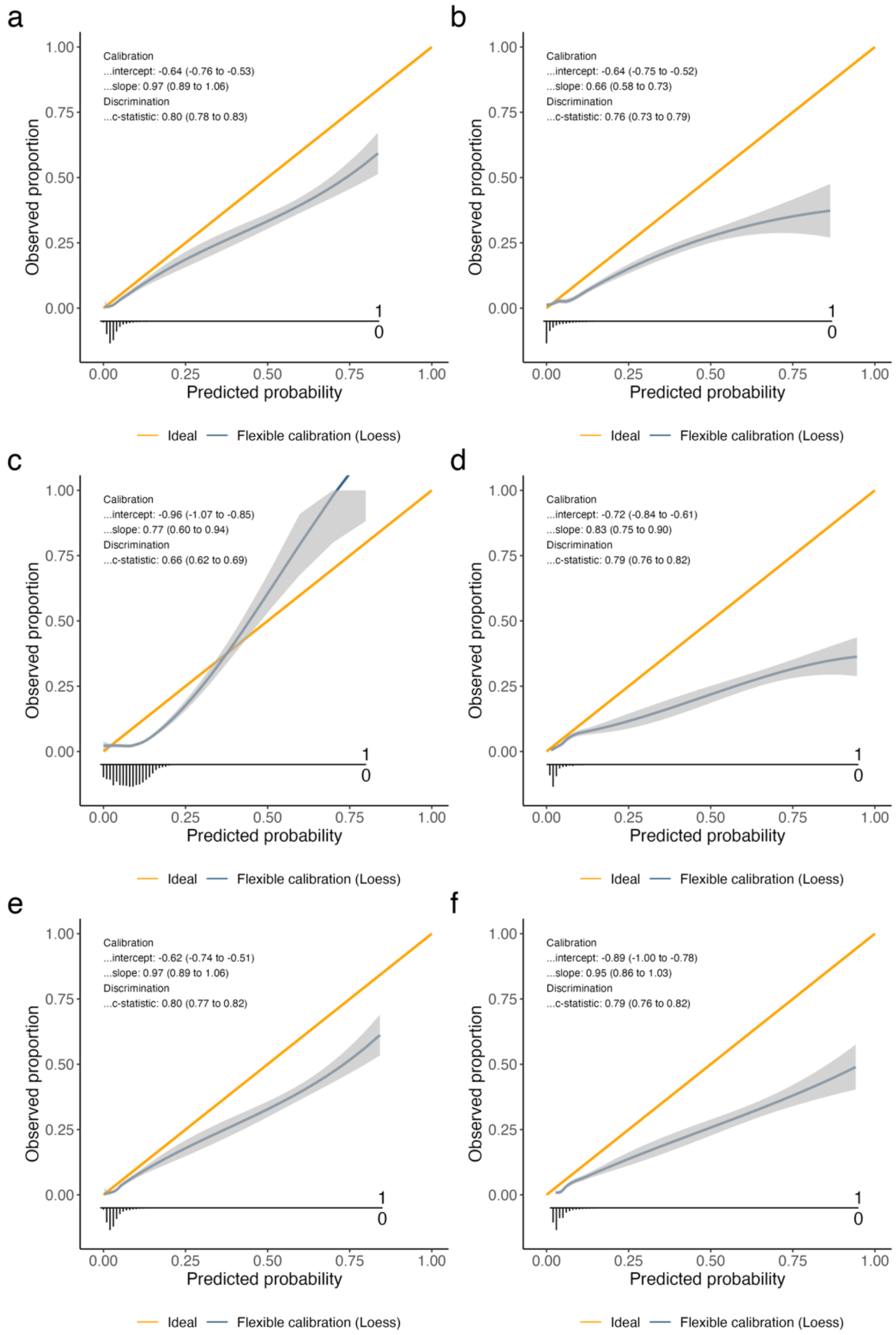

Figure S5.8.1. CAST as test dataset: calibration plot of predicted outcome in the treatment group. The orange line indicates ideal calibration. The outcome variable is death at 14 days for IST and death at 4 weeks for CAST. **a**, T-learner Logistic Regression. **b**, T-learner Random Forest. **c**, T-learner Support Vector Machine. **d**, T-learner XGBoost. **e**, S-learner Logistic Regression. **f**, S-learner XGBoost. Abbreviations: CAST: the Chinese Acute Stroke Trial.

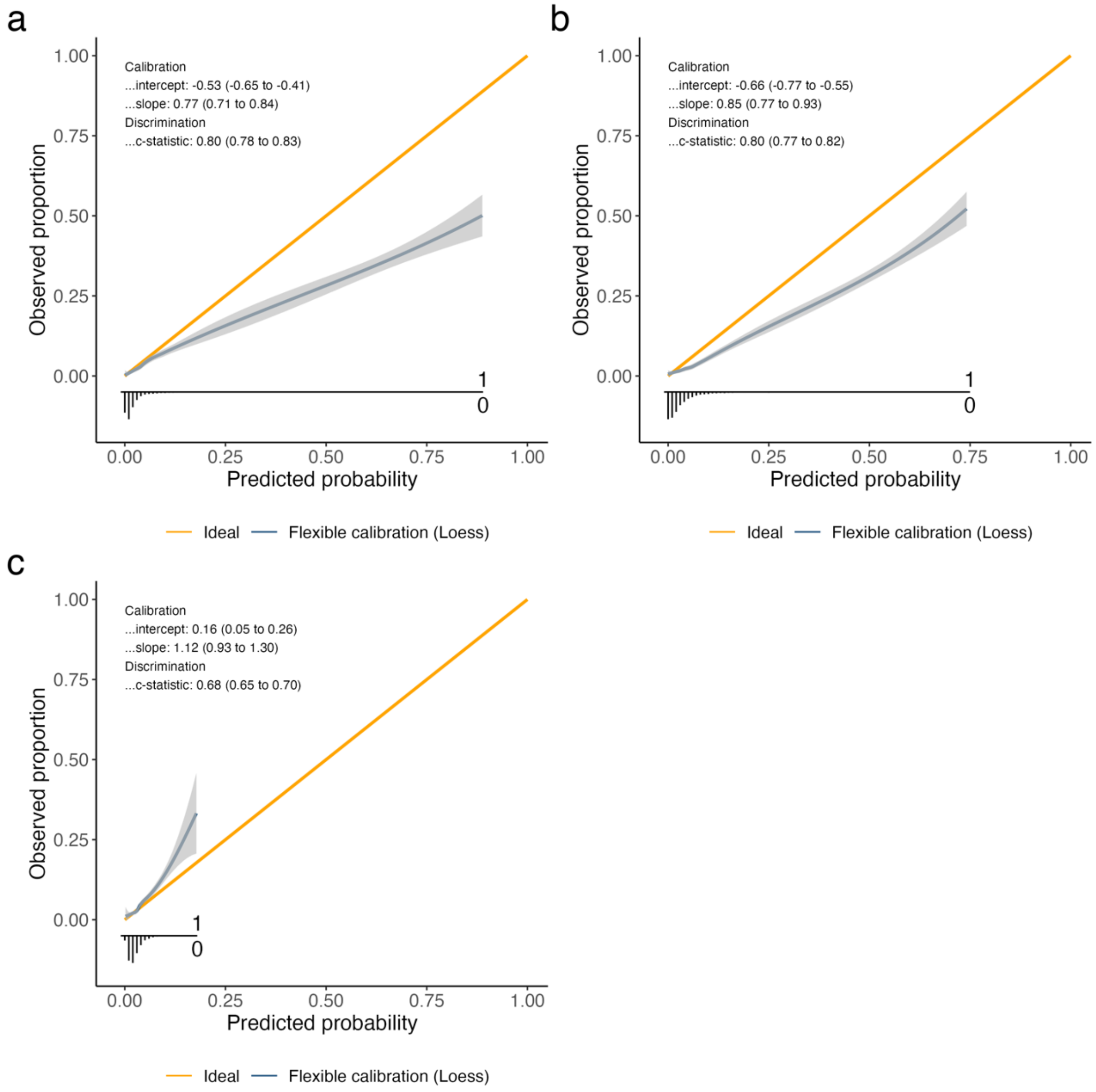

Figure S5.8.2. CAST as test dataset: calibration plot of predicted outcome in the treatment group. The orange line indicates ideal calibration. The outcome variable is death at 14 days for IST and death at 4 weeks for CAST. **a**, S-learner BART. **b**, CVAE. **c**, GANITE. Abbreviations: CAST: the Chinese Acute Stroke Trial.

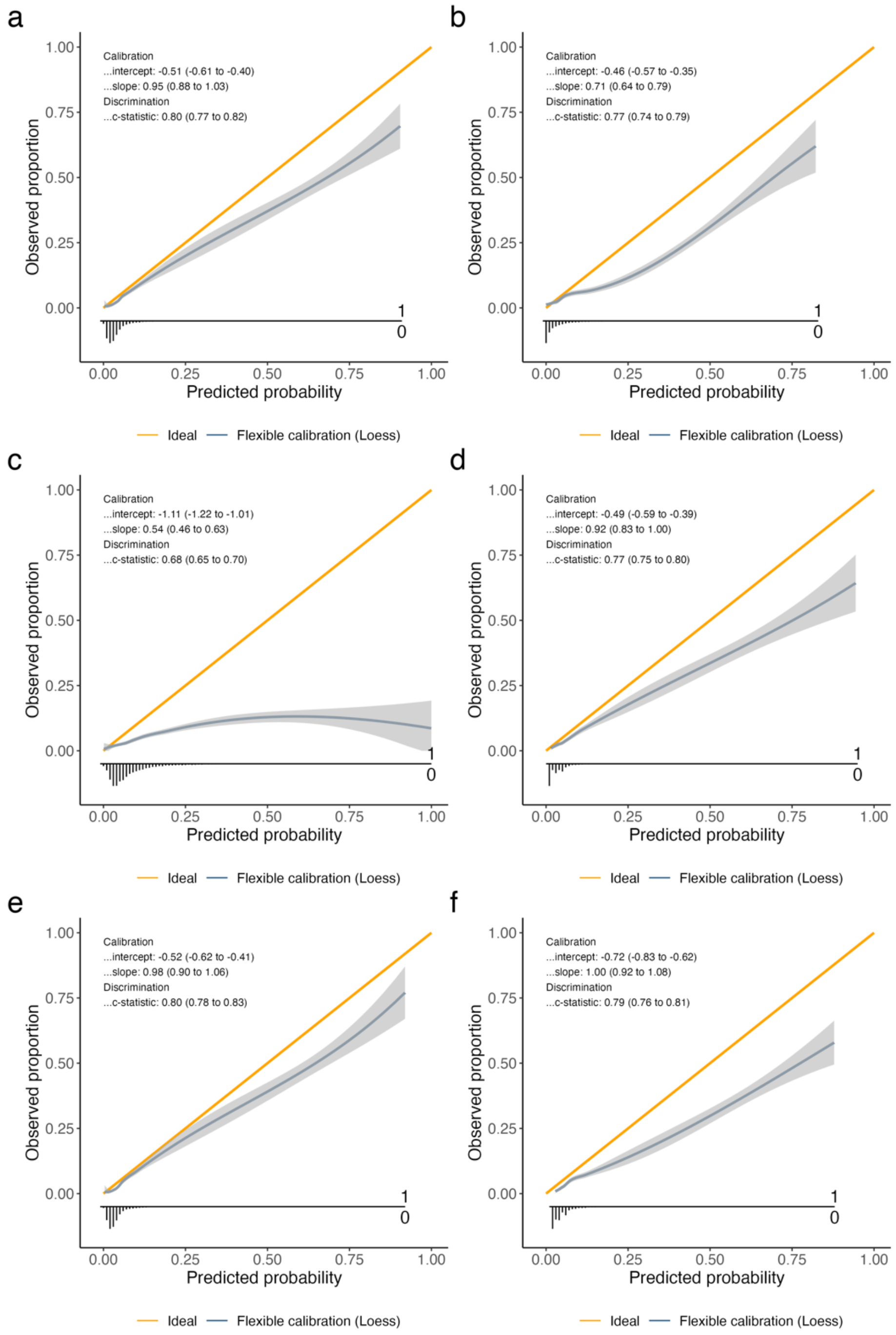

Figure S5.9.1. CAST as test dataset: calibration plot of predicted outcome in the control group. The orange line indicates ideal calibration. The outcome variable is death at 14 days for IST and death at 4 weeks for CAST. **a**, T-learner Logistic Regression. **b**, T-learner Random Forest. **c**, T-learner Support Vector Machine. **d**, T-learner XGBoost. **e**, S-learner Logistic Regression. **f**, S-learner XGBoost. Abbreviations: CAST: the Chinese Acute Stroke Trial.

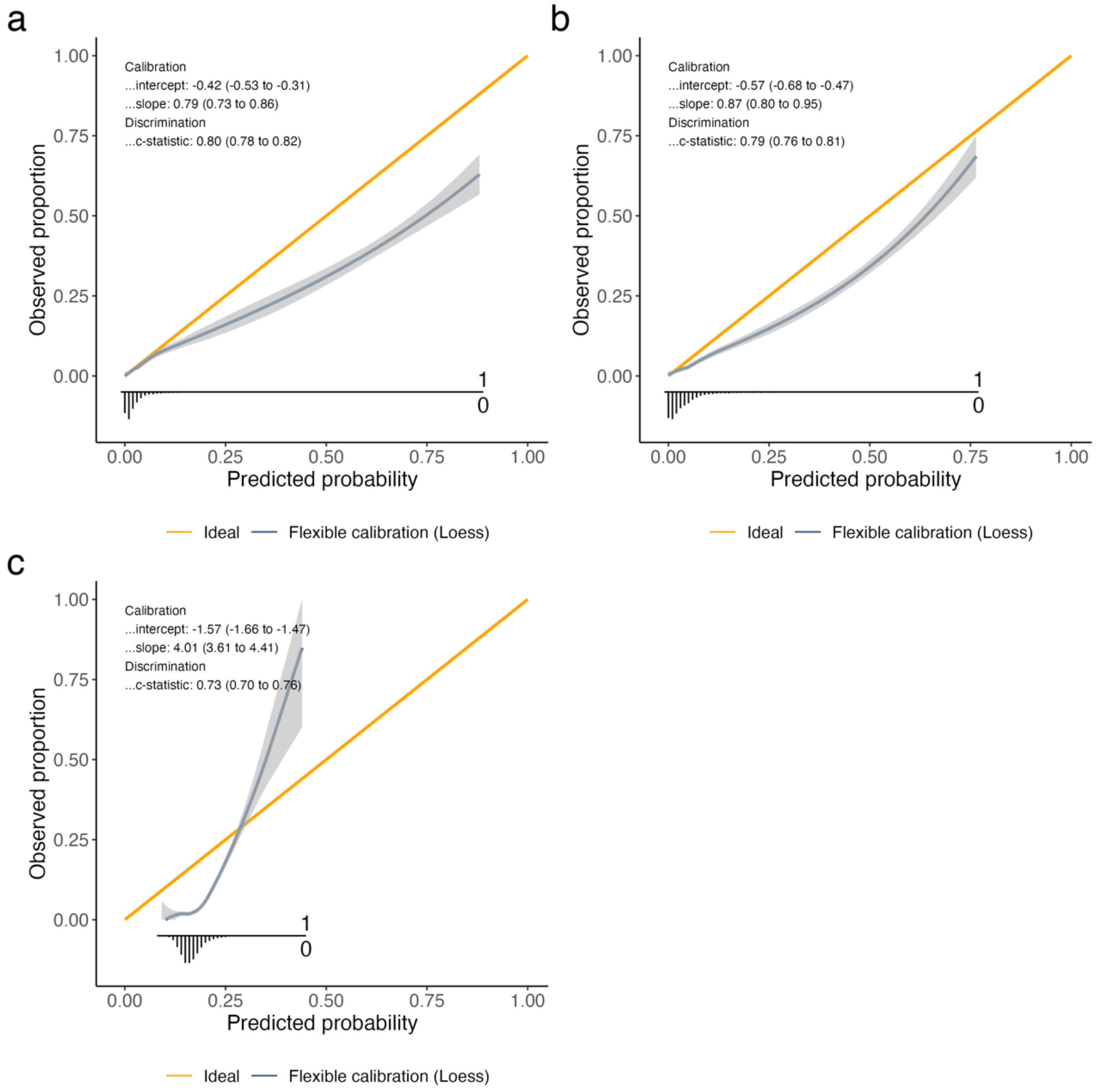

Figure S5.9.2. CAST as test dataset: calibration plot of predicted outcome in the control group. The orange line indicates ideal calibration. The outcome variable is death at 14 days for IST and death at 4 weeks for CAST. **a**, S-learner BART. **b**, CVAE. **c**, GANITE. Abbreviations: CAST: the Chinese Acute Stroke Trial.

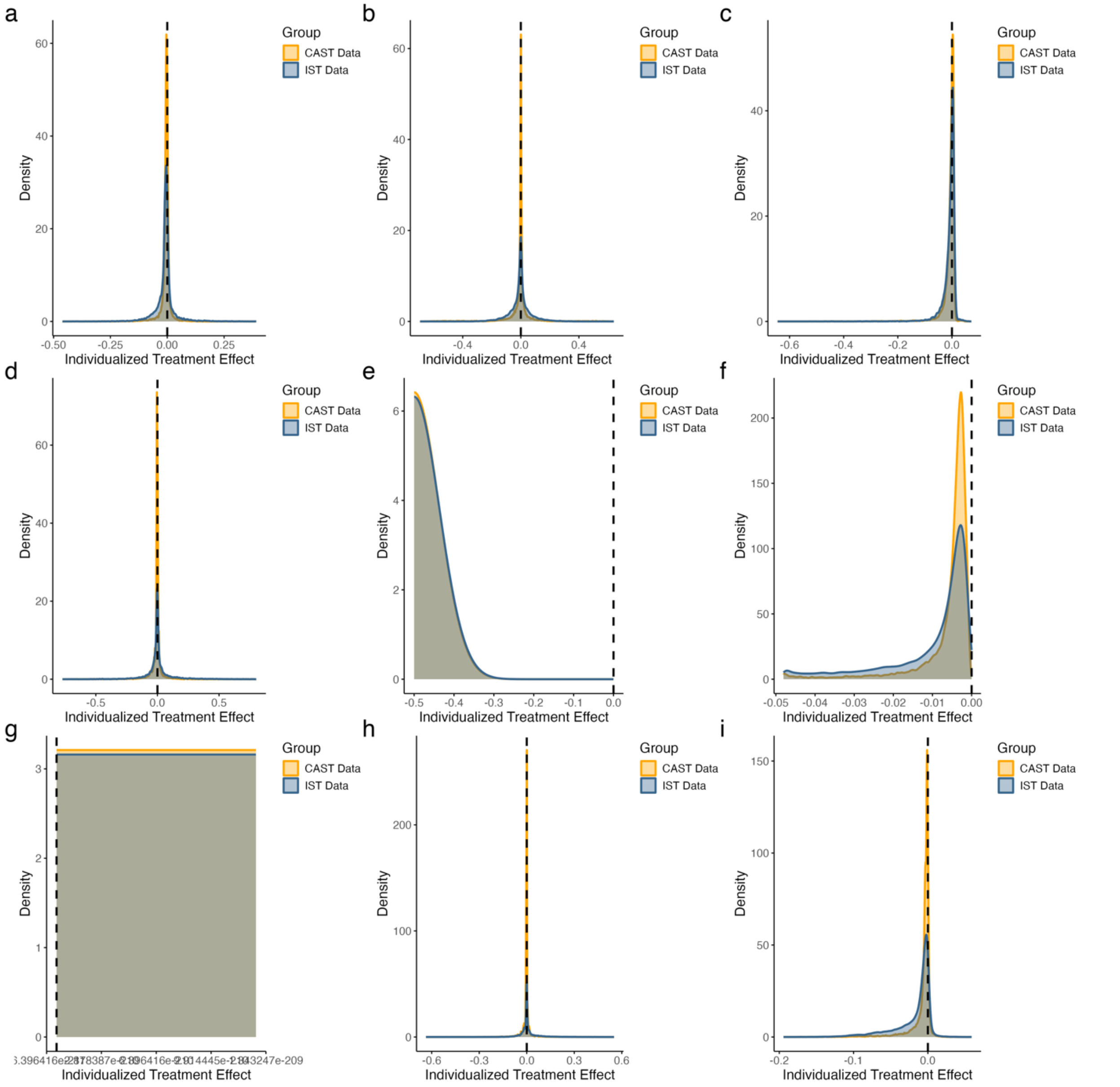

Figure S6.1.1. External validation with CAST as training data and IST as test data: density plots of causal machine learning-based individualized treatment effects. Orange represents the training data, and blue indicates the test data. The outcome variable is death at 14 days for IST and death at 4 weeks for CAST. **a**, T-learner Logistic Regression. **b**, T-learner Random Forest. **c**, T-learner Support Vector Machine. **d**, T-learner XGBoost. **e**, T-learner Penalized Logistic Regression. **f**, S-learner Logistic Regression. **g**, S-learner Penalized Logistic Regression. **h**, S-learner XGBoost. **i**, S-learner BART. Abbreviations: IST: the International Stroke Trial, CAST: the Chinese Acute Stroke Trial, ITE: individualized treatment effect.

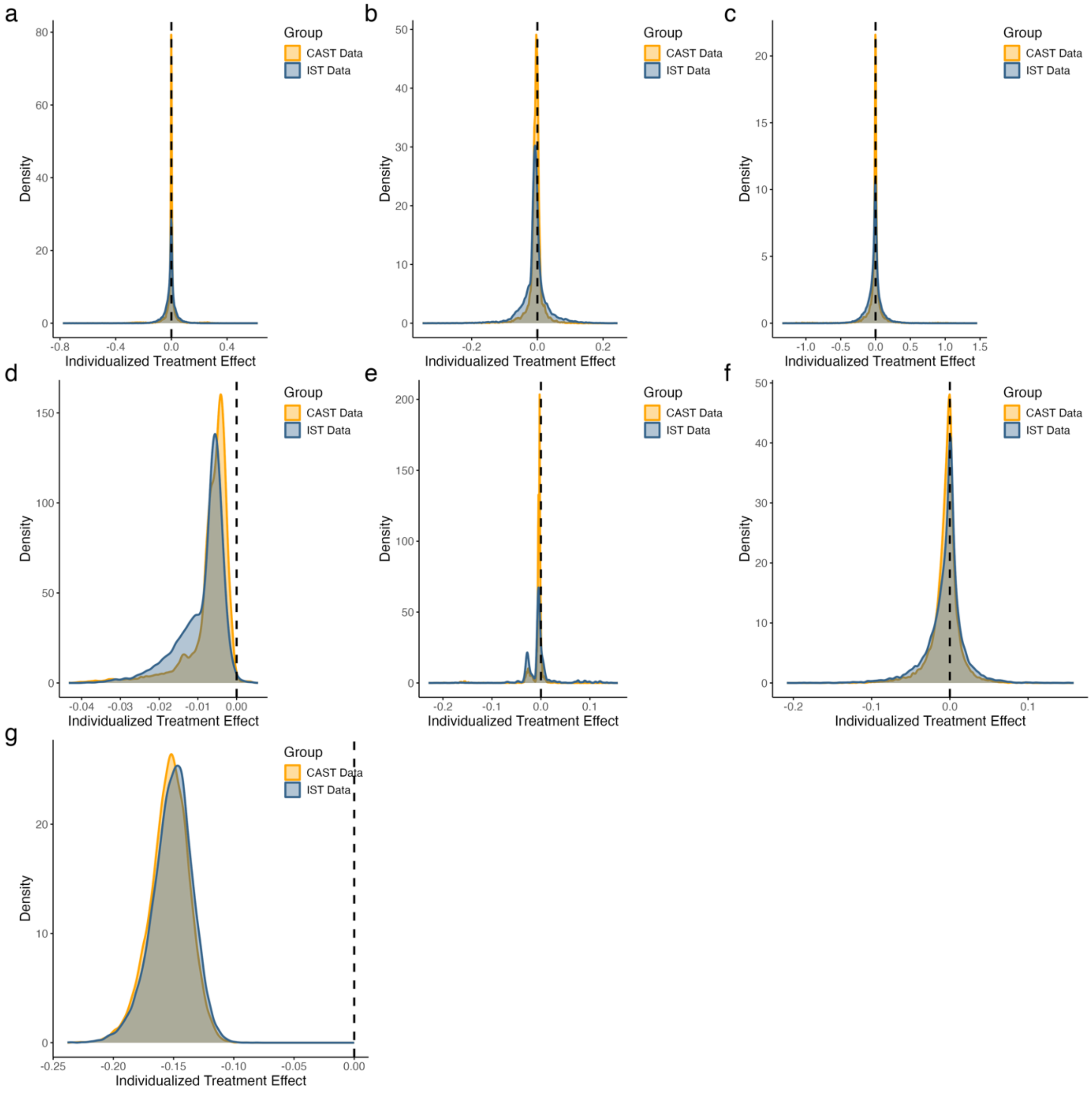

Figure S6.1.2. External validation with CAST as training data and IST as test data: density plots of causal machine learning-based individualized treatment effects. Orange represents the training data, and blue indicates the test data. The outcome variable is death at 14 days for IST and death at 4 weeks for CAST. **a**, X-learner Random Forest. **b**, X-learner BART. **c**, DR-learner Random Forest. **d**, Causal Forest. **e**, Bayesian Causal Forest. **f**, CVAE. **g**, GANITE. Abbreviations: IST: the International Stroke Trial, CAST: the Chinese Acute Stroke Trial, ITE: individualized treatment effect.

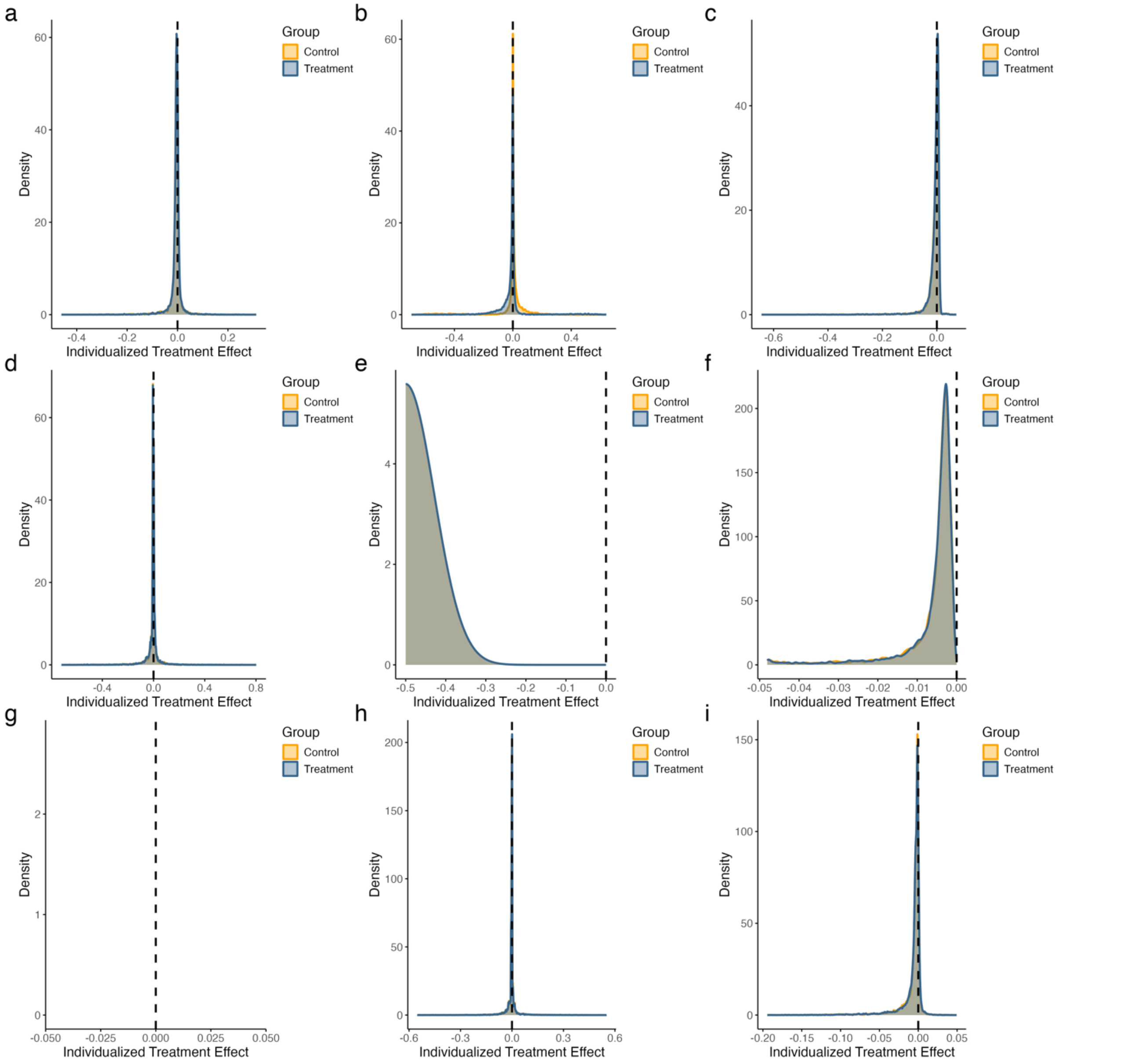

Figure S6.2.1. CAST as training dataset: density plots of causal machine learning-based individualized treatment effects. Orange represents the control group, and blue indicates the treatment group. The outcome variable is death at 14 days for IST and death at 4 weeks for CAST. **a**, T-learner Logistic Regression. **b**, T-learner Random Forest. **c**, T-learner Support Vector Machine. **d**, T-learner XGBoost. **e**, T-learner Penalized Logistic Regression. **f**, S-learner Logistic Regression. **g**, S-learner Penalized Logistic Regression. **h**, S-learner XGBoost. **i**, S-learner BART. Abbreviations: IST: the International Stroke Trial, CAST: the Chinese Acute Stroke Trial, ITE: individualized treatment effect.

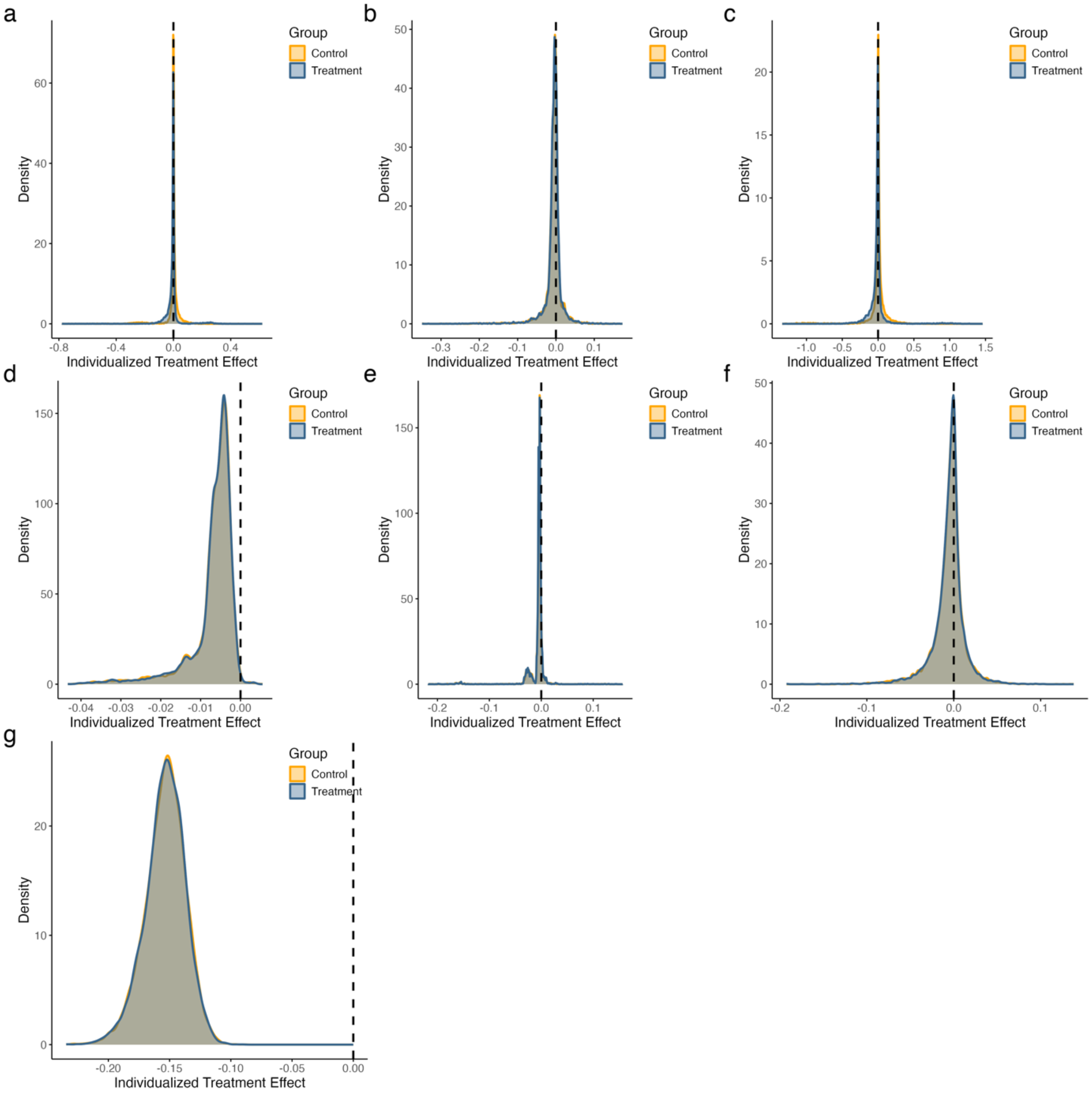

Figure S6.2.2. CAST as training dataset: density plots of causal machine learning-based individualized treatment effects. Orange represents the control group, and blue indicates the treatment group. The outcome variable is death at 14 days for IST and death at 4 weeks for CAST. **a**, X-learner Random Forest. **b**, X-learner BART. **c**, DR-learner Random Forest. **d**, Causal Forest. **e**, Bayesian Causal Forest. **f**, CVAE. **g**, GANITE. Abbreviations: IST: the International Stroke Trial, CAST: the Chinese Acute Stroke Trial, ITE: individualized treatment effect.

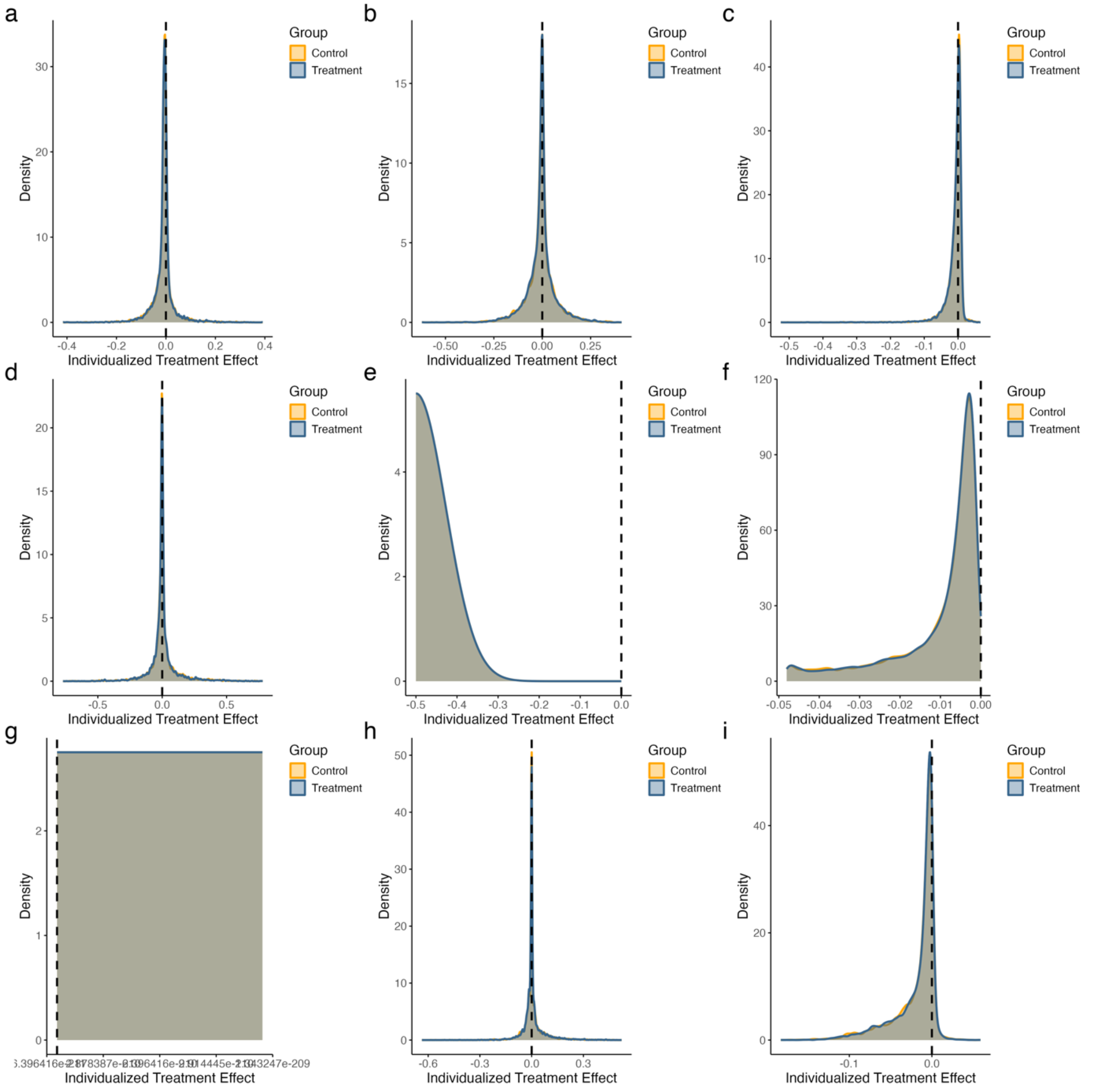

Figure S6.3.1. IST as test dataset: density plots of causal machine learning-based individualized treatment effects. Orange represents the control group, and blue indicates the treatment group. The outcome variable is death at 14 days for IST and death at 4 weeks for CAST. **a**, T-learner Logistic Regression. **b**, T-learner Random Forest. **c**, T-learner Support Vector Machine. **d**, T-learner XGBoost. **e**, T-learner Penalized Logistic Regression. **f**, S-learner Logistic Regression. **g**, S-learner Penalized Logistic Regression. **h**, S-learner XGBoost. **i**, S-learner BART. Abbreviations: IST: the International Stroke Trial, CAST: the Chinese Acute Stroke Trial, ITE: individualized treatment effect.

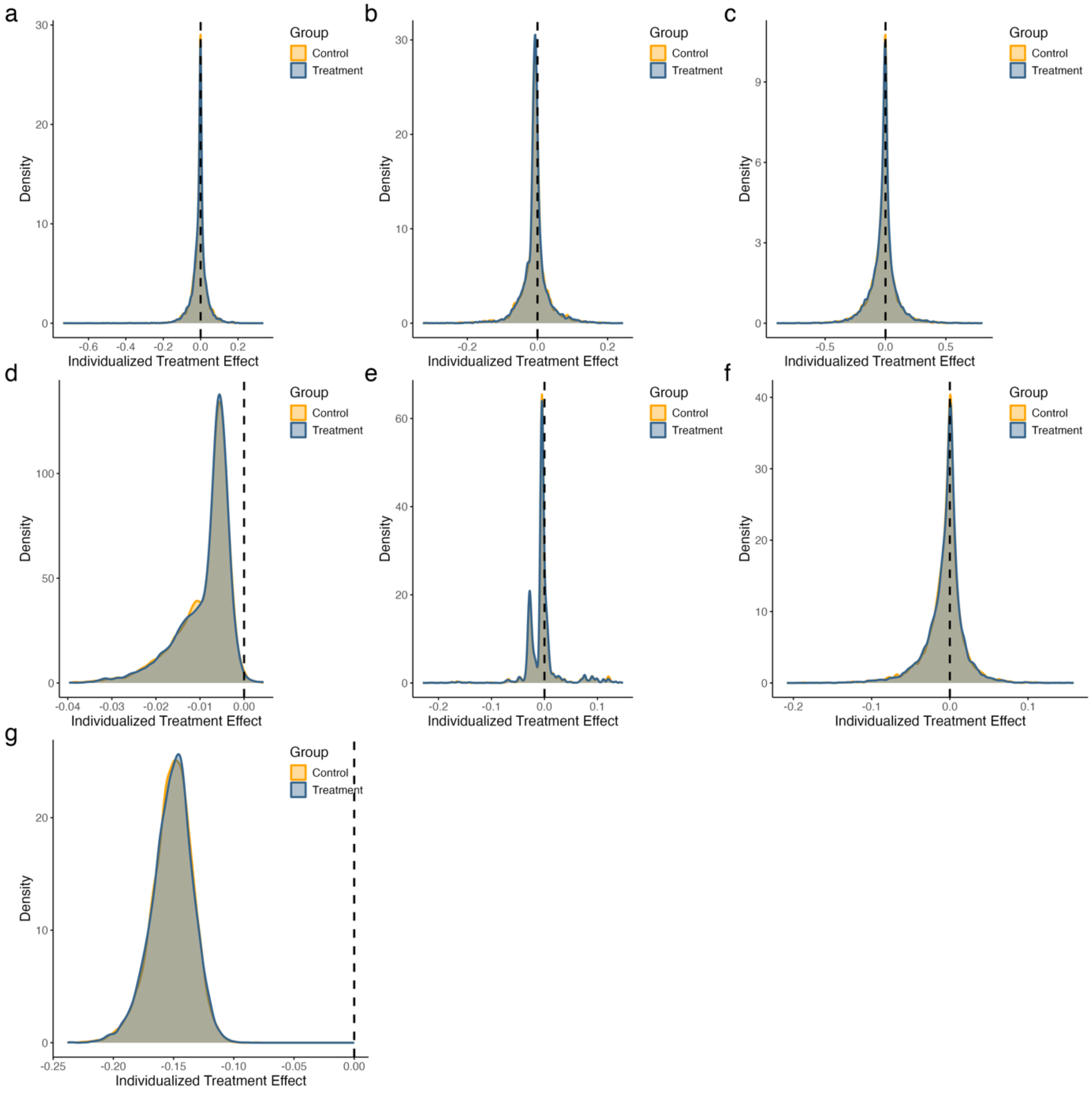

Figure S6.3.2. IST as test dataset: density plots of causal machine learning-based individualized treatment effects. Orange represents the control group, and blue indicates the treatment group. The outcome variable is death at 14 days for IST and death at 4 weeks for CAST. **a**, X-learner Random Forest. **b**, X-learner BART. **c**, DR-learner Random Forest. **d**, Causal Forest. **e**, Bayesian Causal Forest. **f**, CVAE. **g**, GANITE. Abbreviations: IST: the International Stroke Trial, CAST: the Chinese Acute Stroke Trial, ITE: individualized treatment effect.

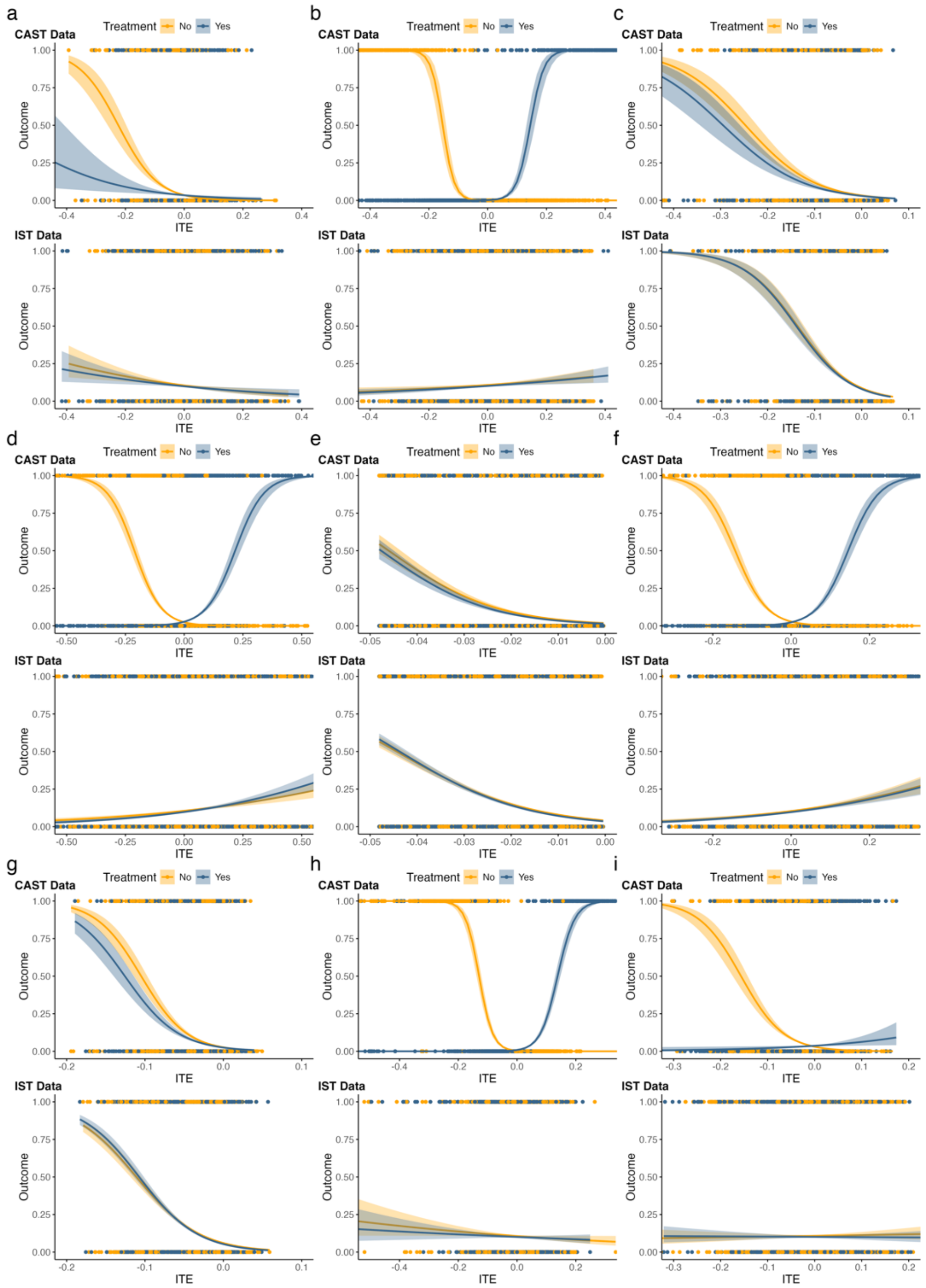

Figure S6.4.1. External validation with CAST as training data and IST as test data: outcome-ITE comparative analysis. Dot plots and line plots depict the true and fitted patient outcomes against estimated ITE values between training data and test data. Orange represents the control group, and blue indicates the treatment group. The outcome variable is death at 14 days for IST and death at 4 weeks for CAST. **a**, T-learner Logistic Regression. **b**, T-learner Random Forest. **c**, T-learner Support Vector Machine. **d**, T-learner XGBoost. **e**, S-learner Logistic Regression. **f**, S-learner XGBoost. **g**, S-learner BART. **h**, X-learner Random Forest. **i**, X-learner BART. Abbreviations: IST: the International Stroke Trial, CAST: the Chinese Acute Stroke Trial, ITE: individualized treatment effect.

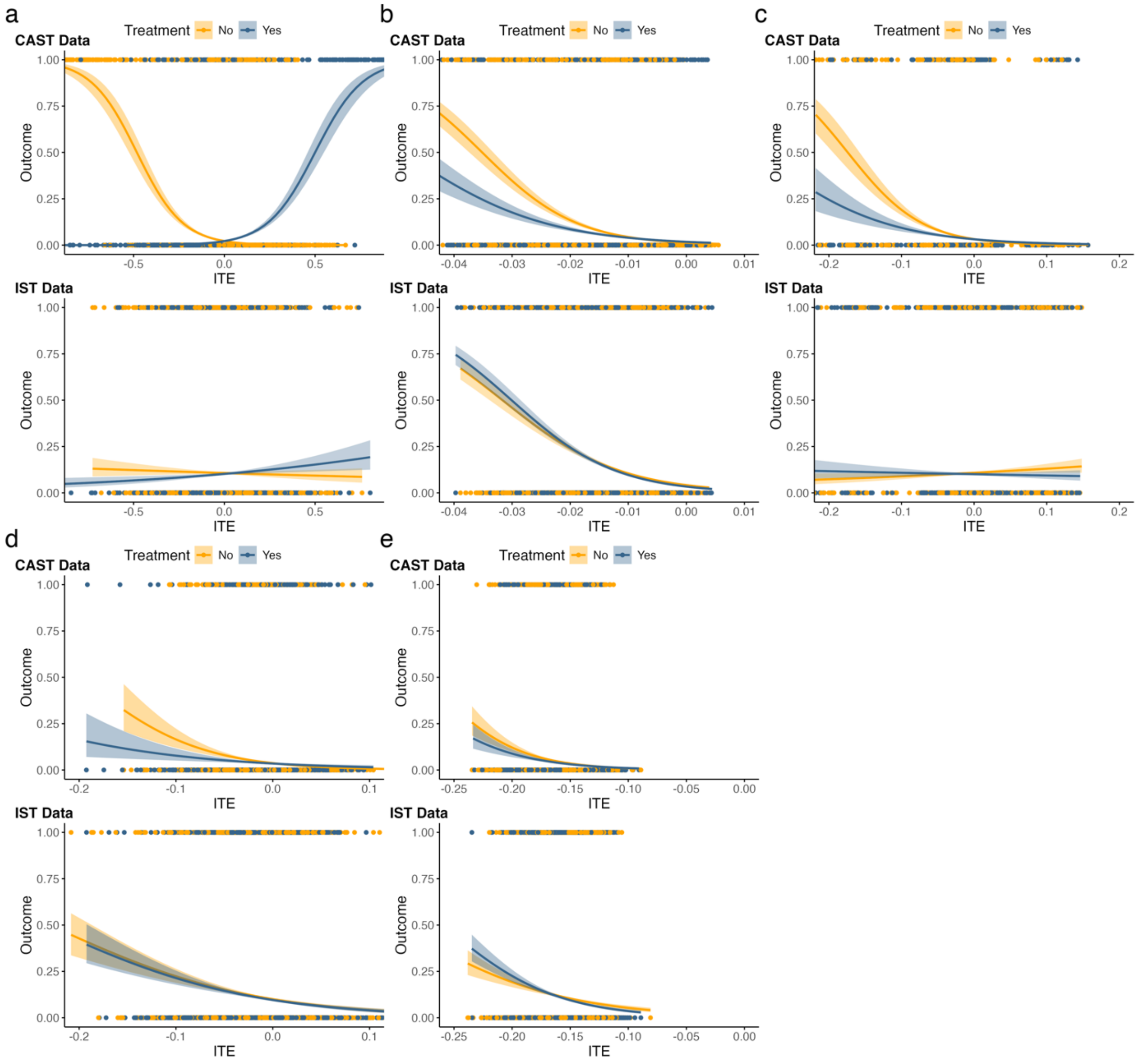

Figure S6.4.2. External validation with CAST as training data and IST as test data: outcome-ITE comparative analysis. Dot plots and line plots depict the true and fitted patient outcomes against estimated ITE values between training data and test data. Orange represents the control group, and blue indicates the treatment group. The outcome variable is death at 14 days for IST and death at 4 weeks for CAST. **a**, DR-learner Random Forest. **b**, Causal Forest. **c**, Bayesian Causal Forest. **d**, CVAE. **e**, GANITE. Abbreviations: IST: the International Stroke Trial, CAST: the Chinese Acute Stroke Trial, ITE: individualized treatment effect.

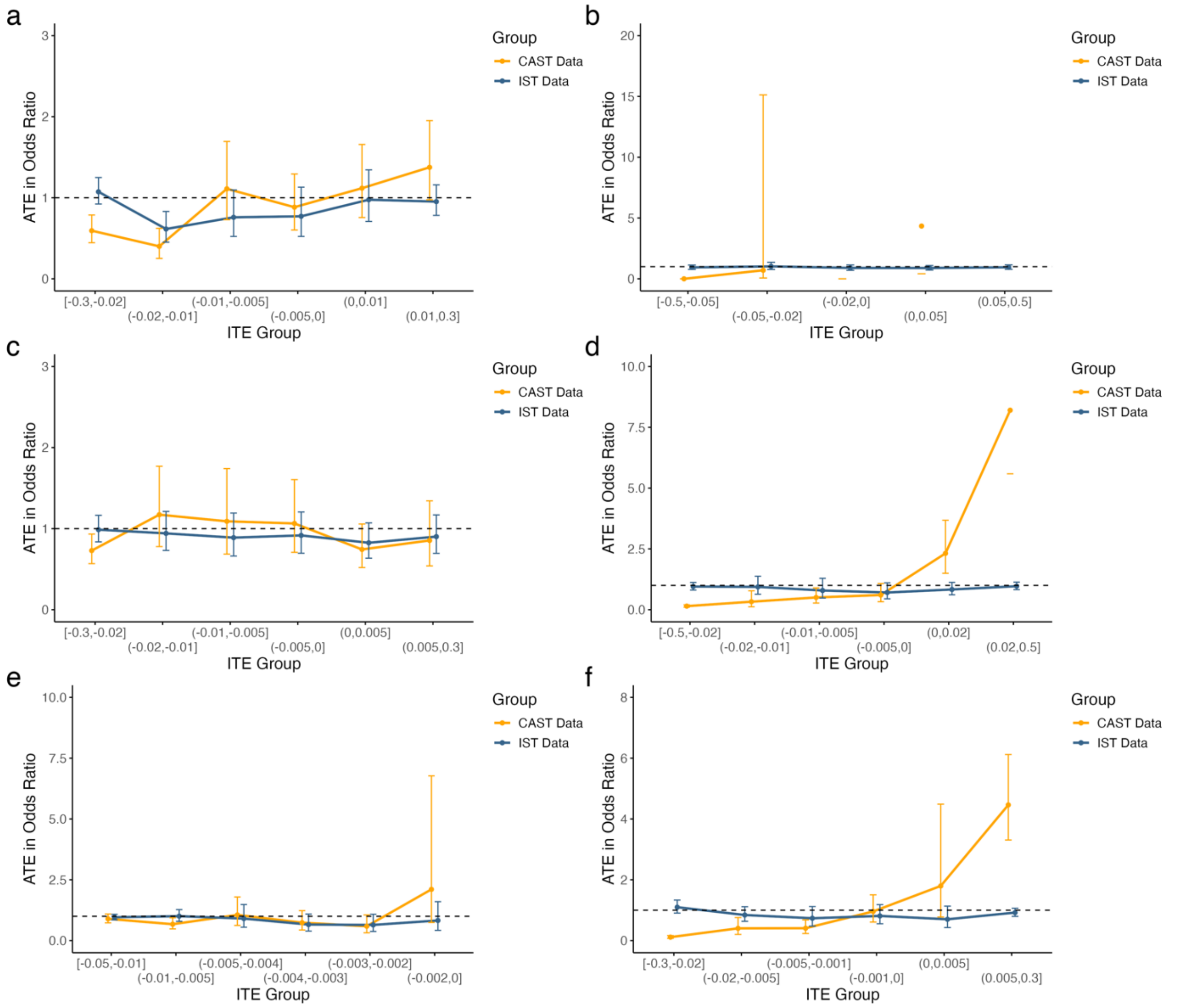

Figure S6.5.1. External validation with CAST as training data and IST as test data: ATE-ITE comparative analysis. Line plots depict ATE in risk ratio within different ITE subgroups and provide the confidence intervals at 95% level. Orange represents the training data, and blue indicates the test data. The horizontal dashed line at 1.0 means no treatment effects. The outcome variable is death at 14 days for IST and death at 4 weeks for CAST. **a**, T-learner Logistic Regression. **b**, T-learner Random Forest. **c**, T-learner Support Vector Machine. **d**, T-learner XGBoost. **e**, S-learner Logistic Regression. **f**, S-learner XGBoost. Abbreviations: IST: the International Stroke Trial, CAST: the Chinese Acute Stroke Trial, ITE: individualized treatment effect, ATE: average treatment effect.

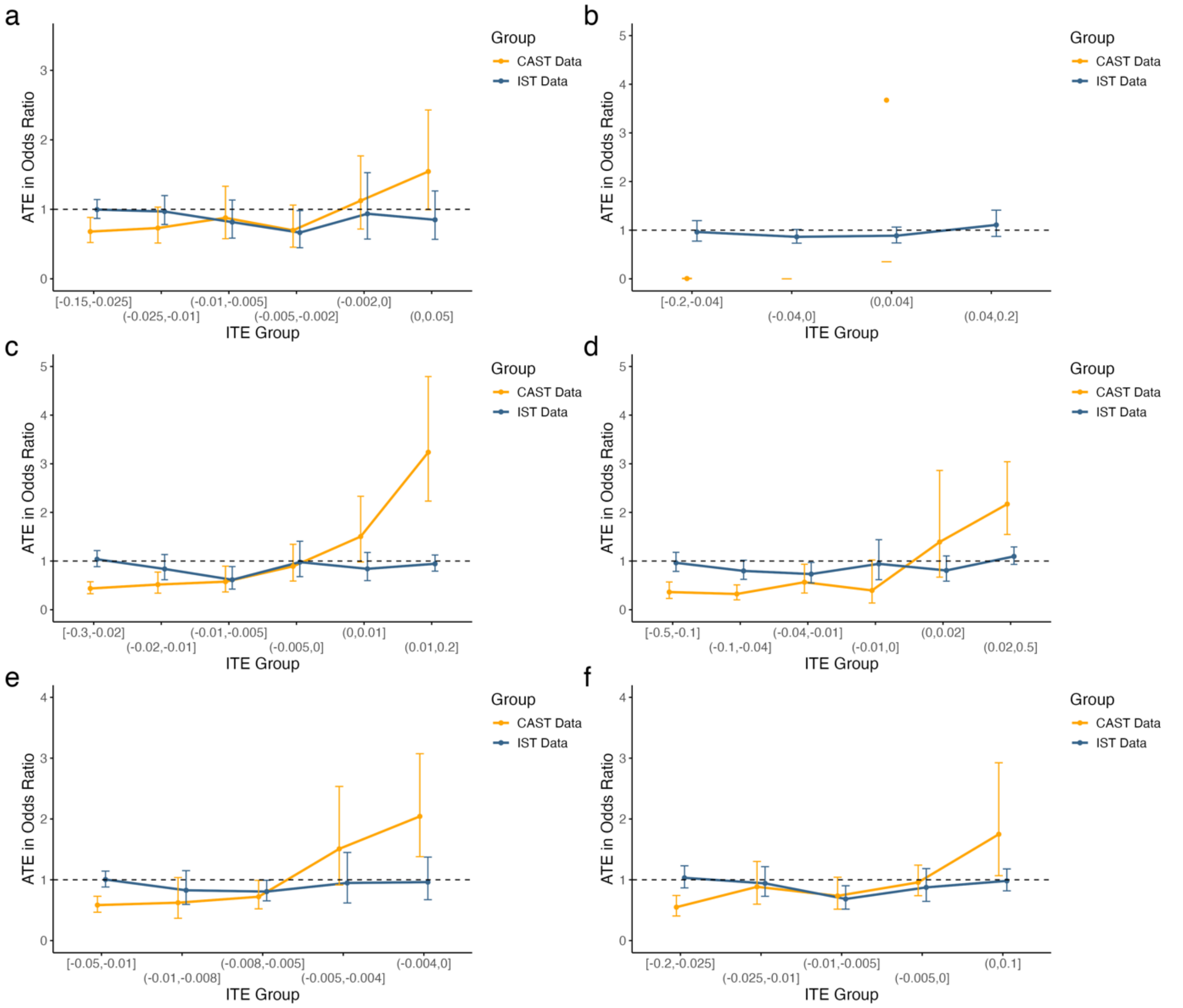

Figure S6.5.2. External validation with CAST as training data and IST as test data: ATE-ITE comparative analysis. Line plots depict ATE in risk ratio within different ITE subgroups and provide the confidence intervals at 95% level. Orange represents the training data, and blue indicates the test data. The horizontal dashed line at 1.0 means no treatment effects. The outcome variable is death at 14 days for IST and death at 4 weeks for CAST. **a**, S-learner BART. **b**, X-learner Random Forest. **c**, X-learner BART. **d**, DR-learner Random Forest. **e**, Causal Forest. **f**, Bayesian Causal Forest. Abbreviations: IST: the International Stroke Trial, CAST: the Chinese Acute Stroke Trial, ITE: individualized treatment effect, ATE: average treatment effect.

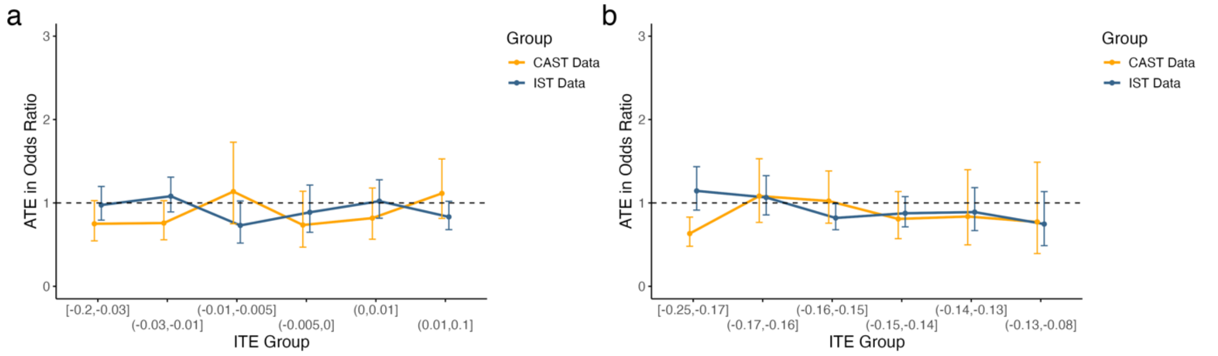

Figure S6.5.2. External validation with CAST as training data and IST as test data: ATE-ITE comparative analysis. Line plots depict ATE in risk ratio within different ITE subgroups and provide the confidence intervals at 95% level. Orange represents the training data, and blue indicates the test data. The horizontal dashed line at 1.0 means no treatment effects. The outcome variable is death at 14 days for IST and death at 4 weeks for CAST. **a**. CVAE. **b**, GANITE. Abbreviations: IST: the International Stroke Trial, CAST: the Chinese Acute Stroke Trial, ITE: individualized treatment effect, ATE: average treatment effect.

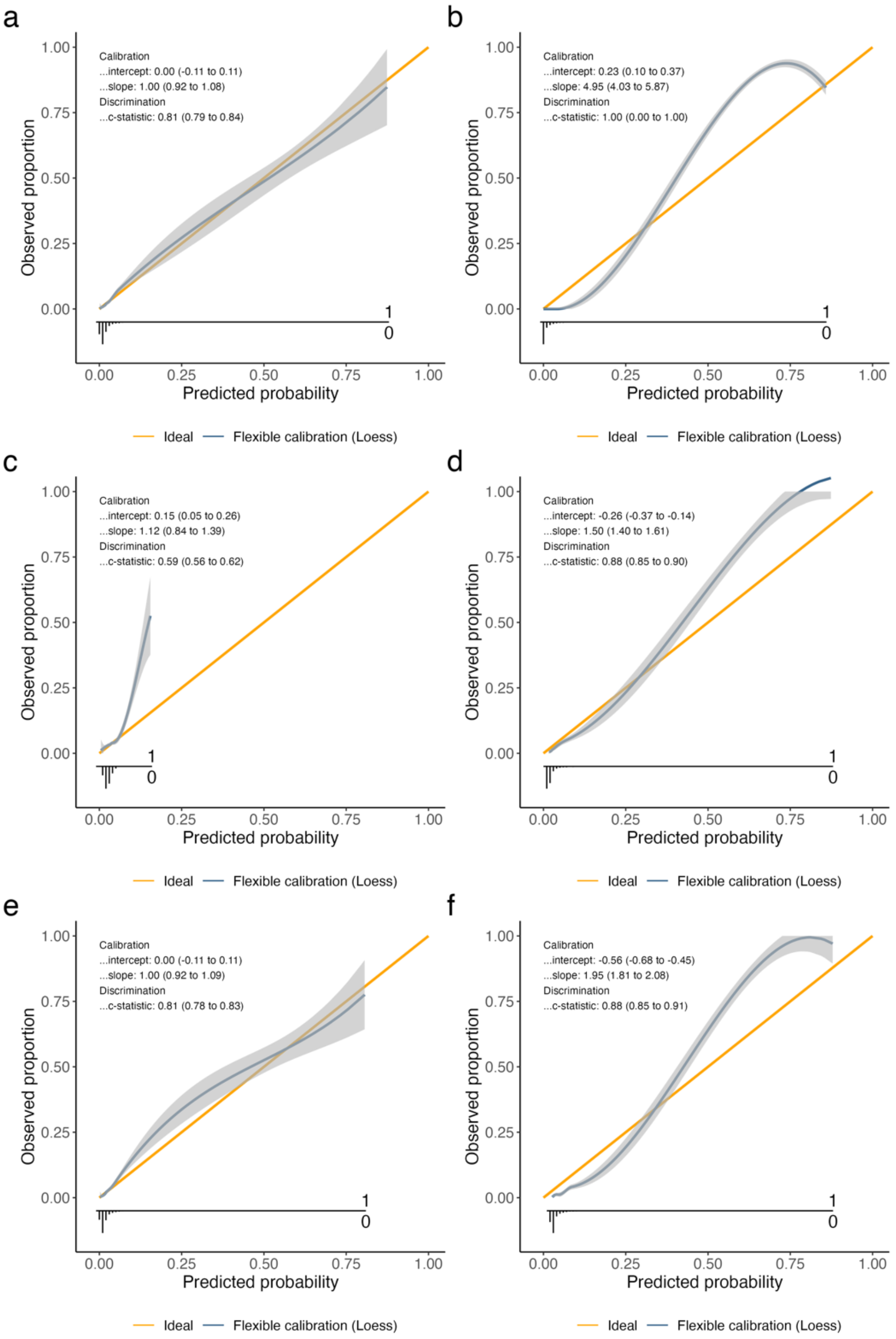

Figure S6.6.1. CAST as training dataset: calibration plot of predicted outcome in the treatment group. The orange line indicates ideal calibration. The outcome variable is death at 14 days for IST and death at 4 weeks for CAST. **a**, T-learner Logistic Regression. **b**, T-learner Random Forest. **c**, T-learner Support Vector Machine. **d**, T-learner XGBoost. **e**, S-learner Logistic Regression. **f**, S-learner XGBoost. Abbreviations: CAST: the Chinese Acute Stroke Trials.

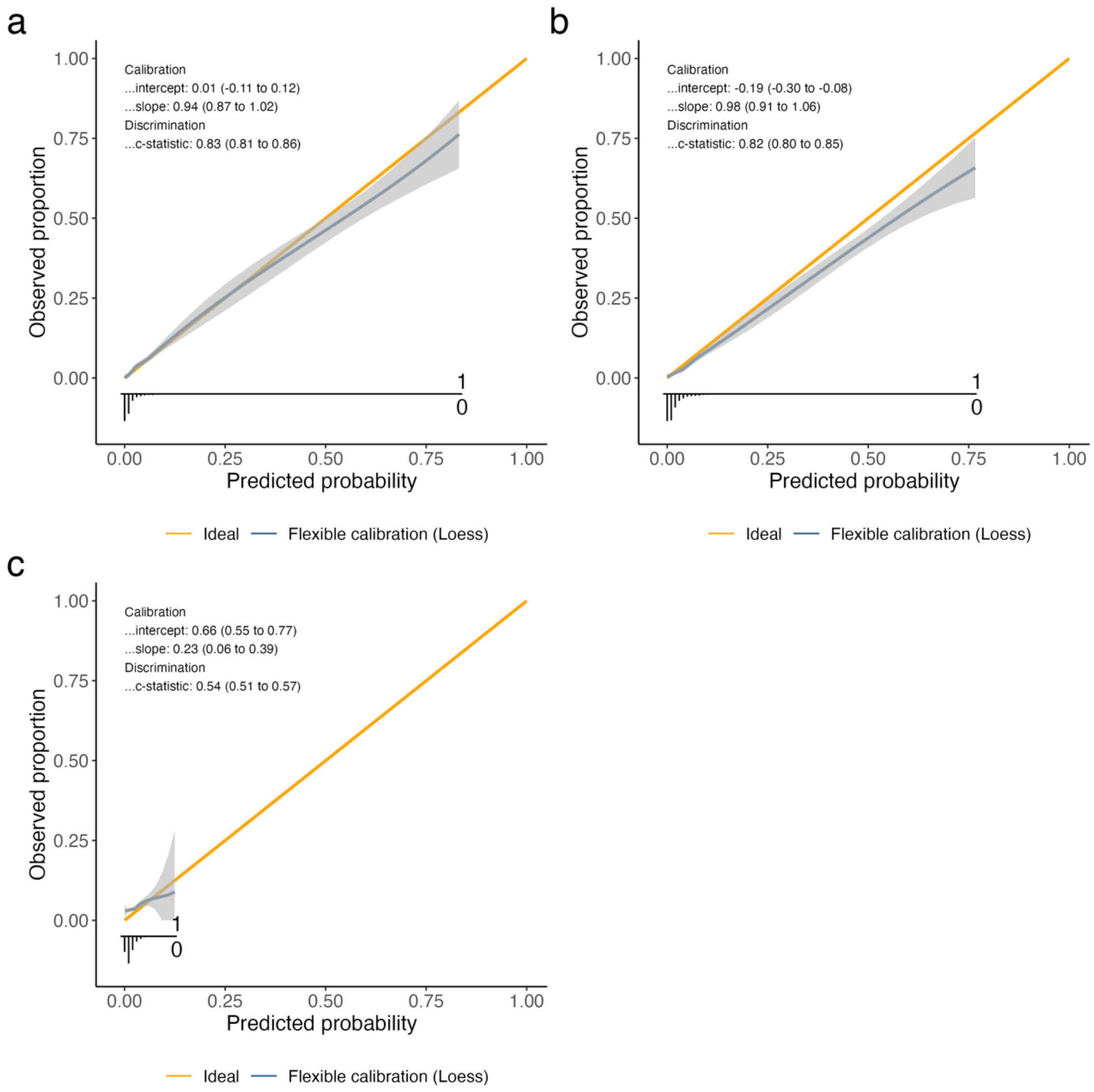

Figure S6.6.2. CAST as training dataset: calibration plot of predicted outcome in the treatment group. The orange line indicates ideal calibration. The outcome variable is death at 14 days for IST and death at 4 weeks for CAST. **a**, S-learner BART. **b**, CVAE. **c**, GANITE. Abbreviations: CAST: the Chinese Acute Stroke Trial.

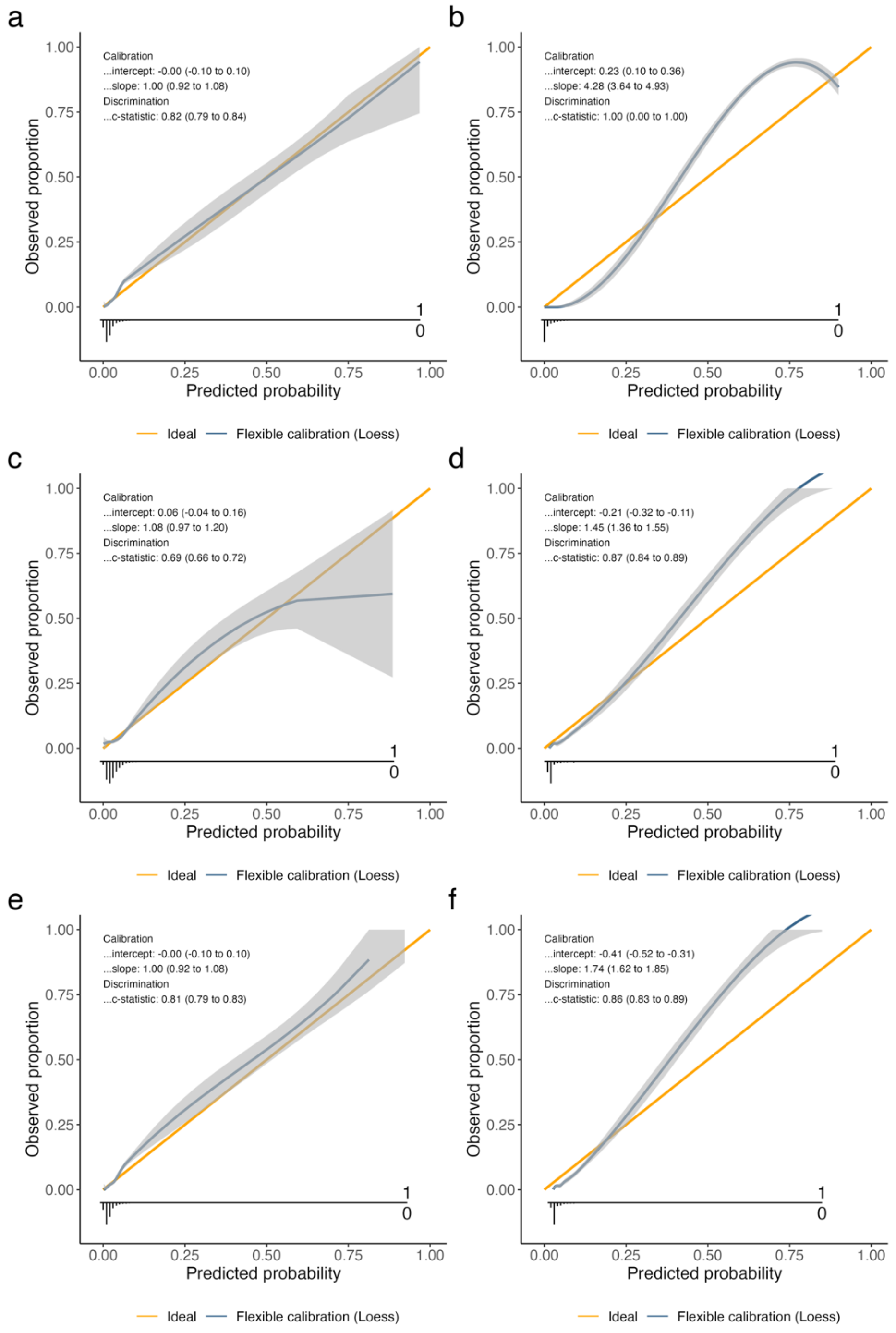

Figure S6.7.1. CAST as training dataset: calibration plot of predicted outcome in the control group. The orange line indicates ideal calibration. The outcome variable is death at 14 days for IST and death at 4 weeks for CAST. **a**, T-learner Logistic Regression. **b**, T-learner Random Forest. **c**, T-learner Support Vector Machine. **d**, T-learner XGBoost. **e**, S-learner Logistic Regression. **f**, S-learner XGBoost. Abbreviations: CAST: the Chinese Acute Stroke Trial.

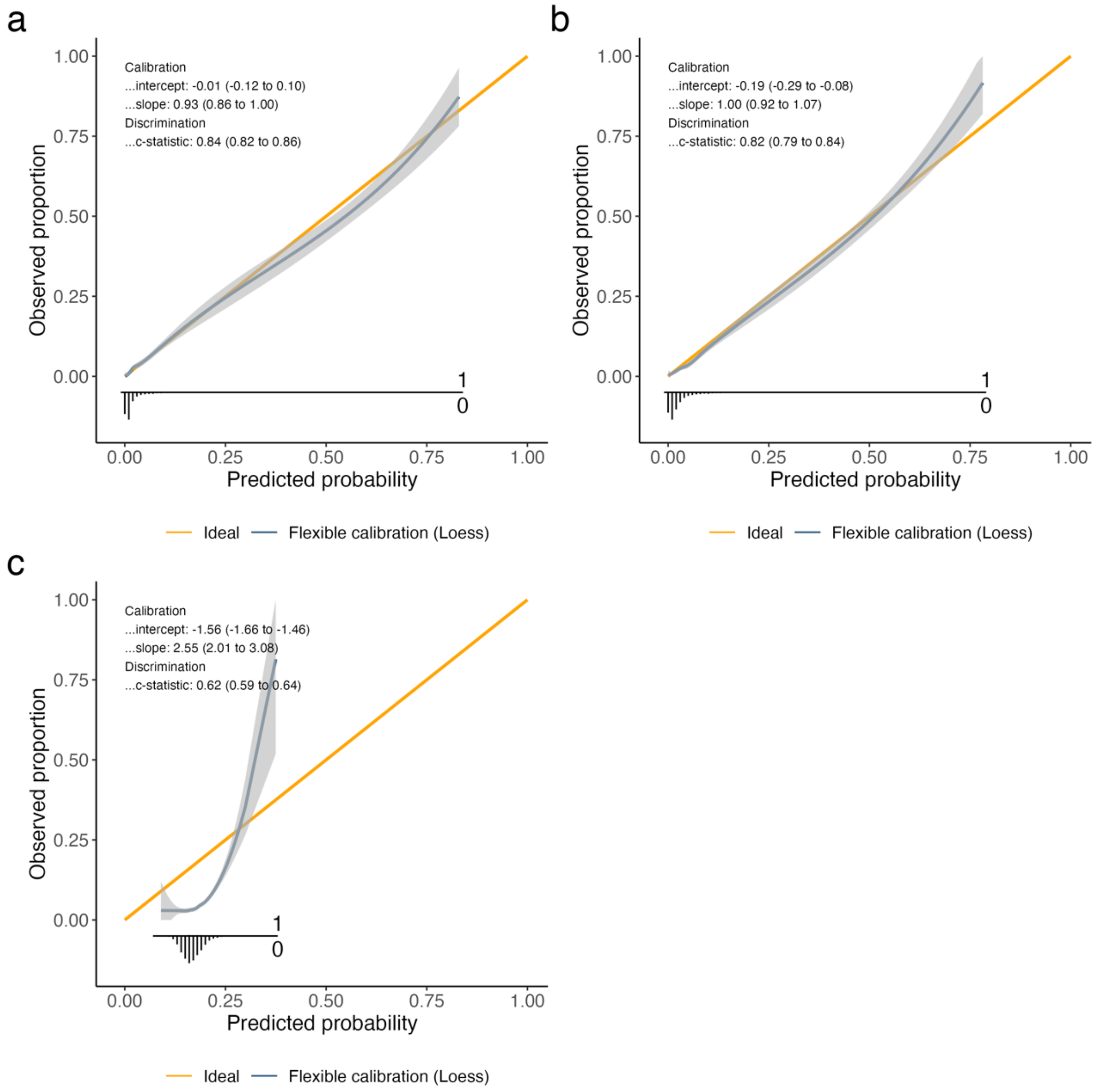

Figure S6.7.2. CAST as training dataset: calibration plot of predicted outcome in the control group. The orange line indicates ideal calibration. The outcome variable is death at 14 days for IST and death at 4 weeks for CAST. **a**, S-learner BART. **b**, CVAE. **c**, GANITE. Abbreviations: CAST: the Chinese Acute Stroke Trial.

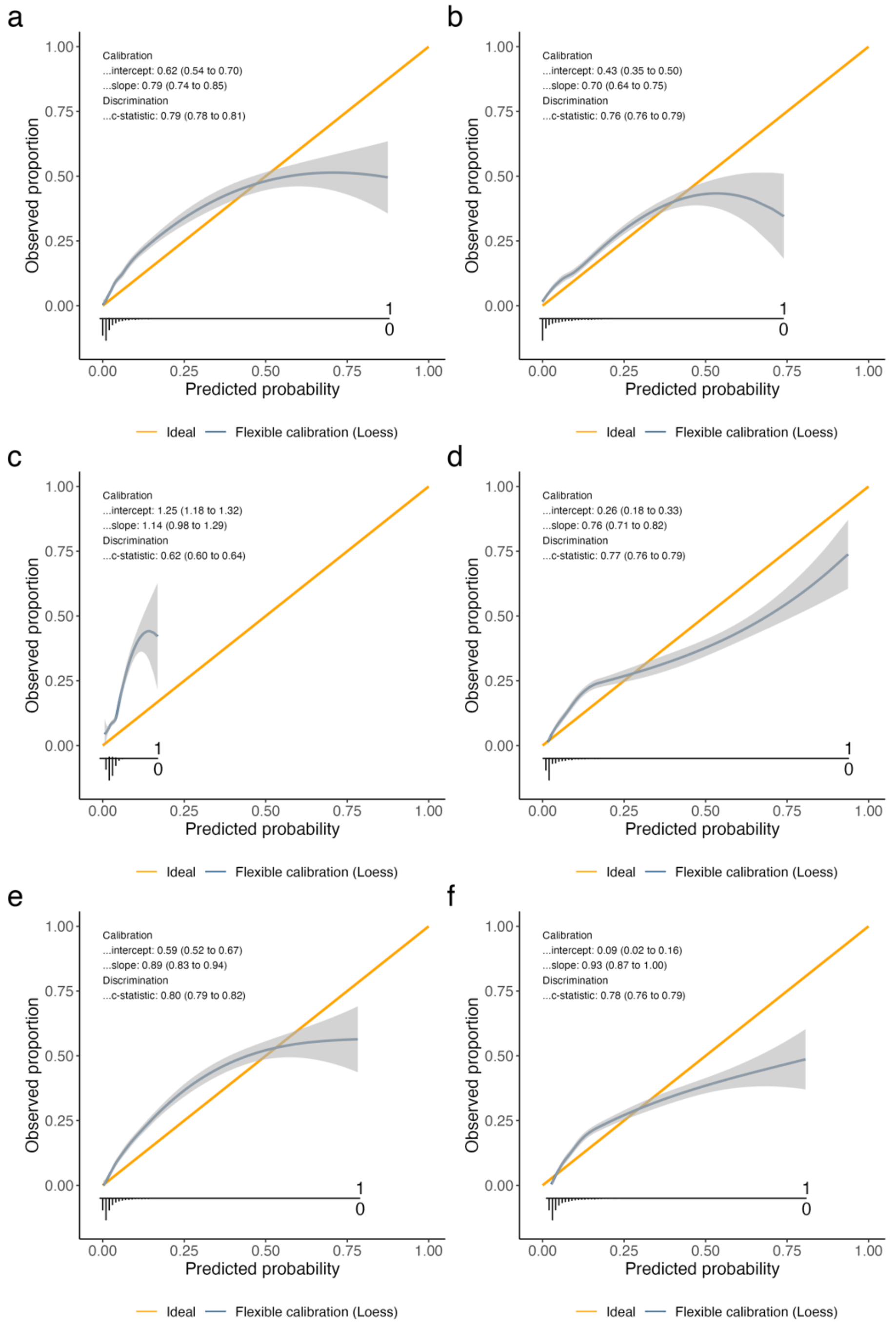

Figure S6.8.1. IST as test dataset: calibration plot of predicted outcome in the treatment group. The orange line indicates ideal calibration. The outcome variable is death at 14 days for IST and death at 4 weeks for CAST. **a**, T-learner Logistic Regression. **b**, T-learner Random Forest. **c**, T-learner Support Vector Machine. **d**, T-learner XGBoost. **e**, S-learner Logistic Regression. **f**, S-learner XGBoost. Abbreviations: IST: the International Stroke Trial.

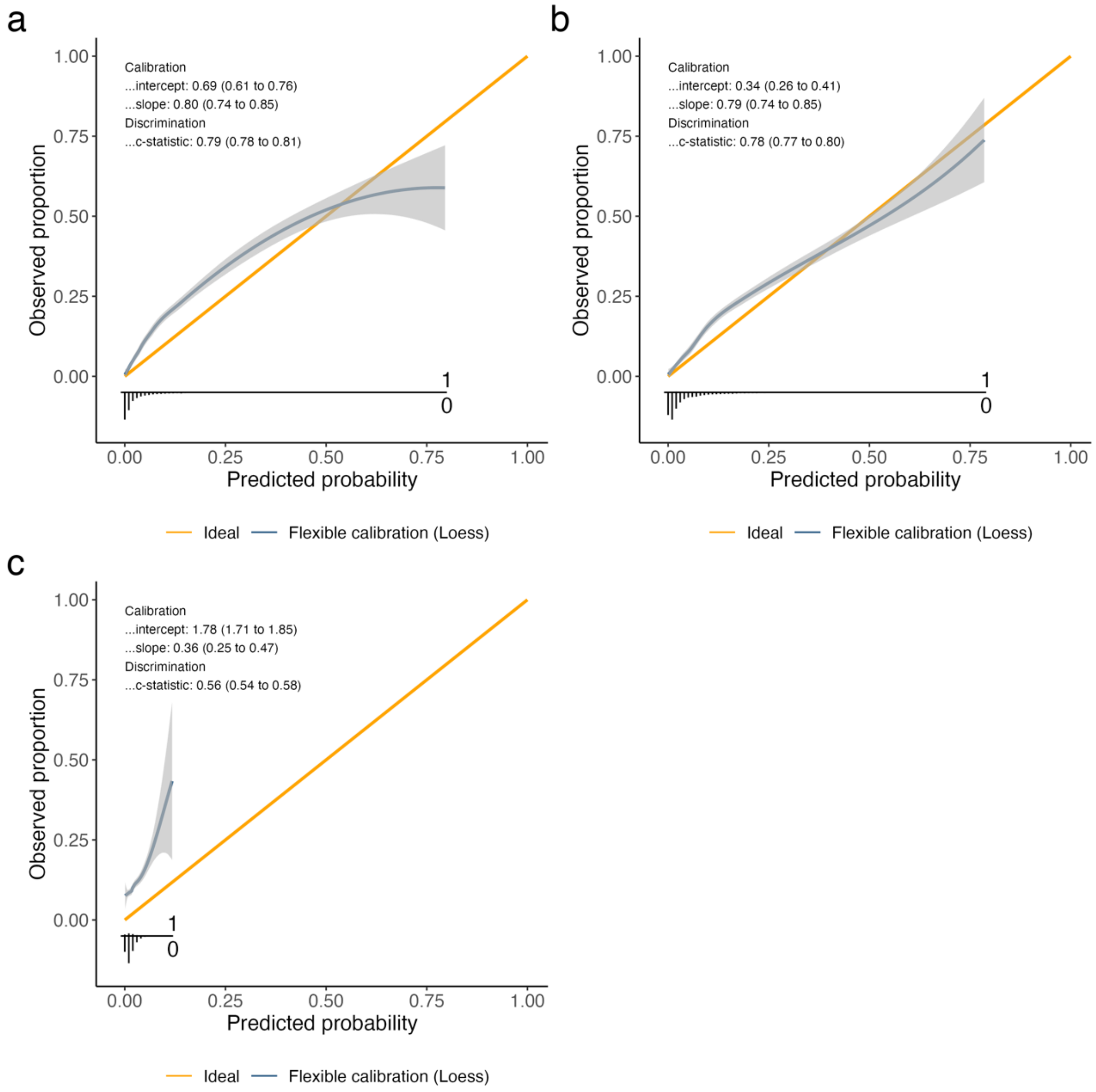

Figure S6.8.2. IST as test dataset: calibration plot of predicted outcome in the treatment group. The orange line indicates ideal calibration. The outcome variable is death at 14 days for IST and death at 4 weeks for CAST. **a**, S-learner BART. **b**, CVAE. **c**, GANITE. Abbreviations: IST: the International Stroke Trial.

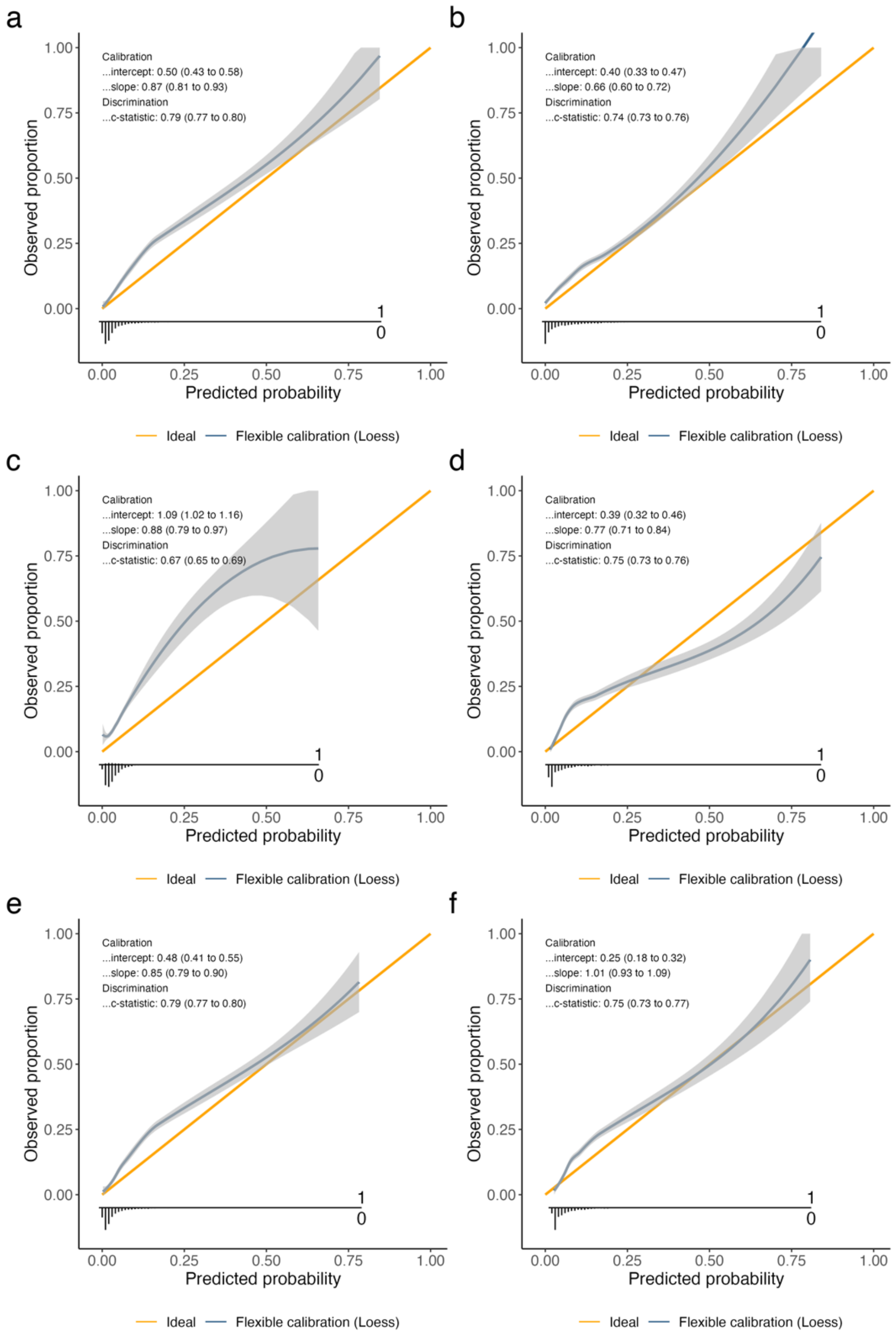

Figure S6.9.1. IST as test dataset: calibration plot of predicted outcome in the control group. The orange line indicates ideal calibration. The outcome variable is death at 14 days for IST and death at 4 weeks for CAST. **a**, T-learner Logistic Regression. **b**, T-learner Random Forest. **c**, T-learner Support Vector Machine. **d**, T-learner XGBoost. **e**, S-learner Logistic Regression. **f**, S-learner XGBoost. Abbreviations: IST: the International Stroke Trial.

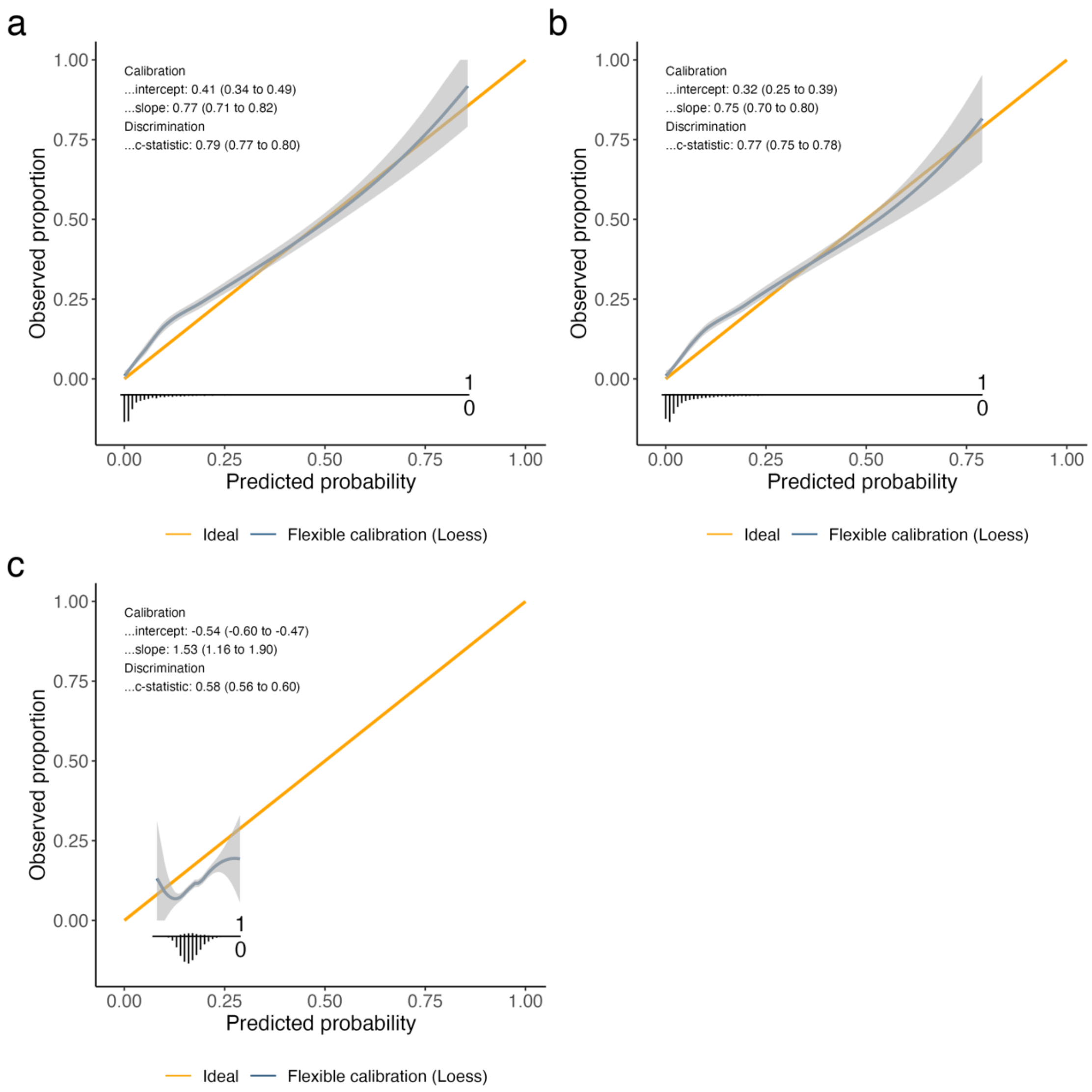

Figure S6.9.2. IST as test dataset: calibration plot of predicted outcome in the control group. The orange line indicates ideal calibration. The outcome variable is death at 14 days for IST and death at 4 weeks for CAST. **a**, S-learner BART. **b**, CVAE. **c**, GANITE. Abbreviations: IST: the International Stroke Trial.

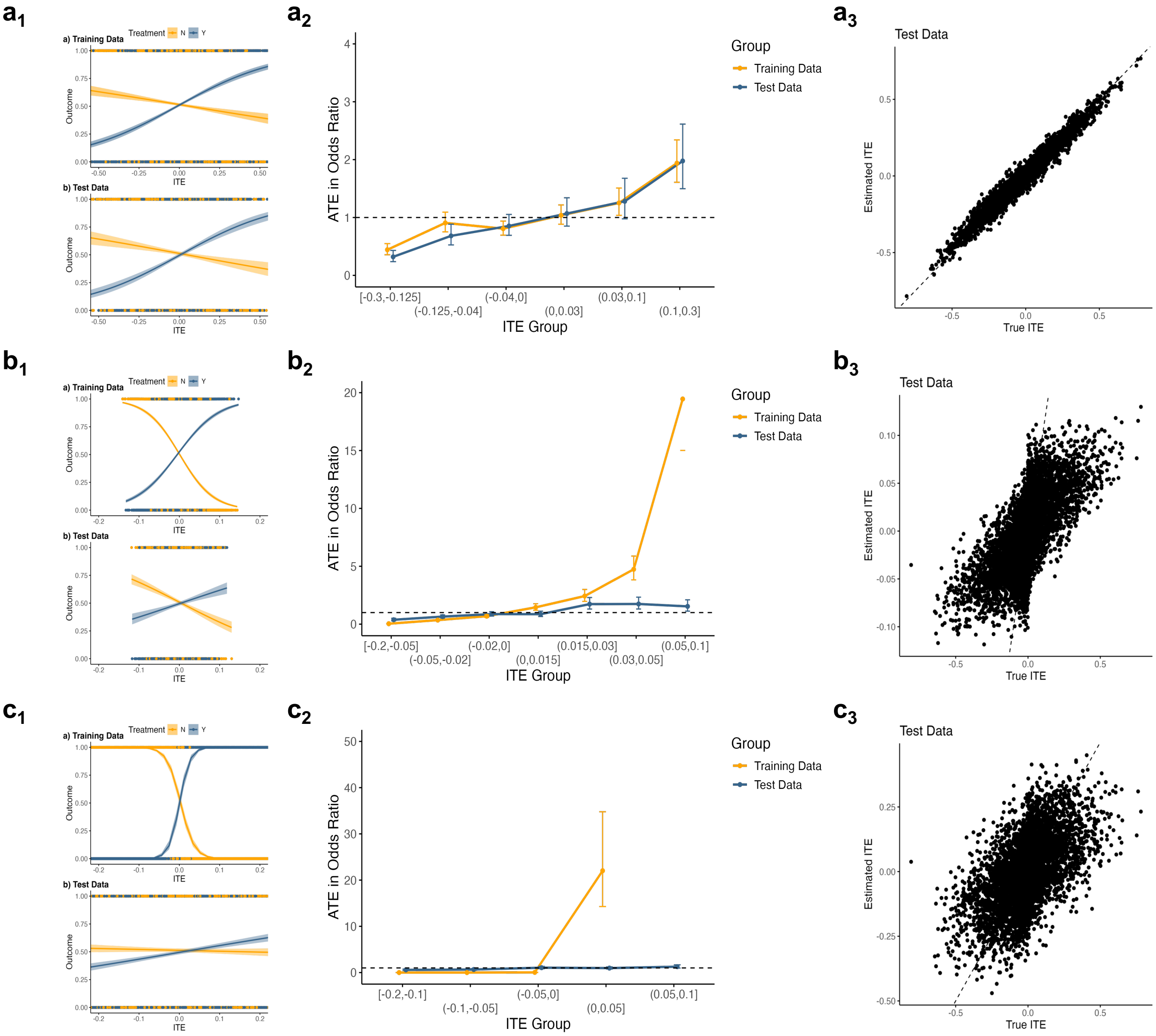

Figure S7.1.1. Simulation study: comparative analysis of causal machine learning-based individualized treatment effects. **a_1-a_3**, T learner Logistic Regression. **b_1-b_3**, Causal Forest. **c_1-c_3**, T learner Random Forest**.** **a_1,b_1,c_1**, Comparing patient outcomes against estimated ITE values between training data and test data. **a_2,b_2,c_2**, Line plots depict ATE in odds ratio within different ITE subgroups and provide the confidence intervals at 95% level. The horizontal dashed line at 1.0 means no treatment effects. **a_3,b_3,c_3**, Dot plots of estimated ITEs against the true ITEs in the test data. The black dashed line indicates ideal prediction when estimated ITEs equal to true ITEs. Abbreviations: ITE: individualized treatment effect, ATE: average treatment effect.

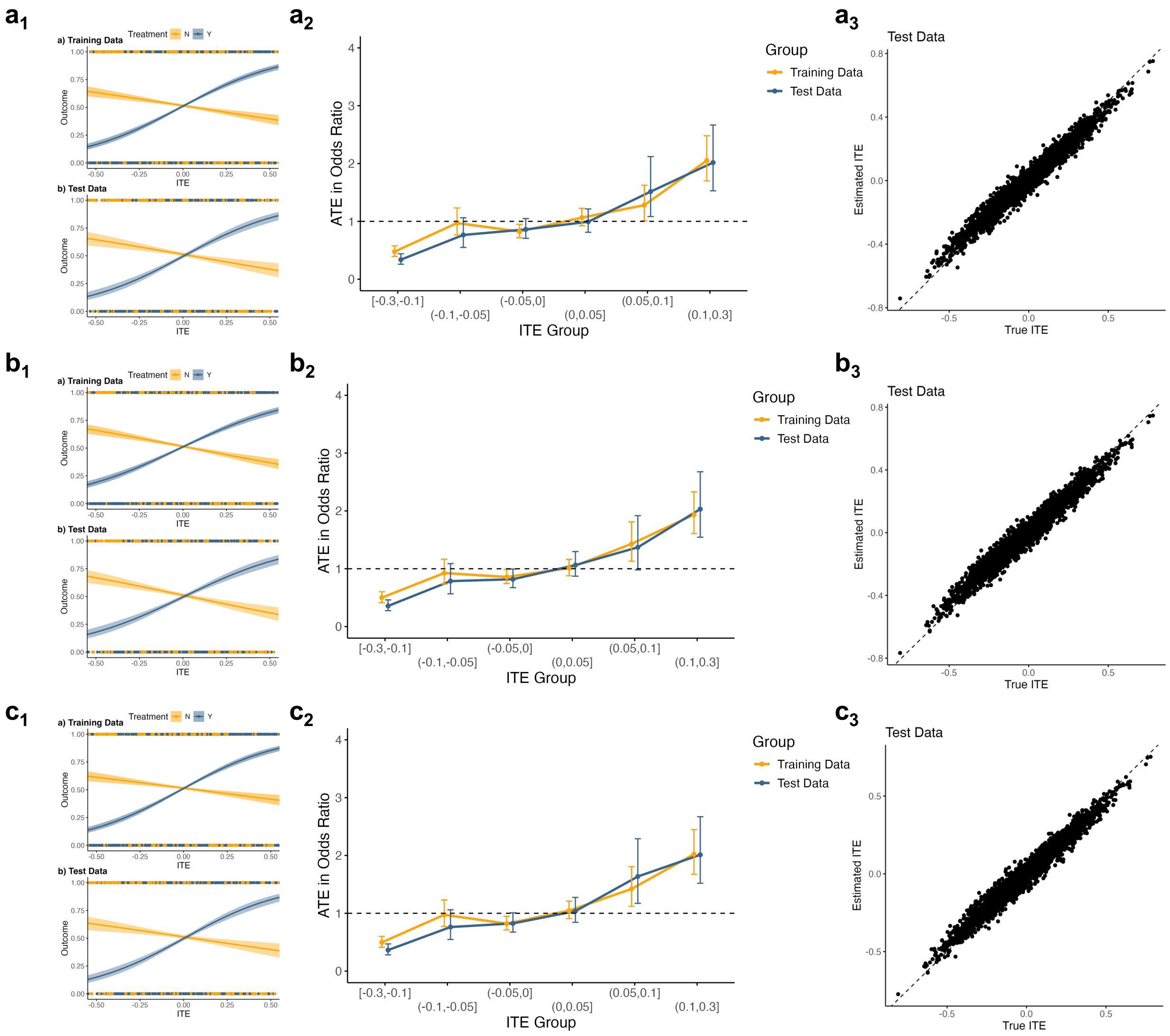

Figure S7.1.2. Simulation study: comparative analysis of causal machine learning-based individualized treatment effects. $\mathbf{a_1}$-$\mathbf{a_3}$, T learner Support Vector Machine. $\mathbf{b_1}$-$\mathbf{b_3}$, T learner Penalized Logistic Regression. $\mathbf{c_1}$-$\mathbf{c_3}$, S learner Penalized Logistic Regression**.** $\mathbf{a_1}$,$\mathbf{b_1}$,$\mathbf{c_1}$, Comparing patient outcomes against estimated ITE values between training data and test data. $\mathbf{a_2}$,$\mathbf{b_2}$,$\mathbf{c_2}$, Line plots depict ATE in odds ratio within different ITE subgroups and provide the confidence intervals at 95% level. A horizontal dashed line at 1.0 means null treatment effects. $\mathbf{a_3}$,$\mathbf{b_3}$,$\mathbf{c_3}$, Dot plots of estimated ITEs against the true ITEs in the test data. The black dashed line indicates ideal prediction when estimated ITEs equal to true ITEs. Abbreviations: ITE: individualized treatment effect, ATE: average treatment effect.

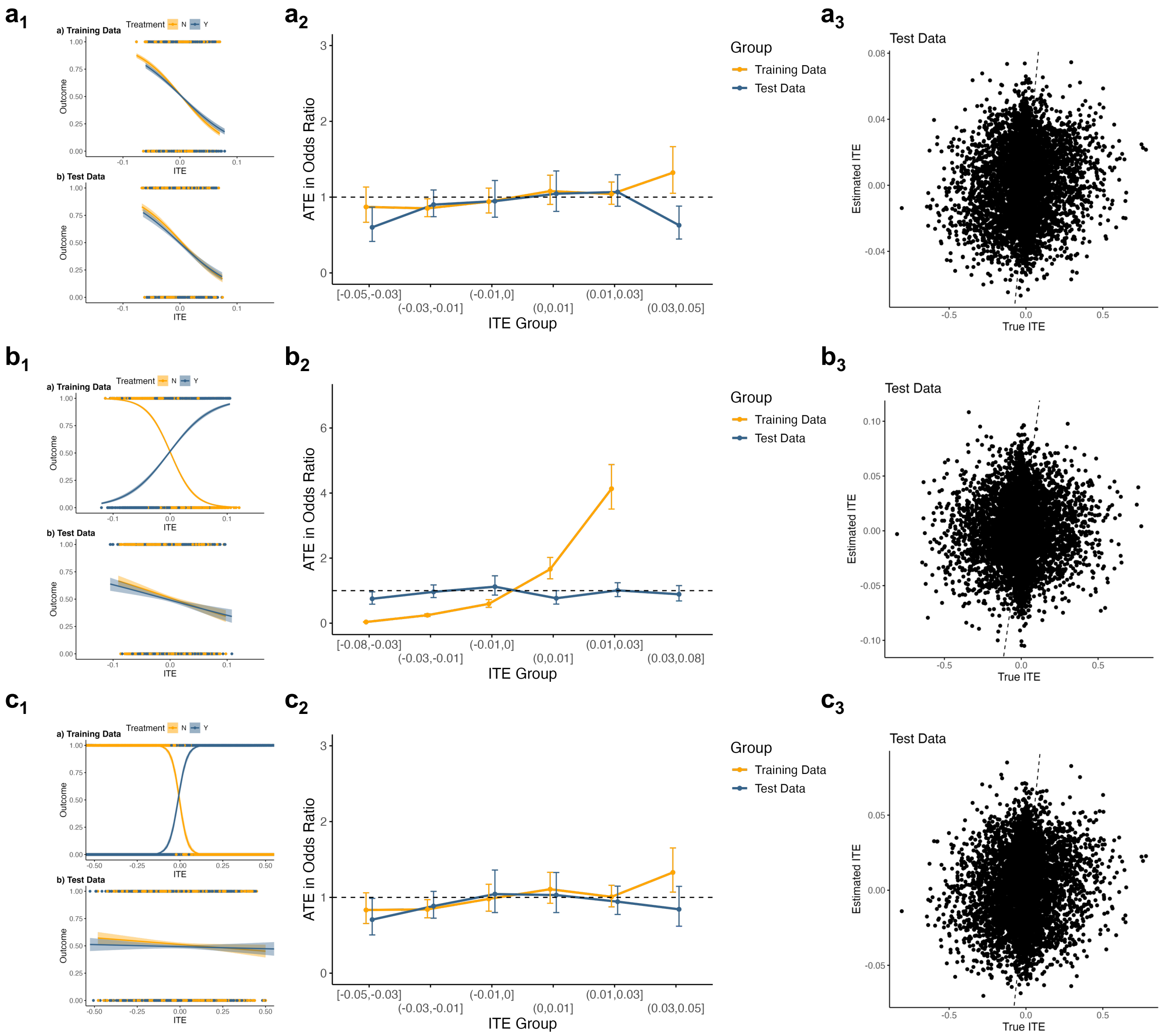

Figure S7.2.1. Simulation study: comparative analysis of causal machine learning-based individualized treatment effects. **a_1-a_3**, T learner Logistic Regression. **b_1-b_3**, Causal Forest. **c_1-c_3**, T learner Random Forest**.** **a_1,b_1,c_1**, Comparing patient outcomes against estimated ITE values between training data and test data. **a_2,b_2,c_2**, Line plots depict ATE in odds ratio within different ITE subgroups and provide the confidence intervals at 95% level. A horizontal dashed line at 1.0 means null treatment effects. **a_3,b_3,c_3**, Dot plots of estimated ITEs against the true ITEs in the test data. The black dashed line indicates ideal prediction when estimated ITEs equal to true ITEs. Abbreviations: ITE: individualized treatment effect, ATE: average treatment effect.

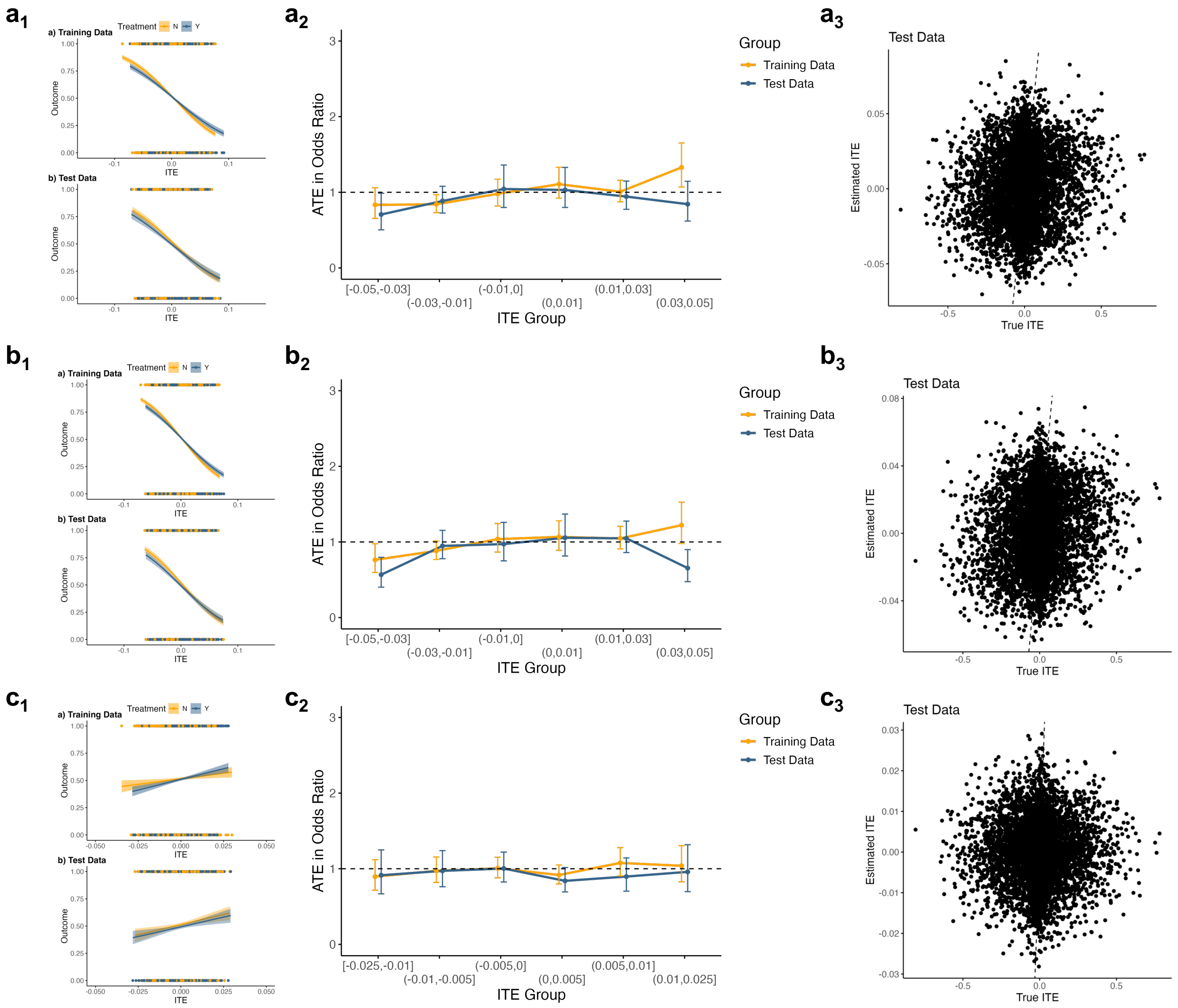

Figure S7.2.2. Simulation study: comparative analysis of causal machine learning-based individualized treatment effects. **a_1-a_3**, T learner Support Vector Machine. **b_1-b_3**, T learner Penalized Logistic Regression. **c_1-c_3**, S learner Penalized Logistic Regression**.** **a_1,b_1,c_1**, Comparing patient outcomes against estimated ITE values between training data and test data. **a_2,b_2,c_2**, Line plots depict ATE in odds ratio within different ITE subgroups and provide the confidence intervals at 95% level. A horizontal dashed line at 1.0 means null treatment effects. **a_3,b_3,c_3**, Dot plots of estimated ITEs against the true ITEs in the test data. The black dashed line indicates ideal prediction when estimated ITEs equal to true ITEs. Abbreviations: ITE: individualized treatment effect, ATE: average treatment effect.

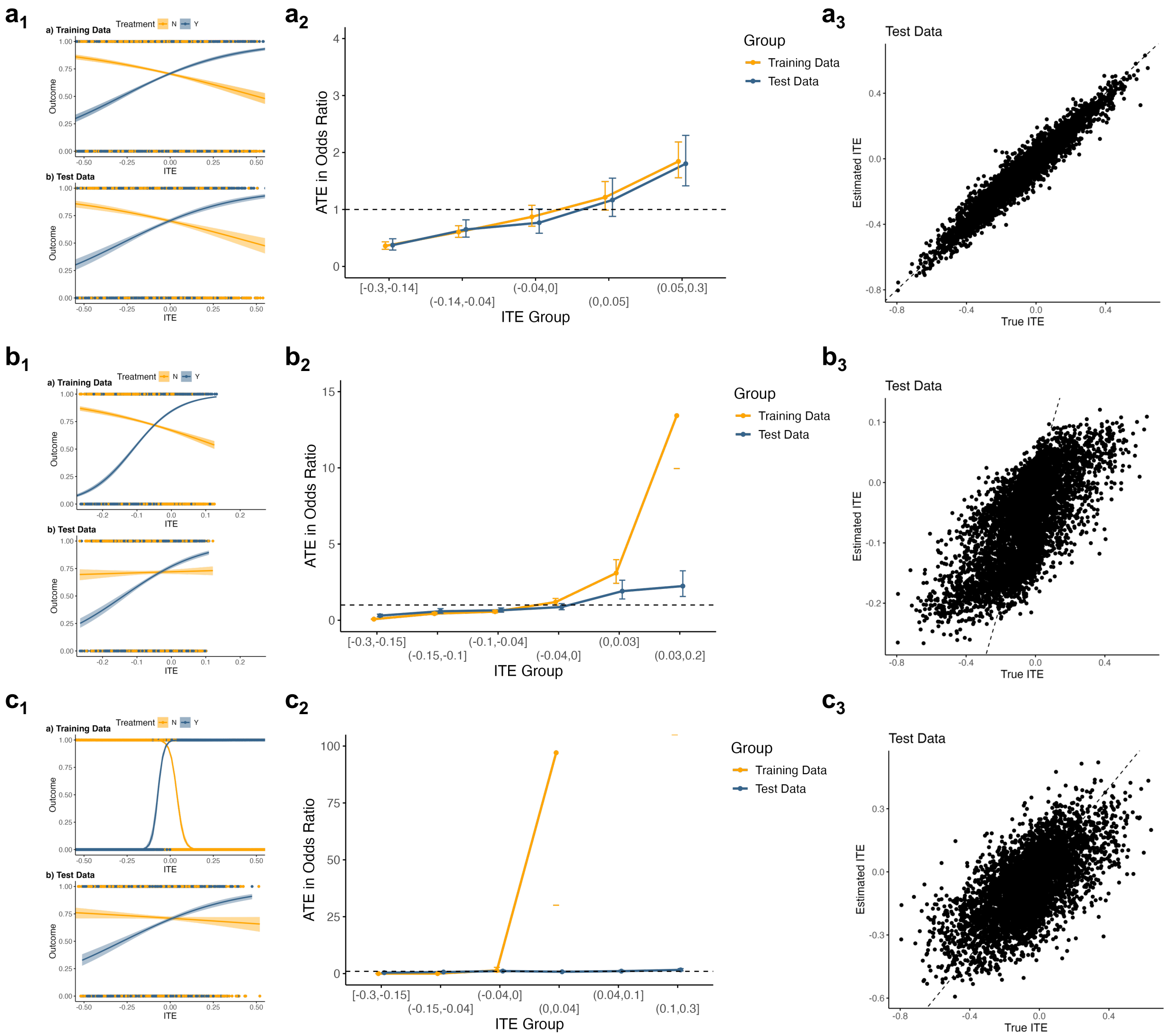

Figure S7.3.1. Simulation study: comparative analysis of causal machine learning-based individualized treatment effects. **a_1-a_3**, T learner Logistic Regression. **b_1-b_3**, Causal Forest. **c_1-c_3**, T learner Random Forest**.** **a_1,b_1,c_1**, Comparing patient outcomes against estimated ITE values between training data and test data. **a_2,b_2,c_2**, Line plots depict ATE in odds ratio within different ITE subgroups and provide the confidence intervals at 95% level. A horizontal dashed line at 1.0 means null treatment effects. **a_3,b_3,c_3**, Dot plots of estimated ITEs against the true ITEs in the test data. The black dashed line indicates ideal prediction when estimated ITEs equal to true ITEs. Abbreviations: ITE: individualized treatment effect, ATE: average treatment effect.

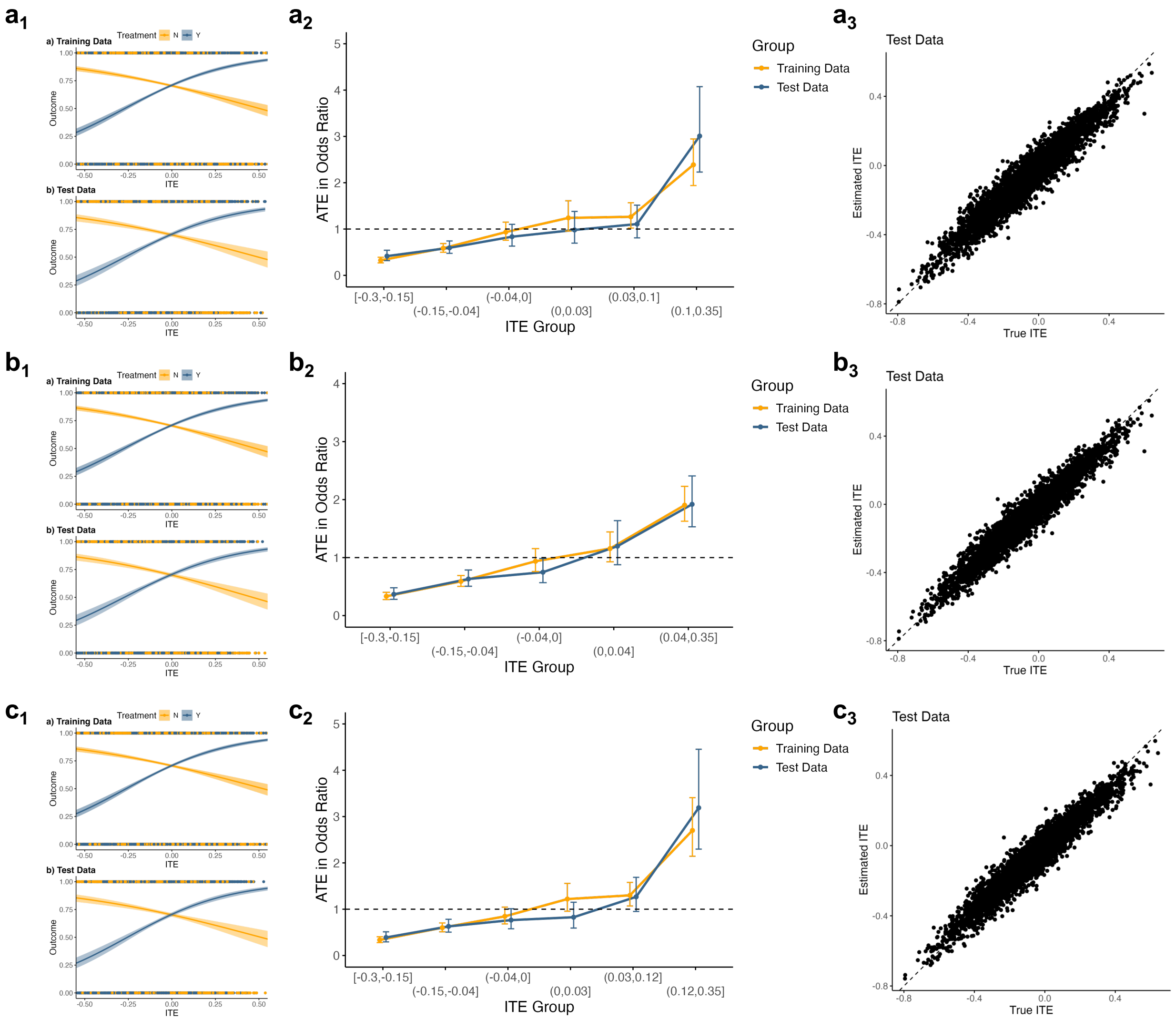

Figure S7.3.2. Simulation study: comparative analysis of causal machine learning-based individualized treatment effects. **a_1-a_3**, T learner Support Vector Machine. **b_1-b_3**, T learner Penalized Logistic Regression. **c_1-c_3**, S learner Penalized Logistic Regression**.** **a_1,b_1,c_1**, Comparing patient outcomes against estimated ITE values between training data and test data. **a_2,b_2,c_2**, Line plots depict ATE in odds ratio within different ITE subgroups and provide the confidence intervals at 95% level. A horizontal dashed line at 1.0 means null treatment effects. **a_3,b_3,c_3**, Dot plots of estimated ITEs against the true ITEs in the test data. The black dashed line indicates ideal prediction when estimated ITEs equal to true ITEs. Abbreviations: ITE: individualized treatment effect, ATE: average treatment effect.

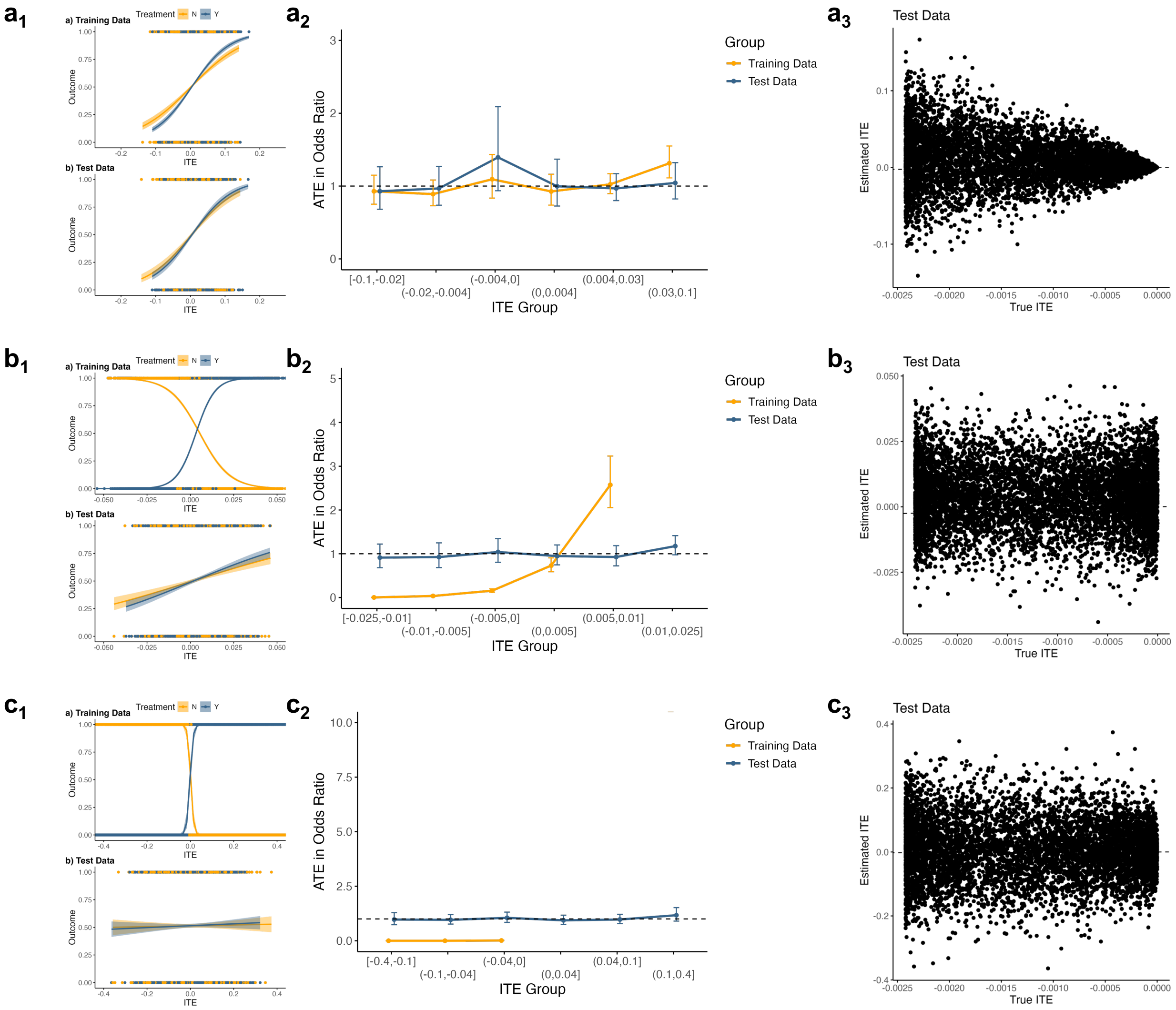

Figure S7.4.1. Simulation study: comparative analysis of causal machine learning-based individualized treatment effects. **a$_1$-a$_3$**, T learner Logistic Regression. **b$_1$-b$_3$**, Causal Forest. **c$_1$-c$_3$**, T learner Random Forest**.** **a$_1$,b$_1$,c$_1$**, Comparing patient outcomes against estimated ITE values between training data and test data. **a$_2$,b$_2$,c$_2$**, Line plots depict ATE in odds ratio within different ITE subgroups and provide the confidence intervals at 95% level. The horizontal dashed line at 1.0 means no treatment effects. **a$_3$,b$_3$,c$_3$**, Dot plots of estimated ITEs against the true ITEs in the test data. The black dashed line indicates ideal prediction when estimated ITEs equal to true ITEs. Abbreviations: ITE: individualized treatment effect, ATE: average treatment effect.

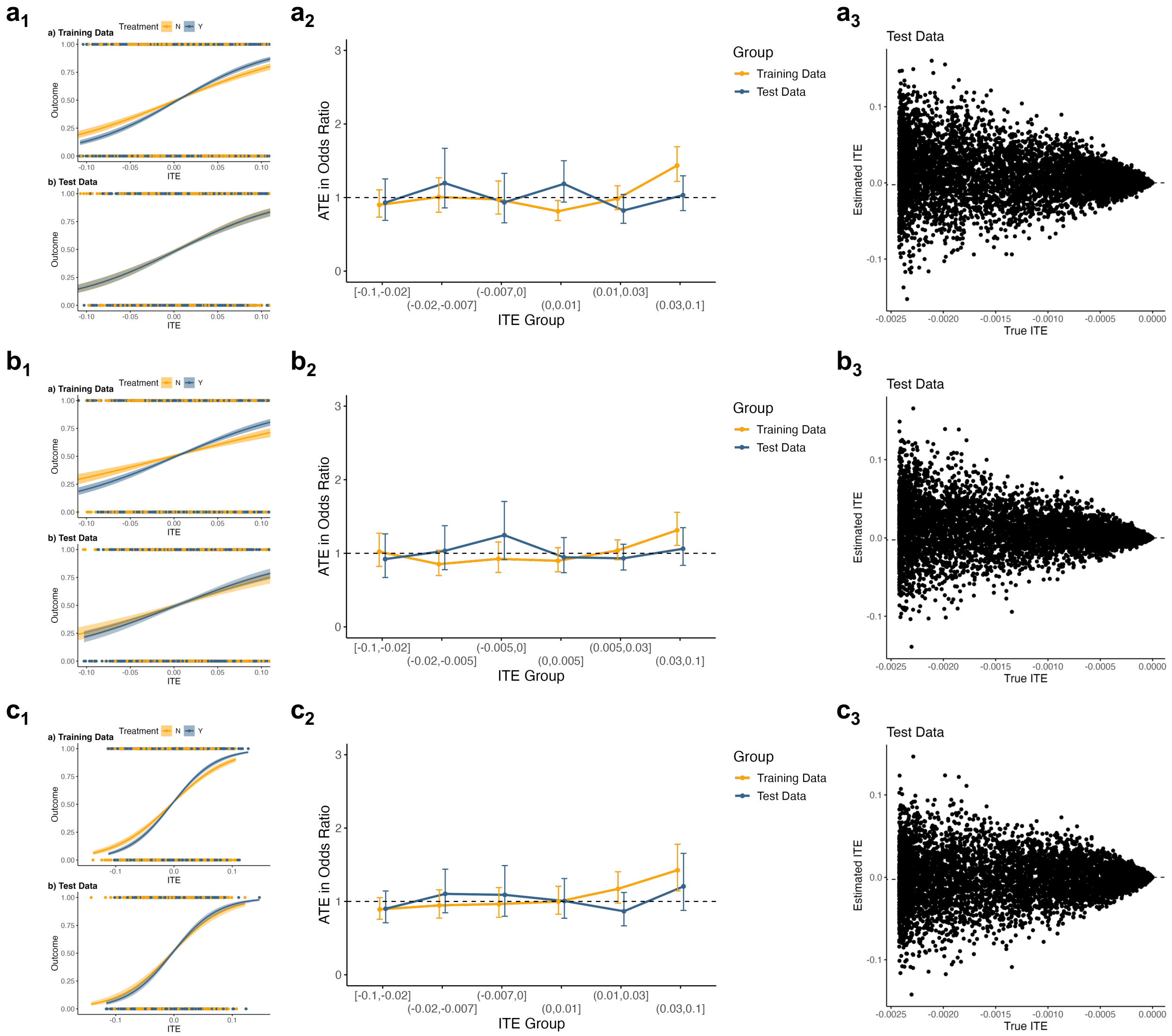

Figure S7.4.2. Simulation study: comparative analysis of causal machine learning-based individualized treatment effects. **a_1-a_3**, T learner Support Vector Machine. **b_1-b_3**, T learner Penalized Logistic Regression. **c_1-c_3**, S learner Penalized Logistic Regression**.** **a_1,b_1,c_1**, Comparing patient outcomes against estimated ITE values between training data and test data. **a_2,b_2,c_2**, Line plots depict ATE in odds ratio within different ITE subgroups and provide the confidence intervals at 95% level. A horizontal dashed line at 1.0 means null treatment effects. **a_3,b_3,c_3**, Dot plots of estimated ITEs against the true ITEs in the test data. The black dashed line indicates ideal prediction when estimated ITEs equal to true ITEs. Abbreviations: ITE: individualized treatment effect, ATE: average treatment effect.
