# Supplementary material for: Machine learning methods for estimating personalized treatment effects—insights on validity from two large trials
Source: Am J Epidemiol. 2026 Mar 20;195(8):2263–72. doi: 10.1093/aje/kwag065 (PMC13437088; doi:10.1093/aje/kwag065)
Supplement: Supplementary file 1 [file web_material_kwag065.zip › supp_materials_references_Final (included in supp materials pdf).docx]

**Appendix References**

1.	Feuerriegel S, Frauen D, Melnychuk V, et al. Causal machine learning for predicting treatment outcomes. *Nat Med*. 2024;30(4):958-968. doi:10.1038/s41591-024-02902-1

2.	Künzel SR, Sekhon JS, Bickel PJ, Yu B. Metalearners for estimating heterogeneous treatment effects using machine learning. *Proceedings of the National Academy of Sciences*. 2019;116(10):4156-4165. doi:10.1073/pnas.1804597116

3.	Nie X, Wager S. Quasi-oracle estimation of heterogeneous treatment effects. *Biometrika*. 2021;108(2):299-319. doi:10.1093/biomet/asaa076

4.	Kennedy EH. Towards optimal doubly robust estimation of heterogeneous causal effects. *Electronic Journal of Statistics*. 2023;17(2):3008-3049. doi:10.1214/23-EJS2157

5.	Athey S, Imbens G. Recursive partitioning for heterogeneous causal effects. *Proc Natl Acad Sci USA*. 2016;113(27):7353-7360. doi:10.1073/pnas.1510489113

6.	Wager S, Athey S. Estimation and Inference of Heterogeneous Treatment Effects using Random Forests. *Journal of the American Statistical Association*. 2018;113(523):1228-1242. doi:10.1080/01621459.2017.1319839

7.	Seibold H, Zeileis A, Hothorn T. Model-Based Recursive Partitioning for Subgroup Analyses. *The International Journal of Biostatistics*. 2016;12(1):45-63. doi:10.1515/ijb-2015-0032

8.	Seibold H, Zeileis A, Hothorn T. model4you: An R Package for Personalised Treatment Effect Estimation. *Journal of Open Research Software*. 2019;7(1). doi:10.5334/jors.219

9.	Hahn PR, Murray JS, Carvalho CM. Bayesian Regression Tree Models for Causal Inference: Regularization, Confounding, and Heterogeneous Effects (with Discussion). *Bayesian Analysis*. 2020;15(3):965-1056. doi:10.1214/19-BA1195

10.	Jawadekar N, Kezios K, Odden MC, et al. Practical Guide to Honest Causal Forests for Identifying Heterogeneous Treatment Effects. *Am J Epidemiol*. 2023;192(7):1155-1165. doi:10.1093/aje/kwad043

11.	Cui Y, Kosorok MR, Sverdrup E, Wager S, Zhu R. Estimating heterogeneous treatment effects with right-censored data via causal survival forests. *J R Stat Soc Ser B Stat Methodol*. 2023;85(2):179-211. doi:10.1093/jrsssb/qkac001

12.	Sohn K, Lee H, Yan X. Learning Structured Output Representation using Deep Conditional Generative Models. In: *Advances in Neural Information Processing Systems*. Vol 28. Curran Associates, Inc.; 2015. Accessed December 9, 2024. https://proceedings.neurips.cc/paper_files/paper/2015/file/8d55a249e6baa5c06772297520da2051-Paper.pdf

13.	Yoon J, Jordon J, Schaar M van der. GANITE: Estimation of Individualized Treatment Effects using Generative Adversarial Nets. In: 2018. Accessed December 9, 2024. https://openreview.net/forum?id=ByKWUeWA-
